# Astrocytic junctional adhesion molecule-A regulates T-cell entry past the glia limitans to promote central nervous system autoimmune attack

**DOI:** 10.1093/braincomms/fcac044

**Published:** 2022-02-18

**Authors:** Mario Amatruda, Candice Chapouly, Viola Woo, Farinaz Safavi, Joy Zhang, David Dai, Anthony Therattil, Chang Moon, Jorge Villavicencio, Alexandra Gordon, Charles Parkos, Sam Horng

**Affiliations:** Department of Neurology, Icahn School of Medicine at Mount Sinai, New York, NY, USA; Univ. Bordeaux, Inserm, Biology of Cardiovascular Diseases, U1034, CHU de Bordeaux, F-33604 Pessac, France; Department of Neurology, Icahn School of Medicine at Mount Sinai, New York, NY, USA; National Institute of Neurological Disorders and Stroke, National Institutes of Health, Bethesda, MD, USA; University of Virginia School of Medicine, Charlottesville, VA, USA; Department of Neurology, Perelman School of Medicine at the University of Pennsylvania, Philadelphia, PA, USA; New York Medical College, Valhalla, NY, USA; Department of Neurology, Icahn School of Medicine at Mount Sinai, New York, NY, USA; Department of Neurology, Icahn School of Medicine at Mount Sinai, New York, NY, USA; Miller School of Medicine at University of Miami, Miami, FL, USA; Department of Pathology, University of Michigan, Ann Arbor, MI, USA; Department of Neurology, Icahn School of Medicine at Mount Sinai, New York, NY, USA; Department of Neuroscience, Icahn School of Medicine at Mount Sinai, New York, NY, USA

**Keywords:** astrocyte immune cell cross-talk, junctional adhesion molecule-A, experimental autoimmune encephalomyelitis, multiple sclerosis, glia limitans

## Abstract

Contact-mediated interactions between the astrocytic endfeet and infiltrating immune cells within the perivascular space are underexplored, yet represent potential regulatory check-points against CNS autoimmune disease and disability. Reactive astrocytes upregulate junctional adhesion molecule-A, an immunoglobulin-like cell surface receptor that binds to T cells via its ligand, the integrin, lymphocyte function-associated antigen-1. Here, we tested the role of astrocytic junctional adhesion molecule-A in regulating CNS autoinflammatory disease. In cell co-cultures, we found that junctional adhesion molecule-A-mediated signalling between astrocytes and T cells increases levels of matrix metalloproteinase-2, C–C motif chemokine ligand 2 and granulocyte-macrophage colony-stimulating factor, pro-inflammatory factors driving lymphocyte entry and pathogenicity in multiple sclerosis and experimental autoimmune encephalomyelitis, an animal model of CNS autoimmune disease. In experimental autoimmune encephalomyelitis, mice with astrocyte-specific *JAM-A* deletion (*mGFAP:CreJAM-A^fl/fl^*) exhibit decreased levels of matrix metalloproteinase-2, reduced ability of T cells to infiltrate the CNS parenchyma from the perivascular spaces and a milder histopathological and clinical course of disease compared with wild-type controls (*JAM-A^fl/fl^*). Treatment of wild-type mice with intraperitoneal injection of soluble junctional adhesion molecule-A blocking peptide decreases the severity of experimental autoimmune encephalomyelitis, highlighting the potential of contact-mediated astrocyte–immune cell signalling as a novel translational target against neuroinflammatory disease.

## Teaser

Amatruda *et al*. report that the astrocytic immune cell receptor, junctional adhesion molecule-A, increases levels of matrix metalloproteinase-2 and promotes T-cell entry into the CNS during autoimmune attack. Blocking contact-mediated astrocyte immune cell signals represents a novel therapeutic approach against multiple sclerosis and other CNS autoinflammatory diseases.

## Graphical Abstract

**Graphical Abstract:**
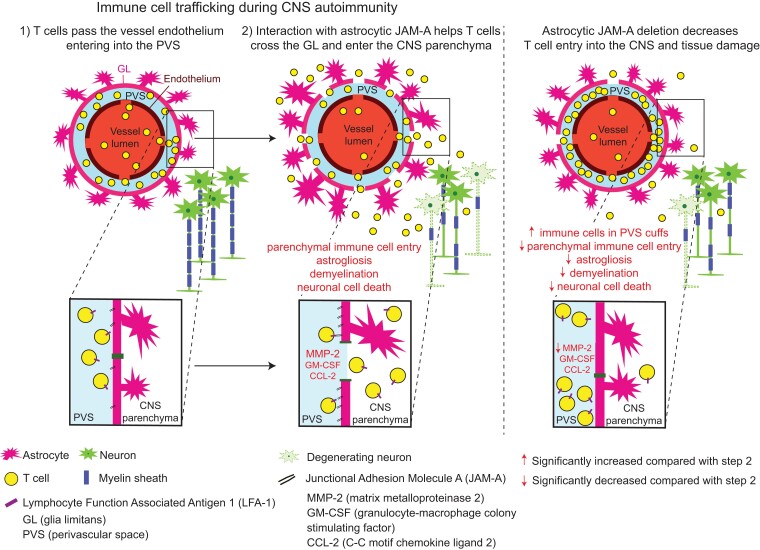

## Introduction

In multiple sclerosis and other autoimmune diseases of the CNS, immune cells inappropriately invade the CNS from the bloodstream and drive inflammatory lesion formation.^1,2^ CNS entry is a two-step process through a specialized structure termed the blood–brain barrier (BBB): first, immune cells cross the endothelial wall, using contact-mediated interactions with the endothelial surface to traffic into an intermediary compartment termed the perivascular space (PVS).^3–5^ Within the PVS, immune cells encounter the glia limitans (GL), a barrier comprised of astrocytic endfeet through which cells must subsequently cross to access the CNS parenchyma and inflict damage.^6^

Interactions between the astrocytic endfeet and immune cells within the PVS have been minimally explored despite their potential significance in regulating the autoimmune response. Cross-talk is known to involve leucocyte matrix metalloproteinase-2 (MMP-2) and matrix metalloproteinase-9 (MMP-9) in degrading PVS basement membranes, enabling the parenchymal entry of infiltrating immune cells during experimental autoimmune encephalomyelitis (EAE).^7–9^ Astrocytic vascular cell adhesion molecule-1 (VCAM-1) acts as a tumour necrosis factor receptor 1–induced cell adhesion molecule and is critical for immune cell trafficking past the GL and into the CNS parenchyma during EAE.^10–12^ Astrocytic VCAM-1 is also modulated in a region-specific manner by interleukin-17 (IL-17) producing effector T cells (Th17) accounting for regional differences in immune cell infiltration.^13^ Recently, tissue resident cluster of differentiation 8 (CD8)-positive T cells were found within multiple sclerosis lesions to express the programmed cell death protein 1 (PD-1) while reactive astrocyte endfeet express PD-1 ligand, suggestive of potential inhibitory interactions within the PVS.^14^ Therefore, both soluble and contact-mediated signals between astrocytes and immune cells within the PVS have the potential to regulate CNS autoinflammatory disease.

We previously reported^15^ that reactive astrocytes upregulate junctional adhesion molecule-A (JAM-A), an immunoglobulin-like cell surface receptor, in response to the pro-inflammatory cytokine, interleukin-1 beta (IL-1β) *in vitro* as well as in *in vivo* models of CNS inflammation. JAM-A has a dual role: (i) initiating and stabilizing tight junction complexes via homophilic binding between identical cell types^16–18^ and (ii) serving as an immune cell surface receptor via heterophilic binding to lymphocyte function-associated antigen-1 on T cells and monocytes.^19^ In gut and CNS vascular endothelium, JAM-A binds to immune cells to induce intracellular signal transduction pathways and promote transmigration through the endothelial layer, ultimately leading to a pro-inflammatory, tissue damaging state.^4,20–25^

We hypothesized that astrocytic JAM-A interacts with immune cells within the PVS to promote effector pathways of CNS inflammation and tissue damage. Here, we focused on the T-cell population given its central role in driving pathogenesis of EAE, an animal model of CNS autoimmune demyelinating disease. We first characterized the effects of astrocytic JAM-A on proteases and cytokines implicated in EAE and multiple sclerosis pathogenesis. Then, using a genetic mouse model in which JAM-A is selectively deleted from reactive astrocytes (*mGFAP:CreJAM-A^fl/fl^*) compared with unaffected (*JAM-A^fl/fl^*) controls,^26,27^ we investigated how astrocytic JAM-A regulates lesion pathology in two models of CNS inflammation and its effects on T-cell trafficking, the inflammatory proteome and clinical disability in EAE. Additionally, we tested the therapeutic potential of an exogenously administered JAM-A blocking peptide (JAM-Ap) in EAE.

## Materials and methods

### Cell mono-culture: astrocytes

Primary human foetal astrocytes were obtained from Lonza (CC-2565) and grown to confluence on glass confocal plates (Mat-Tek, P35GC-1.5-14C) in astrocyte growth medium (AGM). AGM comprised MCDB 131 Medium (Gibco 10372-019) and Astrocyte BulletKit factors (Lonza, CC-3186), providing for 3% FBS, 2 mM l-glutamine, 30 µg/ml gentamicin and 15 ng/ml amphotericin (GA-1000), 70 µM ascorbic acid, 3 ng/ml rhEGF and 7.5 µg/ml insulin. Astrocytes were then pre-treated with MCDB 131 alone for 6 h and then treated with 20 ng/ml human recombinant IL-1β, 100 ng/ml CCL-2, IL-1β + CCL-2 or vehicle for 24 h and then were fixed in ice-cold 4% paraformaldehyde (PFA) in 1× phosphate buffer saline (PBS) for 30 min then processed for immunocytochemical staining.

### Cell co-culture: astrocytes

Primary human foetal astrocytes were plated to 70% confluence on a 20 cm^2^ tissue culture dish (Corning, 353003) in AGM. Astrocytes were washed with PBS twice, dissociated gently with 0.05% trypsin, centrifuged, resuspended and nucleofected with 2 µM siRNA of either non-targeting (*siNT*) or *JAM-A* (*siJAM-A*) targeting sequences, as detailed below. Transfected astrocytes were re-plated and allowed to grow for 24 h in AGM. Astrocytes were then serum-starved in MCDB 131 for 24 h and then treated with 20 ng/ml IL-1β for 24 h. MCDB 131 was refreshed and astrocytes were then paired with 1–2 × 10^6^ isolated CD3^+^ T lymphocytes on a 20 cm^2^ tissue culture dish for 24 h. After co-culture, CD3^+^ T lymphocytes, astrocytes and supernatants were separated, sonicated and stored at −20°C for protease and cytokine array experiments.

### Cell co-culture: T lymphocytes

Human T lymphocytes were extracted from human blood of healthy adult donors freshly collected in lavender K2-EDTA tubes (BD #367861). Briefly, peripheral blood mononuclear cells (PBMCs) were isolated from whole blood samples using density centrifugation with Ficoll-Paque PLUS (GE Healthcare). Six millilitres of whole blood were diluted with an equal volume of HBSS (Mediatech Inc.) and layered onto 15 ml tubes prefilled with 4 ml of density gradient medium. Tubes were centrifuged for 1 h at 620 relative centrifugal force (rcf). PBMCs were collected from their density gradient layer using a transfer pipette, washed in HBSS (Mediatech Inc.) and centrifuged for 15–20 min at 620 rcf × 2. The PBMC pellet was resuspended in eluent buffer and processed with a magnetic labelling and separation protocol using a human pan-T-cell protein (CD3^+^) (Miltenyi, 130-096-535). Cells were then activated in lymphocyte growth medium [comprised of RPMI 1640 (Gibco), 10% FBS, 2 mM l-glutamine, 1% 2-mercaptoethanol] at 37°C for 72 h with 4 µg/ml anti-CD28 (eBioscience 16-0298-85) on 20 cm^2^ tissue culture dishes pre-treated with 7 µg/ml anti-CD3 (eBioscience 16-0037-85) in PBS at 37°C for 2 h. After activation, T lymphocytes were centrifuged and 1–2 × 10^6^ cells were applied to astrocyte cultures for 24 h before sample separation and processing, as above.

### Human protease and cytokine arrays

Reactive astrocyte and CD3^+^ T-lymphocyte co-cultures were prepared as outlined above. Culture medium supernatant with CD3^+^ T lymphocytes was aspirated from co-cultures after 24 h. Aspirant was centrifuged at 620 rcf for 7 min, then the supernatant was stored at 20°C. The CD3^+^ T-lymphocyte pellet was reconstituted and harvested in cell lysis buffer, which was sonicated and then stored at 20°C. Adherent astrocytes from the 20 cm^2^ tissue culture dish were harvested in cell lysis buffer, sonicated and then stored at 20°C. Supernatant (500 µl), T-lymphocyte (100 µg) and astrocyte (100 µg) samples were then applied to human protease (R&D, ARY021B) and cytokine (R&D, ARY005B) ELISA array kits per the manufacturer’s instructions in three biological replicates. Quantification of protease signal was performed by densitometry as follows: non-saturated developed films were scanned using a Canon LiDE scanner (Canon USA), and the mean pixel density of each duplicate array probe was measured using the ImageJ software (NIH). Data were standardized to three duplicated reference probes, and the relative change between *siNT-* and *siJAM-A*-treated conditions was calculated and then compared with the relative change in reference probe signal. Comparisons included all array probes initially and then those without visually detectable signals were excluded and not considered biologically significant.

### Mouse proteome arrays

Spinal cord tissue from wild-type (WT) (*n* = 4) and conditional knock-out (CKO) (*n* = 3) mice were harvested at 5 days from EAE disease onset, homogenized in PBS with protease inhibitors and stored at −80°C before thawing for experiments. Samples were quantified for protein concentration and 200 µg applied to mouse proteome ELISA profiler arrays (R&D, ARY028) per the manufacturer’s instructions in four and three biological replicates. Quantification of protease signal and analysis of the relative change between WT and CKO were performed as outlined in the ‘Human protease and cytokine arrays’ section.

### Chemical and protein reagents

Human IL-1β and CCL-2 were purchased from PeproTech and used at 20 and 10 ng/ml, respectively, for mono-culture experiments described above.

### JAM-A blocking peptide

JAM-Ap and control peptide were synthesized to order at >95% purity from New England Biopeptide with the following sequences: NPKSTRAFSNDDYVLNPTTG for JAM-Ap and NLFSVDTPNGKTASDNYPRT for control, as designed and characterized by a previous group.^20^ Daily intraperitoneal injection of 1 µg in 0.1 ml of sterile 0.9% NaCl starting on Day 7 post-EAE immunization was performed in EAE experiments.

### Antibodies

Catalogue numbers and concentrations of all antibodies are as follows. Anti-GFAP (130300, rat, 1:200), anti-occludin (711500, rabbit, 1:125) and anti-IgG (A11029, mouse, 1:100) were from Invitrogen. Anti–JAM-A (sc53623, mouse, 1:100) was from Santa Cruz Biotechnology. Fluoromyelin was from ThermoFisher (F34651, 1:300). Anti-fibrinogen (A0080, rabbit, 1:150) was from Dako. Anti-CD3 (16-0037-85, mouse, 1:100), anti-CD4 (14-9766-82, rat, 1:100), anti-CD31 (550274, rat, 1:100), anti-CD45 (550539, rat, 1:100) were from eBioscience. Anti-CD4 (ab183685, mouse, 1:50) was from Abcam. Anti-NeuN (MAB377, mouse, 1:100), anti-Olig2 (MABN50, mouse, 1:500) and anti-AQP4 (AB3594, rabbit, 1:200) were from Millipore. Anti-laminin (L9393, rabbit, 1:200) was from Sigma–Aldrich. Anti-Iba1 (109-19741, rabbit, 1:500) was from Wako.

### Small interfering RNA

Human astrocyte cultures were nucleofected with siRNA (2 µM) with non-targeting sequences (*siNT*) or *JAM-A* targeting sequences *(siJAM-A)* (Thermo Scientific Dharmacon, siGENOME SMART pool), using an Amaxa nucleofector (programme A033) with the Basic Glial Kit (Amaxa). The extent and specificity of gene silencing were confirmed by immunoblotting as reported in a previous study.^15^

### Mice

*mGfap-Cre* [B6.Cg-Tg(Gfap-cre)73.12Mvs/J] mice were genetically engineered in the laboratory of Michael Sofroniew (University of California, Los Angeles, CA, USA) and are available for purchase from Jackson laboratories (https://www.jax.org/strain/012886). *Cre* expression is astrocyte-specific except in areas of adult neurogenesis, where it is also observed in some neural progenitors.^27^  *JAM-A^fl/fl^* mice were obtained from Charles Parkos (University of Michigan, Ann Arbor, MI, USA) and Terence Dermody (University of Pittsburgh, Pittsburgh, PA, USA).^26,28^ For all experiments, *mGfap-Cre JAM-A^fl/fl^* female mice were crossed with *JAM-A^fl/fl^* male mice to generate ∼50% *mGfap-Cre JAM-A^fl/fl^* (CKO mice) and ∼50% *JAM-A^fl/fl^* (WT) littermate controls. Selective deletion of JAM-A in glial fibrillary acidic protein (GFAP)-positive cells was described in a previous study^15^ and confirmed here. Total JAM-A knock-out (KO) mice were generated by breeding *mGfap-Cre JAM-A^fl/fl^* male mice (which express Cre in germline cells) to *JAM-A^fl/fl^* to create JAM-A*^fl/−^* mice which were then crossed to create JAM-A*^−/−^*. Genotyping primers were *mGfap-Cre* forward (GfF) ACC AGC CAG CTA TCA ACT C, reverse (GfR) TAT ACG CGT GCT AGC GAA GAT CTC CAT CTT CCA GCA G, 350 bp; *JAM-A* forward (JaKOF) TCT TTT CAC CAA TCG GAA CG, reverse (JF2R) AAA AAC TCT AGG AAC TCA CCC AGG A, band 200 bp (wt), 320 bp (flox); *JAM-A* excised forward (TS379) CCT CTC TTT TCA CCA ATC GGA, JAM-A excised reverse (TS512) TCT TCT TCA GAC GCC GAA CCT, band 489 bp. PCR conditions for all primer sets were as follows: 94°C for 4 min; 35 cycles of 94°C for 30 s, 56°C for 30 s and 72°C for 30 s; then 72°C for 10 min.

### Cortical microinjection of IL-1β expressing adenovirus

Mice (8–12 weeks old, at least five per condition per time point, on the C57BL/6 background) were anaesthetized using isoflurane and placed into a stereotactic frame (Kopf). IL-1β expressing adenovirus (AdIL-1) or AdDL70 control (AdCtrl) (10^6^ PFU) was microinjected into the cerebral cortex at *y* = 1 mm caudal to bregma, *x* = 2 mm, *z* = 1.5 mm. Animals were allowed to recover for 7 days and then were sacrificed and perfused with 10 ml of 1× PBS and 10 ml of 4% PFA in 1× PBS.

### Experimental autoimmune encephalomyelitis

Mice (males and females, 10–13 weeks old, at least eight animals per group for each experiment, on the C57BL/6 background) were subcutaneously injected, at cervical and lumbar sites, with 0.1 cc of myelin oligodendrocyte glycoprotein peptide 35–55 (MOG_35–55_) in complete Freund’s adjuvant (CFA) (1 mg/ml) followed by intraperitoneal injection of 0.1 cc pertussis toxin on Days 0 and 1 (Hooke Laboratories). Healthy control (HC) mice received the injection of CFA emulsion with no MOG_35–55_. Mice were rated daily on a standard 5-point motor scale from Days 7 to 28 after induction: 0, no symptoms; 1, floppy tail; 2, hind limb weakness (paraparesis); 3, hind limb paralysis (paraplegia); 4, forelimb and hind limb paralysis; and 5, death. The average EAE score consisted of the average score across all animals of the same genotype at Day 28 (the end of the experiment). Cumulative EAE score consisted of the average summed score per animal across all animals of the same genotype at Day 28 (the end of the experiment). The average mortality was calculated as the proportion of animals with a score of 5 by the end of the experiment (Day 28). Disease-free values were calculated by the proportion of animals who maintained a score of 0 throughout the entire experiment (Days 7–28). Three independent experiments were performed for WT and CKO and two independent experiments for KO. Raters were blind to genotype where possible; breeding conditions required separate WT and KO cages in parallel to the CKO colony, which produced both WT and CKO offspring. Three independent experiments were performed for JAM-Ap and scramble control experiments. Raters were blinded to treatment group in the treatment experiments.

### Flow cytometry

WT and CKO mice were anaesthetized and perfused with 5 ml PBS at 5 days from the onset of disease in EAE (Days 14–20 from induction). Spinal cords and spleens were collected in cold PBS and mechanically dissociated. Spleen samples were passed through a 70 μm filter, then incubated in red blood cell lysis buffer (BioLegend) for 2 min at room temperature and washed with PBS. Spinal cord samples were passed through a 100 μm filter and separated from myelin using a 60%/30% Percoll gradient. Cell suspensions were then collected, counted and subjected first to a Zombie Yellow stain (Biolegend, 423103) and washed and then followed by incubation with FITC-anti-CD3 (Biolegend, 100306) and APC/Cy7-anti-CD4 (Biolegend, 100355) antibodies in cell staining buffer (BioLegend). Cells were washed with fluorescence-activated cell sorting buffer (2% FBS in PBS), fixed and permeabilized following the manufacturer’s instructions for staining using the FIX & PERM^®^ Cell Permeabilization Kit (Invitrogen). Forward scatter and side scatter were used to gate cells excluding debris and cell aggregates, Zombie Yellow was used to exclude dead cells and then percentages of CD3^+^ cells were measured with a subsequent gate to CD4. Flow cytometry for spinal cord tissues was set to run CD3^+^ events up to 2000; total CD3^+^ counts from actual samples ranged from 298 to 1340. Flow cytometry for spleen tissue all exceeded CD3^+^ counts of 40 000. Data were acquired on the Invitrogen^™^ Attune^™^ NxT Flow Cytometer and analysed with the FCS Express software (De Novo) at the Flow Cytometry CoRE at Mount Sinai.

### Immunohistochemistry

Brains and spinal cords were dissected from animals perfused with 10 ml ice-cold 1× PBS followed by 10 ml 4% PFA-1× PBS, tissues were subjected to 2 h post-fixation in 4% PFA-1× PBS followed by storage in 30% sucrose-1× PBS at 4°C until sectioning. Immunostaining was performed on 25 μm coronal (brain) and axial (spinal cord) sections. For all antibody staining, sections underwent antigen retrieval in citrate (pH 6.0; 100°C) for 20 min. For laminin, CD4 and CD45, sections were treated with 0.5 mg/ml protease XIV (Sigma–Aldrich) at 37°C for 5 min. Primary antibodies were used at concentrations ranging from 1:50 to 1:500. Samples were examined using a Leica Microsystems confocal microscope, and stacks were collected with *z* of 1 μm.

### Morphometric analysis

Morphometric analyses were performed using the NIH ImageJ and Leica LAS software and all analyses were performed blinded to treatment group and genotype. For *in vitro* studies, JAM-A and occludin signal in astrocytes was analysed as pixel intensity in the ImageJ using the *freehand selection* tool to demarcate astrocytes’ cell body without the cell nuclei. Colocalization analyses *in vitro* (JAM-A and occludin) were performed using the ImageJ Just Another Colocalization Plugin (JACoP) through each plane of the *z*-stack. In total, three images were analysed per each of the three technical replicates per condition, from three biological replicates. For colocalization analyses *in vivo* (JAM-A and GFAP, JAM-A and AQP4, JAM-A and CD31, JAM-A and CD45, JAM-A and Iba1) immunohistochemical stains were analysed with the JACoP through each plane of the *z*-stack in at least two (range: 2–3) lumbar spinal cord cross-sections per mouse. Cortical AdIL-1 injection lesions were analysed on coronal brain sections of 25-μm thickness distributed serially across 10 slides from the posterior to anterior end of the brain; adjacent sections on the same slide were roughly 250 μm apart. Sections for analysis were selected to represent the centre of the lesion, corresponding to the area of greatest lesion length as noted by width of neuronal nuclear protein (NeuN) loss and GFAP positivity. Field of analysis for lymphocyte localization relative to the PVS was selected on a 20× field of ∼770 μm width centred over the midline of the cortical injection site. Anatomical localization of CD4^+^ lymphocytes relative to the laminin-demarcated PVS in cortical AdIL-1 lesions was analysed manually through each plane of the *z*-stack. At least two (ranging from two to four) representative sections through the centre of the lesion were analysed per mouse and values were then averaged for each animal. EAE spinal cords were analysed on axial spinal cord sections of 25-μm thickness distributed serially across 10 slides from the caudal to rostral end of the lumbar, thoracic and cervical spinal cord; adjacent sections on the same slide were roughly 250 μm apart. Immunopathological analyses focused on the lumbar white matter (anterolateral tract) and grey matter (ventral horn). At least three (3–6) representative images were quantified and averaged from age- and sex-matched animals per condition per genotype per time point. Myelin loss, neuronal loss and astrocyte reactivity were quantified by measuring the FM-positive area, counting NeuN+ cells and measuring GFAP+ pixel sums normalized to total area in the field of interest in matched projections at ×20 magnification. Quantification in the spinal cord EAE of CD4^+^ lymphocyte cuffs within the BBB, as demarcated by laminin staining, was performed on at least two (2–7) representative lumbar sections per mouse at ×20 magnification, values were normalized to area and averaged for each animal. The proportion of parenchymal infiltration of CD4^+^ cells was calculated from these sections as the number of ×20 sections demonstrating parenchymal infiltration of CD4^+^ cells over the total number of sections averaged for each animal.

### Statistical analysis

Statistical analyses were performed in GraphPad Prism version 9.2. All data were first tested for Gaussian distribution using the Shapiro–Wilk normality test. When the data satisfied the normal distribution assumption, parametric tests were applied: Student’s *t*-test was used to compare two groups of unmatched samples, and the one-way ANOVA with Tukey’s *post hoc* test was used to compare more than two groups to each other. When the data deviated significantly from normality, non-parametric tests were applied: Mann–Whitney *U* test was used to compare two groups of unmatched samples, Kruskal–Wallis one-way ANOVA followed by Dunn’s multiple comparison test was used when comparing more than two groups. Differences in EAE disease course were assessed using non-parametric tests as follows: Friedman’s repeated-measures one-way ANOVA followed by Dunn’s multiple comparison test was used to compare more than two groups, whereas the Wilcoxon matched-pairs signed-rank test was applied when comparing two groups. Survival curves for mortality and disease induction in EAE were analysed with the Mantel–Cox test. The statistical analyses are reported in the figure legends and all results are shown as mean ± SEM. In all cases, *P* < 0.05 was considered significant.

### Study approval

The use of commercially available human astrocytes and anonymized human blood donor samples was approved by the IRB at the Icahn School of Medicine at Mount Sinai (ISMMS). Studies using mice were approved by the IACUC at the ISMMS and adhered to the American Veterinary Medical Association guidelines. The ISMMS has an Animal Welfare Assurance on file with the Office for Laboratory Animal Welfare (assurance no. A3111-01).

### Data availability statement

The data that support the findings of this study are available from the corresponding author, upon reasonable request.

## Results

### Astrocytic JAM-A is upregulated diffusely on the astrocytic cell surface in response to IL-1β *in vitro* and in *in vivo* models of CNS inflammatory disease

We reported previously that reactive astrocytes upregulate the tight junction proteins, claudin-1 (CLDN-1), claudin-4 (CLDN-4) and JAM-A in response to CNS inflammation.^15^ In CNS vascular and gut endothelial cells, JAM-A acts both as a tight junction molecule in *trans* dimeric form and as an immune cell receptor in monomeric form.^29–31^ In vascular endothelium, the cytokine C–C motif chemokine ligand 2 (CCL-2) serves as a switch, causing JAM-A internalization from the tight junction and relocalization to the cell surface as a monomer.^20,30^

**Figure 1:**
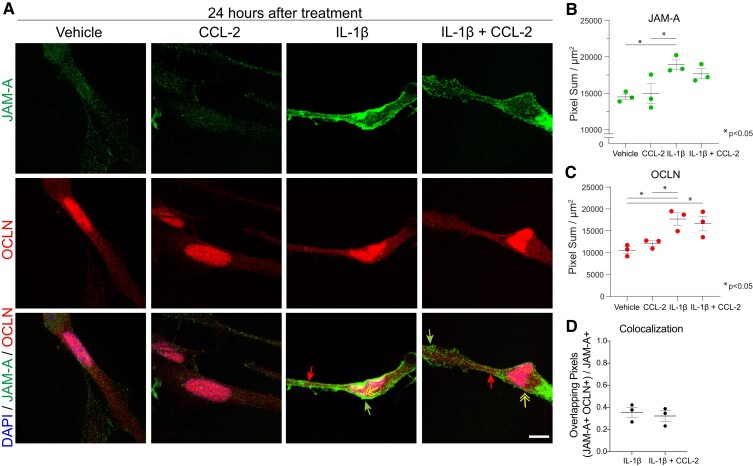
**Inflammation induces reactive astrocytes to express JAM-A diffusely throughout the cell surface membrane *in vitro***. (**A** and **B**) Astrocytic JAM-A (green) was increased *in vitro* at 24 h after treatment with 20 ng/ml IL-1β and, not significantly, by the combination of IL-1β + 100 ng/ml CCL-2 but not CCL-2 alone (average vehicle (14 500) versus IL-1β (18 915) versus CCL-2 (14 950) versus IL-1β + CCL-2 (17 665), vehicle versus IL-1β: *P* = 0.03, CCL-2 versus IL-1β: *P* = 0.04, one-way ANOVA with Tukey’s multiple comparison test). JAM-A was both diffusely localized throughout the cell membrane (green arrows) and co-localized with the tight junction marker, occludin (OCLN, red, red arrows; yellow double-headed arrow pointing to overlay of OCLN and JAM-A in yellow). Scale bar: 10 µm. (**C**) Astrocytic occludin was similarly induced at 24 h after treatment with IL-1β or the combination of IL-1β and CCL-2 but not CCL-2 alone (average vehicle (10 550) versus IL-1β (17 706) versus CCL-2 (12 117) versus IL-1β + CCL-2 (16 657), vehicle versus IL-1β: *P* = 0.01, vehicle versus IL-1β + CCL-2: *P* = 0.03, CCL-2 versus IL-1β: *P* = 0.04, one-way ANOVA with Tukey’s multiple comparison test). (**D**) The addition of CCL-2 to IL-1β did not change the proportion of JAM-A+ pixels co-localized with OCLN+ pixels at 24 h [IL-1β (0.36) versus IL-1β + CCL-2 (0.32), *P* = 0.63, unpaired two-tailed *t*-test]. (**B–D**) The analysis was performed on three images for each three technical replicates per condition. Dot plots show the average of the technical replicates within each biological replicate (*n* = 3 per group).

Using human astrocyte cultures, we confirmed that treatment with IL-1β, but not CCL-2 alone, upregulated the expression of JAM-A and the tight junction protein occludin by 24 h (Fig. 1A–C). Intriguingly and confirming previous work,^32^ occludin also localized to the cell nuclei of cultured astrocytes (Fig. 1A). Upon induction, astrocytic JAM-A not only co-localized with occludin but was also distributed more diffusely throughout the cell membrane (Fig. 1A and D). Combined treatment with IL-1β and CCL-2 did not augment or change the distribution of JAM-A compared with IL-1β alone (Fig. 1A, B and D). Therefore, unlike endothelial JAM-A, astrocytic JAM-A is expressed diffusely throughout the cell membrane after induction by IL-1β and its distribution does not change with the addition of CCL-2.

**Figure 2:**
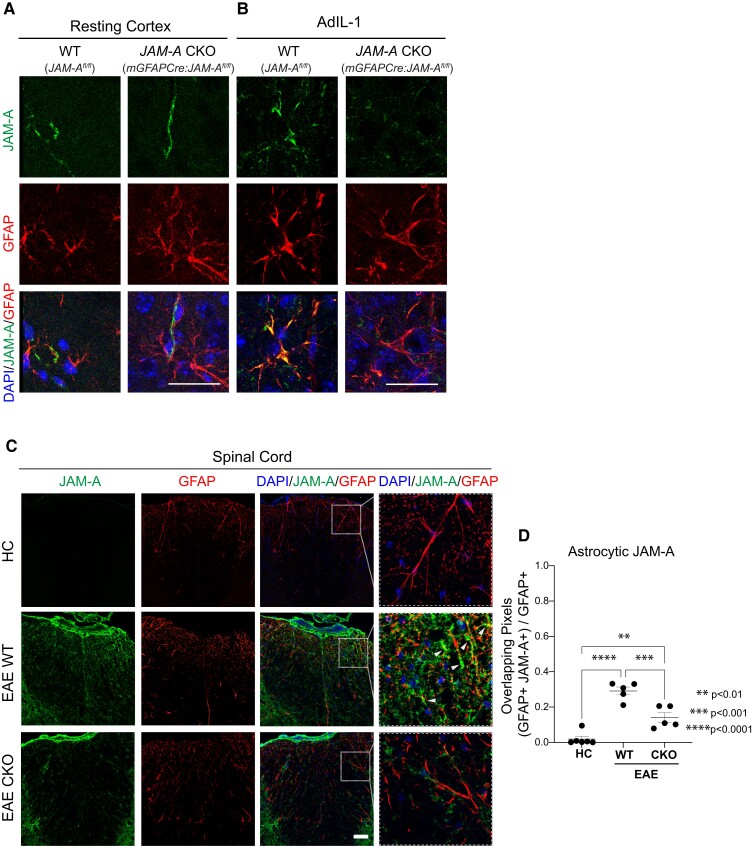
**Astrocytic JAM-A expression was successfully reduced *in vivo* using a transgenic mouse line.** (**A** and **B**) JAM-A and GFAP expression patterns were visualized *in vivo* in the cortex of WT (*JAMA^fl/fl^*) and *JAM-A* CKO (*mGFAPCre:JAMA^fl/fl^*) mice both in resting conditions (**A**) and after the injection of an IL-1β expressing adenovirus (AdIL-1) (**B**). In the resting cortex (**A**), JAM-A (green) does not strongly overlap (yellow) with astrocytes (GFAP, red). In the inflamed cortex of WT AdIL-1-injected brains (**B**), JAM-A (green) overlaps with GFAP (red, overlap: yellow) and astrocytic JAM-A appears diminished in CKOs. Scale bars in **A** and **B** are 25 µm. (**C**) Images show immunofluorescence for JAM-A (green), GFAP (red) and DAPI (blue) in the spinal cord dorsal column of HCs, WT and CKO mice with EAE. JAM-A expression is nearly undetectable in the spinal cord of HC mice, while it is upregulated in EAE WT and EAE CKO mice. EAE CKO mice show decreased immunoreactivity to JAM-A in GFAP-positive astrocytes compared with EAE WT, as shown in the higher magnification inset (white dashed square). White arrowheads point to JAMA-A+ GFAP+ astrocytes (overlap: yellow). Scale bar: 50 µm. (**D**) Colocalization analysis shows a greater proportion of GFAP+ pixels that co-localize with JAM-A+ pixels during EAE compared with HC [average HC (0.19) versus EAE WT (0.29) versus EAE CKO (0.14), HC versus EAE WT: *P* < 0.0001; HC versus EAE CKO: *P* = 0.0036] and a decreased proportion in EAE CKO mice compared with EAE WT mice (EAE WT versus EAE CKO: *P* = 0.0009). Number of animals HC, *n* = 6; EAE WT, *n* = 5; EAE cKO, *n* = 5, one-way ANOVA with Tukey’s multiple comparison test.

We characterized the expression of astrocytic JAM-A in two models of CNS inflammation in the mouse. In resting (i.e. healthy) cortex, JAM-A was not expressed in astrocytes of the CNS parenchyma (identified by labelling for GFAP) but it was expressed in other cell types (Fig. 2A), in a pattern consistent with previous work demonstrating JAM-A expression on the vascular endothelium.^20,30,33,34^ In asymptomatic inflammatory lesions produced by intracortical injections of IL-1β expressing adenovirus (AdIL-1), reactive astrocytes expressed JAM-A (Fig. 2B). Conditional KO mice for *JAM-A* in astrocytes (*mGFAP:CreJAM-A^fl/fl,^* CKO) appeared, at a qualitative level, to not express astrocytic JAM-A 7 days after the intracortical injection of AdIL-1 (Fig. 2B). In healthy spinal cord, astrocytic JAM-A was largely undetectable (Fig. 2C). In inflammatory demyelinating spinal cord lesions of EAE, JAM-A was expressed in astrocytes (GFAP) (Fig. 2C), and particularly in the aquaporin-4 (AQP4)-positive endfeet of the perivascular astrocytes (Supplementary Fig. 1A and B), as well as on infiltrating leukocytes (Supplementary Fig. 2) and additional CNS resident cell types within EAE lesions. JAM-A was present at low levels on blood vessels (cluster of differentiation 31, CD31) (Supplementary Fig. 1A and C), robustly expressed by microglial cells [ionized calcium binding adaptor molecule 1 (Iba1+)] within areas of inflammation (Supplementary Fig. 3A and B) and on the surface of ventral horn neurons (neuronal nuclei, NeuN+) (Supplementary Fig. 4A and B). Oligodendrocytes [oligodendrocyte transcription factor 2 (Olig2+)] did not show clear overlap with JAM-A staining (Supplementary Fig. 5). Astrocyte-specific knock-down of *JAM-A* was demonstrated in EAE lesions, but not in healthy spinal cord, of CKO mice compared with littermate WT (*JAM-A^flfl^*, WT) controls (Fig. 2C and D; Supplementary Fig. 1A and B). CKO mice showed decreases of JAM-A expression within astrocytes (GFAP), including the astrocytic endfeet (AQP4), but not in the endothelium (CD31) during EAE (Fig. 2C and D; Supplementary Fig. 1A and B).

### Astrocytic JAM-A increases levels of pro-inflammatory cytokines and proteases critical for CNS autoinflammatory disease

**Figure 3:**
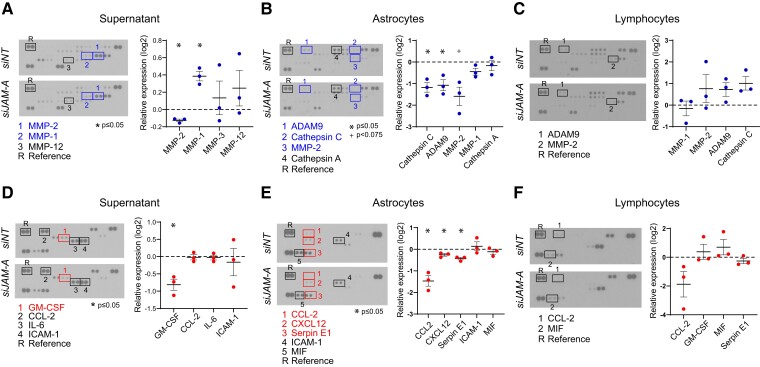
**Astrocytic JAM-A increases pro-inflammatory protease and cytokine levels in astrocyte-CD3^+^ T-cell co-culture.** Astrocytes were transfected with JAM-A or non-targeted siRNA (*siJAM-A* versus *siNT*), then co-cultured with CD3^+^ T cells for 24 h and samples processed for human protease and cytokine ELISA immunoassays. (**A–C**) JAM-A knock-down in astrocytes led to an increase of MMP-1 (relative log_2_ expression 0.38, *P* = 0.019) and a decrease of MMP-2 (relative log_2_ expression −0.13, *P* = 0.014) in the supernatant and decrease of ADAM9 (relative log_2_ expression −1.074, *P* = 0.05) and cathepsin C (relative log_2_ expression −1.174, *P* = 0.006) in astrocyte lysates (**B**). There were no significant changes in protease levels seen in lymphocyte lysates (**C**). (**D–F**) Astrocytic JAM-A knock-down led to decreased levels of (**D**) GM-CSF (relative log_2_ expression −0.79, *P* = 0.04) in the supernatant and (**E**) CCL-2 (relative log_2_ expression −1.3, *P* = 0.03), CXCL12 (relative log_2_ expression −0.13, *P* = 0.04) and serpin E1 (relative log_2_ expression −0.3, *P* = 0.03) in astrocytic lysates. There were no significant changes in cytokine levels seen in lymphocyte lysates (**F**). Data (**A–F**) are from three biological replicates; two-tailed paired *t*-tests were performed on probes demonstrating a visually detectable signal in normalized expression values relative to a reference control.

Local protease and cytokine levels within the PVS play a critical role in facilitating immune cell priming, CNS entry and autoimmune attack.^8,13^ We tested whether astrocytic JAM-A leads to changes in protease and cytokine levels in an astrocyte-T-cell co-culture system. Here, we used a pan-T-cell population (cluster of differentiation 3, CD3) to assess the net effects of astrocytic JAM-A signalling to both cluster of differentiation 4 (CD4^+^) and 8 (CD8^+^) T cells. ELISA arrays were performed on co-cultures of activated (IL-1β-treated) human astrocytes and CD3^+^ T cells in the presence or absence of astrocytic JAM-A. The efficacy of siRNA-mediated knock-down of JAM-A (*siJAM-A*) in comparison to a non-targeting siRNA (*siNT*) was demonstrated previously.^15^ Supernatants extracted from co-cultures with *siJAM-A* transfected astrocytes showed statistically significant decreases in MMP-2 (Fig. 3A) and granulocyte-macrophage colony-stimulating factor (GM-CSF) (Fig. 2D), both factors previously shown to promote EAE pathogenesis.^8,9,35–44^ Lysates of *siJAM-A* transfected astrocytes showed decreased levels of a disintegrin and a metalloproteinase 9 (ADAM9), cathepsin C and CCL-2 (Fig. 3B and E), the latter of which is known to promote EAE pathogenesis via its chemotactic effects on infiltrating monocytes.^45,46^ Lysates of CD3^+^ T cells showed no statistically significant protease or cytokine changes (Fig. 3C and F). In sum, in astrocyte-T-cell co-cultures subjected to pro-inflammatory conditioning of both cell types, astrocytic JAM-A deletion leads to decreased levels of MMP-2, CCL-2 and GM-CSF, EAE promoting signals involved in immune cell infiltration into the CNS parenchyma and pathogenic T-cell activity.

### Astrocytic JAM-A regulates immune cell infiltration past the PVS in IL-1β induced cortical lesions

**Figure 4:**
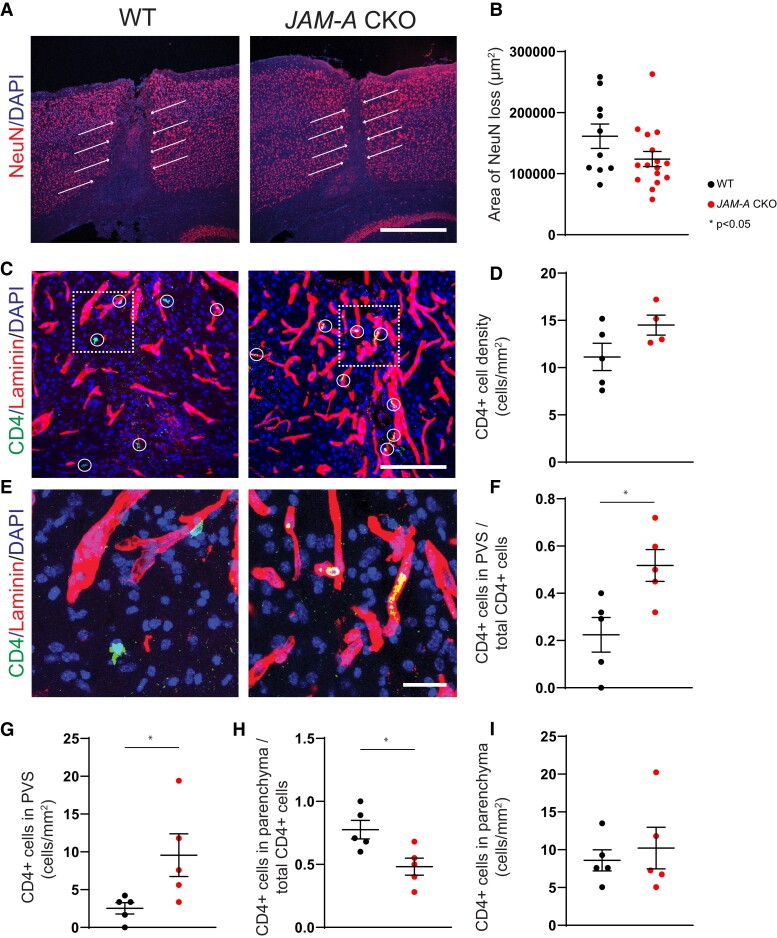
**In inflammatory cortical lesions, CD4^+^ T cells are arrested within the PVS in the absence of astrocytic JAM-A**. Asymptomatic inflammatory cortical lesions were induced in JAM-A CKO and WT mice via the microinjection of AdIL-1 into the frontal cortex. Brains were harvested for histopathology at 7 days post-injection. (**A** and **B**) Lesions in JAM-A CKO mice, as measured by the area of neuronal cell death (loss of NeuN, red, white arrows), showed a trend in smaller lesion size compared with WT mice that did not reach statistical significance (*P* = 0.18, CKO *n* = 16 mice, WT *n* = 10 mice, Mann–Whitney test). (**C** and **D**) CD4^+^ cells (green and circled in white) showed an increased but statistically non-significant trend in number within JAM-A CKO lesions, scale bar: 125 μm [average number/mm^2^, 14.5 (CKO) versus 11.1 (WT), *P* = 0.11; CKO *n* = 4 mice, WT *n* = 5 mice, Mann–Whitney test]. (**E–I**) In JAM-A CKO mice, a higher proportion and total number of CD4^+^ cells (green) co-localize to the laminin-positive (red) basement membrane of the PVS while a lower proportion of cells localize to the parenchyma (laminin-negative) though total number of cells within the parenchyma per area remain the same. (**E**) Scale bar: 25 μm. (**F**) Average proportion of CD4^+^ cells in the PVS over total CD4^+^ cells: 0.50 (CKO) versus 0.29 (WT), *P* = 0.02. (**G**) Average number CD4^+^ cells in the PVS/mm^2^: 7.6 (CKO) versus 3.4 (WT), *P* = 0.03. (**H**) Average proportion of CD4^+^ cells in the parenchyma over total CD4^+^ cells: 0.50 (CKO) versus 0.70 (WT), *P* = 0.02. (**I**) Average number CD4^+^ cells in the PVS/mm^2^: 7.2 (CKO) versus 7.6 (WT), *P* = 0.85. (**F–I**) *N* = 5 mice per group, Mann–Whitney tests.

To test whether astrocytic JAM-A facilitates immune cell infiltration past the PVS and contributes to parenchymal damage, we characterized lesion size and patterns of immune cell entry in asymptomatic cortical lesions induced by intracortical AdIL-1 injection. Lesion size after AdIL-1 injection, measured as area of neuronal loss, showed a decreasing trend not reaching statistical significance in *mGFAP:CreJAM-A^fl/fl^* (CKO) mice compared with *JAM-A^flfl^* (WT) mice (Fig. 4A and B). CKO mice also demonstrated an increasing trend not reaching statistical significance in the number of CD4^+^ immune cells within lesions compared with the WT group (Fig. 4C and D). However, CKO mice showed an increased proportion and total number of CD4^+^ immune cells within the PVS, as demarcated by pan-laminin staining (Fig. 4C and E–G). In contrast, in WT mice, a higher proportion of CD4^+^ cells were located in the parenchyma, indicating successful migration out of the PVS (Fig. 4C, E and H). Although CKO mice showed a decreased proportion of parenchymal CD4^+^ cells compared with WT controls (Fig. 4H), the overall number of CD4^+^ cells in the parenchyma was not statistically different between groups (Fig. 4I) probably due to the higher total number of CD4^+^ cells in CKO mice (Fig. 4D). This finding suggests that astrocytic *JAM-A* knock-down led to the retention and accumulation of CD4^+^ cells within the PVS, yet did not significantly affect the total number of cells ultimately able to access the parenchyma and by extension, the size of the cortical lesion. In sum, astrocytic JAM-A appears to modulate CD4^+^ immune cell infiltration past the GL and into the CNS parenchyma in cortical AdIL-1-induced lesions.

### JAM-A deletion in astrocytes decreases MMP-2 levels and T-cell infiltration in the CNS, and results in reduced tissue damage and disease severity in mice with EAE

**Figure 5:**
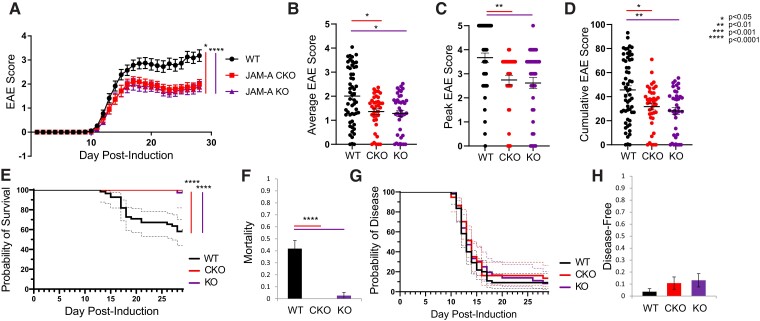
**Astrocytic JAM-A promotes clinical disease severity during EAE.** (**A**) JAM-A CKO and KO mice showed a milder course of clinical disability than WT mice with EAE and there was no difference between CKO and KO animals (WT versus JAM-A CKO: *P* = 0.0122, WT versus JAM-A KO: *P* < 0.0001, JAM-A CKO versus JAM-A KO: *P* > 0.05, Friedman’s one-way ANOVA with Dunn’s multiple comparisons test). (**B–D**) Average (**B**), peak (**C**) and cumulative (**D**) scores of the EAE trial shown in (**A**) were significantly lower in CKO and KO mice compared with WT [average score: 2.0 (WT) versus 1.36 (CKO) versus 1.28 (KO), WT versus CKO: *P* = 0.030, WT versus KO: *P* = 0.016, CKO versus KO: *P* > 0.5; peak score: 3.6 (WT) versus 2.7 (CKO) versus 2.6 (KO), WT versus CKO: *P* = 0.003, WT versus KO: *P* = 0.002, CKO versus KO: *P* > 0.5; cumulative score: 45.58 (WT) versus 31.75 (CKO) versus 28.2 (KO), WT versus CKO: *P* = 0.04, WT versus KO: *P* = 0.007, CKO versus KO: *P* > 0.5, Kruskal–Wallis tests]. Average in graphs shown with SEM. (**E**) Survival curves of mortality revealed that WT mice sustained greater mortality over the course of EAE than CKO or KO, Mantel–Cox test, *P* < 0.0001. (**F**) At Day 28, mortality rate was higher in WT mice compared with CKO and KO [0.41 (WT) versus 0 (CKO) versus 0.26 (KO), *P* < 0.0001, Kruskal–Wallis test]. (**G**) Disease curves demonstrated no differences in susceptibility to or timing of disease, Mantel–Cox test, *P* = 0.65. (**H**) At Day 28, rates of disease resistance (proportion of mice that did not develop neurological deficit) showed an increased trend that was not statistically significant for CKO and KO compared with WT [0.014 (WT) versus 0.108 (CKO) versus 0.131 (KO), *P* = 0.22, Kruskal–Wallis test]. Graphs (**A–H**) show pooled data from three independent EAE experiments for WT and CKO and two independent EAE experiments for KO with a minimum of nine mice per group in each experiment, total WT *n* = 55, JAM-A CKO *n* = 37, JAM-A KO *n* = 38.

**Figure 6:**
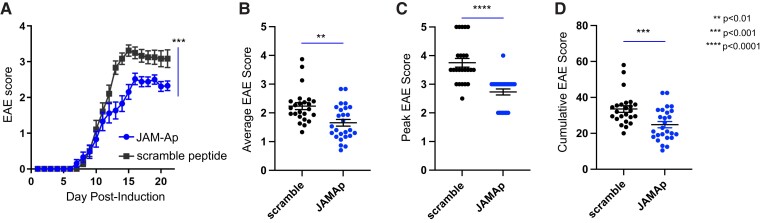
**Treatment with a JAM-Ap reduced clinical disease severity during EAE.** (**A**) WT mice with EAE treated with daily intraperitoneal injection of a JAM-Ap from Day 7 post-immunization showed a significantly milder course of clinical disability compared with scramble peptide-treated controls. Differences in the disease progression over time were assessed with the Wilcoxon matched-pairs signed-rank test (*P* = 0.0005). Average (**B**), peak (**C**) and cumulative (**D**) scores of the EAE trial shown in (**A**) were significantly lower in JAM-Ap-treated mice compared with scramble peptide-treated mice [average score: 2.23 (scramble) versus 1.65 (JAM-Ap), *P* = 0.001; peak score: 3.75 (scramble) versus 2.73 (JAM-Ap), *P* < 0.0001; cumulative score: 33.54 (WT) versus 24.83 (CKO), *P* = 0.001, Mann–Whitney tests]. Average in graphs shown with SEM. Graphs (**A–D**) show pooled data from three independent EAE experiments with a minimum of eight mice per group for each experiment, total JAM-Ap *n* = 24, scramble *n* = 26.

To test the role of astrocytic JAM-A in a model of CNS autoimmune demyelinating disease, EAE was induced in *JAM-A^flfl^* (WT), *mGFAP:CreJAM-A^fl/fl^* (CKO) and *JAM-A^−/−^* (KO) mice. CKO and KO mice were studied to dissociate astrocytic JAM-A loss from total JAM-A deletion. CKO and KO mice both showed statistically significant milder courses of disease (Fig. 5A), including lower average (Fig. 5B), peak (Fig. 5C) and cumulative (Fig. 5D) disease scores at Day 28 post-induction compared with WT mice. Survival curves showed statistically significant differences in mortality, but not disease induction between WT and the CKO and KO mice (Fig. 5E and G). Rates of mortality at Day 28 post-induction were significantly reduced in both CKO and KO mice (Fig. 5F) while rates of resistance to disease induction showed a greater, non-statistically significant trend in CKO and KO mice compared with WTs (Fig. 5H). To confirm the translational potential of JAM-A blockade, WT mice were treated with daily intraperitoneal injection starting at Day 7 post-immunization of either a soluble JAM-Ap specifically targeting the monomeric form or a scramble non-targeting peptide. Treatment with JAM-Ap demonstrated a protective effect against clinical disability in EAE compared with the scramble control (Fig. 6A–D).

**Figure 7:**
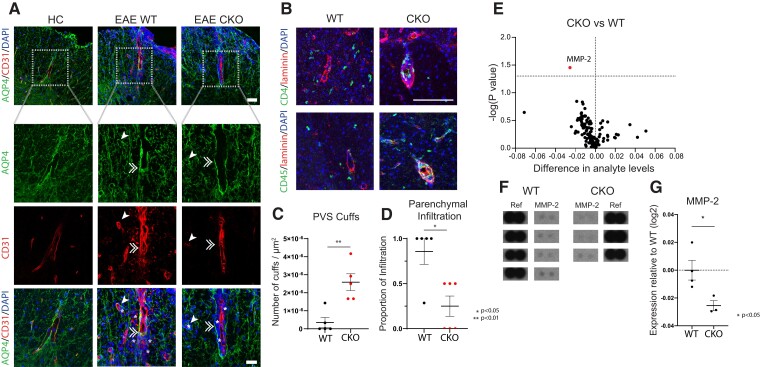
**In EAE, astrocytic JAM-A signalling induces MMP-2 expression and promotes T-lymphocyte entry into the CNS parenchyma from the PVSs.** (**A**) PVSs were identified relative to AQP4 staining of the astrocyte endfeet and CD31 staining of the endothelium in HC, WT and CKO mice. Cell infiltrates (DAPI, blue) were seen in the CNS at 5 days post-EAE disease onset in both WT and CKO but not in HC. Representative images demonstrate that in JAM-A CKO, infiltrating cells (highlighted by white asterisks) accumulated mostly within the PVSs (between AQP4 and CD31) whereas in WT, infiltrating cells localized diffusely past the PVSs within the CNS parenchyma. White single arrowheads and white doubled arrowheads indicate transverse and longitudinal blood vessels, respectively. *Top* panel, scale bar: 50 µm. *Bottom* panels represent inset outlined by dotted white box, scale bar: 30 µm. (**B**) In inflammatory lesions of EAE at Day 21 post-immunization, in CKO mice, CD4^+^ (green, upper panel) and CD45^+^ cells (green, lower panel) clustered in ‘cuffs’, colocalizing with the pan-laminin marker (in red), whereas in WT mice, CD4^+^ and CD45^+^ cells were instead located in the parenchyma. Scale bar: 100 µm. (**C**) Number of CD4^+^ cuffs/µm^2^ of lumbar spinal cord cross-sections were increased in JAM-A CKO mice compared with WTs [average 3.5 × 10^−7^ (WT) versus 2.6 × 10^−6^ (CKO), *P* = 0.008, Mann–Whitney test, number of mice: WT *n* = 5, CKO *n* = 5]. (**D**) Proportion of parenchymal infiltration of CD4^+^ cells seen in serial spinal cord sections was lower in JAM-A CKO compared with WT mice [average 0.86 (WT) versus 0.25 (CKO), *P* = 0.02, Mann–Whitney test, WT *n* = 5, CKO *n* = 6]. (**E**) A volcano plot shows the differential level of 111 pro-inflammatory cytokines, chemokines, proteases and acute phase reactants in spinal cord lysates of JAM-A CKO and WT mice at 5 days post-EAE disease onset as measured using mouse proteome ELISA immunoassays. CKO mice showed an overall reduction of many factors, though MMP-2 (highlighted in red) was the sole statistically significant factor compared with WT controls. (**F**) MMP-2 probes on the ELISA array in CKO and WT mice, along with reference spots, used for signal (pixel intensity) normalization, are shown. Complete ELISA arrays from each mouse are shown in Supplementary Fig. 6 and original blots are included in Supplementary Materials. (**G**) Spinal cord levels of MMP-2 in CKO mice relative to WT controls were significantly decreased at 5 days post-EAE disease onset (relative log_2_ expression −0.025, *P* = 0.0351, two-tailed unpaired *t*-test, WT, *n* = 4; CKO, *n* = 3).

The course and severity of disease did not differ between CKO and KO mice, suggesting that the protective effect of JAM-A blockade may be fully attributed to astrocytic JAM-A. To eliminate confounding mechanisms of JAM-A deletion in other tissues and cell types, we decided to focus on neuropathology in the CKO line. Immunohistopathology was performed in CKO and WT mice to measure first patterns of immune cell infiltration into spinal cord lesions. At 5 days from EAE onset, when immune cells are most exponentially infiltrating the CNS,^47^ infiltrating cells were found to be diffusely distributed throughout the CNS parenchyma in WT mice but accumulated within the PVS between the astrocytic endfeet (AQP4) and blood vessel wall (CD31) in CKO mice (Fig. 7A). Subsequently at Day 21 post-EAE immunization, WT mice continued to show CD4^+^ and CD45^+^ immune cells throughout the CNS parenchyma while CKOs showed ongoing accumulation, or cuffing, of CD4^+^ cells within the laminin-rich PVS and decreased infiltration of CD4^+^ cells into the parenchyma (Fig. 7B–D).

To establish a mechanistic link between astrocytic JAM-A signalling and T-cell infiltration *in vivo*, a proteome profiler probing 111 soluble mouse proteins, including cytokines, chemokines, proteases, growth factors and acute phase signals was used to compare the proteomic patterns of spinal cord tissues from WT and CKO mice at 5 days from EAE disease onset. Of 111 probes, MMP-2, which had shown strong astrocytic JAM-A-dependent regulation *in vitro*, demonstrated the highest fold change *in vivo* and was the sole factor with a statistically significant difference in expression between WT and CKO mice (Fig. 7E–G and Supplemental Fig. 6).

**Figure 8:**
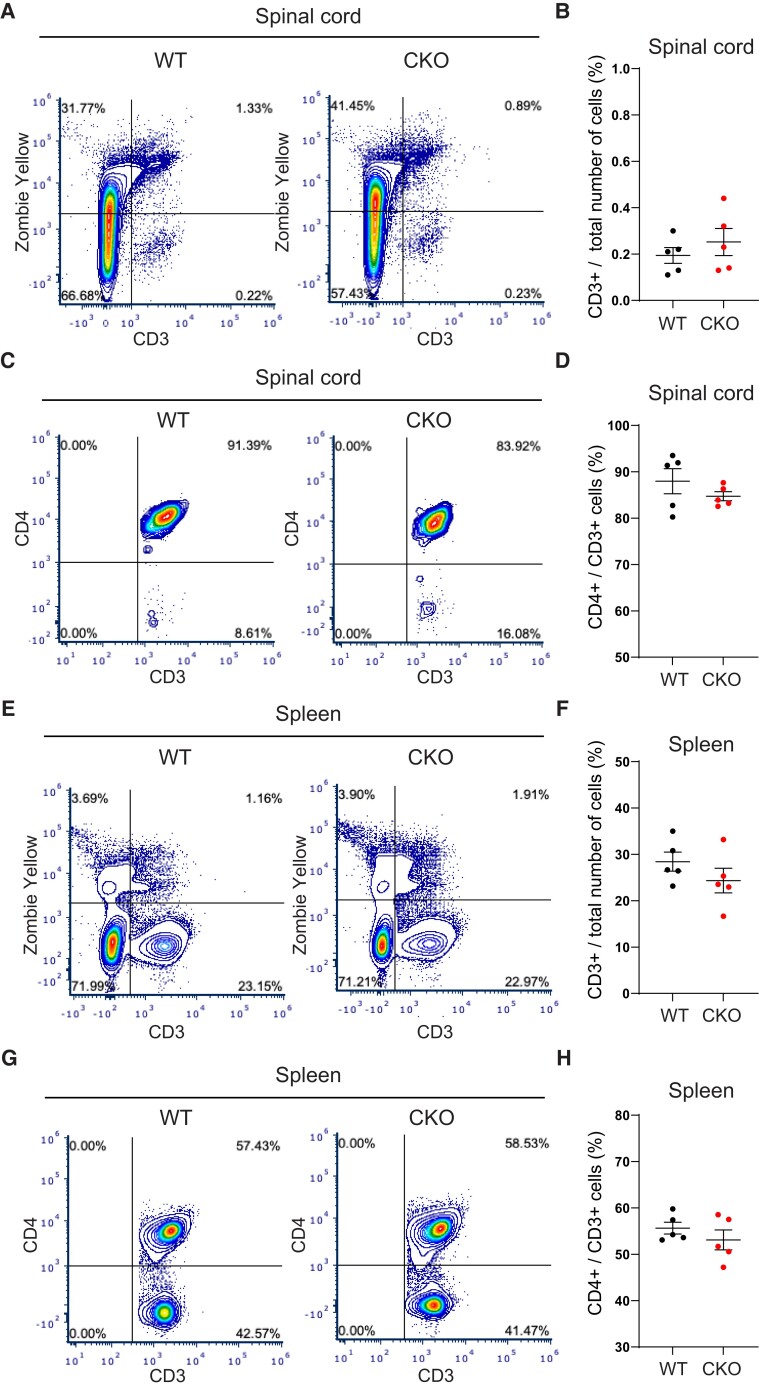
**Astrocytic JAM-A does not change the total number of CD3^+^ or proportion of CD4^+^ cells within the CNS or spleen during EAE.** (**A–H**) Flow cytometry was performed on spinal cords and spleens of WT (*n* = 5) and CKO (*n* = 5) mice with EAE on Day 5 from the onset of disease. Average EAE score: WT 2.8, CKO 2.6. (A) Representative plots show (**B**) similar total CD3^+^ cell counts in the spinal cord of CKO and WT (mean CKO 0.23% versus WT 0.19%, *P* = 0.42, unpaired two-tailed *t*-test). (**C**) Representative plots reflect (**D**) no difference found in the proportion of CD3^+^CD4^+^ cells in the spinal cord of CKOs compared with WTs (mean CKO 84.73% versus WT 87.97%, *P* = 0.29, unpaired two-tailed *t*-test). (**E–H**) In the spleen, total CD3^+^ and CD3^+^CD4^+^ counts were unchanged between CKO and WT groups (CD3^+^: mean CKO 24.3% versus WT 28.4%, *P* = 0.25; CD3^+^CD4^+^: mean CKO 53.1% versus 55.6%, *P* = 0.34, unpaired two-tailed *t*-test).

To test whether the absence of astrocytic JAM-A and PVS cuffing altered the total number of CD3^+^ and CD4^+^ T cells entering the CNS during EAE, flow cytometry was performed in CKOs and WTs at 5 days from EAE disease onset during the ascending phase of disease. No difference in total CD3^+^ T-cell number was found in the spinal cord (Fig. 8A and B) or spleen of CKO mice compared with WTs (Fig. 8E and F). In the spinal cord, CD3^+^CD4^+^ T-cell counts showed a decreasing trend not reaching statistical significance in CKOs compared with WTs (Fig. 8C and D). In the spleen, CD3^+^CD4^+^ counts were not significantly different between groups (Fig. 8G and H). Therefore, astrocytic JAM-A deletion affected the spatial distribution but not the total number of T cells within the CNS during EAE.

**Figure 9:**
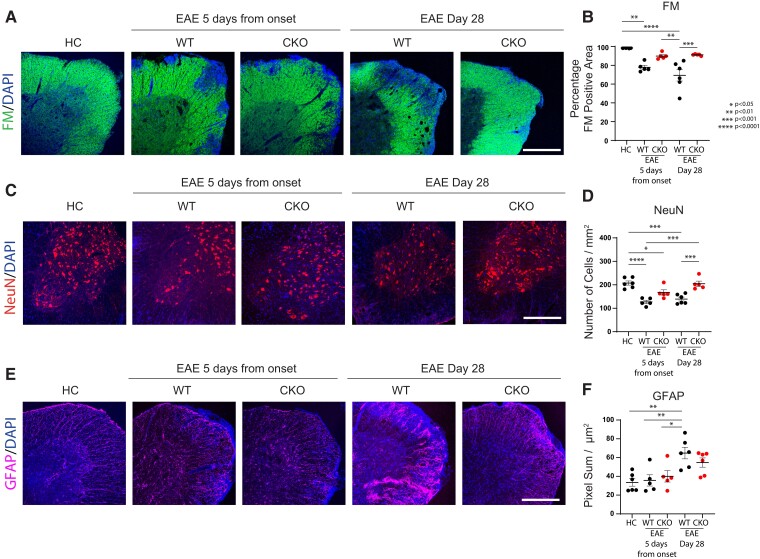
**Astrocytic JAM-A exacerbates histopathological markers of neuroinflammatory damage in EAE.** (**A** and **B**) Proportion of fluoromyelin (FM, green; marker of myelin) positive area of the lumbar anterolateral white matter tracts was significantly decreased in WT—but not in CKO—mice in both the acute (5 days from disease onset) and late chronic stage of EAE (28 days post-induction) compared with HCs [HC (98.53%) versus EAE WT 5 days from disease onset (77.85%), *P* = 0.0014; HC (98.53%) versus EAE WT Day 28 post-induction (69.35%), *P* < 0.0001]. In the acute phase of EAE, CKO mice showed a greater, although non-statistically significant proportion of myelinated white matter area than time-matched WT controls [EAE CKO 5 days (89.91%) versus EAE WT 5 days (77.85%), *P* > 0.05] and a significantly greater proportion of FM-positive area than EAE WT mice at 28 days post-induction [EAE CKO 5 days (89.91%) versus EAE WT Day 28 post-induction (69.35%), *P* = 0.0015]. At the chronic stage of disease, EAE CKO mice showed a significantly larger proportion of myelinated white matter area than time-matched WT controls [EAE WT Day 28 post-induction (69.35%) versus EAE CKO Day 28 post-induction (91.2%), *P* = 0.0007]. Scale bar: 400 µm. (**C** and **D**) Number of NeuN+ neurons/mm^2^ within the ventral grey matter of the lumbar spinal cord was, on average, higher in EAE CKO mice than in EAE WT controls and significantly in EAE CKO mice at Day 28 post-induction compared with time-matched controls (average values for HC (207.5), EAE WT 5 days from disease onset (127.1) EAE CKO 5 days from disease onset (167.1), EAE WT Day 28 post induction (139.3), EAE CKO Day 28 post induction (204.6); HC versus EAE WT 5 days: *P* < 0.0001, HC versus EAE CKO Day 5: *P* = 0.04, HC versus WT Day 28: *P* = 0.0002, EAE WT 5 days versus EAE CKO Day 28: *P* = 0.0001, EAE WT Day 28 versus EAE CKO Day 28: *P* = 0.0005). Scale bar: 100 µm. (**E** and **F**) GFAP-positive (purple, marker of astrocytes) pixel sum/µm^2^ of the lumbar anterolateral white matter tracts was quantified as a proxy for astrogliosis. GFAP levels were similar between HCs and mice with EAE at Day 5 from disease onset and significantly increased in EAE WT mice at Day 28 post-immunization (average values for HC (33.55), EAE WT 5 days from disease onset (35.73), EAE CKO 5 days from disease onset (40.02), EAE WT Day 28 post induction (64.83), EAE CKO Day 28 post induction (54.72); HC versus EAE WT Day 28: *P* = 0.002, EAE WT 5 days versus EAE WT Day 28: *P* = 0.009, EAE WT Day 5 versus EAE WT Day 28: *P* = 0.03). EAE CKO mice at Day 28 post-induction showed a decreased trend of GFAP immunoreactivity compared with time-matched WT controls. Scale bar: 100 µm. (**B**, **D** and **E**) HC *n* = 6 animals, EAE WT 5 days from disease onset *n* = 5, EAE CKO 5 days from disease onset *n* = 5, EAE WT Day 28 post-induction *n* = 6, EAE CKO Day 28 post-induction *n* = 5 for **B**, *n* = 6 for **D** and **E**; one-way ANOVA with Tukey’s multiple comparison test. At least three (from three to six) sections from each animal were analysed.

Immunohistochemical analyses of spinal cord tissues from HCs, and mice at 5 days from EAE disease onset and at 28 days post-immunization, demonstrated that CKO mice were protected against the neuropathological damage of EAE compared with WT mice. Throughout EAE, CKO mice maintained the levels of anterolateral tract flouromyelin staining (a marker of myelinated fibres) that were significantly higher than WT and comparable to HCs (Fig. 9A and B). EAE CKO mice also showed a higher number of NeuN^+^ cells within the ventral horn of the lumbar spinal cord than time-matched WT controls (at Day 5 from EAE onset and at Day 28 post-induction) and, in EAE CKO mice at Day 28 post-induction, the number of NeuN^+^ cells was comparable to that observed in HC (Fig. 9C and D). Finally, EAE CKO mice also had similar levels of GFAP signal in the white matter of the lumbar spinal cord compared with HC, while WT mice with EAE at 28 days post-immunization showed significant increases in GFAP signal, suggesting greater astrogliosis (Fig. 9E and F). Collectively, these histopathologic changes reflect a milder course of the disease which resembles other genetic models^8,48^ in which immune cell trapping within the PVSs prevents parenchymal damage and clinical disability.

## Discussion

The GL is the final barrier separating peripheral infiltrating immune cells and soluble factors from the CNS parenchyma.^3,49^ The PVSs therefore represent the penultimate compartment for incoming cells and factors during CNS autoinflammatory disease. Contact-mediated signals between the astrocytic endfeet of the GL and immune cells have the potential to act as critical check-points for both (i) the entry of inflammatory cells into the CNS parenchyma from the PVS and (ii) the functional differentiation of both cell types in the inflammatory context.^13,50–53^ Here, we demonstrate a novel role for the astrocyte cell signalling receptor, JAM-A, in controlling lymphocyte trafficking into the CNS parenchyma with downstream effects on MMP-2 levels, histopathological damage and clinical disability in mice with EAE. Experiments using functional gene network analysis to characterize how the astrocytic JAM-A-mediated interaction between astrocytes and T cells regulates the functional differentiation of both cell types are currently underway. Additional *in vivo* imaging experiments to characterize the dynamics of immune cell trafficking in the presence and absence of astrocytic JAM-A-mediated signalling are also in progress.

JAM-A is an immunoglobulin-like cell surface receptor with well-characterized roles in tight junction formation, endothelial diapedesis and immune cell signal transduction in vascular, gut and lung endothelial cells.^16–18,54^ We demonstrated that astrocytes upregulate JAM-A *in vitro* in response to IL-1β, a critical pro-inflammatory cytokine in multiple sclerosis and EAE pathogenesis, and *in vivo* during EAE and intracortical injection of AdIL-1. Previous work detailing JAM-A expression within active multiple sclerosis lesions, noted patterns within the BBB that appear to conform to upregulated expression within the astrocytic endfeet.^34^

Protease and cytokine ELISA experiments showed that astrocytic JAM-A increases pro-inflammatory effector proteins MMP-2, CCL-2 and GM-CSF in co-culture with a CD3^+^ T-cell population. These factors have previously been demonstrated to promote EAE pathogenesis and multiple sclerosis lesion formation; MMP-2 by facilitating immune cell migration out of the PVS and into the CNS parenchyma,^8,9,41^ CCL-2 through its chemotactic effects on infiltrating monocytes^45,46^ and GM-CSF via its effects on monocyte recruitment and pathogenic T-cell activity in the acute phase^37,55,56^ with pleiotropic effects on tissue damage in the chronic phase.^57^

Comparing conditional *JAM-A* KO mice and controls, we found that astrocytic JAM-A promotes the entry of T cells into the CNS parenchyma in two *in vivo* models of CNS inflammation and that astrocytic deletion of JAM-A reduces clinical disability and histopathological damage during EAE. Astrocyte-specific and total JAM-A deletion showed similar phenotypes suggesting that astrocytic JAM-A may fully account for its pathogenic effects in EAE, though this does not rule out the additional possibility of both pathogenic and protective effects of JAM-A in other tissues, including the intestinal epithelium and spleen. Exogenous administration of a soluble JAM-Ap ameliorated the disease in mice with EAE, demonstrating a net protective effect and translational potential of blocking astrocyte–immune cell interactions during autoimmune attack.

Proteome ELISA arrays on spinal cord tissues at 5 days from EAE disease onset recapitulated *in vitro* findings in the EAE disease model identifying MMP-2 as a critical astrocytic JAM-A-dependent factor *in vivo*. Previous work established a role for MMP-2 in promoting T-cell entry into the CNS parenchyma from the PVSs via several potential mechanisms such as (i) digesting dystroglycans that anchor the astrocytic endfeet to the parenchymal basement membrane,^41^ (ii) activating the pro-inflammatory NFκB pathway in astrocytes via Notch-1^8^ and (iii) degrading perivascular reserves of CXCL12, which promotes the retention of immune cells within the PVS.^58^ Statistically significant differences were not found *in vivo* for GM-CSF and CCL-2, two additional factors found in our *in vitro* experiments to have decreased levels after astrocytic JAM-A deletion. It is possible that local *in vivo* changes in GM-CSF and CCL-2 within the PVS were below the threshold of detection in total spinal cord lysates and may require higher resolution techniques for detection.

Collectively, we show that astrocytic JAM-A is a key component for the effective trafficking of T cells out of the PVS and into the CNS parenchyma. Nonetheless, astrocytic JAM-A is likely not the only player involved in this process since its deletion in astrocytes did not entirely stop T cells from crossing the GL in CNS autoimmune lesions.

The extent to which T-cell activation and differentiation is influenced by local signalling interactions within the PVS has yet to be determined. Experiments measuring the relative proportions of suppressor [regulatory T-helper (Treg) and T-helper Type 2 (Th2)] and pro-inflammatory [T-helper Type 1 (Th1), Th17, GM-CSF secreting] T-helper-cell subsets in JAM-A CKOs and WTs are now underway to determine whether astrocytic JAM-A-mediated signalling has the capacity to modulate T-cell differentiation patterns. Additional potential immunomodulatory players within the PVS include not only the astrocytic endfeet, but also pericytes, microglial processes, migrating oligodendrocyte precursors, basement membrane components and other circulating immune cells including dendritic cells, macrophages and B cells.

Astrocytes have the capacity to both promote and protect against CNS autoinflammatory disease.^13,40,59–68^ In their reactive state, astrocytes drive both acute and chronic phases of neuroinflammation, and contribute to the transition from a neuroinflammatory to a neurotoxic, or neurodegenerative, state.^40,60,64,65,69,70^ Conversion from acute inflammatory injury to a chronic neurodegenerative state is a clinical hallmark of secondary progressive multiple sclerosis and also occurs in a range of other neurologic diseases, including ischaemic stroke and dementia.^71–73^ Future work defining the temporal dynamics of astrocytic JAM-A signalling and other receptor-mediated astrocyte–immune cell interactions within the PVSs will help us to understand how acute neuroinflammatory changes may prime the CNS for longitudinal injury or repair, leading to novel translational strategies for progressive multiple sclerosis and other neurodegenerative diseases.

## Conclusion

Astrocytic JAM-A increases MMP-2 in spinal cord tissues during EAE and promotes the migration of T cells out of the PVSs and into the parenchyma, exacerbating inflammatory histopathology and clinical disability. Exogenous administration of soluble JAM-Ap decreases the severity of EAE demonstrating that blockade of contact-mediated astrocyte–immune cell signalling within the PVS represents a novel therapeutic strategy against multiple sclerosis and other CNS autoimmune diseases.

## Supplementary Material

fcac044_Supplementary_Data

## Abbreviations

AdIL-1 =: IL-1β expressing adenovirus
AGM =: astrocyte growth medium
AQP4 =: aquaporin-4
BBB =: blood–brain barrier
CCL-2 =: C–C motif chemokine ligand 2
CD3, CD4, CD8, CD31, CD45 =: cluster of differentiation 3, 4, 8, 31, 45
CFA =: complete Freund’s adjuvant
CKO =: conditional knock-out
CLDN-1 =: claudin-1
CLDN-4 =: claudin-4
EAE =: experimental autoimmune encephalomyelitis
GFAP =: glial fibrillary acidic protein
GL =: glia limitans
GM-CSF =: granulocyte-macrophage colony-stimulating factor
HC =: healthy control
Iba1 =: ionized calcium binding adaptor molecule 1
IL-1β =: interleukin-1 beta
JAM-A =: junctional adhesion molecule-A
JAM-Ap =: JAM-A blocking peptide
KO =: knock-out
MMP-2 =: matrix metalloproteinase-2
MMP-9 =: matrix metalloproteinase-9
MOG =: myelin oligodendrocyte glycoprotein
NeuN =: neuronal nuclear protein
Olig2 =: oligodendrocyte transcription factor 2
PBMCs =: peripheral blood mononuclear cells
PBS =: phosphate buffer saline
PD-1 =: programmed cell death protein 1
PFA =: paraformaldehyde
PVSs =: perivascular spaces
VCAM-1 =: vascular cell adhesion molecule 1
WT =: wild-type
