# Supplementary material for: Astrocytic junctional adhesion molecule-A regulates T-cell entry past the glia limitans to promote central nervous system autoimmune attack
Source: Brain Commun. 2022 Feb 18;4(2):fcac044. doi: 10.1093/braincomms/fcac044 (PMC8899531; doi:10.1093/braincomms/fcac044)
Supplement: fcac044_Supplementary_Data [file fcac044_supplementary_data.zip › Original Submission(1).pdf]

OXFORD UNIVERSITY PRESS | Brain Communications

Astrocytic JAM-A Regulates T Cell Entry Past the Glia Limitans to Promote CNS Autoimmune Attack

Journal:	*Brain Communications*
Manuscript ID	BRAINCOM-2020-359
Manuscript Type:	Original Article
Date Submitted by the Author:	17-Nov-2020
Complete List of Authors:	Chapouly, Candice; INSERM U1034,<br>Amatruda, Mario; Icahn School of Medicine at Mount Sinai Friedman Brain Institute, Neurology<br>Woo, Viola ; Icahn School of Medicine at Mount Sinai Friedman Brain Institute, Neurology<br>Safavi, Farinaz; National Institutes of Health, National Institute of Neurological Disorders and Stroke<br>Zhang, Joy; University of Virginia School of Medicine<br>Dai, David; University of Pennsylvania Perelman School of Medicine<br>Therattil, Anthony; New York Medical College School of Medicine<br>Moon, Chang; Icahn School of Medicine at Mount Sinai<br>Gordon, Alexandra; University of Miami Miller School of Medicine<br>Parkos, Charles; University of Michigan Michigan Medicine, Pathology<br>Horng, Sam; Icahn School of Medicine at Mount Sinai Friedman Brain Institute, Neurology
Keywords:	astrocyte, multiple sclerosis, experimental autoimmune encephalomyelitis, perivascular space, T cells

SCHOLARONE™
Manuscripts

Astrocytic JAM-A Regulates T Cell Entry Past the Glia Limitans to Promote CNS Autoimmune Attack

Candice Chapouly PhD,[1] Mario Amatruda PhD,[2] Viola Woo BS,[2] Farinaz Safavi MD PhD,[3] Joy Zhang BS,[4] David Dai BA,[5] Anthony Therattil BS,[6] Chang Moon BA,[2] Alexandra Gordon BA,[7] Charles Parkos MD,[8] and Sam Horng MD PhD[2,9]

[1] Univ. Bordeaux, Inserm, Biology of Cardiovascular Diseases, U1034, CHU de Bordeaux, F-33604 Pessac, France

[2] Icahn School of Medicine at Mount Sinai, Dept of Neurology, NY, NY, USA

[3] National Institute of Neurological Disorders and Stroke, National Institutes of Health, Bethesda, MD, USA

[4] University of Virginia School of Medicine, Charlottesville, VA, USA

[5] Perelman School of Medicine at the University of Pennsylvania, Dept of Neurology, Philadelphia, PA, USA

[6] New York Medical College, Valhalla, NY, USA

[7] Miller School of Medicine at University of Miami, Miami, FL, USA

[8] University of Michigan, Dept of Pathology, Ann Arbor, MI, USA

[9] Icahn School of Medicine at Mount Sinai, Dept of Neuroscience, NY, NY, USA

Corresponding Author:

Sam Horng MD PhD

Icahn School of Medicine at Mount Sinai

Icahn 10-20A

1468 Madison Avenue

New York, NY 10029

Email: sam.horng@mssm.edu

(212) 659-1692

For Review Only

Abstract: Contact mediated interactions between the astrocytic endfeet and infiltrating immune cells within the perivascular space are underexplored, yet represent potential regulatory check-points against CNS autoimmune disease and disability. Reactive astrocytes upregulate Junctional Adhesion Molecule-A (JAM-A), an immunoglobulin-like cell surface receptor that binds to T cells via its ligand, the integrin, lymphocyte function-associated antigen-1 (LFA-1). Here, we tested the role of astrocytic JAM-A in regulating CNS autoinflammatory disease. We found that JAM-A mediated signaling between astrocytes and T cells increases levels of MMP-2, CCL-2 and GM-CSF, proinflammatory factors driving lymphocyte entry and pathogenicity in multiple sclerosis (MS) and experimental autoimmune encephalomyelitis (EAE), an animal model of CNS autoimmune disease. Mice with astrocyte-specific *JAM-A* deletion (*mGFAP:CreJAM-A*$^{fl/fl}$) exhibit a failure of T cells to infiltrate the CNS parenchyma from the perivascular spaces (PVS) along with a milder histopathological and clinical course of disease compared to wild-type controls (*JAM-A*$^{fl/fl}$). Treatment of wild-type mice with intraperitoneal injection of soluble JAM-A blocking peptide (JAM-Ap) protects against EAE, highlighting the potential of astrocyte-immune cell signaling as a novel translational target against neuroinflammatory disease.

For Review Only

Introduction:

In multiple sclerosis (MS) and other autoimmune diseases of the central nervous system (CNS), immune cells inappropriately invade the CNS from the bloodstream and drive inflammatory lesion formation (Frischer *et al.*, 2015; Lassmann, 2018). CNS entry is a two-step process during which immune cells first cross the endothelial blood-brain barrier, using contact-mediated

interactions with the endothelial surface to traffic into a compartment termed the perivascular space (PVS) (Abbott *et al.*, 2006; Engelhardt and Ransohoff, 2012; Schlager *et al.*, 2016). Within the PVS, immune cells encounter the glia limitans (GL), a barrier of astrocytic endfeet through which cells must subsequently cross to reach the CNS parenchyma and inflict damage (Owens *et al.*, 2008).

Interactions between the astrocytic endfeet and immune cells within the PVSs have been minimally explored despite their potential significance in regulating the autoimmune response. Cross-talk is known to involve leukocyte matrix metalloproteases (MMP-2 and MMP-9) in degrading PVS basement membranes, enabling the parenchymal entry of infiltrating immune cells during EAE (Song *et al.*, 2013; Song *et al.*, 2015; Gerwien *et al.*, 2016). Astrocytic VCAM-1 has also been identified as a TNFR1 induced cell adhesion molecule critical for immune cell trafficking past the GL and into the CNS parenchyma during EAE (Gimenez *et al.*, 2004; Gimenez *et al.*, 2006; Laureys *et al.*, 2014). Moreover, astrocytic VCAM-1 is modulated in a region-specific manner by effector T cell (Th1 and Th17) secreted factors accounting for regional differences in immune cell infiltration during EAE (Williams *et al.*, 2020). Recently, tissue resident CD8 T cells were found within multiple sclerosis lesions to express PD-1 while reactive astrocyte endfeet express PD-1 ligand, suggestive of potential inhibitory interactions within the PVS (Smolders *et al.*, 2018). Therefore, both soluble and contact-mediated signals between astrocytes and immune cells within the PVS may play a significant role in regulating CNS autoinflammatory disease.

We previously reported that reactive astrocytes upregulate Junctional Adhesion Molecule-A (JAM-A), an immunoglobulin-like cell-surface receptor, in response to the proinflammatory

For Review Only

cytokine, interleukin-1 beta (IL-1β) *in vitro* as well as in *in vivo* models of CNS inflammation. JAM-A has a dual role: 1) initiating and stabilizing tight junction complexes via homophilic binding between identical cell types (Weber *et al.*, 2007; Luissint *et al.*, 2014; Kummer and Ebnet, 2018) and 2) serving as an immune cell surface receptor via heterophilic binding to LFA-1 on T cells and monocytes (Nourshargh *et al.*, 2006). In gut and CNS vascular endothelium, JAM-A binds to immune cells to induce intracellular signal transduction pathways and promote transmigration through the endothelial layer, ultimately leading to a pro-inflammatory, tissue damaging state (Engelhardt and Ransohoff, 2012; Lakshmi *et al.*, 2012; Schmitt *et al.*, 2014; Sladojevic *et al.*, 2014; Flemming *et al.*, 2018; Fan *et al.*, 2019; Luissint *et al.*, 2019).

We hypothesized that astrocytic JAM-A interacts with immune cells within the PVS to promote effector pathways of CNS inflammation and tissue damage. Here, we focused on the T cell population given its central role in driving pathogenesis of EAE, an animal model of CNS autoimmune demyelinating disease. We first characterized the effects of astrocytic JAM-A on protease and cytokines implicated in EAE and MS pathogenesis. Then, using a genetic mouse model in which JAM-A is selectively deleted from reactive astrocytes (*mGFAP:CreJAM-A*$^{fl/fl}$) compared to unaffected (*JAM-A*$^{fl/fl}$) controls, we investigated how astrocytic JAM-A regulates lesion pathology in two models of CNS inflammation and its effects on clinical disability in EAE (Cera *et al.*, 2004; Garcia *et al.*, 2004). Finally, we tested the therapeutic potential of an exogenously administered JAM-A blocking peptide in EAE.

Results:

For Review Only

Astrocytic JAM-A is upregulated diffusely on the astrocytic cell surface in response to interleukin-1β *in vitro* and in *in vivo* models of CNS inflammatory disease

We reported previously that reactive astrocytes upregulate the tight junction proteins, Claudin-1 (Cldn-1), Claudin-4 (Cldn-4) and JAM-A in response to CNS inflammation (Horng *et al.*, 2017). In CNS vascular and gut endothelial cells, JAM-A acts both as a tight junction molecule in trans dimeric form and as an immune cell receptor in monomeric form (Ebnet *et al.*, 2004; Wojcikiewicz *et al.*, 2009; Stamatovic *et al.*, 2012). In vascular endothelium, the cytokine CCL-2 serves as a switch, causing JAM-A internalization from the tight junction and relocalization to the cell surface as a monomer (Stamatovic *et al.*, 2012; Sladojevic *et al.*, 2014).

Using human astrocyte cultures, we confirmed that treatment with IL-1β induced JAM-A expression by 6 and 24 hours (**Figure 1A, Supplemental Figure 1**). Upon induction, astrocytic JAM-A not only co-localized with the tight junction protein occludin but was also distributed more diffusely throughout the cell membrane (**Figure 1A, Supplemental Figure 1**). Treatment with CCL-2 alone did not induce JAM-A expression and combined treatment with IL-1β and CCL-2 did not augment or change the distribution of JAM-A compared to IL-1β alone (**Figure 1A, Supplemental Figure 1**). Therefore, astrocytic JAM-A is expressed diffusely as a monomeric protein from the time of its upregulation by IL-1β.

We characterized the expression of astrocytic JAM-A in two models of CNS inflammation in the mouse. In resting (ie. healthy) cortex, JAM-A was not expressed in astrocytes of the CNS parenchyma. It was most strongly detected in a pattern matching that of the vascular endothelium,

For Review Only

consistent with previous studies (**Figure 1B, C**) (Padden *et al.*, 2007; Stamatovic *et al.*, 2012; Sladojevic *et al.*, 2014; Bhowmick *et al.*, 2019). In asymptomatic inflammatory lesions produced by intracortical injections of IL-1β expressing adenovirus (AdIL-1), reactive astrocytes expressed JAM-A, most prominently within the endfoot processes encircling blood vessels (**Figure 1B, D**). In inflammatory demyelinating spinal cord lesions of EAE, astrocytic JAM-A was present in the endfeet of perivascular astrocytes, as well as on the surface of infiltrating leukocytes (**Figure 1E**). JAM-A was not expressed by microglial cells (Iba1) or oligodendrocytes (MBP) in AdIL-1 and EAE lesions (**Supplemental Figure 2**). Astrocyte specific knock-down of JAM-A was demonstrated in AdIL-1 and EAE lesions, but not in healthy cortex, of conditional knock-out (*mGFAP:CreJAM-A$^{fl/fl}$*, CKO) mice compared to littermate wild-type (*JAM-A$^{fl/fl}$*, WT) controls (**Figure 1C-E**)

Astrocytic JAM-A increases levels of pro-inflammatory cytokines and proteases critical for CNS autoinflammatory disease

Local protease and cytokine levels within the PVS play a critical role in facilitating immune cell priming, CNS entry and autoimmune attack (Song *et al.*, 2015; Williams *et al.*, 2020). We tested whether astrocytic JAM-A leads to changes in protease and cytokine levels in an astrocyte-T cell co-culture system. Here, we used a pan-T cell population (CD3+) to assess the net effects of astrocytic JAM-A signaling to both CD4+ and CD8+ T cells. ELISA arrays were performed on co-cultures of activated (IL-1β treated) human astrocytes and CD3+ T cells in the presence or absence of astrocytic JAM-A. Supernatants extracted from co-cultures with *siJAM-A* transfected astrocytes showed statistically significant decreases in MMP-2 (**Figure 2A**) and GM-CSF (**Figure 2D**), both factors

For Review Only

previously shown to promote EAE pathogenesis (dos Santos *et al.*, 2005; Agrawal *et al.*, 2006; Kroenke *et al.*, 2010; Rasouli *et al.*, 2015; Song *et al.*, 2015; Gerwien *et al.*, 2016; Levesque *et al.*, 2016; Ifergan *et al.*, 2017; Imitola *et al.*, 2018; Galli *et al.*, 2019; Monaghan and Wan, 2020; Wheeler *et al.*, 2020). Lysates of *siJAM-A* transfected astrocytes showed decreased levels of ADAM9, cathepsin C and CCL-2 (**Figures 2B, E**), the last of which is known to promote EAE pathogenesis via its chemotactic effects on infiltrating monocytes (Ge *et al.*, 2012; Kim *et al.*, 2014). Lysates of CD3+ T cells showed no significantly significant protease or cytokine changes (**Figures 2C, F**). In sum, astrocytic JAM-A expression in co-culture with CD3+ T cells leads to increased levels of MMP-2, CCL-2 and GM-CSF, EAE promoting signals involved in both immune cell infiltration into the CNS parenchyma and pathogenic T cell activity.

Astrocytic JAM-A regulates immune cell infiltration past the perivascular space in IL-1β induced cortical lesions

To test whether astrocytic JAM-A facilitates immune cell infiltration past the PVS, we characterized patterns of immune cell entry in asymptomatic cortical lesions induced by intracortical AdIL-1 injection. Lesion size after AdIL-1 injection, measured as area of neuronal loss, showed a decreasing trend not reaching statistical significance in CKOs compared to WTs (**Figures 3A, B**). Lesions in CKO mice demonstrated on average more CD4+ immune cells than the WT group (**Figures 3C, D**). However, CD4+ immune cells in CKOs were restricted to the PVS, as demarcated by pan-laminin staining (**Figures 3C, E, F**). By contrast, in WT mice, the majority of CD4+ cells were located in the parenchyma, indicating successful migration out of the laminin-rich basement membranes of the PVS (**Figures 3C, E, F**). Therefore, astrocytic JAM-A facilitated CD4+ immune cell infiltration past the

For Review Only

glia limitans and into the CNS parenchyma in cortical AdIL-1induced lesions.

Astrocytic JAM-A promotes disease severity and demyelinating neuropathology in EAE

To test the role of astrocytic JAM-A in a model of CNS autoimmune demyelinating disease, EAE was induced in *JAM-A$^{fl/fl}$* (WT), *mGFAP:CreJAM-A$^{fl/fl}$* (CKO), and *JAM-A$^{-/-}$* (KO) mice. CKO and KO mice were studied to dissociate astrocytic JAM-A loss from total JAM-A deletion. CKO and KO mice both showed milder courses of disease, including lower average and peak disease scores compared to WT mice (**Figures 4A-C**). Mortality was significantly reduced in both CKO and KO mice (**Figure 4D**) while rates of resistance to disease induction showed a greater, non-statistically significant trend in CKO and KO mice compared to WTs (**Figure 4E**). Of note, the course and severity of disease did not differ between CKO and KO mice. To eliminate confounding mechanisms from JAM-A deletion in other tissues and cell types, we decided to focus on neuropathology in the CKO line.

Immunohistopathology was performed in *mGFAP:CreJAM-A$^{fl/fl}$* (CKO) and *JAM-A$^{fl/fl}$* (WT) to measure patterns of immune cell infiltration, demyelination, neuronal cell death and astrocytic activation. (**Figures 4F-H, M-R**). At day 21 post EAE immunization, WT mice showed a typical distribution of CD4+ and CD45+ immune cells throughout the CNS parenchyma while CKOs showed accumulation, or cuffing, of cells within the perivascular spaces (PVS) and decreased infiltration of cells into the parenchyma (**Figures 4F-H**). To test whether the absence of astrocytic JAM-A and PVS cuffing altered the total number of CD3+ and CD4+ T cells entering the CNS during EAE, flow cytometry was performed in CKOs and WTs at 5 days from disease onset during the ascending phase of disease. No difference in total CD3+ T cell number was found in the spinal cord (**Figures 4I, K**) or spleen of CKO

For Review Only

mice compared to WTs (**Figure 4I, K**). In the spinal cord, CD4+ T cell counts showed a decreasing trend not reaching statistical significance in CKOs compared to WTs (**Figure 4J**). In the spleen, CD4+ counts were not significantly different between groups (**Figure 4L**). Therefore, astrocytic JAM-A deletion affects the spatial distribution but not total number of T cells within the CNS.

Further immunohistochemical analysis revealed that CKO mice were protected against subsequent neuropathological hallmarks of EAE. At day 28 post-immunization, CKO mice showed decreased demyelination (**Figures 4M, N**), neuronal cell death (**Figures 4O, P**) and astrocytic activation (**Figures 4Q, R**), corresponding to a milder course of clinical disability and similar to other genetic models (Korner *et al.*, 1997; Song *et al.*, 2015) in which immune cell trapping within the PVSs prevents parenchymal damage and clinical disability.

To confirm the translational potential of JAM-A blockade, WT mice were treated with daily intraperitoneal injection of either a soluble JAM-A blocking peptide (JAM-Ap) specifically targeting the monomeric form or a scramble non-targeting peptide starting at day 7 of EAE. Treatment with JAM-Ap demonstrated a protective effect against clinical disability in EAE compared to the scramble control (**Figure 4S**).

Discussion:

The glia limitans (GL) is the final barrier separating peripheral infiltrating immune cells and soluble factors from the CNS parenchyma (Abbott *et al.*, 2006; Engelhardt and Coisne, 2011). The perivascular spaces (PVSs) therefore represent the penultimate compartment for incoming cells and

For Review Only

factors during CNS autoinflammatory disease. Contact-mediated signals between the astrocytic end-feet of the GL and immune cells have the potential to act as critical checkpoints for both 1) the entry of inflammatory cells into the CNS parenchyma from the PVS and 2) the functional differentiation of both cell types in the inflammatory context (De Keyser *et al.*, 2010; Sofroniew, 2015; Prajeeth *et al.*, 2017; Liddelow and Sofroniew, 2019; Williams *et al.*, 2020). Here, we demonstrate a novel astrocyte-T cell interaction that modulates lymphocyte trafficking into the CNS parenchyma with downstream effects on histopathological damage and clinical disability. Experiments using functional gene network analysis to characterize how the astrocytic JAM-A mediated interaction between astrocytes and T cells regulates the functional differentiation of both cell types are currently underway.

JAM-A is an immunoglobulin-like cell surface receptor with well-characterized roles in tight junction formation, endothelial diapedesis and immune cell signal transduction in vascular, gut and lung endothelial cells (Weber *et al.*, 2007; Luissint *et al.*, 2014; Kummer and Ebnet, 2018; Hartmann *et al.*, 2020). We demonstrated that astrocytes upregulate JAM-A *in vitro* in response to IL-1β, a critical pro-inflammatory cytokine in multiple sclerosis and EAE pathogenesis, and *in vivo* during EAE and intracortical injection of AdIL-1. Previous work detailing JAM-A expression within active multiple sclerosis lesions, noted patterns within the blood brain barrier that appear to conform to upregulated expression within the astrocytic endfeet (Padden *et al.*, 2007).

Protease and cytokine ELISA experiments confirmed that astrocytic JAM-A increases proinflammatory effector proteins MMP-2, CCL-2 and GM-CSF in co-culture with a CD3+ T cell population. These factors have previously been demonstrated to promote EAE pathogenesis and multiple sclerosis lesion formation; MMP-2 by facilitating immune cell migration out of the PVS and

For Review Only

into the CNS parenchyma (Agrawal *et al.*, 2006; Song *et al.*, 2015; Gerwien *et al.*, 2016), CCL-2 through its chemotactic effects on infiltrating monocytes (Ge *et al.*, 2012; Kim *et al.*, 2014) and GM-CSF via its effects on monocyte recruitment and pathogenic T cell activity in the acute phase (Ponomarev *et al.*, 2007; Kroenke *et al.*, 2010; Kara *et al.*, 2015) with pleiotropic effects on tissue damage in the chronic phase (Duncker *et al.*, 2018).

Comparing conditional JAM-A knock out mice and controls, we found that astrocytic JAM-A promotes the entry of T cells into the CNS parenchyma in two *in vivo* models of CNS inflammation and that astrocytic deletion of JAM-A protects against clinical disability and histopathological damage during EAE. Astrocyte specific and total JAM-A deletion showed similar phenotypes suggesting that astrocytic JAM-A may fully account for its pathogenic effects in EAE, though this does not rule out the additional possibility of both pathogenic and protective effects of JAM-A in other tissues, including the intestinal epithelium and spleen. Exogenous administration of a soluble JAM-A blocking peptide protected against EAE, demonstrating a net protective effect and translational potential of blocking astrocyte-immune cell interactions during autoimmune attack.

For Review Only

The extent to which T cell activation and differentiation is influenced by local signaling interactions within the PVS has yet to be determined. Experiments measuring the relative proportions of suppressor (Treg and Th2) and proinflammatory (Th1, Th17, GM-CSF secreting) helper T cell subsets in JAM-A CKOs and WTs are now in progress to determine whether astrocytic JAM-A-mediated signaling has the capacity to modulate T cell differentiation patterns. Additional potential immunomodulatory players within the PVS include not only the astrocytic endfeet, but also pericytes, microglial processes, migrating oligodendrocyte precursors, basement

membrane components and other circulating immune cells including dendritic cells, macrophages and B cells.

Astrocytes have the capacity to both promote and protect against CNS autoinflammatory disease (Cekanaviciute *et al.*, 2014; Mayo *et al.*, 2014; Anderson *et al.*, 2016; Levine *et al.*, 2016; Rothhammer *et al.*, 2016; Liddelow *et al.*, 2017; Chhatbar *et al.*, 2018; Itoh *et al.*, 2018; Tassoni *et al.*, 2019; Barbar *et al.*, 2020; Wheeler *et al.*, 2020; Williams *et al.*, 2020). In their reactive state, astrocytes drive both acute and chronic phases of neuroinflammation, and contribute to the transition from a neuroinflammatory to a neurotoxic, or neurodegenerative, state (Rothhammer et al., 2016, Wheeler et al., 2020, Liddelow et al., 2017). Conversion from acute inflammatory injury to a chronic neurodegenerative state is a clinical hallmark of secondary progressive MS and also occurs in a range of other neurologic diseases, including ischemic stroke and dementia (Cekanaviciute and Buckwalter, 2016; Arranz and De Strooper, 2019; Guerrero-Garcia, 2020). Future work defining the temporal dynamics of astrocytic JAM-A signaling and other receptor-mediated astrocyte-immune cell interactions within the PVSs will help us to understand how acute neuroinflammatory changes may prime the CNS for longitudinal injury or repair, leading to novel translational strategies for progressive MS and other neurodegenerative diseases.

For Review Only

Summary:

Astrocytic JAM-A increases MMP-2, CCL-2 and GM-CSF in co-culture with T cells and promotes the migration of T cells out of the perivascular spaces and into the parenchyma in an animal model of CNS autoimmune disease, exacerbating inflammatory histopathology and clinical

disability. Exogenous administration of soluble JAM-A blocking peptide protects against EAE demonstrating that blockade of astrocyte-immune cell signaling within the perivascular space represents a novel therapeutic strategy against multiple sclerosis (MS) and other CNS autoimmune diseases.

Materials and Methods:

Cell mono-culture: astrocytes. Primary human fetal astrocytes were obtained from Lonza (CC-2565) and grown to confluence on glass confocal plates (Mat-Tek, P35GC-1.5-14C) in Astrocyte Growth Medium (AGM). AGM was comprised of MCDB 131 Medium (Gibco 10372-019) and Astrocyte BulletKit factors (Lonza, CC-3186), providing for 3% FBS, 2mM L-glutamine, 30ug/ml gentamicin and 15ng/ml amphotericin (GA-1000), 70uM ascorbic acid, 3ng/ml rhEGF and 7.5ug/ml insulin. Astrocytes were then pre-treated with MCDB 131 alone for 24 hours and then treated with 20ng/ml human recombinant interleukin-1 beta (IL-1β), CCL-2, IL-1β + CCL-2 or vehicle for 6 and 24 hours and then were fixed in ice cold 4% paraformaldehyde (PFA)-1x PBS for 30 minutes then processed for immunohistochemical staining.

Cell co-culture: astrocytes. Primary human fetal astrocytes were plated to 70% confluence on a $20cm^2$ tissue culture dish (Corning, 353003) in AGM. Astrocytes were washed with PBS twice, dissociated gently with 0.05% trypsin, centrifuged, resuspended and nucleofected with 2 μM siRNA of either non-targeting (*siNT*) or *JAM-A (siJAM-A)* targeting sequences, as detailed below. Transfected astrocytes were re-plated and allowed to grow for 24 hours in AGM. Astrocytes were then serum-starved in MCDB 131 for 24 hours and then treated with 20ng/ml IL-1β for 24 hours.

MCDB 131 was refreshed and astrocytes were then paired with 1-2 × 10^6 isolated CD3+ T lymphocytes on a $20cm^2$ tissue culture dish for 24 hours. After co-culture, $CD3^+$ T lymphocytes, astrocytes and supernatants were separated, sonicated and stored at -20°C for protease and cytokine array experiments.

Cell co-culture: T lymphocytes. Human T lymphocytes were extracted from human blood of healthy adult donors freshly collected in lavender K2-EDTA tubes (BD #367861). Briefly, peripheral blood mononuclear cells (PBMCs) were isolated from whole blood samples using density centrifugation with Ficoll-Paque PLUS (GE Healthcare). Six milliliters of whole blood were diluted with an equal volume of HBSS (Mediatech Inc.) and layered onto 15-ml tubes prefilled with 4 ml of density gradient medium. Tubes were centrifuged for 1 hour at 620 relative centrifugal force (rcf). PBMCs were collected from their density gradient layer using a transfer pipette, washed in HBSS (Mediatech Inc.) and centrifuged for 15-20 minutes at 620 rcf x 2. The PBMC pellet was resuspended in eluent buffer and processed with a magnetic labeling and separation protocol using a human pan-T cell ($CD3^+$) (Miltenyi, 130-096-535). Cells were then activated in lymphocyte growth medium ((LGM), comprised of RPMI 1640 (Gibco), 10% FBS, 2mM L-glutamine, 1% 2-mercaptoethanol) at 37°C for 72 hours with 4ug/ml anti-CD28 (eBioscience 16-0298-85) on $20cm^2$ tissue culture dishes pre-treated with 7ug/ml anti-$CD3^+$ (eBioscience 16-0037-85) in PBS at 37°C for 2 hours. After activation, T lymphocytes were centrifuged and 1-2 × 10^6 cells applied to astrocyte cultures for 24 hours before sample separation and processing, as above.

Protease and Cytokine Arrays. Reactive astrocyte and $CD3^+$ T lymphocyte co-cultures were prepared as outlined above. Culture medium supernatant with $CD3^+$ T lymphocytes was aspirated from co-cultures after 24 hours. Aspirant was centrifuged at 620 rcf for 7 minutes, then supernatant stored at 20°C. The $CD3^+$ T lymphocyte pellet was reconstituted and harvested in cell lysis buffer, which was sonicated and then stored at 20°C. Adherent astrocytes from the 20cm^2 tissue culture dish were harvested in cell lysis buffer, sonicated and then stored at 20°C. Supernatant (500ul), T lymphocyte (100ug) and astrocyte (100ug) samples were then applied to human protease (R&D, ARY021B) and cytokine (R&D, ARY005B) ELISA array kits per the manufacturer's instructions in 3 biological triplicates. Quantification of protease signal was performed by densitometry as follows: nonsaturated developed films were scanned using a Canon LiDE scanner (Canon USA), and mean pixel density of each duplicate array probe was measured using ImageJ software (NIH). Data were standardized to 3 duplicated reference probes, and log_2fold change was calculated.

Chemical and Protein Reagents. Human IL-1β and CCL-2 were purchased from PeproTech and used at 20 ng/ml and 10ng/ml, respectively, for mono-culture experiment described above.

JAM-Ap. JAM-A blocking peptide (JAM-Ap) and control peptide were synthesized to order at >95% purity from New England Biopeptide with the following sequences: NPKSTRAFSNDDYVLNPTTG for JAMA-p and NLFSVDTPNGKTASDNYPRT for control, as designed and characterized by a previous group (Sladojevic *et al.*, 2014). Daily intraperitoneal injection of 1ug in 0.1ml of sterile 0.9% NaCl starting on Day 7 was performed in EAE experiments.

Antibodies. Catalog numbers and concentrations of all antibodies are as follows. Anti-GFAP (130300, rat, 1:200), anti-occludin (rabbit, 1:125), anti-IgG (A11029, mouse, 1:100) were from Invitrogen. Anti–JAM-A (sc53623, mouse, 1:100) was from Santa Cruz Biotechnology. Anti-fibrinogen (A0080, rabbit, 1:150) was from Dako. Anti-CD3 (16-0037-85), anti-CD4 (14-9766-82), anti-CD11b (14-0112-82), and anti-CD45 (550539, all rat) were from eBioscience. Anti-CD4 (ab183685, mouse, 1:50) and anti-CD8 (ab217344, rabbit, 1:50) were from Abcam. Anti-NeuN (MAB377, mouse, 1:100) and anti–myelin basic protein (MBP) (MAB386, rat, 1:500) were from Millipore. Anti-laminin (L9393, rabbit, 1:200) was from Sigma-Aldrich.

siRNA. Human astrocyte cultures were nucleofected with siRNA (2 μM) with non-targeting sequences (*siNT*) or *JAM-A (siJAM-A)* targeting sequences (Thermo Scientific Dharmacon, siGENOME SMART pool), using an Amaxa nucleofector (program A033) with the Basic Glial Kit (Amaxa). The extent and specificity of gene silencing was confirmed by immunoblotting as reported in a previous study (Horng *et al.*, 2017).

Mice. *mGfap-Cre* (B6.Cg-Tg(Gfap-cre)73.12Mvs/J) mice were genetically engineered in the laboratory of Michael Sofroniew (UCLA) and are available for purchase from Jackson laboratories (https://www.jax.org/strain/012886). Cre expression is astrocyte-specific except in areas of adult neurogenesis, where it is also observed in some neural progenitors (Garcia *et al.*, 2004). *JAM-A$^{f/fl}$* mice were obtained from Charles Parkos (University of Michigan, Ann Arbor, Michigan, USA) and Terence Dermody (University of Pittsburgh, Pittsburgh, Pennsylvania, USA) (Cera *et al.*, 2004; Laukoetter *et al.*, 2007). For all experiments, *mGfap-Cre JAM-*

$A^{fl/fl}$ female mice were crossed with *JAM-$A^{fl/fl}$* male mice to generate 50% *mGfap-Cre JAM-$A^{fl/fl}$* (conditional knock-out (CKO) mice) and 50% *JAM-$A^{fl/fl}$* (wild type (WT)) littermate controls. Selective deletion of JAM-A in GFAP positive cells was confirmed in a previous study (Horng *et al.*, 2017). Total JAM-A knock out (KO) mice were generated by breeding *mGfap-Cre JAM-$A^{fl/fl}$* male mice (which express Cre in germline cells) to *JAM-$A^{fl/fl}$* to create JAM-$A^{fl/-}$ mice which were then crossed to create JAM-$A^{-/-}$. Genotyping primers were: *mGfap-Cre* forward (GfF) ACC AGC CAG CTA TCA ACT C, reverse (GfR) TAT ACG CGT GCT AGC GAA GAT CTC CAT CTT CCA GCA G, 350 bp; *JAM-A* forward (JaKOF) TCT TTT CAC CAA TCG GAA CG, reverse (JF2R) AAA AAC TCT AGG AAC TCA CCC AGG A, band 200 bp (wt), 320 bp (flox); *JAM-A* excised forward (TS379) CCT CTC TTT TCA CCA ATC GGA, JAM-A excised reverse (TS512) TCT TCT TCA GAC GCC GAA CCT. PCR conditions for all primer sets were: 94°C for 4 minutes; 35 cycles of 94°C for 30 seconds, 56°C for 30 seconds, and 72°C for 30 seconds; then 72°C for 10 minutes.

Cortical microinjection of AdIL-1. Mice (8–12 weeks old, at least 5 per condition per time point, on the C57BL/6 background) were anesthetized using isoflurane and placed into a stereotactic frame (Kopf). AdIL-1 or AdDL70 control (AdCtrl) (10^6 PFU) was microinjected into the cerebral cortex at y = 1 mm caudal to bregma, x = 2 mm, z = 1.5 mm. Animals were allowed to recover for 7 days and then were sacrificed and perfused with 10ml of 1x PBS and 10ml 4% PFA in1x PBS.

EAE. Mice (male and females, 10–13 weeks old, at least 8 animals per group for each experiment, on the C57BL/6 background) were subcutaneously injected with 0.1 cc of $MOG_{35–}$

For Review Only

(Hooke Laboratories) at cervical and lumbar sites followed by intraperitoneal injection of 0.1 cc pertussis toxin on day 0 and day 1. Mice were rated daily on a standard 5-point motor scale from days 7–21 after induction: 0, no symptoms; 1, floppy tail; 2, hind limb weakness (paraparesis); 3, hind limb paralysis (paraplegia); 4, forelimb and hind limb paralysis; 5, death. Three independent experiments were performed for WT and CKO and two independent experiments for KO.

Flow cytometry. WT and CKO mice were anesthetized and perfused with 5 ml PBS at 4-5 days from onset of disease in EAE (days 14-20 from induction). Spinal cords and spleens were collected in cold PBS and mechanically dissociated. Spleen samples were passed through a 70-µm filter, then incubated in red blood cell (RBC) lysis buffer (BioLegend) for 2 minutes at room temperature and washed with PBS. Spinal cords were passed through a 100-µm filter and separated from myelin using a 60%/30% Percoll gradient. Cell suspensions were then collected, counted, and subjected first to a Zombie Yellow stain (Biolegend, 423103) and wash followed by incubation with FITC-anti-CD3 (Biolegend, 100306) and APC/Cy7-anti-CD4 (Biolegend, 100355) antibodies in cell staining buffer (BioLegend). Cells were washed with FACS buffer (2% FBS in PBS), fixed and permeabilized following manufacturer's instructions for staining using the FIX & PERM® Cell Permeabilization Kit (Invitrogen). Forward scatter and side scatter were used to gate cells excluding debris and cell aggregates, Zombie Yellow was used to exclude dead cells and then percentages of CD3 positive cells were measured with a subsequent gate to CD4. Data was acquired on the Invitrogen™ Attune™ NxT Flow Cytometer and analyzed with FCS Express software (De Novo) at the Flow Cytometry CoRE at Mount Sinai.

Immunohistochemistry. Brains and spinal cords were dissected from animals perfused with 10ml ice cold 1x PBS followed by 10ml 4% PFA-1x PBS, tissues were subjected to 2 hours post-fixation in 4% PFA-1xPBS followed by storage in 30% sucrose-1x PBS at 4 degrees until sectioning. Immunostaining was performed on 25um coronal (brain) and axial (spinal cord) sections. For all antibody staining, sections underwent antigen retrieval in citrate (pH 6.0; 100°C) for 20 minutes. For laminin, CD4 and CD45, sections were treated with 0.5 mg/ml protease XIV (Sigma-Aldrich) at 37°C for 5 minutes. Primary antibodies were used at concentrations ranging from 1:50-1:500. Samples were examined using a Leica Microsystems confocal microscope, and stacks were collected with *z* of 1 μm.

Morphometric analysis. Morphometric analyses were performed using NIH ImageJ and Leica LAS softwares. For studies in vivo, JAM-A, occludin, pan-laminin, Olig2, Iba1, CD4, CD45, fluoromyelin (FM), NeuN and GFAP histochemical stains were analyzed in projections from cortical and spinal cord sections. The same number of images was quantified from 3-5 animals per condition per genotype per time point and 3-5 age- and sex-matched normal controls unless otherwise indicated, and at least 4 random ×10 to ×20 fields in 3-5 sections per animal. Myelin loss, neuronal loss and astrocyte reactivity were quantified by measuring the FM positive area, counting NeuN+ cells and measuring GFAP positive pixels from the total area in each field of interest in matched projections at ×20 magnification.

Statistics. Results are reported as mean ± SEM. Student's *t* test and Mann-Whitney U tests were used to compare two groups of unmatched samples. Kruskal-Wallis H test was used to compare two groups with multiple comparisons. For multiple comparisons in EAE, 2-way ANOVA

followed by Bonferroni post-test correction was used. In all cases, p less than 0.05 was considered significant.

Study approval. Use of commercially available human astrocytes and anonymized human blood donor samples was approved by the IRB at the Icahn School of Medicine at Mount Sinai (ISMMS). Studies using mice were approved by the IACUC at the ISMMS, and adhered to the American Veterinary Medical Association guidelines. The ISMMS has an Animal Welfare Assurance on file with the Office for Laboratory Animal Welfare (Assurance no. A3111-01).

Data Availability Statement:

The data that support the findings of this study are available from the corresponding author, upon reasonable request.

Competing Interests:

The authors have no conflicts of interest to disclose.

CRediT Author contributions:

Candice Chapouly: Conceptualization, Methodology, Validation, Formal analysis, Investigation, Writing – Review and Editing, Visualization, Project administration

For Review Only

Mario Amatruda: Conceptualization, Methodology, Visualization, Validation, Formal analysis, Investigation, Writing – Review and Editing, Visualization, Project administration

Viola Woo: Methodology, Validation, Formal analysis

Farinaz Safavi: Methodology, Formal analysis

Joy Zhang: Methodology, Investigation, Validation, Formal analysis

David Dai: Methodology, Investigation, Validation, Formal analysis

Anthony Therattil: Methodology, Investigation

Chang Moon: Investigation

Alexandra Gordon: Investigation

Charles Parkos: Resources

Sam Horng: Conceptualization, Methodology, Validation, Formal analysis, Investigation, Writing – Original Draft, Review and Editing, Visualization, Supervision, Project administration, Funding acquisition

For Review Only

Acknowledgments:

The authors thank Dr. Anne Schaefer, Dr. Patrizia Casaccia, Dr. Fred Lublin and Dr. Stuart Sealfon for their helpful discussions on this project and the manuscript.

Funding:

This work was supported by grant funding to Dr. Horng: National Institutes of Health (NIH) National Institute of Neurological Diseases and Stroke (NINDS) K08 NS102507-01A1, NIH R25NS079102, a

Career Transition Award by the National Multiple Sclerosis Society and the Conrad N. Hilton Foundation, philanthropic support by the Jayne and Harvey Beker Foundation and a post-doctoral Neuroscience fellowship from the Leon Levy Foundation. Dr. Safavi was supported by a post-doctoral Neuroscience fellowship from the Leon Levy Foundation.

The MSSM Microscopy and Flow Cytometry Shared Resource Facilities were utilized in this study; core facilities receive support from National Institutes of Health/National Cancer Institute Grant R24 CA095823.

For Review Only

References:

Abbott NJ, Ronnback L, Hansson E. Astrocyte-endothelial interactions at the blood-brain barrier. Nat Rev Neurosci 2006; 7(1): 41-53.

Agrawal S, Anderson P, Durbeej M, van Rooijen N, Ivars F, Opdenakker G*, et al.* Dystroglycan is selectively cleaved at the parenchymal basement membrane at sites of leukocyte extravasation in experimental autoimmune encephalomyelitis. J Exp Med 2006; 203(4): 1007-19.

Anderson MA, Burda JE, Ren Y, Ao Y, O'Shea TM, Kawaguchi R*, et al.* Astrocyte scar formation aids central nervous system axon regeneration. Nature 2016; 532(7598): 195-200.

Arranz AM, De Strooper B. The role of astroglia in Alzheimer's disease: pathophysiology and clinical implications. Lancet Neurol 2019; 18(4): 406-14.

Barbar L, Jain T, Zimmer M, Kruglikov I, Sadick JS, Wang M*, et al.* CD49f Is a Novel Marker of Functional and Reactive Human iPSC-Derived Astrocytes. Neuron 2020; 107(3): 436-53 e12.

Bhowmick S, D'Mello V, Caruso D, Wallerstein A, Abdul-Muneer PM. Impairment of pericyte-endothelium crosstalk leads to blood-brain barrier dysfunction following traumatic brain injury. Exp Neurol 2019; 317: 260-70.

Cekanaviciute E, Buckwalter MS. Astrocytes: Integrative Regulators of Neuroinflammation in Stroke and Other Neurological Diseases. Neurotherapeutics 2016; 13(4): 685-701.

Cekanaviciute E, Fathali N, Doyle KP, Williams AM, Han J, Buckwalter MS. Astrocytic transforming growth factor-beta signaling reduces subacute neuroinflammation after stroke in mice. Glia 2014; 62(8): 1227-40.

For Review Only

Cera MR, Del Prete A, Vecchi A, Corada M, Martin-Padura I, Motoike T, *et al.* Increased DC trafficking to lymph nodes and contact hypersensitivity in junctional adhesion molecule-A-deficient mice. J Clin Invest 2004; 114(5): 729-38.

Chhatbar C, Detje CN, Grabski E, Borst K, Spanier J, Ghita L, *et al.* Type I Interferon Receptor Signaling of Neurons and Astrocytes Regulates Microglia Activation during Viral Encephalitis. Cell Rep 2018; 25(1): 118-29 e4.

De Keyser J, Laureys G, Demol F, Wilczak N, Mostert J, Clinckers R. Astrocytes as potential targets to suppress inflammatory demyelinating lesions in multiple sclerosis. Neurochem Int 2010; 57(4): 446-50.

dos Santos AC, Barsante MM, Arantes RM, Bernard CC, Teixeira MM, Carvalho-Tavares J. CCL2 and CCL5 mediate leukocyte adhesion in experimental autoimmune encephalomyelitis--an intravital microscopy study. J Neuroimmunol 2005; 162(1-2): 122-9.

Duncker PC, Stoolman JS, Huber AK, Segal BM. GM-CSF Promotes Chronic Disability in Experimental Autoimmune Encephalomyelitis by Altering the Composition of Central Nervous System-Infiltrating Cells, but Is Dispensable for Disease Induction. J Immunol 2018; 200(3): 966-73.

Ebnet K, Suzuki A, Ohno S, Vestweber D. Junctional adhesion molecules (JAMs): more molecules with dual functions? J Cell Sci 2004; 117(Pt 1): 19-29.

Engelhardt B, Coisne C. Fluids and barriers of the CNS establish immune privilege by confining immune surveillance to a two-walled castle moat surrounding the CNS castle. Fluids Barriers CNS 2011; 8(1): 4.

Engelhardt B, Ransohoff RM. Capture, crawl, cross: the T cell code to breach the blood-brain barriers. Trends Immunol 2012; 33(12): 579-89.

For Review Only

Fan S, Weight CM, Luissint AC, Hilgarth RS, Brazil JC, Ettel M*, et al.* Role of JAM-A tyrosine phosphorylation in epithelial barrier dysfunction during intestinal inflammation. Mol Biol Cell 2019; 30(5): 566-78.

Flemming S, Luissint AC, Nusrat A, Parkos CA. Analysis of leukocyte transepithelial migration using an in vivo murine colonic loop model. JCI Insight 2018; 3(20).

Frischer JM, Weigand SD, Guo Y, Kale N, Parisi JE, Pirko I*, et al.* Clinical and pathological insights into the dynamic nature of the white matter multiple sclerosis plaque. Ann Neurol 2015; 78(5): 710-21.

Galli E, Hartmann FJ, Schreiner B, Ingelfinger F, Arvaniti E, Diebold M*, et al.* GM-CSF and CXCR4 define a T helper cell signature in multiple sclerosis. Nat Med 2019; 25(8): 1290-300.

Garcia AD, Doan NB, Imura T, Bush TG, Sofroniew MV. GFAP-expressing progenitors are the principal source of constitutive neurogenesis in adult mouse forebrain. Nat Neurosci 2004; 7(11): 1233-41.

Ge S, Shrestha B, Paul D, Keating C, Cone R, Guglielmotti A*, et al.* The CCL2 synthesis inhibitor bindarit targets cells of the neurovascular unit, and suppresses experimental autoimmune encephalomyelitis. J Neuroinflammation 2012; 9: 171.

Gerwien H, Hermann S, Zhang X, Korpos E, Song J, Kopka K*, et al.* Imaging matrix metalloproteinase activity in multiple sclerosis as a specific marker of leukocyte penetration of the blood-brain barrier. Sci Transl Med 2016; 8(364): 364ra152.

Gimenez MA, Sim J, Archambault AS, Klein RS, Russell JH. A tumor necrosis factor receptor 1-dependent conversation between central nervous system-specific T cells and the central nervous system is required for inflammatory infiltration of the spinal cord. Am J Pathol 2006; 168(4): 1200-9.

For Review Only

Gimenez MA, Sim JE, Russell JH. TNFR1-dependent VCAM-1 expression by astrocytes exposes the CNS to destructive inflammation. J Neuroimmunol 2004; 151(1-2): 116-25.

Guerrero-Garcia JJ. The role of astrocytes in multiple sclerosis pathogenesis. Neurologia 2020; 35(6): 400-8.

Hartmann C, Schwietzer YA, Otani T, Furuse M, Ebnet K. Physiological functions of junctional adhesion molecules (JAMs) in tight junctions. Biochim Biophys Acta Biomembr 2020; 1862(9): 183299.

Horng S, Therattil A, Moyon S, Gordon A, Kim K, Argaw AT*, et al.* Astrocytic tight junctions control inflammatory CNS lesion pathogenesis. J Clin Invest 2017; 127(8): 3136-51.

Ifergan I, Davidson TS, Kebir H, Xu D, Palacios-Macapagal D, Cann J*, et al.* Targeting the GM-CSF receptor for the treatment of CNS autoimmunity. J Autoimmun 2017; 84: 1-11.

Imitola J, Rasouli J, Watanabe F, Mahajan K, Sharan AD, Ciric B*, et al.* Elevated expression of granulocyte-macrophage colony-stimulating factor receptor in multiple sclerosis lesions. J Neuroimmunol 2018; 317: 45-54.

Itoh N, Itoh Y, Tassoni A, Ren E, Kaito M, Ohno A*, et al.* Cell-specific and region-specific transcriptomics in the multiple sclerosis model: Focus on astrocytes. Proc Natl Acad Sci U S A 2018; 115(2): E302-E9.

Kara EE, McKenzie DR, Bastow CR, Gregor CE, Fenix KA, Ogunniyi AD*, et al.* CCR2 defines in vivo development and homing of IL-23-driven GM-CSF-producing Th17 cells. Nat Commun 2015; 6: 8644.

Kim RY, Hoffman AS, Itoh N, Ao Y, Spence R, Sofroniew MV*, et al.* Astrocyte CCL2 sustains immune cell infiltration in chronic experimental autoimmune encephalomyelitis. J Neuroimmunol 2014; 274(1-2): 53-61.

For Review Only

Korner H, Riminton DS, Strickland DH, Lemckert FA, Pollard JD, Sedgwick JD. Critical points of tumor necrosis factor action in central nervous system autoimmune inflammation defined by gene targeting. J Exp Med 1997; 186(9): 1585-90.

Kroenke MA, Chensue SW, Segal BM. EAE mediated by a non-IFN-gamma/non-IL-17 pathway. Eur J Immunol 2010; 40(8): 2340-8.

Kummer D, Ebnet K. Junctional Adhesion Molecules (JAMs): The JAM-Integrin Connection. Cells 2018; 7(4).

Lakshmi SP, Reddy AT, Naik MU, Naik UP, Reddy RC. Effects of JAM-A deficiency or blocking antibodies on neutrophil migration and lung injury in a murine model of ALI. Am J Physiol Lung Cell Mol Physiol 2012; 303(9): L758-66.

Lassmann H. Pathogenic Mechanisms Associated With Different Clinical Courses of Multiple Sclerosis. Front Immunol 2018; 9: 3116.

Laukoetter MG, Nava P, Lee WY, Severson EA, Capaldo CT, Babbin BA*, et al.* JAM-A regulates permeability and inflammation in the intestine in vivo. J Exp Med 2007; 204(13): 3067-76.

Laureys G, Gerlo S, Spooren A, Demol F, De Keyser J, Aerts JL. beta(2)-adrenergic agonists modulate TNF-alpha induced astrocytic inflammatory gene expression and brain inflammatory cell populations. J Neuroinflammation 2014; 11: 21.

Levesque SA, Pare A, Mailhot B, Bellver-Landete V, Kebir H, Lecuyer MA*, et al.* Myeloid cell transmigration across the CNS vasculature triggers IL-1beta-driven neuroinflammation during autoimmune encephalomyelitis in mice. J Exp Med 2016; 213(6): 929-49.

Levine J, Kwon E, Paez P, Yan W, Czerwieniec G, Loo JA*, et al.* Traumatically injured astrocytes release a proteomic signature modulated by STAT3-dependent cell survival. Glia 2016; 64(5): 668-94.

For Review Only

Liddelow SA, Guttenplan KA, Clarke LE, Bennett FC, Bohlen CJ, Schirmer L*, et al.* Neurotoxic reactive astrocytes are induced by activated microglia. Nature 2017; 541(7638): 481-7.

Liddelow SA, Sofroniew MV. Astrocytes usurp neurons as a disease focus. Nat Neurosci 2019; 22(4): 512-3.

Luissint AC, Nusrat A, Parkos CA. JAM-related proteins in mucosal homeostasis and inflammation. Semin Immunopathol 2014; 36(2): 211-26.

Luissint AC, Williams HC, Kim W, Flemming S, Azcutia V, Hilgarth RS*, et al.* Macrophage-dependent neutrophil recruitment is impaired under conditions of increased intestinal permeability in JAM-A-deficient mice. Mucosal Immunol 2019; 12(3): 668-78.

Mayo L, Trauger SA, Blain M, Nadeau M, Patel B, Alvarez JI*, et al.* Regulation of astrocyte activation by glycolipids drives chronic CNS inflammation. Nat Med 2014; 20(10): 1147-56.

Monaghan KL, Wan ECK. The Role of Granulocyte-Macrophage Colony-Stimulating Factor in Murine Models of Multiple Sclerosis. Cells 2020; 9(3).

Nourshargh S, Krombach F, Dejana E. The role of JAM-A and PECAM-1 in modulating leukocyte infiltration in inflamed and ischemic tissues. J Leukoc Biol 2006; 80(4): 714-8.

Owens T, Bechmann I, Engelhardt B. Perivascular spaces and the two steps to neuroinflammation. Journal of neuropathology and experimental neurology 2008; 67(12): 1113-21.

Padden M, Leech S, Craig B, Kirk J, Brankin B, McQuaid S. Differences in expression of junctional adhesion molecule-A and beta-catenin in multiple sclerosis brain tissue: increasing evidence for the role of tight junction pathology. Acta Neuropathol 2007; 113(2): 177-86.

For Review Only

Ponomarev ED, Shriver LP, Maresz K, Pedras-Vasconcelos J, Verthelyi D, Dittel BN. GM-CSF production by autoreactive T cells is required for the activation of microglial cells and the onset of experimental autoimmune encephalomyelitis. J Immunol 2007; 178(1): 39-48.

Prajeeth CK, Kronisch J, Khorooshi R, Knier B, Toft-Hansen H, Gudi V*, et al.* Effectors of Th1 and Th17 cells act on astrocytes and augment their neuroinflammatory properties. J Neuroinflammation 2017; 14(1): 204.

Rasouli J, Ciric B, Imitola J, Gonnella P, Hwang D, Mahajan K*, et al.* Expression of GM-CSF in T Cells Is Increased in Multiple Sclerosis and Suppressed by IFN-beta Therapy. J Immunol 2015; 194(11): 5085-93.

Rothhammer V, Mascanfroni ID, Bunse L, Takenaka MC, Kenison JE, Mayo L*, et al.* Type I interferons and microbial metabolites of tryptophan modulate astrocyte activity and central nervous system inflammation via the aryl hydrocarbon receptor. Nat Med 2016; 22(6): 586-97.

Schlager C, Korner H, Krueger M, Vidoli S, Haberl M, Mielke D*, et al.* Effector T-cell trafficking between the leptomeninges and the cerebrospinal fluid. Nature 2016; 530(7590): 349-53.

Schmitt MM, Fraemohs L, Hackeng TM, Weber C, Koenen RR. Atherogenic mononuclear cell recruitment is facilitated by oxidized lipoprotein-induced endothelial junctional adhesion molecule-A redistribution. Atherosclerosis 2014; 234(2): 254-64.

Sladojevic N, Stamatovic SM, Keep RF, Grailer JJ, Sarma JV, Ward PA*, et al.* Inhibition of junctional adhesion molecule-A/LFA interaction attenuates leukocyte trafficking and inflammation in brain ischemia/reperfusion injury. Neurobiol Dis 2014; 67: 57-70.

Smolders J, Heutinck KM, Fransen NL, Remmerswaal EBM, Hombrink P, Ten Berge IJM*, et al.* Tissue-resident memory T cells populate the human brain. Nat Commun 2018; 9(1): 4593.

For Review Only

Sofroniew MV. Astrocyte barriers to neurotoxic inflammation. Nat Rev Neurosci 2015; 16(5): 249-63.

Song J, Wu C, Korpos E, Zhang X, Agrawal SM, Wang Y*, et al.* Focal MMP-2 and MMP-9 activity at the blood-brain barrier promotes chemokine-induced leukocyte migration. Cell Rep 2015; 10(7): 1040-54.

Song J, Wu C, Zhang X, Sorokin LM. In vivo processing of CXCL5 (LIX) by matrix metalloproteinase (MMP)-2 and MMP-9 promotes early neutrophil recruitment in IL-1beta-induced peritonitis. J Immunol 2013; 190(1): 401-10.

Stamatovic SM, Sladojevic N, Keep RF, Andjelkovic AV. Relocalization of junctional adhesion molecule A during inflammatory stimulation of brain endothelial cells. Mol Cell Biol 2012; 32(17): 3414-27.

Tassoni A, Farkhondeh V, Itoh Y, Itoh N, Sofroniew MV, Voskuhl RR. The astrocyte transcriptome in EAE optic neuritis shows complement activation and reveals a sex difference in astrocytic C3 expression. Sci Rep 2019; 9(1): 10010.

Weber C, Fraemohs L, Dejana E. The role of junctional adhesion molecules in vascular inflammation. Nat Rev Immunol 2007; 7(6): 467-77.

Wheeler MA, Clark IC, Tjon EC, Li Z, Zandee SEJ, Couturier CP*, et al.* MAFG-driven astrocytes promote CNS inflammation. Nature 2020; 578(7796): 593-9.

Williams JL, Manivasagam S, Smith BC, Sim J, Vollmer LL, Daniels BP*, et al.* Astrocyte-T cell crosstalk regulates region-specific neuroinflammation. Glia 2020; 68(7): 1361-74.

Wojcikiewicz EP, Koenen RR, Fraemohs L, Minkiewicz J, Azad H, Weber C*, et al.* LFA-1 binding destabilizes the JAM-A homophilic interaction during leukocyte transmigration. Biophys J 2009; 96(1): 285-93.

For Review Only

Figure 1

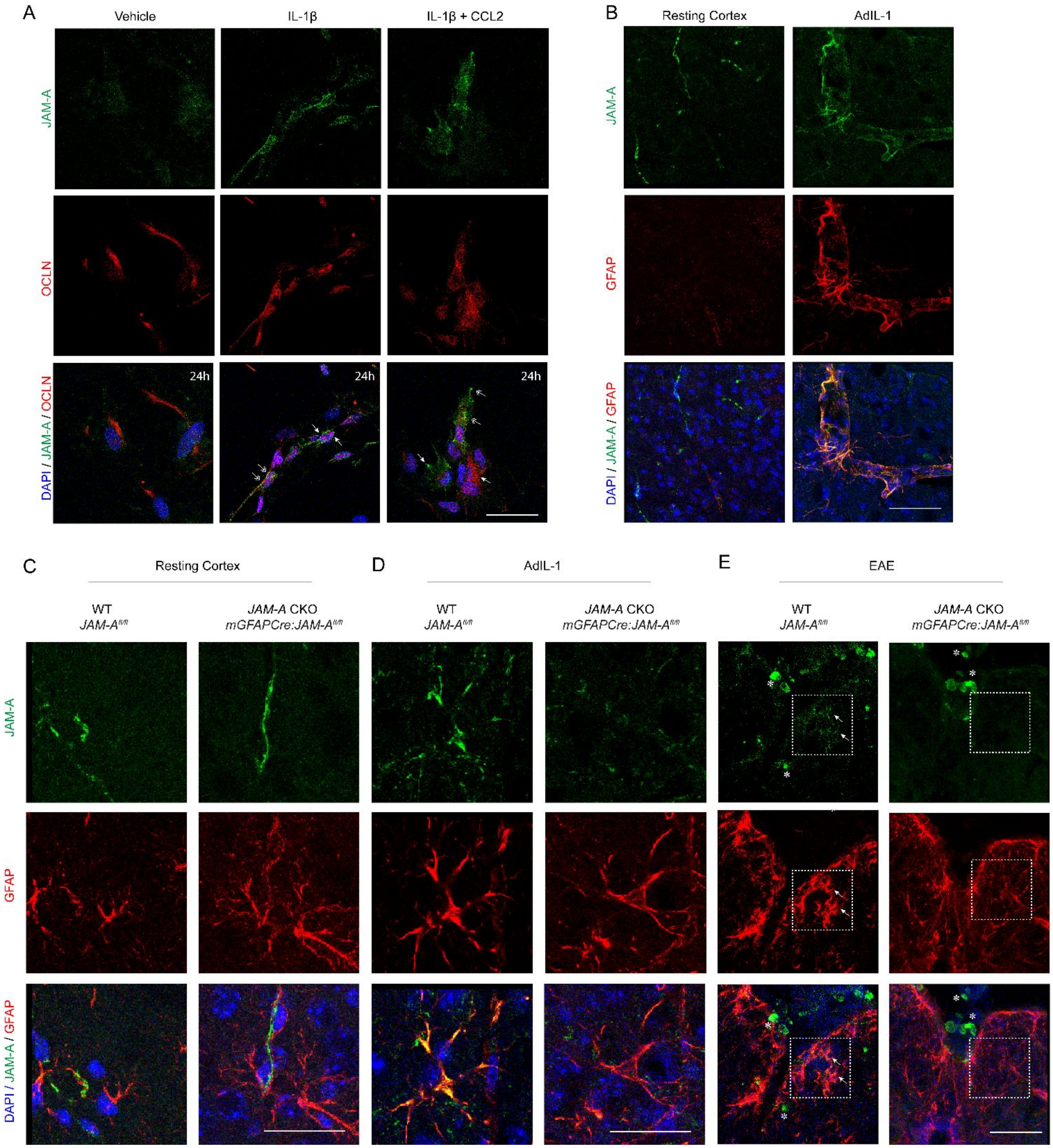
A
Vehicle
IL-1β
IL-1β + CCL2
JAM-A
OCLN
DAPI / JAM-A / OCLN
24h
B
Resting Cortex
AdIL-1
JAM-A
GFAP
DAPI / JAM-A / GFAP
C
Resting Cortex
D
AdIL-1
E
EAE
WT
JAM-A fl/fl
JAM-A CKO
mGFAPCre:JAM-A fl/fl
JAM-A
GFAP
DAPI / JAM-A / GFAP

Figure 1: **Inflammation induces reactive astrocytes to express JAM-A diffusely throughout the cell surface membrane *in vitro* and *in vivo* and this expression was successfully prevented using an astrocyte-specific JAM-A knock-out mouse line.** (A) Exposure of astrocytes cultured *in vitro* to 20ng/ml of IL-1β for 24 hours induces astrocytic expression of JAM-A (green), which is both diffusely localized throughout the cell membrane (white arrows) and co-localized with the tight junction marker, occludin (OCLN, red; white double headed arrows pointing to overlay of the two proteins in yellow). The addition of CCL-2 did not change the distribution of JAM-A. Scale bar 50 µm. Results were confirmed in at least 3 fields of view on two to three technical replicates in each group. (B) In healthy (resting) cortex, both JAM-A (green) and GFAP (red) are minimally expressed and do not co-localize. After intracortical injection of IL-1β expressing adenovirus (AdIL-1), JAM-A (green) is detected most prominently in the reactive astrocytic endfeet (GFAP, red; co-localization, yellow) encircling the blood vessel wall. Scale bar 50 µm. (C-E) Expression patterns of JAM-A and GFAP in resting cortex, AdIL-1 and EAE spinal cord tissue of control (WT) ($JAMA^{fl/fl}$), and *JAM-A* conditional knock-out (CKO) ($mGFAPCre{:}JAMA^{fl/fl}$) mice. In the resting cortex (C), JAM-A (green) of the vascular endothelium abuts the astrocytic endfeet (GFAP, red) but is not expressed by astrocytes. In the inflamed cortex of WT AdIL-1 brains (D), JAM-A is expressed by astrocytes, co-localizing with GFAP. This astrocytic pattern of JAM-A expression is lost in CKOs. Scale bars in (C) and (D) 25 µm. In EAE spinal cord (E), JAM-A (green) is expressed in WTs by reactive astrocytes (GFAP, red) of the glia limitans (area of interest outlined in the dotted white box with white arrows pointing to JAM-A within the astrocytic processes), as well as by leukocytes infiltrating the subarachnoid space and CNS parenchyma (white asterisks). In CKOs, leukocytes, but not astrocytes, express JAM-A (white asterisks). Scale bar in (E) 50 µm.

For Review Only

Figure 2

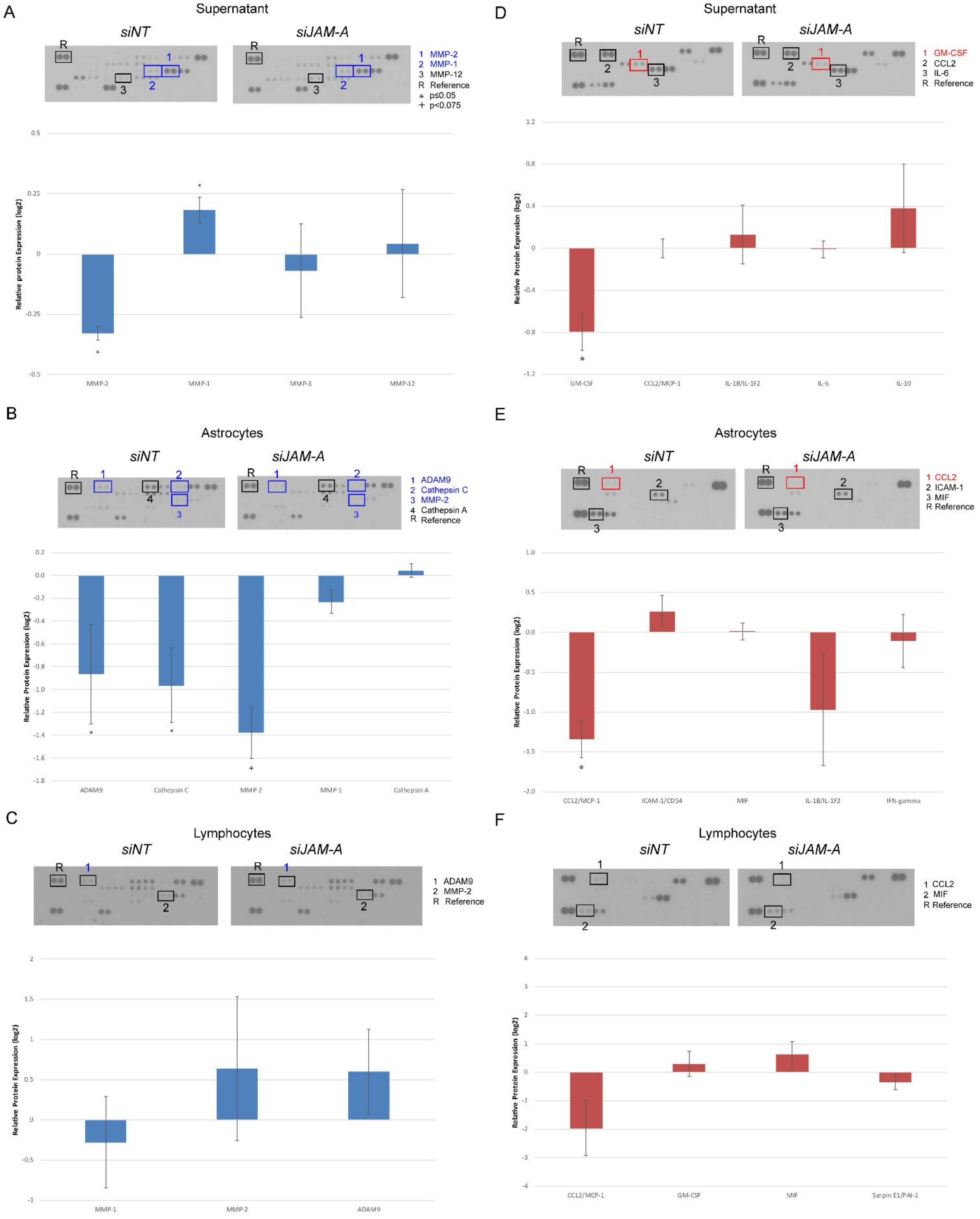
A
Supernatant
siNT
siJAM-A
R
1
2
3
1 MMP-2
2 MMP-1
3 MMP-12
R Reference
* p≤0.05
+ p<0.075
Relative protein Expression (log2)
MMP-2
MMP-1
MMP-3
MMP-12
B
Astrocytes
siNT
siJAM-A
1 ADAM9
2 Cathepsin C
3 MMP-2
4 Cathepsin A
R Reference
Relative Protein Expression (log2)
ADAM9
Cathepsin C
MMP-2
MMP-1
Cathepsin A
C
Lymphocytes
siNT
siJAM-A
1 ADAM9
2 MMP-2
R Reference
Relative Protein Expression (log2)
MMP-1
MMP-2
ADAM9
D
Supernatant
siNT
siJAM-A
1 GM-CSF
2 CCL2
3 IL-6
R Reference
Relative Protein Expression (log2)
GM-CSF
CCL2/MCP-1
IL-1B/IL-1F2
IL-6
IL-10
E
Astrocytes
siNT
siJAM-A
1 CCL2
2 ICAM-1
3 MIF
R Reference
Relative Protein Expression (log2)
CCL2/MCP-1
ICAM-1/CD54
MIF
IL-1B/IL-1F2
IFN-gamma
F
Lymphocytes
siNT
siJAM-A
1 CCL2
2 MIF
R Reference
Relative Protein Expression (log2)
CCL2/MCP-1
GM-CSF
MIF
Serpin E1/PAI-1

Figure 2: Astrocytic JAM-A increases pro-inflammatory protease and cytokine levels in astrocyte-CD3+ T cell co-culture. Astrocytes were transfected with JAM-A or non-targeted siRNA (*siJAM-A* vs. *siNT*, then co-cultured with CD3+ T cells for 24 hours and samples processed for human protease and cytokine ELISA immunoassays. (A-C) JAM-A knock-down in astrocytes led to an increase of MMP-1 (relative log_2 expression 0.182, p=0.019, two-tailed paired t-test) and decrease of MMP-2 (relative log_2 expression -0.329, p=0.014) in the supernatant and decrease of ADAM9 (relative log_2 expression -0.86, p=0.05) and cathepsin C (relative log_2 expression -0.96, p=0.03) in astrocyte lysates (B). There were no significant changes in protease levels seen in lymphocyte lysates (C). (D-F) Astrocytic JAM-A knock down led to decreased levels of (D) GM-CSF (relative log_2 expression -0.79, p=0.04) in the supernatant and (E) CCL-2 (relative log_2 expression -1.3, p=0.03) in astrocytic lysates. There were no significant changes in cytokine levels seen in lymphocyte lysates (F). Data (A-F) are from three biological replicates; two-tailed paired t-tests were performed on probes demonstrating a visually detectable difference in normalized expression values relative to a reference control.

For Review Only

Figure 3

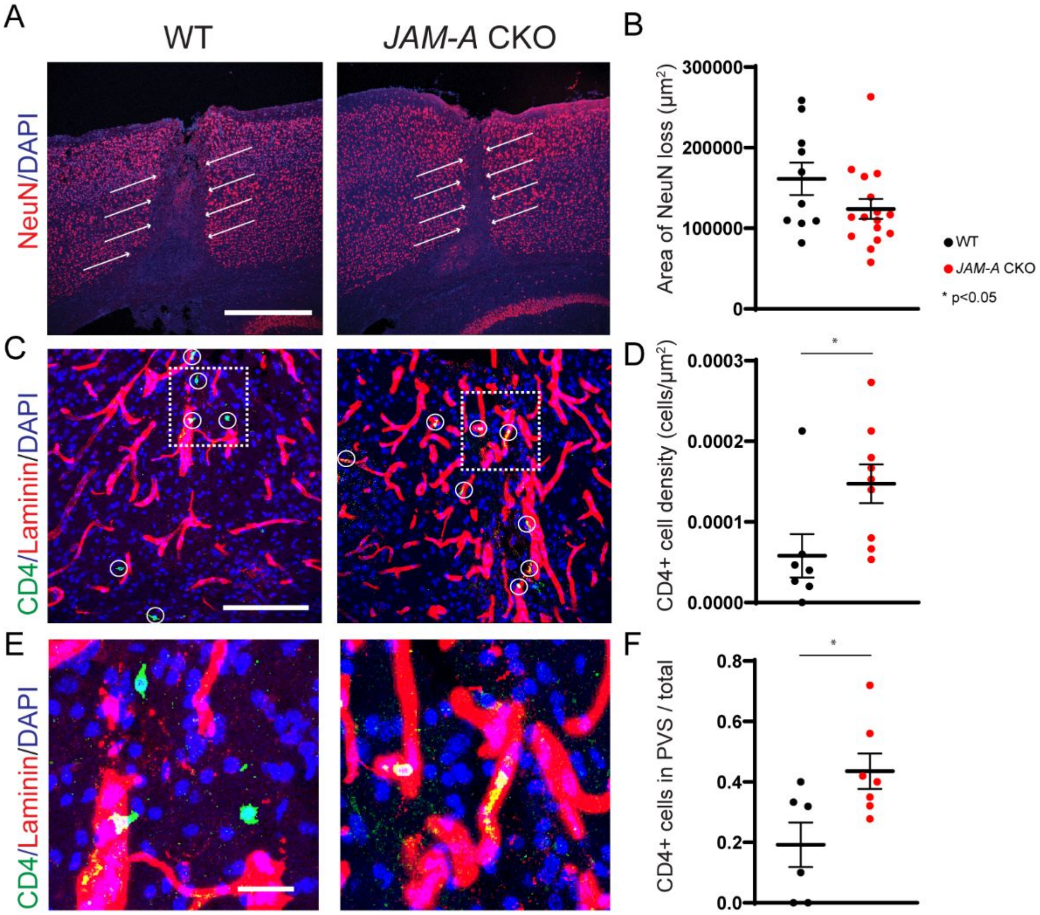
A
WT
JAM-A CKO
NeuN/DAPI
B
Area of NeuN loss (µm²)
300000
200000
100000
0
WT
JAM-A CKO
* p<0.05
C
CD4/Laminin/DAPI
D
CD4+ cell density (cells/µm²)
0.0003
0.0002
0.0001
0.0000
*
E
CD4/Laminin/DAPI
F
CD4+ cells in PVS / total
0.8
0.6
0.4
0.2
0.0
*

Figure 3: **In inflammatory cortical lesions, CD4+ T cells are arrested in the perivascular spaces in the absence of astrocytic JAM-A**. Asymptomatic inflammatory cortical lesions were induced in JAM-A CKO and WT mice with an IL-1β expressing adenovirus (ADIL-1) microinjected into the frontal cortex, with brains harvested for histopathology at 7 days post-injection. (A,B) Lesions in JAM-A CKO mice, as measured by the area of neuronal cell death (loss of NeuN, red, white arrows) showed a trend in smaller lesion size compared to WT mice that did not reach statistical significance ($p= 0.18$, CKO $n=11$, WT $n=8$, Mann Whitney test). (C, D) CD4+ cells (green and circled in white) were increased in number in JAM-A CKO lesions, scale bar: 125 µm (average number per 40x field, 22.1 (CKO) vs. 8.7 (WT), $p=0.012$, CKO $n=9$ KO, WT $n=7$, Mann-Whitney test). (E,F) In JAM-A CKO mice, a higher proportion of CD4+ cells (green) co-localize to the PVS (laminin (red)) than in WT mice (average percentage of CD4+ cells in PVS/total: 43.5 (CKO) vs. 19.2 (WT), $p=0.03$, CKO $n=8$, WT $n=6$, Mann-Whitney test; scale bar: 25µm).

For Review Only

Figure 4

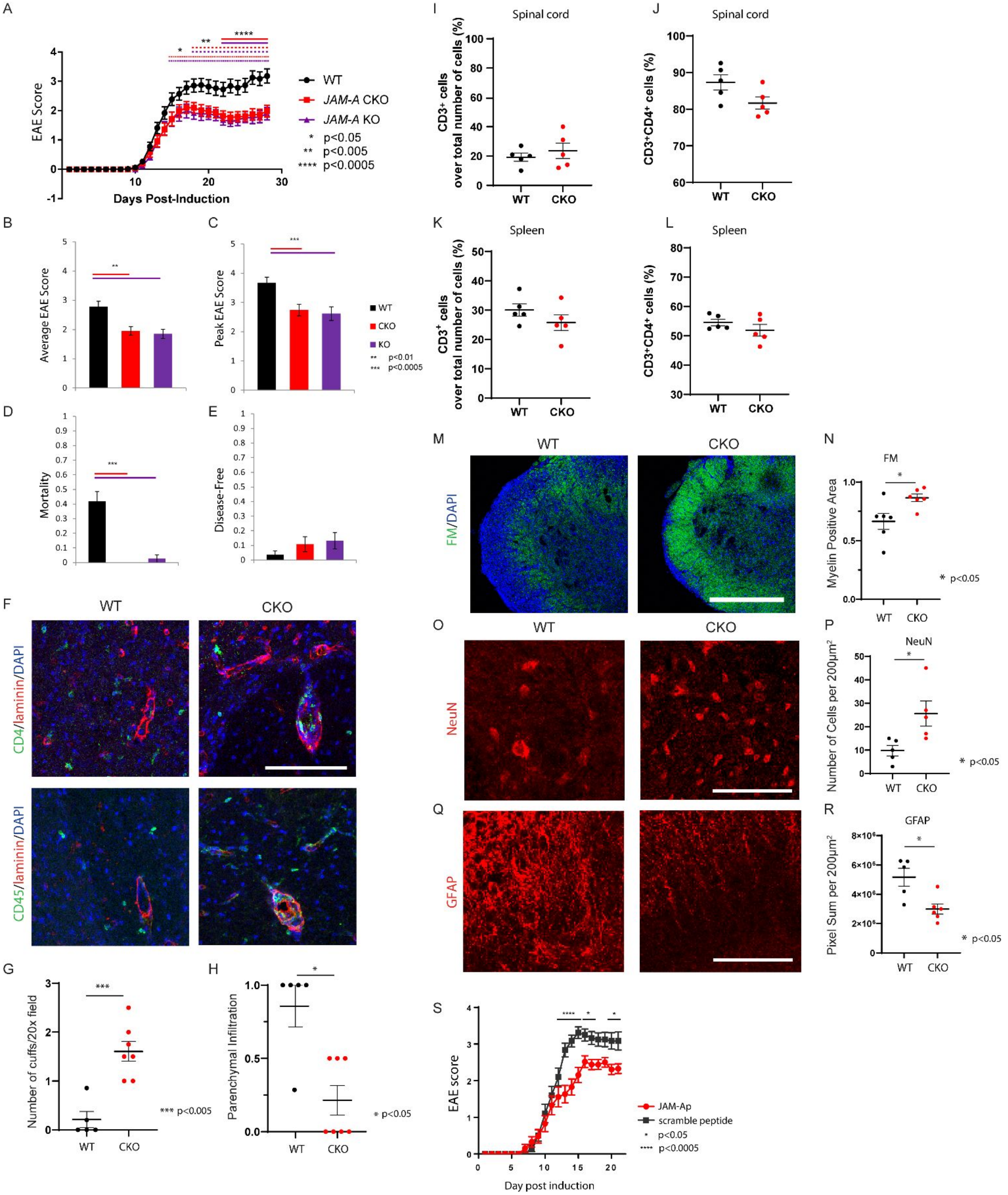
A
EAE Score
Days Post-Induction
WT
JAM-A CKO
JAM-A KO
* p<0.05
** p<0.005
**** p<0.0005
B
Average EAE Score
C
Peak EAE Score
WT
CKO
KO
** p<0.01
*** p<0.0005
D
Mortality
E
Disease-Free
F
WT
CKO
CD4/laminin/DAPI
CD45/laminin/DAPI
G
Number of cuffs/20x field
*** p<0.005
H
Parenchymal Infiltration
* p<0.05
I
Spinal cord
CD3+ cells over total number of cells (%)
J
Spinal cord
CD3+CD4+ cells (%)
K
Spleen
CD3+ cells over total number of cells (%)
L
Spleen
CD3+CD4+ cells (%)
M
FM/DAPI
N
FM
Myelin Positive Area
* p<0.05
O
NeuN
P
NeuN
Number of Cells per 200µm²
* p<0.05
Q
GFAP
R
GFAP
Pixel Sum per 200µm²
* p<0.05
S
EAE score
Day post induction
JAM-Ap
scramble peptide
* p<0.05
**** p<0.0005

Figure 4: Astrocytic JAM-A promotes clinical disability and T lymphocyte entry into the CNS parenchyma, demyelination, neuronal loss and astrocyte reactivity during EAE. (A) JAM-A CKO and KO mice show a milder course of clinical disability than WT mice with EAE; day 15-17: $p<0.05$ for WT vs CKO and WT vs. KO; day 18-21: $p<0.005$ for WT vs. CKO and WT vs. KO; day 22-25: $p<0.0005$ for WT vs. CKO and WT vs. KO, two-way ANOVA with Bonferroni correction. There was no difference between CKO and KO animals. Graph shows pooled data from 2-3 independent EAE experiments with a minimum of 8 mice per group in each experiment, total WT n=55, JAM-A CKO n=37, JAM-A KO n=38. (B-E) Average (B) and peak (C) EAE score, as well as mortality (D) was significantly lower in CKO and KO mice compared to WT (average score: 2.0 (WT) vs. 1.36 (CKO) vs. 1.28 (KO), p=0.0059; peak score: 3.6 (WT) vs. 2.7 (CKO) vs. 2.6 (KO), p=.0004, mortality: 0.41 (WT) vs. 0 (CKO) vs. 0.26 (KO), $p<0.0001$) while (E) a non-significant increased proportion of CKO and KO mice were resistant to disease induction compared with WT controls (0.014 (WT) vs 0.108 (CKO) vs. 0.131 (KO), p=0.22, Kruskal-Wallis test for all comparisons. Average in bar graphs shown with SEM. (F) Images show inflammatory lesions in the spinal cord of CKO and WT mice with EAE at day 21 post-immunization. In CKO mice, CD4+ (green, upper panel) and CD45+ cells (green, lower panel) were mostly clustered in perivascular (PVS) "cuffs," colocalizing with the pan-laminin marker (in red), whereas in WT mice, CD4+ and CD45+ cells were instead located in the parenchyma. Scale bar 100µm. (G) CD4+ cuffs quantified within a 20x field of view of thoracic spinal cord cross-sections were increased in JAM-A CKO mice compared to WTs (average 0.21 (WT) vs. 1.61 (CKO), p=0.0025, Mann Whitney test, WT n=5, CKO n=7). (H) Parenchymal infiltration of CD4+ cells were lower in JAM-A CKO compared to WT mice (average 0.86 (WT) vs. 0.21 (CKO), p=0.01, Mann Whitney test). (I-K) Flow cytometry was performed on spinal cord and

For Review Only

spleen of WT and CKO mice with EAE on day 5 from onset of disease. (I) Total CD3+ cell counts were similar in CKO and WT (mean 23% vs. 19%, p= 0.480, unpaired two-tailed t-test, n=5 WT, n=5 CKO, average EAE score WT 2.8, CKO 2.6). (J) The proportion of CD4+/CD3+ cells showed a decreasing, statistically non-significant trend in CKOs compared to WTs (mean 81.69% vs. 81.69%, p=0.07, unpaired two-tailed t-test). (K, L) In spleen, total CD3+ and CD4+/CD3+ were unchanged between CKO and WT groups (CD3+: CKO vs. WT, mean 26% vs. 30%, p=0.24; CD4+/CD3+: mean 52% vs. 54%, p=0.28; CD8+/CD3+: 42% vs 41%, p=0.53, unpaired two-tailed t-test). (M, N) Demyelination (fluoromyelin positive area of the lumbar anterolateral white matter tracts) was spared in CKO mice compared to WTs (average 0.66 (WT) vs. 0.86 (CKO), p=0.026, Mann Whitney test). Scale bar 400 µm. (O, P) CKO mice at EAE day 28 post-immunization were protected from neurodegeneration compared with time-matched WT controls, indicated by a greater neuronal survival in the lumbar spinal cord (number of NeuN+ neurons per 200μm^2) (average 9.8 (WT) vs. 25.6 (CKO), p=0.016, WT n=5, CKO n=5, Mann Whitney test). Scale bar 100 µm. (Q, R) Astrocytic activation within white matter lesions of EAE (GFAP pixel sum per 200μm^2 area of the lumbar white matter) was decreased in CKO compared to WT (average 5.2 x 10^6 (WT) vs. 2.0 x 10^6 (CKO), p=0.017, WT n=5, CKO n=6, Mann Whitney test). Scale bar 100 µm. (S) WT mice with EAE treatment with daily intraperitoneal injection of a JAM-A blocking peptide (JAM-Ap) from day 7 post-immunization were protected against clinical disability compared to scramble peptide treated controls (day 13-15, p<0.0001, day 16-17, p<0.05, day 20-21, p<0.05; graph shows pooled data from 3 independent EAE experiments with a minimum of 8 mice per group for each experiment; total JAM-Ap n=24, scramble n=26, two-way ANOVA with Bonferroni correction).

For Review Only

Supplemental Figure 1

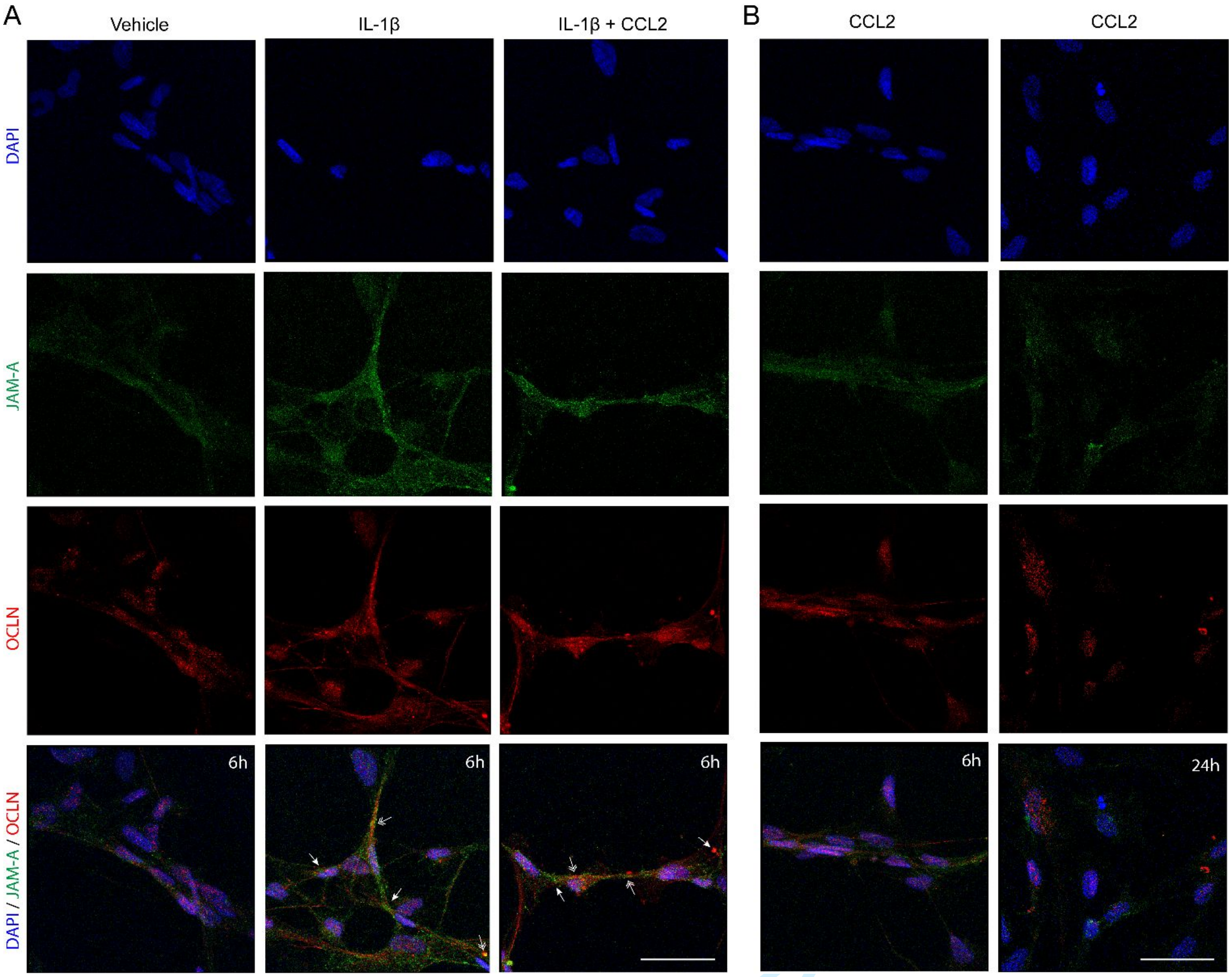

Supplemental Figure 1: Spatial and temporal dynamics of astrocytic JAM-A induction in vitro. (A) Astrocytic JAM-A (green) induction *in vitro* is detected at 6 hours after treatment with 20ng/ml IL-1β. JAM-A shows a similar pattern as seen at 24 hours in which it is diffusely localized throughout the cell membrane (white arrows) and co-localized with the tight junction marker, occludin (OCLN, red; white double headed arrows pointing to overlay of the two proteins in yellow). (B) Addition of CCL-2 did not change the distribution of JAM-A as has been previously demonstrated in CNS vascular endothelium. Scale bar 50 µm. Results were confirmed in at least 3 fields of view on two to three technical replicates in each group.

For Review Only

Supplemental Figure 2

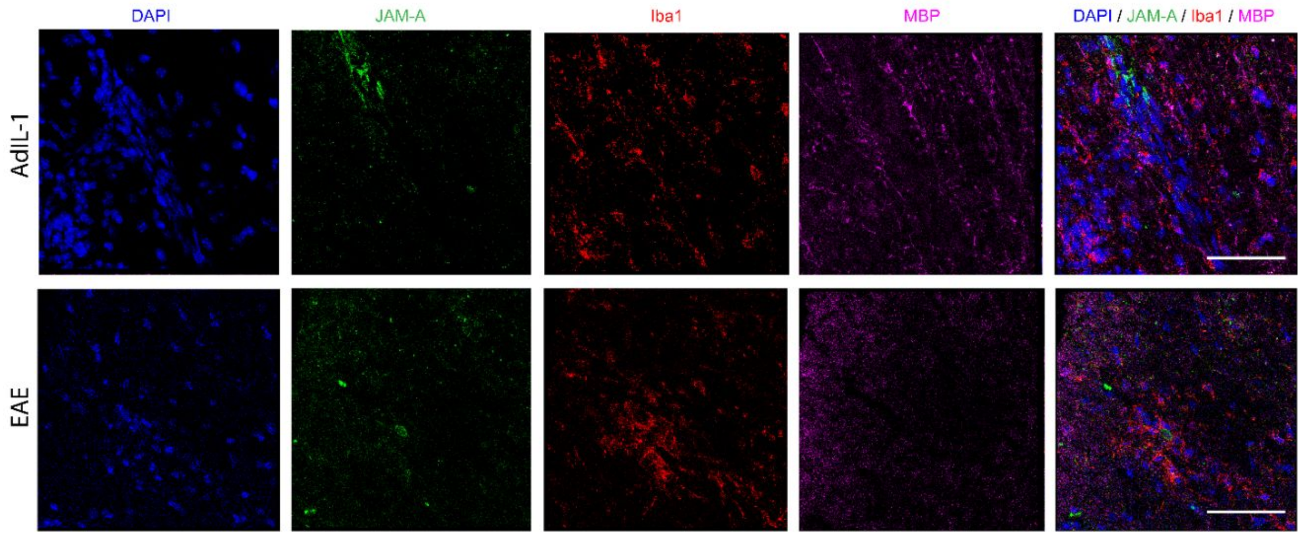

For Review Only

Supplemental Figure 2: Reactive astrocytic JAM-A is not expressed in microglia or oligodendrocytes in vivo. JAM-A expression in AdIL-1 cortical lesions and EAE spinal cord lesions does not co-localize with microglial marker Iba1 or oligodendrocyte marker MBP. Scale bar 50µm.

For Review Only
