# Supplementary material for: Astrocytic junctional adhesion molecule-A regulates T-cell entry past the glia limitans to promote central nervous system autoimmune attack
Source: Brain Commun. 2022 Feb 18;4(2):fcac044. doi: 10.1093/braincomms/fcac044 (PMC8899531; doi:10.1093/braincomms/fcac044)
Supplement: fcac044_Supplementary_Data [file fcac044_supplementary_data.zip › Revision 1.pdf]

OXFORD UNIVERSITY PRESS | Brain Communications

Astrocytic JAM-A Regulates T Cell Entry Past the Glia Limitans to Promote CNS Autoimmune Attack

Journal:	*Brain Communications*
Manuscript ID	BRAINCOM-2020-359.R1
Manuscript Type:	Original Article
Date Submitted by the Author:	31-May-2021
Complete List of Authors:	Amatruda, Mario; Icahn School of Medicine at Mount Sinai Friedman Brain Institute, Neurology<br>Chapouly, Candice; INSERM U1034,<br>Woo, Viola ; Icahn School of Medicine at Mount Sinai Friedman Brain Institute, Neurology<br>Safavi, Farinaz; National Institutes of Health, National Institute of Neurological Disorders and Stroke<br>Zhang, Joy; University of Virginia School of Medicine<br>Dai, David; University of Pennsylvania Perelman School of Medicine<br>Therattil, Anthony; New York Medical College School of Medicine<br>Moon, Chang; Icahn School of Medicine at Mount Sinai<br>Gordon, Alexandra; University of Miami Miller School of Medicine<br>Parkos, Charles; University of Michigan Michigan Medicine, Pathology<br>Horng, Sam; Icahn School of Medicine at Mount Sinai Friedman Brain Institute, Neurology
Keywords:	astrocyte, multiple sclerosis, experimental autoimmune encephalomyelitis, perivascular space, T cells

SCHOLARONE™
Manuscripts

Astrocytic JAM-A Regulates T Cell Entry Past the Glia Limitans to Promote CNS Autoimmune Attack

Mario Amatruda PhD,[1*] Candice Chapouly PhD,[~~1~~2*] ~~Mario Amatruda PhD,[2]~~ Viola Woo BS,[~~2~~ 1] Farinaz Safavi MD PhD,[3] Joy Zhang BS,[4] David Dai BA,[5] Anthony Therattil BS,[6] Chang Moon BA,[~~2~~ 1] Alexandra Gordon BA,[7] Charles Parkos MD,[8] and Sam Horng MD ~~PhD~~[~~2~~]PhD[1,9]

*Co-first authors

[1] Icahn School of Medicine at Mount Sinai, Dept of Neurology, NY, NY, USA

[~~1~~ 2] Univ. Bordeaux, Inserm, Biology of Cardiovascular Diseases, U1034, CHU de Bordeaux, F-33604 Pessac, France

~~[2] Icahn School of Medicine at Mount Sinai, Dept of Neurology, NY, NY, USA~~

[3] National Institute of Neurological Disorders and Stroke, National Institutes of Health, Bethesda, MD, USA

[4] University of Virginia School of Medicine, Charlottesville, VA, USA

[5] Perelman School of Medicine at the University of Pennsylvania, Dept of Neurology, Philadelphia, PA, USA

[6] New York Medical College, Valhalla, NY, USA

[7] Miller School of Medicine at University of Miami, Miami, FL, USA

[8] University of Michigan, Dept of Pathology, Ann Arbor, MI, USA

[9] Icahn School of Medicine at Mount Sinai, Dept of Neuroscience, NY, NY, USA

Corresponding Author:

Sam Horng MD PhD

Icahn School of Medicine at Mount Sinai

Icahn 10-20A

1468 Madison Avenue

New York, NY 10029

Email: sam.horng@mssm.edu

For Review Only

(212) 659-1692

Graphical Abstract:

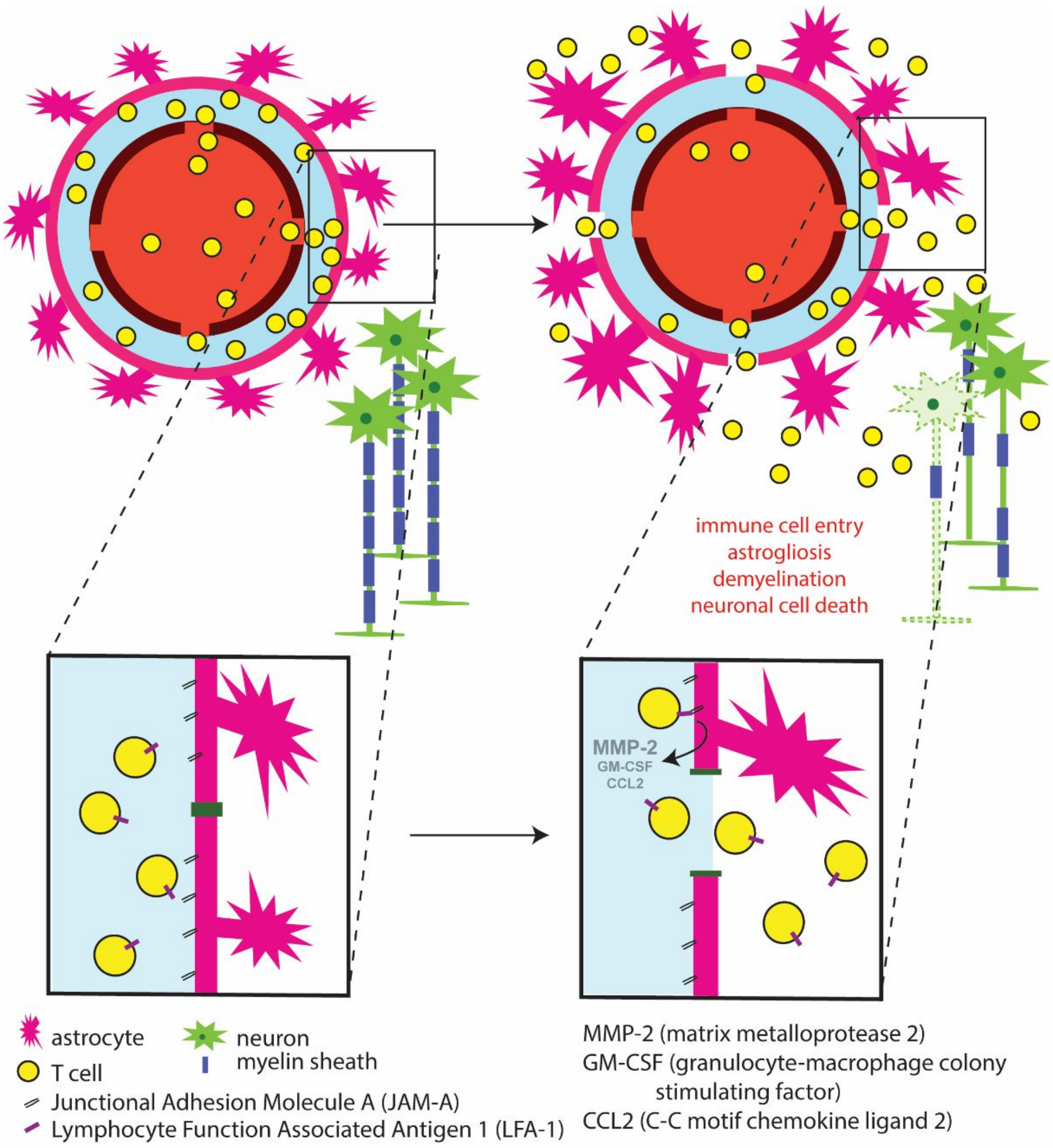

Abbreviated Summary:

Amatruda et al. report that the astrocytic immune cell receptor, Junctional Adhesion Molecule-A (JAM-A) promotes T cell entry into the CNS during autoimmune attack via the production of matrix metalloprotease 2 (MMP-2). Blocking contact-mediated astrocyte immune cell signals represents a novel therapeutic approach against multiple sclerosis and other CNS autoinflammatory diseases.

Abstract: Contact mediated interactions between the astrocytic endfeet and infiltrating immune cells within the perivascular space are underexplored, yet represent potential regulatory check-points against CNS autoimmune disease and disability. Reactive astrocytes upregulate Junctional Adhesion Molecule-A (JAM-A), an immunoglobulin-like cell surface receptor that binds to T cells via its ligand, the integrin, lymphocyte function-associated antigen-1 (LFA-1). Here, we tested the role of astrocytic JAM-A in regulating CNS autoinflammatory disease. ~~We~~ In cell co-cultures, we found that JAM-A mediated signaling between astrocytes and T cells increases levels of MMP-2, CCL-2 and GM-CSF, proinflammatory factors driving lymphocyte entry and pathogenicity in multiple sclerosis (MS) and experimental autoimmune encephalomyelitis (EAE), an animal model of CNS autoimmune disease. In EAE, ~~M~~mice with astrocyte-specific *JAM-A* deletion (*mGFAP:CreJAM-A*$^{fl/fl}$) exhibit decreased levels of MMP2, a failure of T cells to infiltrate the CNS parenchyma from the perivascular spaces (PVS), and ~~along with~~ a milder histopathological and clinical course of disease compared to wild-type controls (*JAM-A*$^{fl/fl}$). Treatment of wild-type mice with intraperitoneal injection of soluble JAM-A blocking peptide (JAM-Ap) protects against EAE, highlighting the potential of contact mediated astrocyte-immune cell signaling as a novel translational target against neuroinflammatory disease.

For Review Only

Introduction:

In multiple sclerosis (MS) and other autoimmune diseases of the central nervous system (CNS), immune cells inappropriately invade the CNS from the bloodstream and drive inflammatory lesion formation (Frischer *et al.*, 2015; Lassmann, 2018). CNS entry is a two-step process through

a specialized structure termed the neurovascular unit (NVU):~~during which~~ first, immune cells ~~first~~ cross the endothelial blood-brain barrier, using contact-mediated interactions with the endothelial surface to traffic into an intermediary compartment termed the perivascular space (PVS) (Abbott *et al.*, 2006; Engelhardt and Ransohoff, 2012; Schlager *et al.*, 2016). Within the PVS, immune cells encounter the glia limitans (GL), a- barrier comprised of astrocytic endfeet through which cells must subsequently cross to access~~reach~~ the CNS parenchyma and inflict damage (Owens *et al.*, 2008).

Interactions between the astrocytic endfeet and immune cells within the PVSs have been minimally explored despite their potential significance in regulating the autoimmune response. Cross-talk is known to involve leukocyte matrix metalloproteases (MMP-2 and MMP-9) in degrading PVS basement membranes, enabling the parenchymal entry of infiltrating immune cells during EAE (Song *et al.*, 2013; Song *et al.*, 2015; Gerwien *et al.*, 2016). Astrocytic VCAM-1 has also been identified as a TNFR1--induced cell adhesion molecule critical for immune cell trafficking past the GL and into the CNS parenchyma during EAE (Gimenez *et al.*, 2004; Gimenez *et al.*, 2006; Laureys *et al.*, 2014). Moreover, astrocytic VCAM-1 is modulated in a region-specific manner by effector T cell (Th1 and Th17) secreted factors accounting for regional differences in immune cell infiltration during EAE (Williams *et al.*, 2020). Recently, tissue resident CD8 T cells were found within multiple sclerosis lesions to express PD-1 while reactive astrocyte endfeet express PD-1 ligand, suggestive of potential inhibitory interactions within the PVS (Smolders *et al.*, 2018). Therefore, both soluble and contact-mediated signals between astrocytes and immune cells within the PVS may play a significant role in regulating CNS autoinflammatory disease.

For Review Only

We previously reported (Horng *et al.*, 2017)that reactive astrocytes upregulate Junctional Adhesion Molecule-A (JAM-A), an immunoglobulin-like cell-surface receptor, in response to the proinflammatory cytokine, interleukin-1 beta (IL-1β) *in vitro* as well as in *in vivo* models of CNS inflammation. JAM-A has a dual role: 1) initiating and stabilizing tight junction complexes via homophilic binding between identical cell types (Weber *et al.*, 2007; Luissint *et al.*, 2014; Kummer and Ebnet, 2018) and 2) serving as an immune cell surface receptor via heterophilic binding to LFA-1 on T cells and monocytes (Nourshargh *et al.*, 2006). In gut and CNS vascular endothelium, JAM-A binds to immune cells to induce intracellular signal transduction pathways and promote transmigration through the endothelial layer, ultimately leading to a pro-inflammatory, tissue damaging state (Engelhardt and Ransohoff, 2012; Lakshmi *et al.*, 2012; Schmitt *et al.*, 2014; Sladojevic *et al.*, 2014; Flemming *et al.*, 2018; Fan *et al.*, 2019; Luissint *et al.*, 2019).

We hypothesized that astrocytic JAM-A interacts with immune cells within the PVS to promote effector pathways of CNS inflammation and tissue damage. Here, we focused on the T cell population given its central role in driving pathogenesis of EAE, an animal model of CNS autoimmune demyelinating disease. We first characterized the effects of astrocytic JAM-A on protease and cytokines implicated in EAE and MS pathogenesis. Then, using a genetic mouse model in which JAM-A is selectively deleted from reactive astrocytes (*mGFAP:CreJAM-A$^{fl/fl}$*) compared to unaffected (*JAM-A$^{fl/fl}$*) controls, we investigated how astrocytic JAM-A regulates lesion pathology in two models of CNS inflammation and its effects on T cell trafficking, the inflammatory proteome and clinical disability in EAE (Cera *et al.*, 2004; Garcia *et al.*, 2004). ~~Finally~~Additionally, we tested the therapeutic potential of an exogenously administered JAM-A

For Review Only

blocking peptide in EAE.

Results:

Astrocytic JAM-A is upregulated diffusely on the astrocytic cell surface in response to interleukin-1β *in vitro* and in *in vivo* models of CNS inflammatory disease

We reported previously that reactive astrocytes upregulate the tight junction proteins, Claudin-1 (Cldn-1), Claudin-4 (Cldn-4) and JAM-A in response to CNS inflammation (Horng *et al.*, 2017). In CNS vascular and gut endothelial cells, JAM-A acts both as a tight junction molecule in trans dimeric form and as an immune cell receptor in monomeric form (Ebnet *et al.*, 2004; Wojcikiewicz *et al.*, 2009; Stamatovic *et al.*, 2012). In vascular endothelium, the cytokine CCL-2 serves as a switch, causing JAM-A internalization from the tight junction and relocalization to the cell surface as a monomer (Stamatovic *et al.*, 2012; Sladojevic *et al.*, 2014).

Using human astrocyte cultures, we confirmed that treatment with IL-1β, but not CCL2 alone, induced expression of JAM-A and the tight junction protein occludin ~~expression~~ by 6 and 24 hours (**Figure 1A,B, Supplemental Figure 1A,B**). Upon induction, astrocytic JAM-A not only co-localized with the tight junction protein occludin but was also distributed more diffusely throughout the cell membrane (**Figure ~~1A~~1C, Supplemental Figure 1C**). ~~Treatment with CCL-2 alone did not induce JAM-A expression and c~~Combined treatment with IL-1β and CCL-2 did not augment or change the ~~distribution~~ colocalization of JAM-A with the tight junction protein occludin, compared to IL-1β alone (**Figure ~~1A~~1C, Supplemental Figure 1C**). Therefore,

For Review Only

astrocytic JAM-A is found both in overlap with the tight junction and apart from it after induction by IL-1β and its distribution apart from the tight junction does not appear to change with the addition of CCL-2. Therefore, astrocytic JAM-A is expressed diffusely as a monomeric protein from the time of its upregulation by IL-1β.

We characterized the expression of astrocytic JAM-A in two models of CNS inflammation in the mouse. In resting (ie. healthy) cortex, JAM-A was not expressed in astrocytes- of the CNS parenchyma (identified using GFAP staining) (**Figure 1F**). It was most strongly detected in a pattern matching that of the vascular endothelium, consistent with previous studies (**Figure 1B, C1F**) (Padden *et al.*, 2007; Stamatovic *et al.*, 2012; Sladojevic *et al.*, 2014; Bhowmick *et al.*, 2019). In asymptomatic inflammatory lesions produced by intracortical injections of IL-1β expressing adenovirus (AdIL-1), reactive astrocytes expressed JAM-A, most prominently within the endfoot astrocytic processes encircling known to encircle blood vessels, as identified using aquaporin-4 (AQP4) staining (**Figure 1B, D1G, supplemental Figure 2A, B**). In healthy spinal cord, astrocytic JAM-A was largely undetectable (**Figure 1H, Supplemental Figure 2A,B**). In inflammatory demyelinating spinal cord lesions of EAE, astrocytic JAM-A was present expressed in astrocytes, particularly in the AQP4 positive endfeet of the perivascular astrocytes (**Figure 1H,I, Supplemental Figure 2A,B**), as well as on the surface of infiltrating leukocytes (**Figure 1E1H, Supplemental Figure 3**) and additional CNS resident cell types w.-ithin EAE lesions. JAM-A was present at low levels on blood vessels (CD31+) (**Supplemental Figure 2A, C**), robustly JAM-A was not expressed by microglial cells (Iba1+), being enriched within areas of inflammation (**Supplemental Figure 4**) and on the surface of ventral horn neurons (NeuN+) (**Supplemental Figure 5**). Oligodendrocytesor oligodendrocytes (Olig2+)(MBP) in did not show

clear overlap with JAM-A staining. AdIL-1 and EAE lesions (**Supplemental Figure 62**). . Astrocyte specific knock-down of JAM-A was demonstrated in AdIL-1 and EAE lesions, but not in healthy cortex and spinal cord, of conditional knock-out (*mGFAP:CreJAM-A$^{fl/fl}$*, CKO) mice compared to littermate wild-type (*JAM-A$^{fl/fl}$*, WT) controls (**Figure 1C-EFigure 1H-J, Supplemental Figure 2A, B**). CKO mice showed decreases of JAM-A expression within astrocytes (GFAP), including the endfeet (AQP4), but not the endothelium (CD31) during EAE (**Figure 1I, J, Supplemental Figure 2A-C**).

Astrocytic JAM-A increases levels of pro-inflammatory cytokines and proteases critical for CNS autoinflammatory disease

Local protease and cytokine levels within the PVS play a critical role in facilitating immune cell priming, CNS entry and autoimmune attack (Song *et al.*, 2015; Williams *et al.*, 2020). We tested whether astrocytic JAM-A leads to changes in protease and cytokine levels in an astrocyte-T cell co-culture system. Here, we used a pan-T cell population (CD3+) to assess the net effects of astrocytic JAM-A signaling to both CD4+ and CD8+ T cells. ELISA arrays were performed on co-cultures of activated (IL-1β treated) human astrocytes and CD3+ T cells in the presence or absence of astrocytic JAM-A. Efficacy of siRNA mediated knock-down of JAMA (*siJAM-A*) in comparison to a non-targeting siRNA (*siNT*) was demonstrated previously (Horng *et al.*, 2017). Supernatants extracted from co-cultures with *siJAM-A* transfected astrocytes showed statistically significant decreases in MMP-2 (**Figure 2A**) and GM-CSF (**Figure 2D**), both factors previously shown to promote EAE pathogenesis (dos Santos *et al.*, 2005; Agrawal *et al.*, 2006; Kroenke *et al.*, 2010; Rasouli *et al.*, 2015; Song *et al.*, 2015; Gerwien *et al.*, 2016; Levesque *et al.*, 2016; Ifergan *et al.*, 2017; Imitola *et al.*, 2018; Galli *et al.*,

For Review Only

2019; Monaghan and Wan, 2020; Wheeler *et al.*, 2020). Lysates of *siJAM-A* transfected astrocytes showed decreased levels of ADAM9, cathepsin C and CCL-2 (**Figures 2B, E**), the last of which is known to promote EAE pathogenesis via its chemotactic effects on infiltrating monocytes (Ge *et al.*, 2012; Kim *et al.*, 2014). Lysates of CD3+ T cells showed no ~~significantly~~ statistically significant protease or cytokine changes (**Figures 2C, F**). In sum, in astrocyte-T cell co-cultures subjected to pro-inflammatory conditioning of both cell types, astrocytic JAM-A ~~expression in co-culture with CD3+ T cells~~ lead~~s~~ to increased levels of MMP-2, CCL-2 and GM-CSF, EAE promoting signals involved in both immune cell infiltration into the CNS parenchyma and pathogenic T cell activity.

Astrocytic JAM-A regulates immune cell infiltration past the ~~perivascular space~~neurovascular unit (NVU) in IL-1β induced cortical lesions

To test ~~whether~~ how astrocytic JAM-A ~~facilitates~~ controls immune cell trafficking~~infiltration~~ through~~past~~ the ~~PVS~~neurovascular unit (NVU), we characterized patterns of immune cell entry in asymptomatic cortical lesions induced by intracortical AdIL-1 injection. Lesion size after AdIL-1 injection, measured as area of neuronal loss, showed a decreasing trend not reaching statistical significance in *mGFAP:CreJAM-A*$^{fl/fl}$ (CKO)~~s~~ mice compared to *JAM-A*$^{fl/fl}$ (WT)~~s~~ mice (**Figures 3A, B**). Lesions in CKO mice demonstrated on average more CD4+ immune cells than the WT group (**Figures 3C, D**). However, CD4+ immune cells in CKOs were restricted to the ~~PVS~~NVU, as demarcated by pan-laminin staining which labels the basement membranes of the NVU (**Figures 3C, E, F**). By contrast, in WT mice, the majority of CD4+ cells were located in the parenchyma, indicating successful migration out of the NVU ~~laminin-rich basement membranes of the PVS~~ (**Figures 3C, E, ~~F~~G**). Therefore, astrocytic JAM-A facilitated CD4+ immune cell infiltration past the glia limitans and

For Review Only

into the CNS parenchyma in cortical AdIL-1induced lesions.

Astrocytic JAM-A promotes EAE disease severity and tissue damage via the regulation of MMP-2 levels and T cell trafficking out of the perivascular spaces (PVS)and demyelinating neuropathology in EAE

To test the role of astrocytic JAM-A in a model of CNS autoimmune demyelinating disease, EAE was induced in *JAM-A*$^{fl/fl}$ (WT), *mGFAP:CreJAM-A*$^{fl/fl}$ (CKO), and *JAM-A*$^{-/-}$ (KO) mice. CKO and KO mice were studied to dissociate differentiate effects of astrocytic JAM-A loss from total JAM-A deletion. CKO and KO mice both showed statistically significant milder courses of disease **(Figure 4A)**, including lower average **(Figure 4B)** and, peak **(Figure 4C)** and cumulative **(Figure 4D)** disease scores at Day 28 post-immunization compared to WT mice. **(Figures 4A-C)**. Survival curves showed statistically significant differences in mortality, but not disease induction between WT and the CKO and KO mice (**Figures 4E, 4G**). Rates of mMortality- at Day 28 post-immunization wereeas significantly reduced in both CKO and KO mice (**Figure 4FD**) while rates of resistance to disease induction showed -a greater, non-statistically significant trend in CKO and KO mice compared to WTs (**Figure 4HE**). To confirm the translational potential of JAM-A blockade, WT mice with EAE were treated with daily intraperitoneal injection starting at Day 7 post-immunization of either a soluble JAM-A blocking peptide (JAM-Ap) specifically targeting the monomeric form or a scramble non-targeting peptide. Treatment with JAM-Ap demonstrated a protective effect against clinical disability in EAE compared to the scramble control (**Figure 4I**). Of note, t

The course and severity of disease did not differ between CKO and KO mice, suggesting that the

protective effect of JAM-A blockade may be fully attributed to astrocytic JAM-A. To eliminate confounding mechanisms offrom JAM-A deletion in other tissues and cell types, we decided to focus on neuropathology in the CKO line. Immunohistopathology was performed in *mGFAP:CreJAM-A$^{fl/fl}$* (CKO) and *JAM-A$^{fl/fl}$* (WT) mice to measure first patterns of immune cell infiltration into spinal cord lesions. At 5 days from EAE onset, when immune cells are most exponentially infiltrating the CNS (Barthelmes *et al.*, 2016), immune cells were found to be diffusely distributed throughout the CNS parenchyma in WT mice but accumulated within the perivascular spaces (PVS) between the astrocytic endfeet (AQP4) and blood vessel wall (CD31) in CKO mice (Figure 5A). Subsequently at At day 21 post EAE immunization, WT mice showed continued to show a typical distribution of CD4$^+$ and CD45$^+$ immune cells throughout the CNS parenchyma while CKOs showedemonstrated ongoing arresteccumulation, or cuffing, of cells within the laminin-rich perivascular spaces (PVS) and limiteddecreased infiltration of cells into the parenchyma (**Figures 45B-DF-H**). To confirm the translational potential of JAM-A blockade, WT mice were treated with daily intraperitoneal injection of either a soluble JAM-A blocking peptide (JAM-Ap) specifically targeting the monomeric form or a scramble non-targeting peptide starting at day 7 of EAE. Treatment with JAM-Ap demonstrated a protective effect against clinical disability in EAE compared to the scramble control (**Figure 4S**).

To establish a mechanistic link between astrocytic JAM-A signaling and T cell infiltration *in vivo*, a proteome profiler probing 111 soluble mouse proteins, including cytokines, chemokines, proteases, growth factors and acute phase signals was used to compare the proteomic patterns of spinal cord tissues from WT and CKO mice at 5 days from EAE disease onset. Of 111 probes, MMP-2, which had shown strong astrocytic-JAM-A dependent regulation *in vitro*, demonstrated the highest fold change *in vivo* and was the sole factor with a statistically significant difference in

For Review Only

expression between WT and CKO mice (**Figure 5E-G**).

Immunohistopathology was performed in *mGFAP:CreJAM-A*$^{f/f}$ (CKO) and *JAM-A*$^{f/f}$ (WT) to measure patterns of immune cell infiltration, demyelination, neuronal cell death and astrocytic activation. (**Figures 4F-H, M-R**). At day 21 post EAE immunization, WT mice showed a typical distribution of CD4+ and CD45+ immune cells throughout the CNS parenchyma while CKOs showed accumulation, or cuffing, of cells within the perivascular spaces (PVS) and decreased infiltration of cells into the parenchyma (**Figures 4F-H**). To test whether the absence of astrocytic JAM-A and PVS cuffing altered the total number of $CD3^+$ and $CD4^+$ T cells entering the CNS during EAE, flow cytometry was performed in CKOs and WTs at 5 days from EAE disease onset during the ascending phase of disease. No difference in total $CD3^+$ T cell number was found in the spinal cord (**Figures 4I, K5H, I**) or spleen of CKO mice compared to WTs (**Figure 45L, MI, K**). In the spinal cord, $CD3^+CD4^+$ T cell counts showed a decreasing trend not reaching statistical significance in CKOs compared to WTs (**Figure 4J5J, K**). In the spleen, $CD3^+CD4^+$ counts were not significantly different between groups (**Figure 45N, OL**). Therefore, astrocytic JAM-A deletion affecteds the spatial distribution but not total number of T cells within the CNS during EAE.

Further iImmunohistochemical analysis in healthy control (HC) spinal cord tissues, at 5 days from EAE disease onset and at 28 days post-immunization demonstratedrevealed that CKO mice were protected against subsequent neuropathological damage hallmarks of EAE. At Throughout EAEday 28 post-immunization, CKO mice maintainedshowed levels of decreased demyelination anterolateral tract flouromyelin staining that were higher than WT and comparable to HCs (**Figures 4M, N6A, B**). EAE

For Review Only

CKO mice also showed increased, numbers of neurons (NeuN positive cells) within the ventral horn of the lumbar spinal cord to HC neuronal cell death (**Figures 4O, P**) compared with time-matched (at day 5 from EAE onset and at Day 28 post immunization) WT controls and, in EAE CKO mice at Day 28 post immunization, the number of NeuN+ neurons was comparable to that observed in HC (**Figures 6C, D**)). and Finally, EAE CKO mice also had similar levels of GFAP signals astrocytic activation (**Figures 4Q, R**)in the white matter of the lumbar spinal cord compared to HC while WT mice with EAE at 28 days post immunization showed significant increases in GFAP signal, suggesting greater astrogliosis (**Figures 6E, F**). Collectively, these histopathologic changes, corresponding to reflect a milder course of the disease which resembles clinical disability and similar to other genetic models (Korner *et al.*, 1997; Song *et al.*, 2015) in which immune cell trapping within the PVSs prevents parenchymal damage and clinical disability.

To confirm the translational potential of JAM-A blockade, WT mice were treated with daily intraperitoneal injection of either a soluble JAM-A blocking peptide (JAM-Ap) specifically targeting the monomeric form or a scramble non-targeting peptide starting at day 7 of EAE. Treatment with JAM-Ap demonstrated a protective effect against clinical disability in EAE compared to the scramble control (**Figure 4S**).

Discussion:

The glia limitans (GL) is the final barrier separating peripheral infiltrating immune cells and soluble factors from the CNS parenchyma (Abbott *et al.*, 2006; Engelhardt and Coisne, 2011). The perivascular spaces (PVSs) therefore represent the penultimate compartment for incoming cells and

For Review Only

factors during CNS autoinflammatory disease. Contact-mediated signals between the astrocytic end-feet of the GL and immune cells have the potential to act as critical checkpoints for both 1) the entry of inflammatory cells into the CNS parenchyma from the PVS and 2) the functional differentiation of both cell types in the inflammatory context (De Keyser *et al.*, 2010; Sofroniew, 2015; Prajeeth *et al.*, 2017; Liddelow and Sofroniew, 2019; Williams *et al.*, 2020). Here, we demonstrate a novel role for the astrocyte~~ T cell interaction~~ cell signaling receptor, JAM-A, in~~that~~ ~~modulates~~ in controlling via MMP-2 lymphocyte trafficking into the CNS parenchyma promoting~~with~~ downstream effects of~~n~~ histopathological damage and clinical disability. Experiments using functional gene network analysis to characterize how the astrocytic JAM-A mediated interaction between astrocytes and T cells regulates the functional differentiation of both cell types are currently underway. Additional *in vivo* imaging experiments to characterize the dynamics of immune cell trafficking in the presence and absence of astrocytic JAM-A mediated signaling are also in progress.

For Review Only

JAM-A is an immunoglobulin-like cell surface receptor with well-characterized roles in tight junction formation, endothelial diapedesis and immune cell signal transduction in vascular, gut and lung endothelial cells (Weber *et al.*, 2007; Luissint *et al.*, 2014; Kummer and Ebnet, 2018; Hartmann *et al.*, 2020). We demonstrated that astrocytes upregulate JAM-A *in vitro* in response to IL-1β, a critical pro-inflammatory cytokine in multiple sclerosis and EAE pathogenesis, and *in vivo* during EAE and intracortical injection of AdIL-1. Previous work detailing JAM-A expression within active multiple sclerosis lesions, noted patterns within the blood brain barrier that appear to conform to upregulated expression within the astrocytic endfeet (Padden *et al.*, 2007).

Protease and cytokine ELISA experiments ~~confirmed~~ showed that astrocytic JAM-A increases

proinflammatory effector proteins MMP-2, CCL-2 and GM-CSF in co-culture with a CD3+ T cell population. These factors have previously been demonstrated to promote EAE pathogenesis and multiple sclerosis lesion formation; MMP-2 by facilitating immune cell migration out of the PVS and into the CNS parenchyma (Agrawal *et al.*, 2006; Song *et al.*, 2015; Gerwien *et al.*, 2016), CCL-2 through its chemotactic effects on infiltrating monocytes (Ge *et al.*, 2012; Kim *et al.*, 2014) and GM-CSF via its effects on monocyte recruitment and pathogenic T cell activity in the acute phase (Ponomarev *et al.*, 2007; Kroenke *et al.*, 2010; Kara *et al.*, 2015) with pleiotropic effects on tissue damage in the chronic phase (Duncker *et al.*, 2018).

For Review Only

Comparing conditional JAM-A knock out mice and controls, we found that astrocytic JAM-A promotes the entry of T cells into the CNS parenchyma in two *in vivo* models of CNS inflammation and that astrocytic deletion of JAM-A ~~protects against~~reduces clinical disability and histopathological damage during EAE. Astrocyte specific and total JAM-A deletion showed similar phenotypes suggesting that astrocytic JAM-A may fully account for its pathogenic effects in EAE, though this does not rule out the additional possibility of both pathogenic and protective effects of JAM-A in other tissues, including the intestinal epithelium and spleen. Exogenous administration of a soluble JAM-A blocking peptide protected against EAE, demonstrating a net protective effect and translational potential of blocking astrocyte-immune cell interactions during autoimmune attack.

Proteome ELISA arrays on spinal cord tissues at 5 days from EAE disease onset recapitulated *in vitro* findings in the EAE disease model identifying MMP-2 as a critical astrocytic JAM-A dependent factor *in vivo*. Previous work established a role for MMP-2 in promoting T cell entry into the CNS parenchyma from the perivascular spaces via several mechanisms: 1) digesting

dystroglycans that anchor the astrocytic endfeet to the parenchymal basement membrane (Agrawal *et al.*, 2006), 2) activating the proinflammatory NFκB pathway in astrocytes via Notch-1 (Song *et al.*, 2015) and 3) degrading perivascular reserves of CXCL12, which promotes the retention of immune cells within the perivascular space (McCandless *et al.*, 2006). However, no statistically significant differences were found for GM-CSF and CCL2, the two other factors identified in our *in vitro* experiments. Nonetheless, it cannot be excluded that local changes in GM-CSF and CCL2 levels within the perivascular spaces were below the threshold of detection in total spinal cord lysates and may require higher resolution techniques to establish a link *in vivo*.

The extent to which T cell activation and differentiation is influenced by local signaling interactions within the PVS has yet to be determined. Experiments measuring the relative proportions of suppressor (Treg and Th2) and proinflammatory (Th1, Th17, GM-CSF secreting) helper T cell subsets in JAM-A CKOs and WTs are now ~~in progress~~ underway to determine whether astrocytic JAM-A-mediated signaling has the capacity to modulate T cell differentiation patterns. Additional potential immunomodulatory players within the PVS include not only the astrocytic endfeet, but also pericytes, microglial processes, migrating oligodendrocyte precursors, basement membrane components and other circulating immune cells including dendritic cells, macrophages and B cells.

Astrocytes have the capacity to both promote and protect against CNS autoinflammatory disease (Cekanaviciute *et al.*, 2014; Mayo *et al.*, 2014; Anderson *et al.*, 2016; Levine *et al.*, 2016; Rothhammer *et al.*, 2016; Liddelow *et al.*, 2017; Chhatbar *et al.*, 2018; Itoh *et al.*, 2018; Tassoni *et al.*, 2019; Barbar *et al.*, 2020; Wheeler *et al.*, 2020; Williams *et al.*, 2020). In their reactive state, astrocytes drive both

For Review Only

acute and chronic phases of neuroinflammation, and contribute to the transition from a neuroinflammatory to a neurotoxic, or neurodegenerative, state (Rothhammer et al., 2016, Wheeler et al., 2020, Liddelow et al., 2017). Conversion from acute inflammatory injury to a chronic neurodegenerative state is a clinical hallmark of secondary progressive MS and also occurs in a range of other neurologic diseases, including ischemic stroke and dementia (Cekanaviciute and Buckwalter, 2016; Arranz and De Strooper, 2019; Guerrero-Garcia, 2020). Future work defining the temporal dynamics of astrocytic JAM-A signaling and other receptor-mediated astrocyte-immune cell interactions within the PVSs will help us to understand how acute neuroinflammatory changes may prime the CNS for longitudinal injury or repair, leading to novel translational strategies for progressive MS and other neurodegenerative diseases.

For Review Only

Summary:

Astrocytic JAM-A increases MMP-2, CCL-2 and GM-CSF in co-culture with T cells, and increases MMP-2 in spinal cord tissues during EAE ~~and promotes~~promoting the migration of T cells out of the perivascular spaces and into the parenchyma ~~in an animal model of CNS autoimmune disease~~, exacerbating inflammatory histopathology and clinical disability. Exogenous administration of soluble JAM-A blocking peptide protects against EAE demonstrating that blockade of contact mediated astrocyte-immune cell signaling within the perivascular space represents a novel therapeutic strategy against multiple sclerosis (MS) and other CNS autoimmune diseases.

Materials and Methods:

Cell mono-culture: astrocytes. Primary human fetal astrocytes were obtained from Lonza (CC-2565) and grown to confluence on glass confocal plates (Mat-Tek, P35GC-1.5-14C) in Astrocyte Growth Medium (AGM). AGM was comprised of MCDB 131 Medium (Gibco 10372-019) and Astrocyte BulletKit factors (Lonza, CC-3186), providing for 3% FBS, 2mM L-glutamine, 30µ~~u~~g/ml gentamicin and 15ng/ml amphotericin (GA-1000), 70uM ascorbic acid, 3ng/ml rhEGF and 7.5µ~~u~~g/ml insulin. Astrocytes were then pre-treated with MCDB 131 alone for 24 hours and then treated with 20ng/ml human recombinant interleukin-1 beta (IL-1β), 100ng/ml CCL-2, IL-1β + CCL-2 or vehicle for 6 and 24 hours and then were fixed in ice cold 4% paraformaldehyde (PFA) in~~-~~1x PBS for 30 minutes then processed for immunohistochemical staining.

Cell co-culture: astrocytes. Primary human fetal astrocytes were plated to 70% confluence on a 20 cm^2 tissue culture dish (Corning, 353003) in AGM. Astrocytes were washed with PBS twice, dissociated gently with 0.05% trypsin, centrifuged, resuspended and nucleofected with 2 µM siRNA of either non-targeting (*siNT*) or *JAM-A (siJAM-A)* targeting sequences, as detailed below. Transfected astrocytes were re-plated and allowed to grow for 24 hours in AGM. Astrocytes were then serum-starved in MCDB 131 for 24 hours and then treated with 20ng/~~ml~~ mL IL-1β for 24 hours. MCDB 131 was refreshed and astrocytes were then paired with 1-2 × 10^6 isolated $CD3^+$ T lymphocytes on a 20 cm^2 tissue culture dish for 24 hours. After co-culture, $CD3^+$ T lymphocytes, astrocytes and supernatants were separated, sonicated and stored at -20°C for protease and cytokine array experiments.

Cell co-culture: T lymphocytes. Human T lymphocytes were extracted from human blood of healthy adult donors freshly collected in lavender K2-EDTA tubes (BD #367861). Briefly, peripheral blood mononuclear cells (PBMCs) were isolated from whole blood samples using density centrifugation with Ficoll-Paque PLUS (GE Healthcare). Six milliliters of whole blood were diluted with an equal volume of HBSS (Mediatech Inc.) and layered onto 15-ml tubes prefilled with 4 ~~ml~~ mL of density gradient medium. Tubes were centrifuged for 1 hour at 620 relative centrifugal force (rcf). PBMCs were collected from their density gradient layer using a transfer pipette, washed in HBSS (Mediatech Inc.) and centrifuged for 15-20 minutes at 620 rcf x 2. The PBMC pellet was resuspended in eluent buffer and processed with a magnetic labeling and separation protocol using a human pan-T cell (CD3^{+}) (Miltenyi, 130-096-535). Cells were then activated in lymphocyte growth medium ((LGM), comprised of RPMI 1640 (Gibco), 10% FBS, 2mM L-glutamine, 1% 2-mercaptoethanol) at 37°C for 72 hours with 4µ~~u~~g/mL~~l~~ anti-CD28 (eBioscience 16-0298-85) on 20cm^2 tissue culture dishes pre-treated with 7µ~~u~~g/~~ml~~ mL anti-CD3^{+} (eBioscience 16-0037-85) in PBS at 37°C for 2 hours. After activation, T lymphocytes were centrifuged and 1-2 × 10^6 cells applied to astrocyte cultures for 24 hours before sample separation and processing, as above.

Human Protease and Cytokine Arrays. Reactive astrocyte and CD3^{+} T lymphocyte co-cultures were prepared as outlined above. Culture medium supernatant with CD3^{+} T lymphocytes was aspirated from co-cultures after 24 hours. Aspirant was centrifuged at 620 rcf for 7 minutes, then supernatant stored at 20°C. The CD3^{+} T lymphocyte pellet was reconstituted and harvested in cell lysis buffer, which was sonicated and then stored at 20°C. Adherent astrocytes from the 20 cm^2 tissue culture dish were harvested in cell lysis buffer, sonicated and then stored at 20°C.

Supernatant (500 µµl), T lymphocyte (100 µµg) and astrocyte (100 µµg) samples were then applied to human protease (R&D, ARY021B) and cytokine (R&D, ARY005B) ELISA array kits per the manufacturer's instructions in 3 biological reptriplicates. Quantification of protease signal was performed by densitometry as follows: non-saturated developed films were scanned using a Canon LiDE scanner (Canon USA), and mean pixel density of each duplicate array probe was measured using ImageJ software (NIH). Data were standardized to 3 duplicated reference probes, and the relative change between *siNT* and *siJAM-A* treated conditions was calculated and then compared to the relative change in reference probe signal. Statistical analyses were performed using unpaired two sample t-tests with unequal variance**.** Comparisons included all array probes initially and then those without visually detectable signals above background were excluded and not considered biologically significant. $\log_2$fold change was calculated.

Mouse Proteome Arrays. Spinal cord tissue from WT (n=4) and CKO (n=3) mice was harvested at 5 days from EAE disease onset, homogenized in PBS with protease inhibitors and stored at -80° C before thawing for experiments. Samples were quantified for protein concentration and 200 µg applied to mouse proteome ELISA profiler arrays (R&D, ARY028) per the manufacturer's instructions in 4 and 3 biological replicates. Quantification of protease signal and analysis of the relative change between WT and CKO was performed as outlined above in *Human Protease and Cytokine Arrays.*

Chemical and Protein Reagents. Human IL-1β and CCL-2 were purchased from PeproTech and used at 20 ng/ml mL and 10ng/mlmL, respectively, for mono-culture experiment described above.

JAM-Ap. JAM-A blocking peptide (JAM-Ap) and control peptide were synthesized to order at >95% purity from New England Biopeptide with the following sequences: NPKSTRAFSNDDYVLNPTTG for JAMA-p and NLFSVDTPNGKTASDNYPRT for control, as designed and characterized by a previous group (Sladojevic *et al.*, 2014). Daily intraperitoneal injection of 1µ~~u~~g in 0.~~1ml~~ 1mL of sterile 0.9% NaCl starting on Day 7 post EAE immunization was performed in EAE experiments.

Antibodies. Catalog numbers and concentrations of all antibodies are as follows. Anti-GFAP (130300, rat, 1:200), anti-occludin (711500, rabbit, 1:125), anti-IgG (A11029, mouse, 1:100) were from Invitrogen. Anti–JAM-A (sc53623, mouse, 1:100) was from Santa Cruz Biotechnology. Fluoromyelin was from ThermoFisher (F34651, 1:300). Anti-fibrinogen (A0080, rabbit, 1:150) was from Dako. Anti-CD3 (16-0037-85, 1:100), anti-CD4 (14-9766-82, 1:100), anti-CD31~~11b~~ (550274~~14-0112-82~~ 1:100), and anti-CD45 (550539, 1:100~~all rat~~), all rat, were from eBioscience. Anti-CD4 (ab183685, mouse, 1:50)~~ and anti-CD8 (ab217344, rabbit, 1:50)~~ was~~ere~~ from Abcam. Anti-NeuN (MAB377, mouse, 1:100) and anti–myelin basic protein (MBP) (MAB386, rat, 1:500), anti-Olig2 (MABN50, mouse, 1:500) and anti-AQP4 (AB3594, 1:200) were from Millipore. Anti-laminin (L9393, rabbit, 1:200) was from Sigma-Aldrich. Anti-Iba1 (109-19741, rabbit, 1:500) was from Wako.

siRNA. Human astrocyte cultures were nucleofected with siRNA (2 µM) with non-targeting sequences (*siNT*) or *JAM-A (siJAM-A)* targeting sequences (Thermo Scientific Dharmacon, siGENOME SMART pool), using an Amaxa nucleofector (program A033) with the Basic Glial

Kit (Amaxa). The extent and specificity of gene silencing was confirmed by immunoblotting as reported in a previous study (Horng *et al.*, 2017).

Mice. *mGfap-Cre* (B6.Cg-Tg(Gfap-cre)73.12Mvs/J) mice were genetically engineered in the laboratory of Michael Sofroniew (UCLA) and are available for purchase from Jackson laboratories (https://www.jax.org/strain/012886). *Cre* expression is astrocyte-specific except in areas of adult neurogenesis, where it is also observed in some neural progenitors (Garcia *et al.*, 2004). *JAM-A$^{f/fl}$* mice were obtained from Charles Parkos (University of Michigan, Ann Arbor, Michigan, USA) and Terence Dermody (University of Pittsburgh, Pittsburgh, Pennsylvania, USA) (Cera *et al.*, 2004; Laukoetter *et al.*, 2007). For all experiments, *mGfap-Cre JAM-A$^{fl/fl}$* female mice were crossed with *JAM-A$^{fl/fl}$* male mice to generate ~50% *mGfap-Cre JAM-A$^{fl/fl}$* (conditional knock-out (CKO) mice) and ~50% *JAM-A$^{fl/fl}$* (wild type (WT)) littermate controls. Selective deletion of JAM-A in GFAP positive cells was confirmed in a previous study (Horng *et al.*, 2017). Total JAM-A knock out (KO) mice were generated by breeding *mGfap-Cre JAM-A$^{fl/fl}$* male mice (which express Cre in germline cells) to *JAM-A$^{fl/fl}$* to create JAM-A$^{fl/-}$ mice which were then crossed to create JAM-A$^{-/-}$. Genotyping primers were: *mGfap-Cre* forward (GfF) ACC AGC CAG CTA TCA ACT C, reverse (GfR) TAT ACG CGT GCT AGC GAA GAT CTC CAT CTT CCA GCA G, 350 bp; *JAM-A* forward (JaKOF) TCT TTT CAC CAA TCG GAA CG, reverse (JF2R) AAA AAC TCT AGG AAC TCA CCC AGG A, band 200 bp (wt), 320 bp (flox); *JAM-A* excised forward (TS379) CCT CTC TTT TCA CCA ATC GGA, JAM-A excised reverse (TS512) TCT TCT TCA GAC GCC GAA CCT. PCR conditions for all primer sets were: 94°C for 4 minutes; 35 cycles of 94°C for 30 seconds, 56°C for 30 seconds, and 72°C for 30 seconds; then 72°C for 10 minutes.

For Review Only

Cortical microinjection of AdIL-1. Mice (8–12 weeks old, at least 5 per condition per time point, on the C57BL/6 background) were anesthetized using isoflurane and placed into a stereotactic frame (Kopf). AdIL-1 or AdDL70 control (AdCtrl) (10^6 PFU) was microinjected into the cerebral cortex at $y = 1$ mm caudal to bregma, $x = 2$ mm, $z = 1.5$ mm. Animals were allowed to recover for 7 days and then were sacrificed and perfused with ~~10ml~~ 10mL of 1x PBS and 10ml 4% PFA in1x PBS.

EAE. Mice (male and females, 10–13 weeks old, at least 8 animals per group for each experiment, on the C57BL/6 background) were subcutaneously injected at cervical and lumbar sites ~~with~~ 0.1 cc of $MOG_{35–55}$ in complete Freund's adjuvant (CFA) (1 mg/mL) followed by intraperitoneal injection of 0.1 cc pertussis toxin on day 0 and day 1 (Hooke Laboratories) ~~at cervical and lumbar sites followed by intraperitoneal injection of 0.1 cc pertussis toxin on day 0 and day 1~~. Healthy control (HC) mice received the injection of CFA emulsion with no MOG_{35-55}. Mice were rated daily on a standard 5-point motor scale from days 7–~~21~~ 28 after induction: 0, no symptoms; 1, floppy tail; 2, hind limb weakness (paraparesis); 3, hind limb paralysis (paraplegia); 4, forelimb and hind limb paralysis; 5, death. The average EAE score consisted of the average score across all animals of the same genotype at Day 28 (the end of the experiment). Cumulative EAE score consisted of the average summed score per animal across all animals of the same genotype at Day 28 (the end of the experiment). Average mortality was calculated as the proportion of animals with a score of 5 by the end of the experiment (Day 28). Disease free values were calculated by the proportion of animals who maintained a score of 0 throughout the entire experiment (Day 7-28). Survival curves for mortality and disease induction were performed and

analyzed with a Mantel-Cox test. Three independent experiments were performed for WT and CKO and two independent experiments for KO. Raters were blind to genotype where possible; breeding conditions required separate WT and KO cages in parallel to the CKO colony, which produced both WT and CKO offspring. Three independent experiments were performed for JAM-Ap and scramble control experiments. Raters were blinded to treatment group in the treatment experiments.

Flow cytometry. WT and CKO mice were anesthetized and perfused with 5 ~~ml~~ mL PBS at 4-5 days from onset of disease in EAE (days 14-20 from induction). Spinal cords and spleens were collected in cold PBS and mechanically dissociated. Spleen samples were passed through a 70-µm filter, then incubated in red blood cell (RBC) lysis buffer (BioLegend) for 2 minutes at room temperature and washed with PBS. Spinal cords were passed through a 100-µm filter and separated from myelin using a 60%/30% Percoll gradient. Cell suspensions were then collected, counted, and subjected first to a Zombie Yellow stain (Biolegend, 423103) and wash followed by incubation with FITC-anti-CD3 (Biolegend, 100306) and APC/Cy7-anti-CD4 (Biolegend, 100355) antibodies in cell staining buffer (BioLegend). Cells were washed with FACS buffer (2% FBS in PBS), fixed and permeabilized following manufacturer's instructions for staining using the FIX & PERM® Cell Permeabilization Kit (Invitrogen). Forward scatter and side scatter were used to gate cells excluding debris and cell aggregates, Zombie Yellow was used to exclude dead cells and then percentages of CD3 positive cells were measured with a subsequent gate to CD4. Flow cytometry for spinal cord tissues was set to run CD3+ events up to 2000; total CD3+ counts from actual samples ranged from 298 to 1340. Flow cytometry for spleen tissues all exceeded CD3+ counts of 40,000. Data was acquired on the Invitrogen™ Attune™ NxT Flow Cytometer

and analyzed with FCS Express software (De Novo) at the Flow Cytometry CoRE at Mount Sinai.

Immunohistochemistry. Brains and spinal cords were dissected from animals perfused with 10ml 10mL ice cold 1x PBS followed by 10ml 4% PFA-1x PBS, tissues were subjected to 2 hours post-fixation in 4% PFA-1xPBS followed by storage in 30% sucrose-1x PBS at 4 degrees until sectioning. Immunostaining was performed on 25um coronal (brain) and axial (spinal cord) sections. For all antibody staining, sections underwent antigen retrieval in citrate (pH 6.0; 100°C) for 20 minutes. For laminin, CD4 and CD45, sections were treated with 0.5 mg/ml mL protease XIV (Sigma-Aldrich) at 37°C for 5 minutes. Primary antibodies were used at concentrations ranging from 1:50-1:500. Samples were examined using a Leica Microsystems confocal microscope, and stacks were collected with *z* of 1 µm.

Morphometric analysis. Morphometric analyses were performed using NIH ImageJ and Leica LAS softwares and all analyses were performed blinded to treatment group and genotype. For studies *in vitro*, JAM-A and occludin histochemical stains were analyzed in projections from astrocyte cultures. Colocalization analysis was performed using the ImageJ Just Another Colocalization Plugin (JACoP). For studies *in vivo*, JAM-A, occludin, pan-laminin, Olig2, Iba1, CD4, CD45, fluoromyelin (FM), NeuN and GFAP histochemical stains were analyzed in projections from cortical and spinal cord sections at the lumbar level. Cortical AdIL-1 injection lesions were analyzed on coronal brain sections of 25 µm thickness distributed serially across 10 slides from the posterior to anterior end of the brain; adjacent sections on the same slide were roughly 250 µm apart. Sections for analysis were selected to represent the center of the lesion,

corresponding to the area of greatest lesion length as noted by width of NeuN loss and GFAP positivity. Field of analysis for lymphocyte localization relative to the PVS was selected on a 40x field of approximately 385 μm width centered over the midline of the cortical injection site. EAE spinal cord lesions were analyzed on axial spinal cord sections of 25 μm thickness distributed serially across 10 slides from the caudal to rostral end of the lumbar, thoracic and cervical spinal cord; adjacent sections on the same slide were roughly 250 μm apart. Sections for analysis focused on the lumbar white matter (anterolateral tract) and gray matter (dorsal horn). ~~The same number of~~At least three representative images were~~as~~ quantified and averaged from 3-~~5~~ 6 age- and sex-matched animals per condition per genotype per time point~~ and 3-5 age- and sex-matched normal controls unless otherwise indicated, and at least 4 random ×10 to ×20 fields in 3-5 sections per animal~~. Myelin loss, neuronal loss and astrocyte reactivity were quantified by measuring the FM positive area, counting NeuN$^+$ cells and measuring GFAP positive pixel sums~~s~~ ~~from the~~normalized to total area in each 200 x 200 μm^2 field of interest in matched projections at ×20 magnification.

Statistics. Results are reported as mean ± SEM. Student's *t* test and Mann-Whitney U tests were used to compare two groups of unmatched samples. One way ANOVA and Kruskal-Wallis H test were~~as~~ used to compare more than two groups with multiple comparisons. For multiple comparisons ~~in~~ of the EAE disease course, 2-way ANOVA followed by Bonferroni post-test correction was used. Non-parametric analyses of EAE scores were also performed using one-way ANOVA (Friedman) with Dunn's multiple comparisons correction and Kolmogorov-Smirnov test comparing cumulative distributions. In all cases, p less than 0.05 was considered significant.

Study approval. Use of commercially available human astrocytes and anonymized human blood donor samples was approved by the IRB at the Icahn School of Medicine at Mount Sinai (ISMMS). Studies using mice were approved by the IACUC at the ISMMS, and adhered to the American Veterinary Medical Association guidelines. The ISMMS has an Animal Welfare Assurance on file with the Office for Laboratory Animal Welfare (Assurance no. A3111-01).

Data Availability Statement:

The data that support the findings of this study are available from the corresponding author, upon reasonable request.

Competing Interests:

The authors have no conflicts of interest to disclose.

CRediT Author contributions:

Mario Amatruda: Conceptualization, Methodology, Visualization, Validation, Formal analysis, Investigation, Writing – Review and Editing, ~~Visualization,~~ Project administration

Candice Chapouly: Conceptualization, Methodology, Validation, Formal analysis, Investigation, Writing – Review and Editing, Visualization, Project administration

~~Mario Amatruda: Conceptualization, Methodology, Visualization, Validation, Formal analysis, Investigation, Writing – Review and Editing, Visualization, Project administration~~

For Review Only

Viola Woo: Methodology, Validation, Formal analysis

Farinaz Safavi: Methodology, Formal analysis

Joy Zhang: Methodology, Investigation, Validation, Formal analysis

David Dai: Methodology, Investigation, Validation, Formal analysis

Anthony Therattil: Methodology, Investigation

Chang Moon: Investigation

Alexandra Gordon: Investigation

Charles Parkos: Resources

Sam Horng: Conceptualization, Methodology, Validation, Formal analysis, Investigation, Writing – Original Draft, Review and Editing, Visualization, Supervision, Project administration, Funding acquisition

For Review Only

Acknowledgments:

The authors thank Dr. Anne Schaefer, Dr. Patrizia Casaccia, Dr. Fred Lublin and Dr. Stuart Sealfon for their helpful discussions on this project and the manuscript.

Funding:

This work was supported by grant funding to Dr. Horng: National Institutes of Health (NIH) National Institute of Neurological Diseases and Stroke (NINDS) K08 NS102507-01A1, NIH R25NS079102, a Career Transition Award by the National Multiple Sclerosis Society and the Conrad N. Hilton Foundation, philanthropic support by the Jayne and Harvey Beker Foundation and a post-doctoral

Neuroscience fellowship from the Leon Levy Foundation. Dr. Safavi was supported by a post-doctoral Neuroscience fellowship from the Leon Levy Foundation.

The MSSM Microscopy and Flow Cytometry Shared Resource Facilities were utilized in this study; core facilities receive support from National Institutes of Health/National Cancer Institute Grant R24 CA095823.

For Review Only

References:

Abbott NJ, Ronnback L, Hansson E. Astrocyte-endothelial interactions at the blood-brain barrier. Nat Rev Neurosci 2006; 7(1): 41-53.
Agrawal S, Anderson P, Durbeej M, van Rooijen N, Ivars F, Opdenakker G*, et al.* Dystroglycan is selectively cleaved at the parenchymal basement membrane at sites of leukocyte extravasation in experimental autoimmune encephalomyelitis. J Exp Med 2006; 203(4): 1007-19.
Anderson MA, Burda JE, Ren Y, Ao Y, O'Shea TM, Kawaguchi R*, et al.* Astrocyte scar formation aids central nervous system axon regeneration. Nature 2016; 532(7598): 195-200.
Arranz AM, De Strooper B. The role of astroglia in Alzheimer's disease: pathophysiology and clinical implications. Lancet Neurol 2019; 18(4): 406-14.
Barbar L, Jain T, Zimmer M, Kruglikov I, Sadick JS, Wang M*, et al.* CD49f Is a Novel Marker of Functional and Reactive Human iPSC-Derived Astrocytes. Neuron 2020; 107(3): 436-53 e12.
Barthelmes J, Tafferner N, Kurz J, de Bruin N, Parnham MJ, Geisslinger G*, et al.* Induction of Experimental Autoimmune Encephalomyelitis in Mice and Evaluation of the Disease-dependent Distribution of Immune Cells in Various Tissues. J Vis Exp 2016(111).
Bhowmick S, D'Mello V, Caruso D, Wallerstein A, Abdul-Muneer PM. Impairment of pericyte-endothelium crosstalk leads to blood-brain barrier dysfunction following traumatic brain injury. Exp Neurol 2019; 317: 260-70.
Cekanaviciute E, Buckwalter MS. Astrocytes: Integrative Regulators of Neuroinflammation in Stroke and Other Neurological Diseases. Neurotherapeutics 2016; 13(4): 685-701.
Cekanaviciute E, Fathali N, Doyle KP, Williams AM, Han J, Buckwalter MS. Astrocytic transforming growth factor-beta signaling reduces subacute neuroinflammation after stroke in mice. Glia 2014; 62(8): 1227-40.
Cera MR, Del Prete A, Vecchi A, Corada M, Martin-Padura I, Motoike T*, et al.* Increased DC trafficking to lymph nodes and contact hypersensitivity in junctional adhesion molecule-A-deficient mice. J Clin Invest 2004; 114(5): 729-38.
Chhatbar C, Detje CN, Grabski E, Borst K, Spanier J, Ghita L*, et al.* Type I Interferon Receptor Signaling of Neurons and Astrocytes Regulates Microglia Activation during Viral Encephalitis. Cell Rep 2018; 25(1): 118-29 e4.
De Keyser J, Laureys G, Demol F, Wilczak N, Mostert J, Clinckers R. Astrocytes as potential targets to suppress inflammatory demyelinating lesions in multiple sclerosis. Neurochem Int 2010; 57(4): 446-50.
dos Santos AC, Barsante MM, Arantes RM, Bernard CC, Teixeira MM, Carvalho-Tavares J. CCL2 and CCL5 mediate leukocyte adhesion in experimental autoimmune encephalomyelitis--an intravital microscopy study. J Neuroimmunol 2005; 162(1-2): 122-9.
Duncker PC, Stoolman JS, Huber AK, Segal BM. GM-CSF Promotes Chronic Disability in Experimental Autoimmune Encephalomyelitis by Altering the Composition of Central Nervous System-Infiltrating Cells, but Is Dispensable for Disease Induction. J Immunol 2018; 200(3): 966-73.
Ebnet K, Suzuki A, Ohno S, Vestweber D. Junctional adhesion molecules (JAMs): more molecules with dual functions? J Cell Sci 2004; 117(Pt 1): 19-29.

For Review Only

Engelhardt B, Coisne C. Fluids and barriers of the CNS establish immune privilege by confining immune surveillance to a two-walled castle moat surrounding the CNS castle. Fluids Barriers CNS 2011; 8(1): 4.
Engelhardt B, Ransohoff RM. Capture, crawl, cross: the T cell code to breach the blood-brain barriers. Trends Immunol 2012; 33(12): 579-89.
Fan S, Weight CM, Luissint AC, Hilgarth RS, Brazil JC, Ettel M, *et al.* Role of JAM-A tyrosine phosphorylation in epithelial barrier dysfunction during intestinal inflammation. Mol Biol Cell 2019; 30(5): 566-78.
Flemming S, Luissint AC, Nusrat A, Parkos CA. Analysis of leukocyte transepithelial migration using an in vivo murine colonic loop model. JCI Insight 2018; 3(20).
Frischer JM, Weigand SD, Guo Y, Kale N, Parisi JE, Pirko I, *et al.* Clinical and pathological insights into the dynamic nature of the white matter multiple sclerosis plaque. Ann Neurol 2015; 78(5): 710-21.
Galli E, Hartmann FJ, Schreiner B, Ingelfinger F, Arvaniti E, Diebold M, *et al.* GM-CSF and CXCR4 define a T helper cell signature in multiple sclerosis. Nat Med 2019; 25(8): 1290-300.
Garcia AD, Doan NB, Imura T, Bush TG, Sofroniew MV. GFAP-expressing progenitors are the principal source of constitutive neurogenesis in adult mouse forebrain. Nat Neurosci 2004; 7(11): 1233-41.
Ge S, Shrestha B, Paul D, Keating C, Cone R, Guglielmotti A, *et al.* The CCL2 synthesis inhibitor bindarit targets cells of the neurovascular unit, and suppresses experimental autoimmune encephalomyelitis. J Neuroinflammation 2012; 9: 171.
Gerwien H, Hermann S, Zhang X, Korpos E, Song J, Kopka K, *et al.* Imaging matrix metalloproteinase activity in multiple sclerosis as a specific marker of leukocyte penetration of the blood-brain barrier. Sci Transl Med 2016; 8(364): 364ra152.
Gimenez MA, Sim J, Archambault AS, Klein RS, Russell JH. A tumor necrosis factor receptor 1-dependent conversation between central nervous system-specific T cells and the central nervous system is required for inflammatory infiltration of the spinal cord. Am J Pathol 2006; 168(4): 1200-9.
Gimenez MA, Sim JE, Russell JH. TNFR1-dependent VCAM-1 expression by astrocytes exposes the CNS to destructive inflammation. J Neuroimmunol 2004; 151(1-2): 116-25.
Guerrero-Garcia JJ. The role of astrocytes in multiple sclerosis pathogenesis. Neurologia 2020; 35(6): 400-8.
Hartmann C, Schwietzer YA, Otani T, Furuse M, Ebnet K. Physiological functions of junctional adhesion molecules (JAMs) in tight junctions. Biochim Biophys Acta Biomembr 2020; 1862(9): 183299.
Horng S, Therattil A, Moyon S, Gordon A, Kim K, Argaw AT, *et al.* Astrocytic tight junctions control inflammatory CNS lesion pathogenesis. J Clin Invest 2017; 127(8): 3136-51.
Ifergan I, Davidson TS, Kebir H, Xu D, Palacios-Macapagal D, Cann J, *et al.* Targeting the GM-CSF receptor for the treatment of CNS autoimmunity. J Autoimmun 2017; 84: 1-11.
Imitola J, Rasouli J, Watanabe F, Mahajan K, Sharan AD, Ciric B, *et al.* Elevated expression of granulocyte-macrophage colony-stimulating factor receptor in multiple sclerosis lesions. J Neuroimmunol 2018; 317: 45-54.
Itoh N, Itoh Y, Tassoni A, Ren E, Kaito M, Ohno A, *et al.* Cell-specific and region-specific transcriptomics in the multiple sclerosis model: Focus on astrocytes. Proc Natl Acad Sci U S A 2018; 115(2): E302-E9.

For Review Only

Kara EE, McKenzie DR, Bastow CR, Gregor CE, Fenix KA, Ogunniyi AD*, et al.* CCR2 defines in vivo development and homing of IL-23-driven GM-CSF-producing Th17 cells. Nat Commun 2015; 6: 8644.
Kim RY, Hoffman AS, Itoh N, Ao Y, Spence R, Sofroniew MV*, et al.* Astrocyte CCL2 sustains immune cell infiltration in chronic experimental autoimmune encephalomyelitis. J Neuroimmunol 2014; 274(1-2): 53-61.
Korner H, Riminton DS, Strickland DH, Lemckert FA, Pollard JD, Sedgwick JD. Critical points of tumor necrosis factor action in central nervous system autoimmune inflammation defined by gene targeting. J Exp Med 1997; 186(9): 1585-90.
Kroenke MA, Chensue SW, Segal BM. EAE mediated by a non-IFN-gamma/non-IL-17 pathway. Eur J Immunol 2010; 40(8): 2340-8.
Kummer D, Ebnet K. Junctional Adhesion Molecules (JAMs): The JAM-Integrin Connection. Cells 2018; 7(4).
Lakshmi SP, Reddy AT, Naik MU, Naik UP, Reddy RC. Effects of JAM-A deficiency or blocking antibodies on neutrophil migration and lung injury in a murine model of ALI. Am J Physiol Lung Cell Mol Physiol 2012; 303(9): L758-66.
Lassmann H. Pathogenic Mechanisms Associated With Different Clinical Courses of Multiple Sclerosis. Front Immunol 2018; 9: 3116.
Laukoetter MG, Nava P, Lee WY, Severson EA, Capaldo CT, Babbin BA*, et al.* JAM-A regulates permeability and inflammation in the intestine in vivo. J Exp Med 2007; 204(13): 3067-76.
Laureys G, Gerlo S, Spooren A, Demol F, De Keyser J, Aerts JL. beta(2)-adrenergic agonists modulate TNF-alpha induced astrocytic inflammatory gene expression and brain inflammatory cell populations. J Neuroinflammation 2014; 11: 21.
Levesque SA, Pare A, Mailhot B, Bellver-Landete V, Kebir H, Lecuyer MA*, et al.* Myeloid cell transmigration across the CNS vasculature triggers IL-1beta-driven neuroinflammation during autoimmune encephalomyelitis in mice. J Exp Med 2016; 213(6): 929-49.
Levine J, Kwon E, Paez P, Yan W, Czerwieniec G, Loo JA*, et al.* Traumatically injured astrocytes release a proteomic signature modulated by STAT3-dependent cell survival. Glia 2016; 64(5): 668-94.
Liddelow SA, Guttenplan KA, Clarke LE, Bennett FC, Bohlen CJ, Schirmer L*, et al.* Neurotoxic reactive astrocytes are induced by activated microglia. Nature 2017; 541(7638): 481-7.
Liddelow SA, Sofroniew MV. Astrocytes usurp neurons as a disease focus. Nat Neurosci 2019; 22(4): 512-3.
Luissint AC, Nusrat A, Parkos CA. JAM-related proteins in mucosal homeostasis and inflammation. Semin Immunopathol 2014; 36(2): 211-26.
Luissint AC, Williams HC, Kim W, Flemming S, Azcutia V, Hilgarth RS*, et al.* Macrophage-dependent neutrophil recruitment is impaired under conditions of increased intestinal permeability in JAM-A-deficient mice. Mucosal Immunol 2019; 12(3): 668-78.
Mayo L, Trauger SA, Blain M, Nadeau M, Patel B, Alvarez JI*, et al.* Regulation of astrocyte activation by glycolipids drives chronic CNS inflammation. Nat Med 2014; 20(10): 1147-56.
McCandless EE, Wang Q, Woerner BM, Harper JM, Klein RS. CXCL12 limits inflammation by localizing mononuclear infiltrates to the perivascular space during experimental autoimmune encephalomyelitis. J Immunol 2006; 177(11): 8053-64.
Monaghan KL, Wan ECK. The Role of Granulocyte-Macrophage Colony-Stimulating Factor in Murine Models of Multiple Sclerosis. Cells 2020; 9(3).

For Review Only

Nourshargh S, Krombach F, Dejana E. The role of JAM-A and PECAM-1 in modulating leukocyte infiltration in inflamed and ischemic tissues. J Leukoc Biol 2006; 80(4): 714-8.
Owens T, Bechmann I, Engelhardt B. Perivascular spaces and the two steps to neuroinflammation. Journal of neuropathology and experimental neurology 2008; 67(12): 1113-21.
Padden M, Leech S, Craig B, Kirk J, Brankin B, McQuaid S. Differences in expression of junctional adhesion molecule-A and beta-catenin in multiple sclerosis brain tissue: increasing evidence for the role of tight junction pathology. Acta Neuropathol 2007; 113(2): 177-86.
Ponomarev ED, Shriver LP, Maresz K, Pedras-Vasconcelos J, Verthelyi D, Dittel BN. GM-CSF production by autoreactive T cells is required for the activation of microglial cells and the onset of experimental autoimmune encephalomyelitis. J Immunol 2007; 178(1): 39-48.
Prajeeth CK, Kronisch J, Khorooshi R, Knier B, Toft-Hansen H, Gudi V*, et al.* Effectors of Th1 and Th17 cells act on astrocytes and augment their neuroinflammatory properties. J Neuroinflammation 2017; 14(1): 204.
Rasouli J, Ciric B, Imitola J, Gonnella P, Hwang D, Mahajan K*, et al.* Expression of GM-CSF in T Cells Is Increased in Multiple Sclerosis and Suppressed by IFN-beta Therapy. J Immunol 2015; 194(11): 5085-93.
Rothhammer V, Mascanfroni ID, Bunse L, Takenaka MC, Kenison JE, Mayo L*, et al.* Type I interferons and microbial metabolites of tryptophan modulate astrocyte activity and central nervous system inflammation via the aryl hydrocarbon receptor. Nat Med 2016; 22(6): 586-97.
Schlager C, Korner H, Krueger M, Vidoli S, Haberl M, Mielke D*, et al.* Effector T-cell trafficking between the leptomeninges and the cerebrospinal fluid. Nature 2016; 530(7590): 349-53.
Schmitt MM, Fraemohs L, Hackeng TM, Weber C, Koenen RR. Atherogenic mononuclear cell recruitment is facilitated by oxidized lipoprotein-induced endothelial junctional adhesion molecule-A redistribution. Atherosclerosis 2014; 234(2): 254-64.
Sladojevic N, Stamatovic SM, Keep RF, Grailer JJ, Sarma JV, Ward PA*, et al.* Inhibition of junctional adhesion molecule-A/LFA interaction attenuates leukocyte trafficking and inflammation in brain ischemia/reperfusion injury. Neurobiol Dis 2014; 67: 57-70.
Smolders J, Heutinck KM, Fransen NL, Remmerswaal EBM, Hombrink P, Ten Berge IJM*, et al.* Tissue-resident memory T cells populate the human brain. Nat Commun 2018; 9(1): 4593.
Sofroniew MV. Astrocyte barriers to neurotoxic inflammation. Nat Rev Neurosci 2015; 16(5): 249-63.
Song J, Wu C, Korpos E, Zhang X, Agrawal SM, Wang Y*, et al.* Focal MMP-2 and MMP-9 activity at the blood-brain barrier promotes chemokine-induced leukocyte migration. Cell Rep 2015; 10(7): 1040-54.
Song J, Wu C, Zhang X, Sorokin LM. In vivo processing of CXCL5 (LIX) by matrix metalloproteinase (MMP)-2 and MMP-9 promotes early neutrophil recruitment in IL-1beta-induced peritonitis. J Immunol 2013; 190(1): 401-10.
Stamatovic SM, Sladojevic N, Keep RF, Andjelkovic AV. Relocalization of junctional adhesion molecule A during inflammatory stimulation of brain endothelial cells. Mol Cell Biol 2012; 32(17): 3414-27.
Tassoni A, Farkhondeh V, Itoh Y, Itoh N, Sofroniew MV, Voskuhl RR. The astrocyte transcriptome in EAE optic neuritis shows complement activation and reveals a sex difference in astrocytic C3 expression. Sci Rep 2019; 9(1): 10010.
Weber C, Fraemohs L, Dejana E. The role of junctional adhesion molecules in vascular inflammation. Nat Rev Immunol 2007; 7(6): 467-77.

Wheeler MA, Clark IC, Tjon EC, Li Z, Zandee SEJ, Couturier CP*, et al.* MAFG-driven astrocytes promote CNS inflammation. Nature 2020; 578(7796): 593-9.
Williams JL, Manivasagam S, Smith BC, Sim J, Vollmer LL, Daniels BP*, et al.* Astrocyte-T cell crosstalk regulates region-specific neuroinflammation. Glia 2020; 68(7): 1361-74.
Wojcikiewicz EP, Koenen RR, Fraemohs L, Minkiewicz J, Azad H, Weber C*, et al.* LFA-1 binding destabilizes the JAM-A homophilic interaction during leukocyte transmigration. Biophys J 2009; 96(1): 285-93.

For Review Only

Figure 1

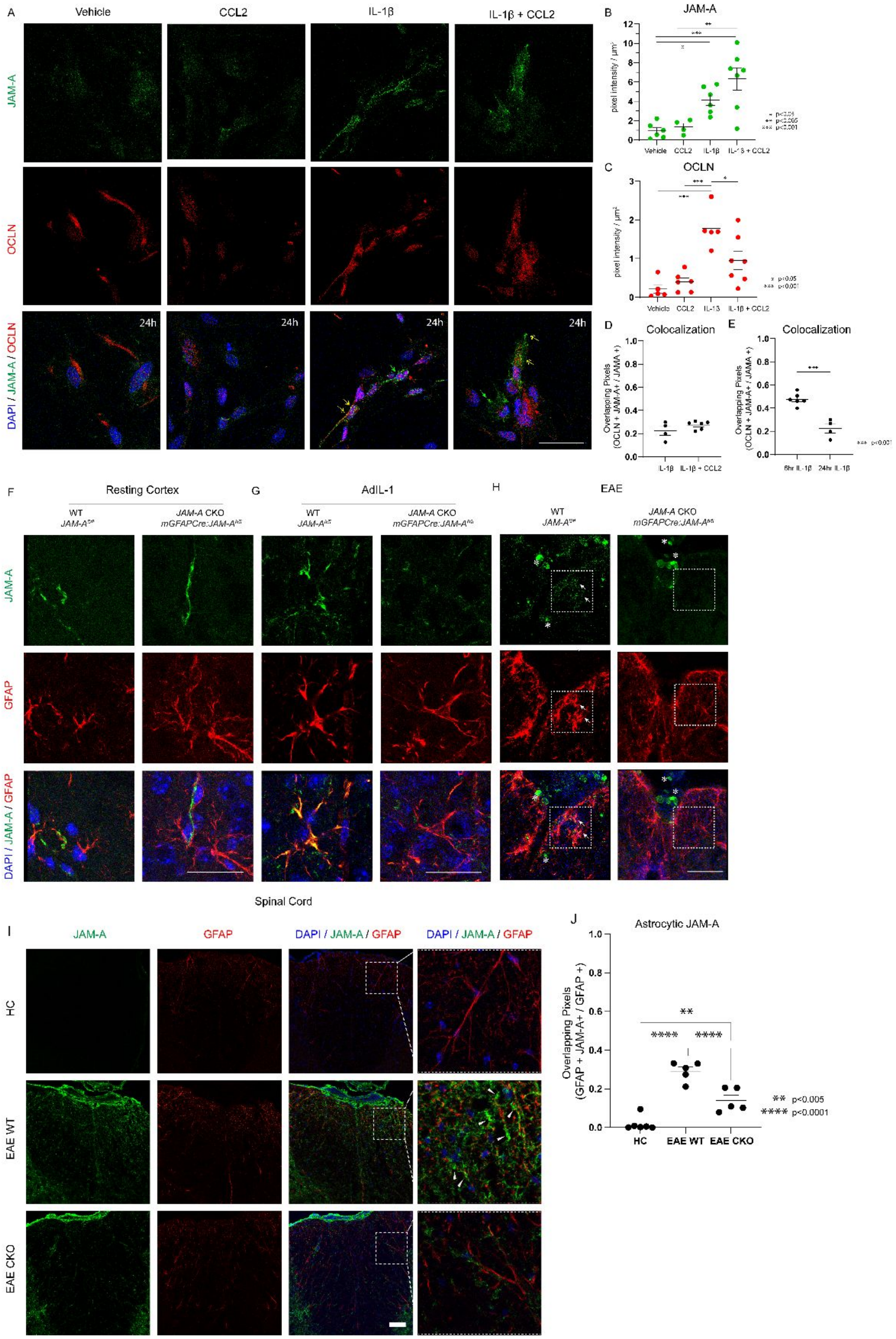
A
Vehicle
CCL2
IL-1β
IL-1β + CCL2
JAM-A
OCLN
DAPI / JAM-A / OCLN
24h
B
JAM-A
pixel intensity / μm²
C
OCLN
D
Colocalization
E
Colocalization
Overlapping Pixels
F
Resting Cortex
G
AdIL-1
H
EAE
WT
JAM-A CKO
GFAP
DAPI / JAM-A / GFAP
Spinal Cord
I
HC
EAE WT
EAE CKO
J
Astrocytic JAM-A
Overlapping Pixels (GFAP + JAM-A+ / GFAP +)
** p<0.005
**** p<0.0001

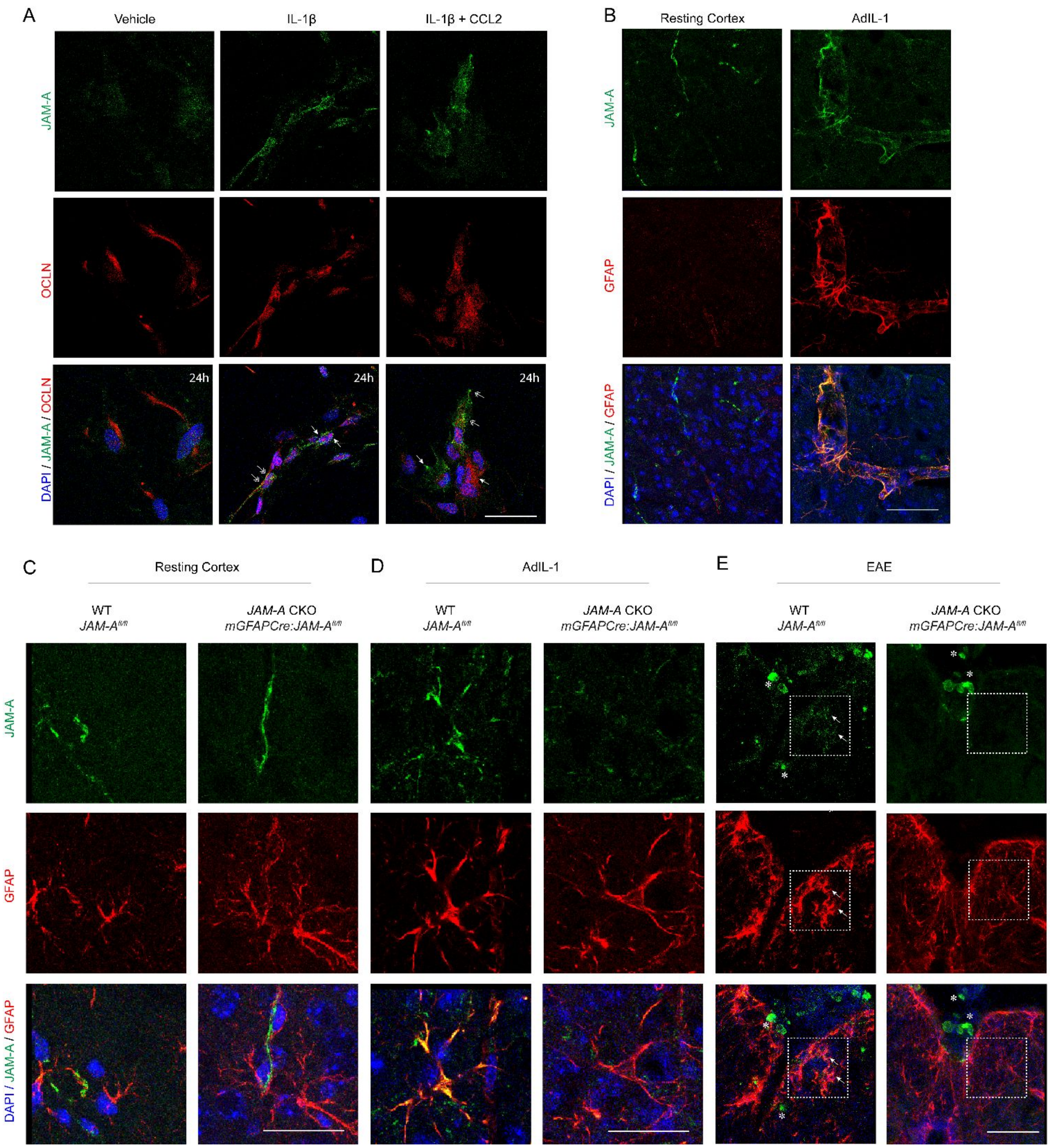
A
Vehicle
IL-1β
IL-1β + CCL2
JAM-A
OCLN
DAPI / JAM-A / OCLN
24h
24h
24h
B
Resting Cortex
AdIL-1
JAM-A
GFAP
DAPI / JAM-A / GFAP
C
Resting Cortex
D
AdIL-1
E
EAE
WT
JAM-A fl/fl
JAM-A CKO
mGFAPCre:JAM-A fl/fl
WT
JAM-A fl/fl
JAM-A CKO
mGFAPCre:JAM-A fl/fl
WT
JAM-A fl/fl
JAM-A CKO
mGFAPCre:JAM-A fl/fl
JAM-A
GFAP
DAPI / JAM-A / GFAP

Figure 1: **Inflammation induces reactive astrocytes to express JAM-A diffusely throughout the cell surface membrane *in vitro* and *in vivo* and this expression was successfully prevented using an astrocyte-specific JAM-A knock-out mouse line.** (A-B) Astrocytic JAM-A (green) was induced *in vitro* at 24 hours after treatment with 20ng/mL IL-1β and the combination of IL-1β + 100ng/mL CCL2 but not CCL2 alone (average vehicle (0.93) vs. IL-1β (4.14) vs. CCL2 (1.36) vs. IL-1β + CCL2 (6.316), vehicle vs. IL-1β: $p=0.04$, vehicle vs. IL-1β + CCL2: $p=0.0004$, CCL2 vs. IL-1β + CCL2: $p=0.0030$, other comparisons $p>0.05$, analyzed images n=6 vehicle, n=4 CCL2, n=6 IL-1β, n=7 IL-1β + CCL2, one way ANOVA with Tukey's multiple comparison test). JAM-A was both diffusely localized throughout the cell membrane (green arrows) and co-localized with the tight junction marker, occludin (OCLN, red, red arrows; white double headed arrows pointing to overlay of the two proteins in yellow). All results were quantified from at least 4 fields of view from two to three technical replicates per group. Scale bar 50 µm. (C) Astrocytic occludin was similarly induced at 24 hours after treatment with IL-1β but not CCL2 or the combination of IL-1β and CCL2 (average vehicle (0.21) vs. IL-1β (1.78) vs. CCL2 (0.39) vs. IL-1β + CCL2 (0.95), vehicle vs. IL-1β: $p=0.0002$, CCL-2 vs. IL-1β: $p=0.0004$, IL-1β vs. IL-1β + CCL2: $p=0.028$, other comparisons $p>0.05$, analyzed images n=5 vehicle, n=6 CCL2, n=5 IL-1β, n=7 IL-1β + CCL2, one way ANOVA with Tukey's multiple comparison test). (D) The addition of CCL2 to IL-1β did not change the proportion of JAM-A$^+$ pixels colocalized with OCN$^+$ pixels at 24 hours. (IL-1β (0.22) vs. IL-1β + CCL2 (0.27), $p=0.19$, analyzed images n=4 IL-1β, n=6 IL-1β + CCL2, unpaired two-tailed t-test. (E) In IL-1β treated cultures, the proportion of JAM-A$^+$ pixels colocalized with OCN$^+$ pixels decreased over time from 6 hours to 24 hours (average at 6 hr (0.47) vs 24 hr (0.21), $p<0.0003$, analyzed images n=6 at 6 hr, n=4 at 24 hr, unpaired two-tailed t-test).~~Exposure of astrocytes cultured *in vitro* to 20ng/ml of IL-1β for 24~~

For Review Only

~~hours induces astrocytic expression of JAM-A (green), which is both diffusely localized throughout the cell membrane (white arrows) and co-localized with the tight junction marker, occludin (OCLN, red; white double headed arrows pointing to overlay of the two proteins in yellow). The addition of CCL-2 did not change the distribution of JAM-A. Scale bar 50 µm. Results were confirmed in at least 3 fields of view on two to three technical replicates in each group. (B) In healthy (resting) cortex, both JAM-A (green) and GFAP (red) are minimally expressed and do not co-localize. After intracortical injection of IL-1β expressing adenovirus (AdIL-1), JAM-A (green) is detected most prominently in the reactive astrocytic endfeet (GFAP, red; co-localization, yellow) encircling the blood vessel wall. Scale bar 50 µm.~~ (~~C-E~~F-H) JAM-A and GFAP ~~Expression~~ expression patterns were visualized *in vivo* ~~of JAM-A and GFAP~~ in resting cortex, AdIL-1 and EAE spinal cord tissue of control (WT) (*$JAMA^{fl/fl}$*), and *JAM-A* conditional knock-out (CKO) (*$mGFAPCre{:}JAMA^{fl/fl}$*) mice. In the resting cortex (F~~C~~), JAM-A (green) does not strongly overlap (yellow)~~of the vascular endothelium abuts~~with astrocytes ~~the astrocytic endfeet~~ (GFAP, red) ~~but is not expressed by astrocytes. In the~~In the inflamed cortex of WT AdIL-1 brains (G~~D~~), JAM-A (green) overlaps with~~is expressed by astrocytes, co-localizing~~ with GFAP (red, overlap: yellow) and~~. This~~ astrocytic ~~pattern of~~ JAM-A appears diminished ~~expression is lost~~ in CKOs. Scale bars in (F, G~~C) and (D~~) 25 µm. In EAE spinal cord at Day 28 (H~~E~~), JAM-A (green) overlaps with ~~is expressed in WTs by~~ reactive astrocytes (GFAP, red) of the glia limitans (area of interest outlined in the dotted white box with white arrows pointing to JAM-A within the astrocytic processes), as well as by leukocytes infiltrating the subarachnoid space and CNS parenchyma (white asterisks). In CKOs, leukocytes (white asterisks), but not astrocytes, show~~express~~ JAM-A signal~~(white asterisks)~~. Scale bar in (~~E~~H) 50 µm. (I) Astrocytic expression of JAM-A was quantified in spinal cord tissues of healthy controls (HC) and WT and CKO mice at 5 days after the onset of EAE

For Review Only

(clinical scores ranging 2.5-3.5 for both groups). Images show immunofluorescences for JAM-A (green), GFAP (red), and DAPI (blue) in the spinal cord dorsal column of HC, WT and CKO mice with EAE. JAM-A expression is nearly undetectable in the spinal cord of HC mice, while it is upregulated in EAE WT and EAE CKO mice. EAE CKO mice show decreased immunoreactivity to JAM-A in GFAP positive astrocytes compared with EAE WT as shown in the higher magnification inset (white dashed square). White arrowheads point to JAM-A$^+$ GFAP$^+$ astrocytes. Scale bar = 50 um. (J) Colocalization analysis shows a greater proportion of GFAP$^+$ pixels that co-localize with JAM-A$^+$ pixels during EAE compared with HC (average HC (0.19) vs. EAE WT (0.29) vs. EAE CKO (0.14), HC vs. EAE WT: $p<0.0001$; HC vs EAE CKO: $p=0.0036$) and a decreased proportion in EAE CKO mice compared to EAE WT mice (EAE WT vs. EAE CKO: $p=0.0009$), number of animals HC, n=6; EAE WT, n=5; EAE CKO, n=5, one way ANOVA with Tukey's multiple comparison test. Image analysis was performed on at least 2 images per animal.

For Review Only

Figure 2

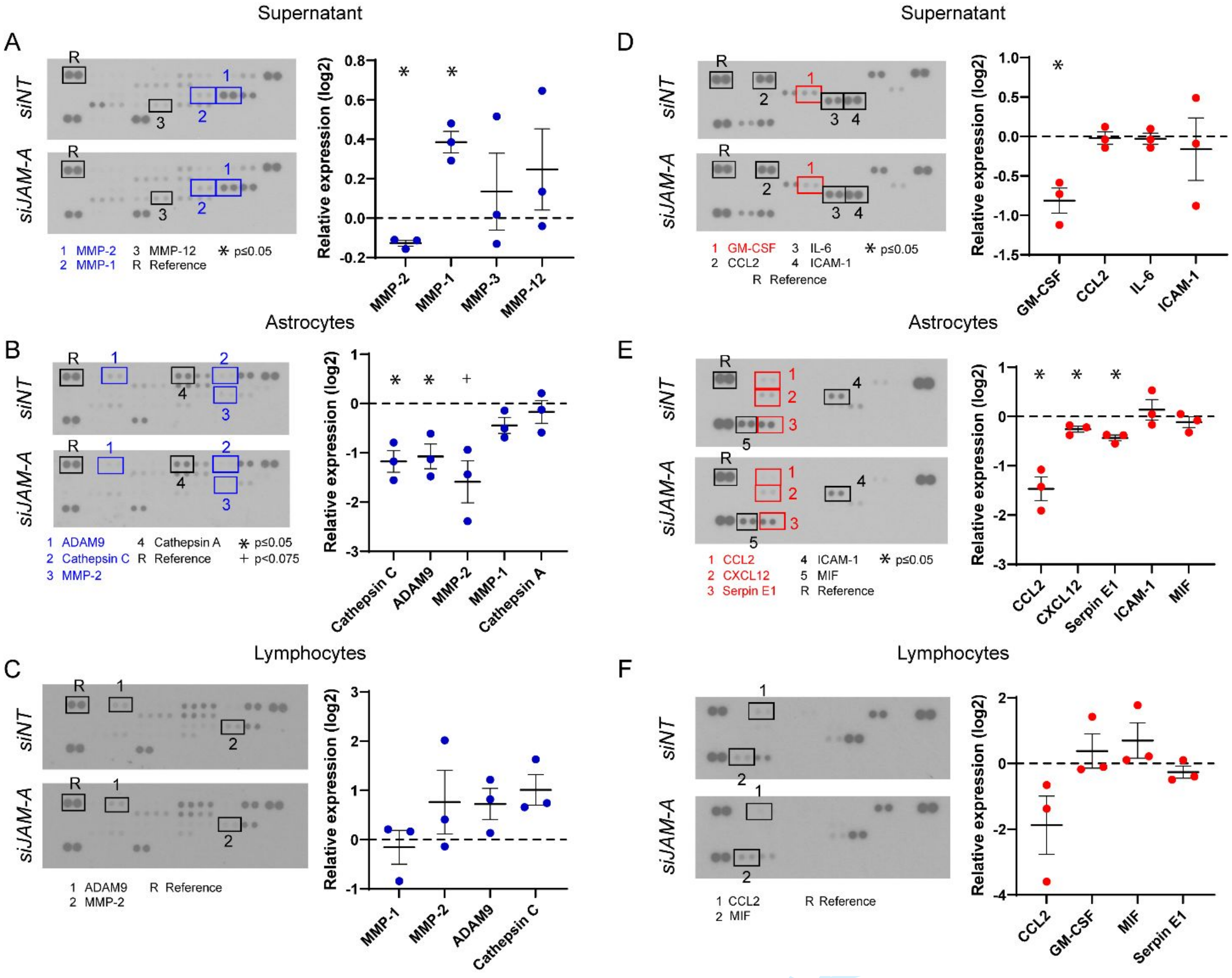
Supernatant
A
siNT
siJAM-A
1 MMP-2
2 MMP-1
3 MMP-12
R Reference
* p≤0.05
Relative expression (log2)
MMP-2
MMP-1
MMP-3
MMP-12
Supernatant
D
1 GM-CSF
2 CCL2
3 IL-6
4 ICAM-1
R Reference
GM-CSF
CCL2
IL-6
ICAM-1
Astrocytes
B
1 ADAM9
2 Cathepsin C
3 MMP-2
4 Cathepsin A
+ p<0.075
Cathepsin C
ADAM9
Cathepsin A
Astrocytes
E
1 CCL2
2 CXCL12
3 Serpin E1
4 ICAM-1
5 MIF
CXCL12
Serpin E1
MIF
Lymphocytes
C
1 ADAM9
2 MMP-2
Lymphocytes
F
1 CCL2
2 MIF

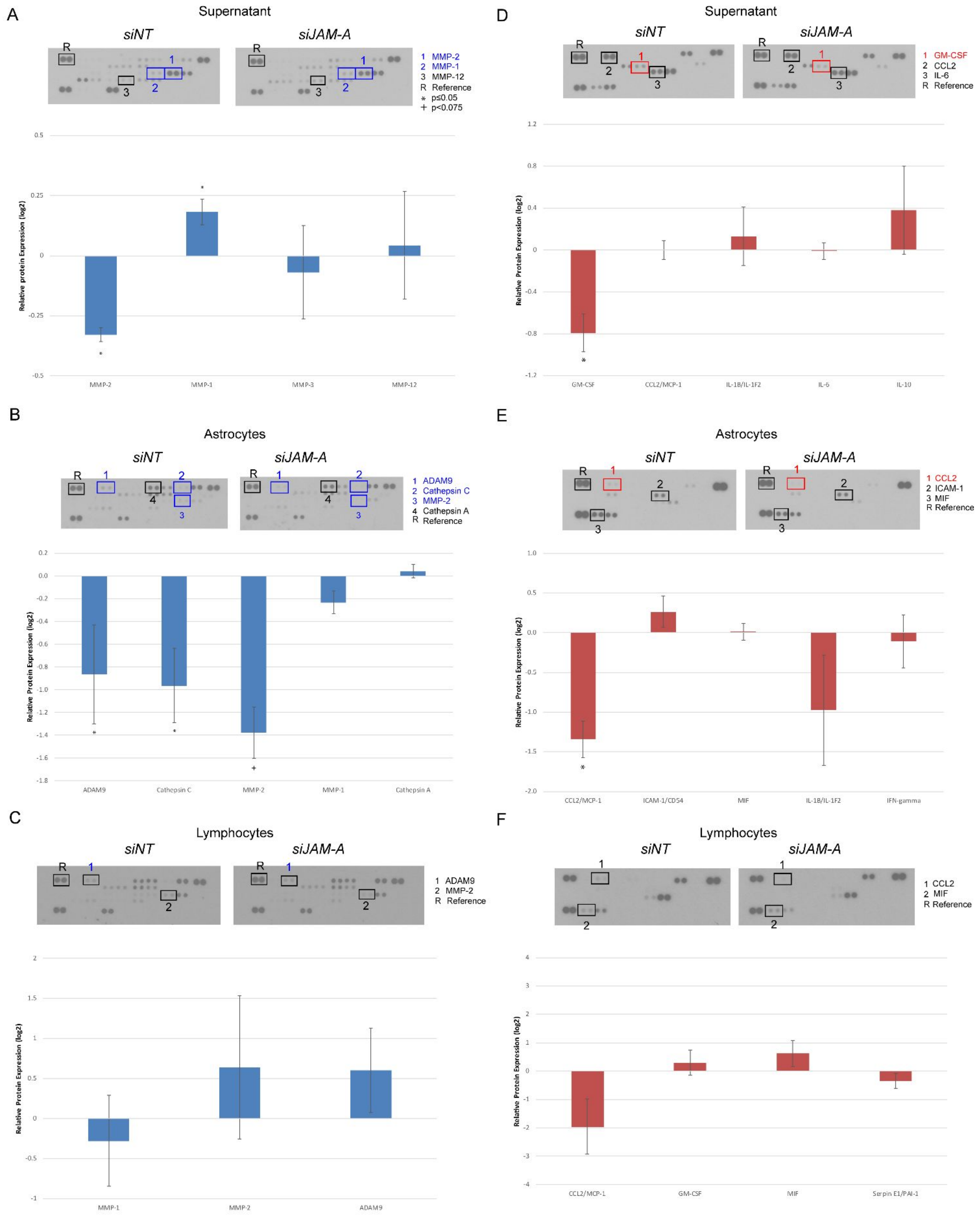
A
Supernatant
siNT
siJAM-A
R
1 MMP-2
2 MMP-1
3 MMP-12
R Reference
* p≤0.05
+ p<0.075
Relative protein Expression (log2)
MMP-2
MMP-1
MMP-3
MMP-12
B
Astrocytes
siNT
siJAM-A
1 ADAM9
2 Cathepsin C
3 MMP-2
4 Cathepsin A
R Reference
Relative Protein Expression (log2)
ADAM9
Cathepsin C
MMP-2
MMP-1
Cathepsin A
C
Lymphocytes
siNT
siJAM-A
1 ADAM9
2 MMP-2
R Reference
Relative Protein Expression (log2)
MMP-1
MMP-2
ADAM9
D
Supernatant
siNT
siJAM-A
1 GM-CSF
2 CCL2
3 IL-6
R Reference
Relative Protein Expression (log2)
GM-CSF
CCL2/MCP-1
IL-1B/IL-1F2
IL-6
IL-10
E
Astrocytes
siNT
siJAM-A
1 CCL2
2 ICAM-1
3 MIF
R Reference
Relative Protein Expression (log2)
CCL2/MCP-1
ICAM-1/CD54
MIF
IL-1B/IL-1F2
IFN-gamma
F
Lymphocytes
siNT
siJAM-A
1 CCL2
2 MIF
R Reference
Relative Protein Expression (log2)
CCL2/MCP-1
GM-CSF
MIF
Serpin E1/PAI-1

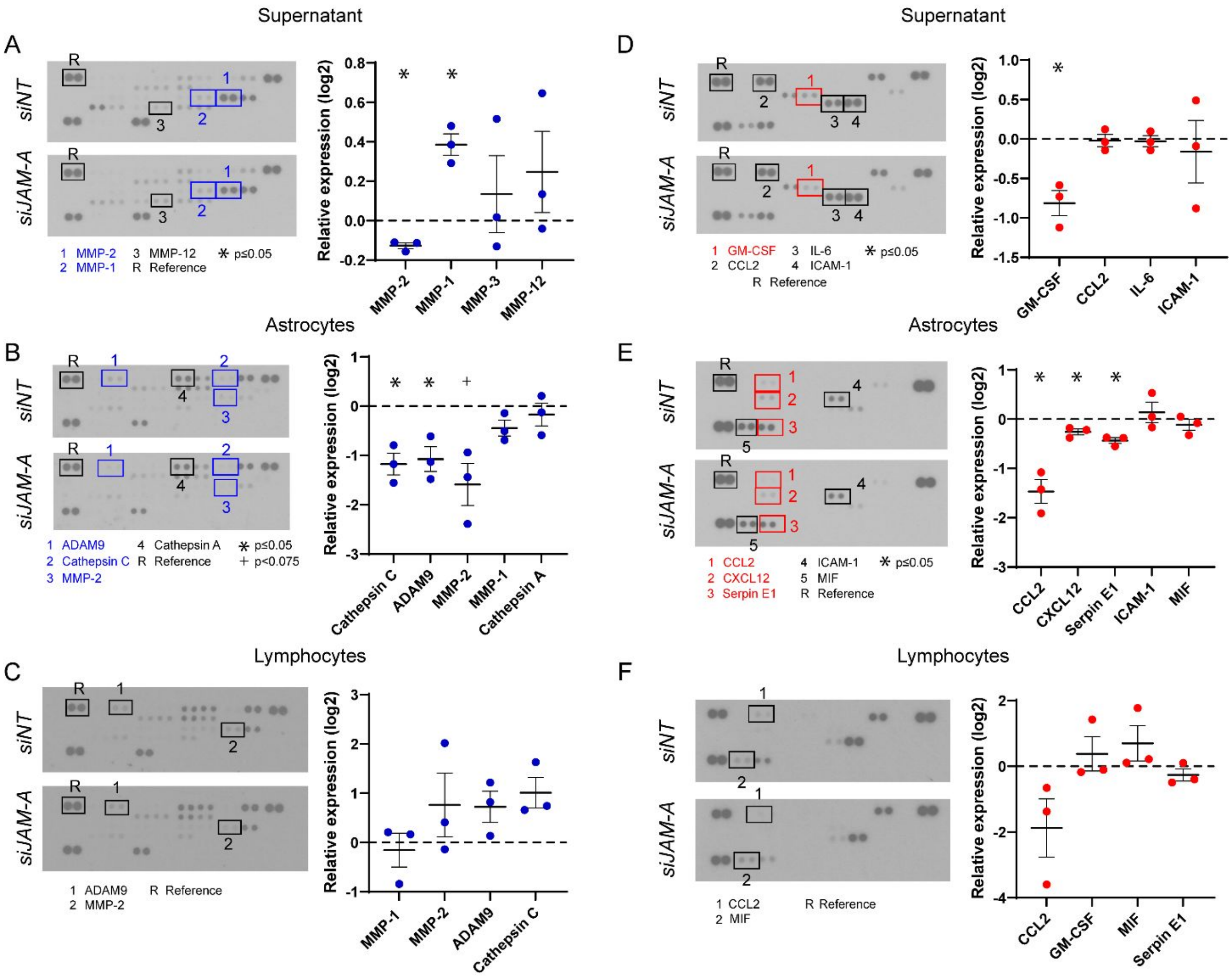

Figure 2: Astrocytic JAM-A increases pro-inflammatory protease and cytokine levels in astrocyte-CD3+ T cell co-culture. Astrocytes were transfected with JAM-A or non-targeted siRNA (*siJAM-A* vs. *siNT*, then co-cultured with CD3$^+$ T cells for 24 hours and samples processed for human protease and cytokine ELISA immunoassays. (A-C) JAM-A knock-down in astrocytes led to an increase of MMP-1 (relative $\log_2$ expression 0.~~182~~ 38, p=0.019, two-tailed ~~paired~~ unpaired two sample t-test with unequal variance) and decrease of MMP-2 (relative $\log_2$ expression -0.~~329~~13, p=0.014) in the supernatant and decrease of ADAM9 (relative $\log_2$ expression -~~0.86~~1.074, p=0.05) and cathepsin C (relative $\log_2$ expression -~~0.96~~1.174, p=0.006~~3~~) in astrocyte lysates (B). There were no significant changes in protease levels seen in lymphocyte

lysates (C). (D-F) Astrocytic JAM-A knock down led to decreased levels of (D) GM-CSF (relative log_2 expression -0.79, p=0.04) in the supernatant and (E) CCL-2 (relative log_2 expression -1.3, p=0.03) in astrocytic lysates. There were no significant changes in cytokine levels seen in lymphocyte lysates (F). Data (A-F) are from three biological replicates; two-tailed paired t-tests were performed on probes demonstrating a visually detectable ~~difference~~ signal in normalized expression values relative to a reference control.

For Review Only

Figure 3

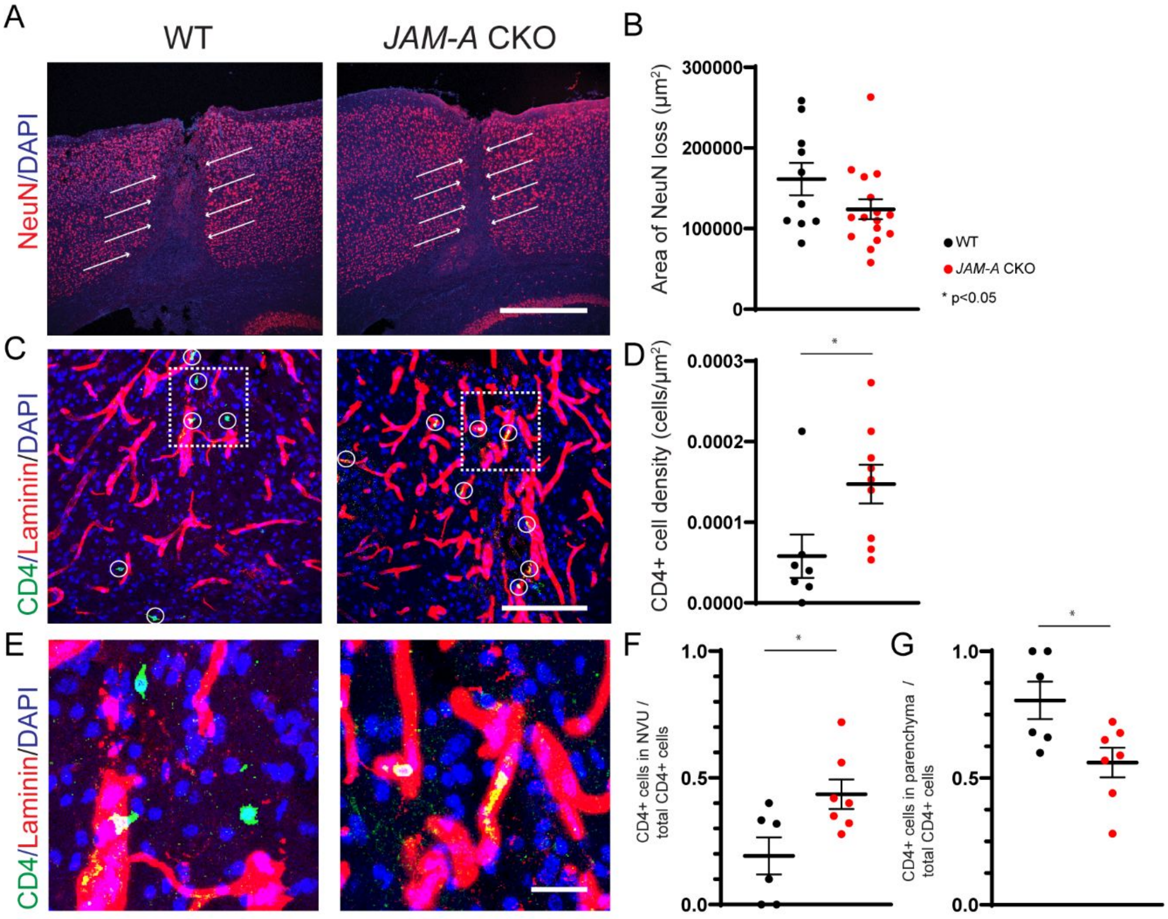

Only

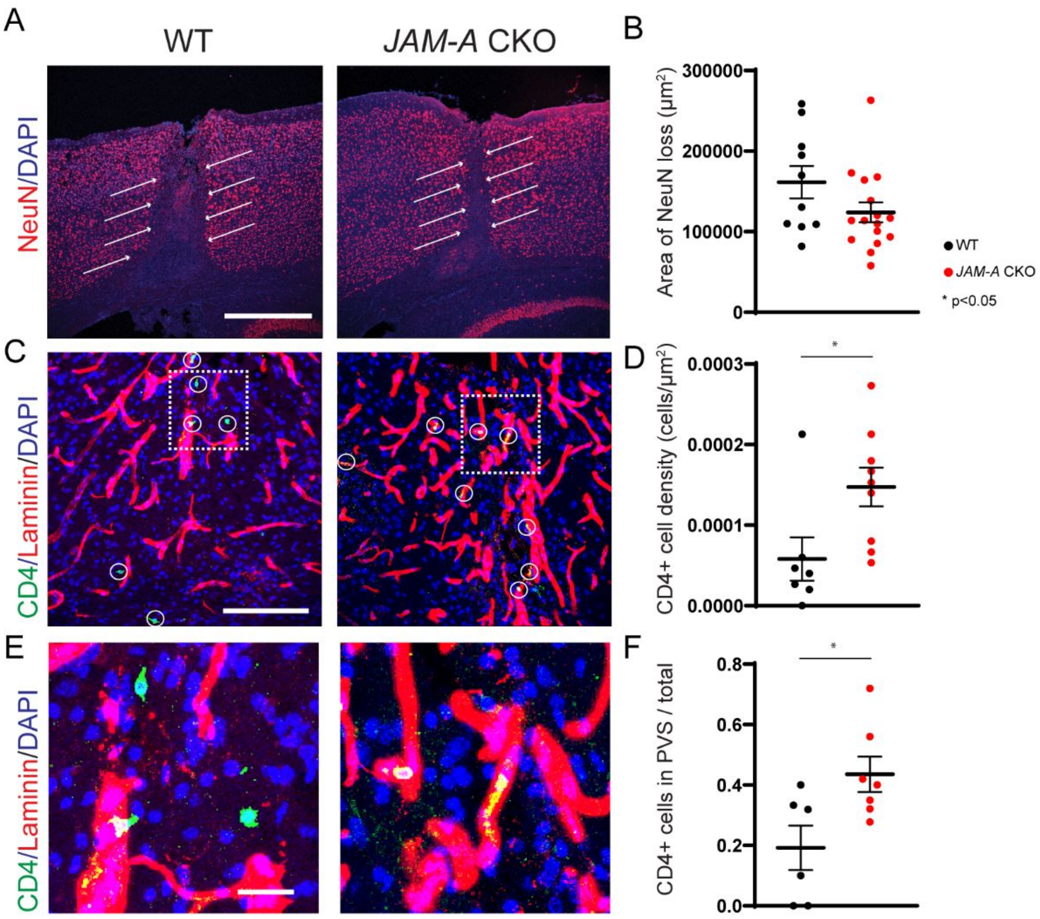

Figure 3: **In inflammatory cortical lesions, CD4$^+$ T cells are arrested at the neurovascular unit (NVU)~~in the perivascular spaces~~ in the absence of astrocytic JAM-A**. Asymptomatic

inflammatory cortical lesions were induced in JAM-A CKO and WT mice with an IL-1β expressing adenovirus (ADIL-1) microinjected into the frontal cortex, with brains harvested for histopathology at 7 days post-injection. (A,B) Lesions in JAM-A CKO mice, as measured by the area of neuronal cell death (loss of NeuN, red, white arrows) showed a trend in smaller lesion size compared to WT mice that did not reach statistical significance (p= 0.18, CKO n=11 mice, WT n=8 mice, Mann Whitney test). (C, D) CD4$^+$ cells (green and circled in white) were increased in number in JAM-A CKO lesions, scale bar: 125 μm (average number~~-~~/μm^2~~per 40x field~~, ~~22.1~~1.5 x 10^{-4} (CKO) vs. ~~8.7~~5.8 x 10^{-5} (WT), p=0.012, for WT, a range of 0 to 78 cells were analyzed per mouse (Average 22.6); for CKO, a range of 8 to 230 cells were analyzed per mouse (average 47); CKO n=9 mice~~KO~~, WT n=7 mice, Mann-Whitney test). (E-G~~,F~~) In JAM-A CKO mice, a higher proportion of CD4$^+$ cells (green) co-localize to the ~~PVS (~~the laminin (red) positive basement membrane of the neurovascular unit (NVU~~)~~ and a lower proportion in the parenchyma (laminin-negative CNS parenchyma demarcated by DAPI) than in WT mice ~~.~~(F) ~~average~~ Average proportion~~ercentage~~ of CD4$^+$ cells in NVU~~PVS/~~ over total CD4$^+$ cells: 0.43~~.5~~ (CKO) vs. 0.19~~.2~~ (WT), p=0.03~~.~~; (G) Average proportion of CD4$^+$ cells in the parenchyma /total CD4$^+$ cells: 0.81 (CKO) vs 0.56 (WT), p=0.03; in the WT, a range of 1 to 10 cells (average 6.5) were analyzed per mouse; in the CKO, a range of 5 to 18 cells (average 10.1) were analyzed per mouse; CKO n=8 mice, WT n=6 mice, Mann-Whitney tests; scale bar: 25μm).

For Review Only

Figure 4

For Review Only

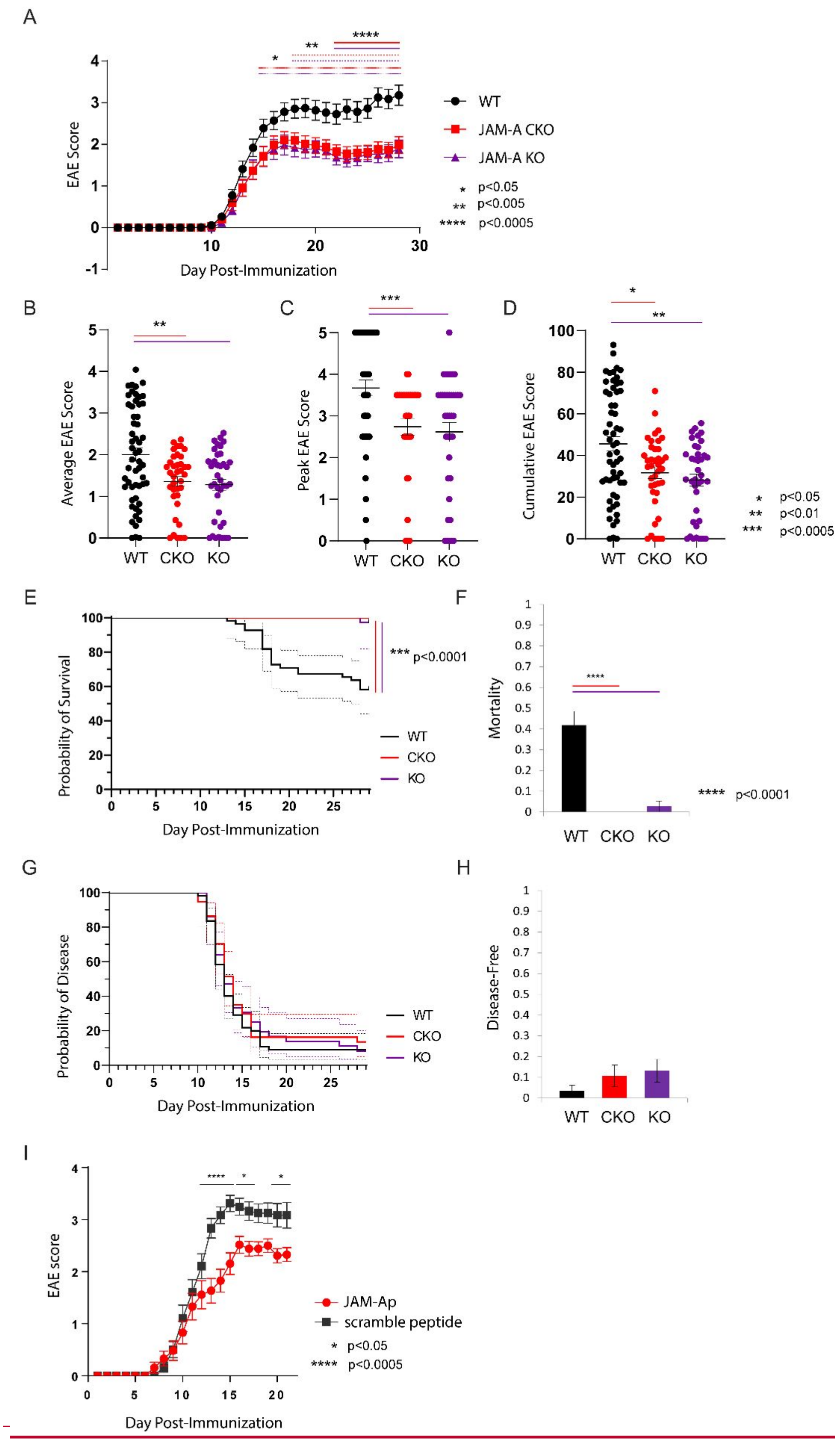
A
EAE Score
Day Post-Immunization
WT
JAM-A CKO
JAM-A KO
* p<0.05
** p<0.005
**** p<0.0005
B
Average EAE Score
WT CKO KO
C
Peak EAE Score
WT CKO KO
D
Cumulative EAE Score
WT CKO KO
* p<0.05
** p<0.01
*** p<0.0005
E
Probability of Survival
Day Post-Immunization
*** p<0.0001
WT
CKO
KO
F
Mortality
WT CKO KO
**** p<0.0001
G
Probability of Disease
Day Post-Immunization
WT
CKO
KO
H
Disease-Free
WT CKO KO
I
EAE score
Day Post-Immunization
JAM-Ap
scramble peptide
* p<0.05
**** p<0.0005

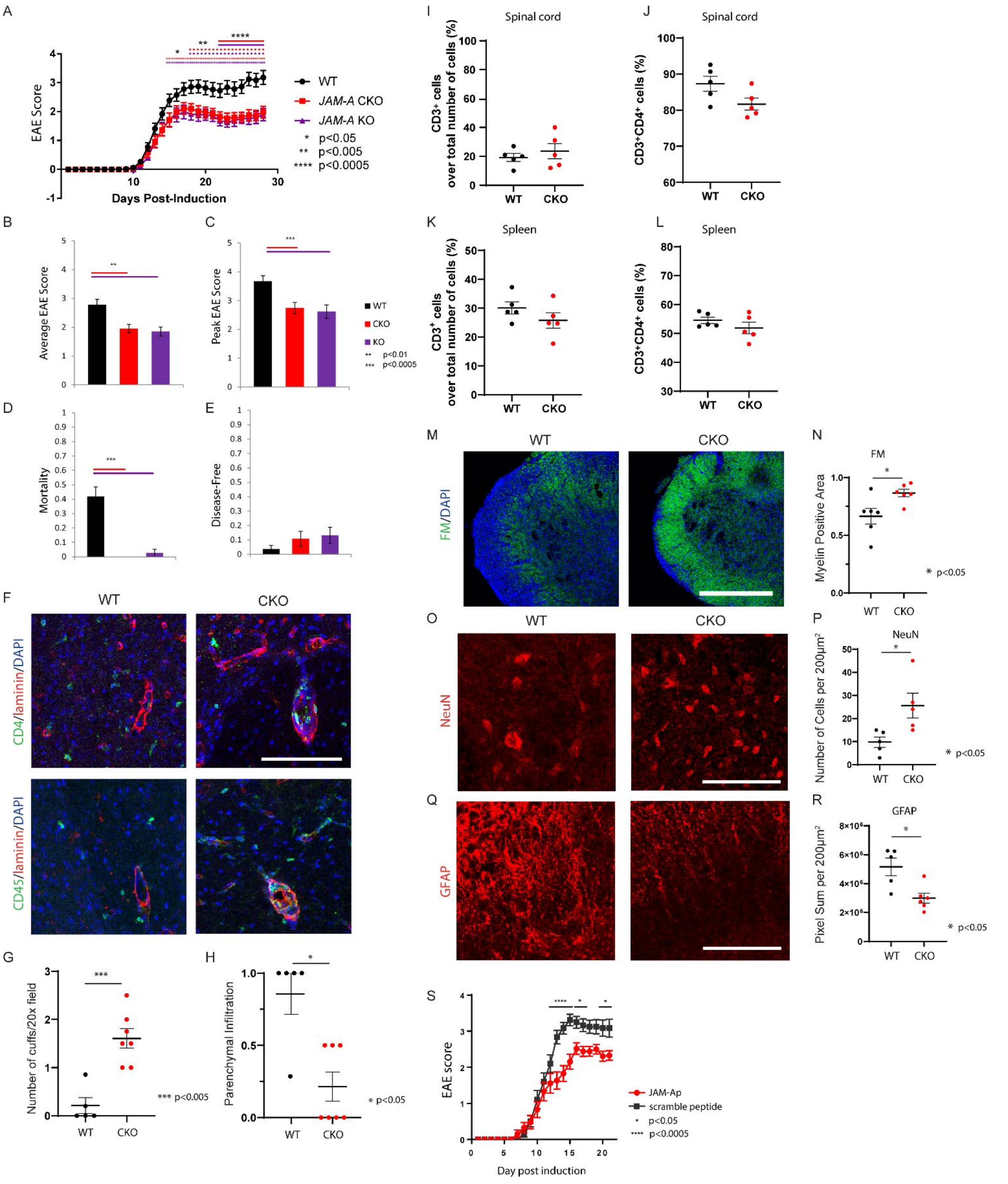
A
EAE Score
Days Post-Induction
WT
JAM-A CKO
JAM-A KO
* p<0.05
** p<0.005
**** p<0.0005
B
Average EAE Score
C
Peak EAE Score
WT
CKO
KO
** p<0.01
*** p<0.0005
D
Mortality
E
Disease-Free
F
WT
CKO
CD4/laminin/DAPI
CD45/laminin/DAPI
G
Number of cuffs/20x field
*** p<0.005
H
Parenchymal Infiltration
* p<0.05
I
Spinal cord
CD3+ cells over total number of cells (%)
J
Spinal cord
CD3+CD4+ cells (%)
K
Spleen
CD3+ cells over total number of cells (%)
L
Spleen
CD3+CD4+ cells (%)
M
FM/DAPI
N
FM
Myelin Positive Area
* p<0.05
O
NeuN
P
NeuN
Number of Cells per 200µm²
* p<0.05
Q
GFAP
R
GFAP
Pixel Sum per 200µm²
* p<0.05
S
EAE score
Day post induction
JAM-Ap
scramble peptide
* p<0.05
**** p<0.0005

Figure 4: Astrocytic JAM-A ~~promotes clinical disability and T lymphocyte entry into the CNS parenchyma, demyelination, neuronal loss and astrocyte reactivity during EAE~~promotes clinical disease severity during EAE. (A) JAM-A CKO and KO mice showed a milder course of clinical disability than WT mice with EAE and there~~There~~ was no difference between CKO and KO animals.; (WT vs. CKO: p=0.01, WT vs KO: p<0.0001, CKO vs KO: p=0.07 using non-parametric one way ANOVA Friedman test with Dunn's multiple comparison test; day 15-17: p<0.05 for WT vs CKO and WT vs. KO; day 18-21: p<0.005 for WT vs. CKO and WT vs. KO; day 22-25: p<0.0005 for WT vs. CKO and WT vs. KO, two-way ANOVA with Bonferroni correction). ~~There was no difference between CKO and KO animals.~~ Graph shows pooled data from 2-3 independent EAE experiments with a minimum of 8 mice per group in each experiment, total WT n=55, JAM-A CKO n=37, JAM-A KO n=38. (B-~~E~~D) Average (B),~~ and~~ peak (C) and cumulative (D) ~~EAE~~ scores of the EAE trial shown in (A)~~, as well as mortality (D)~~ were~~as~~ significantly lower in CKO and KO mice compared to WT (average score: 2.0 (WT) vs. 1.36 (CKO) vs. 1.28 (KO), WT vs. CKO: p=0.~~005~~~~9~~03, WT vs. KO: p=0.01, CKO vs. KO: p>0.999; peak score: 3.6 (WT) vs. 2.7 (CKO) vs. 2.6 (KO), WT vs. CKO: p=0.003, WT vs. KO: p=0.002, CKO vs. KO: p>0.999~~p=.0004;~~, cumulative score: 45.58 (WT) vs. 31.75 (CKO) vs. 28.2 (KO), WT vs. CKO: p=0.003, WT vs. KO: p=0.002, CKO vs. KO: p>0.999 ~~mortality: 0.41 (WT) vs. 0 (CKO) vs. 0.26 (KO), p<0.0001) while (E) a non-significant increased proportion of CKO and KO mice were resistant to disease induction compared with WT controls (0.014 (WT) vs 0.108 (CKO) vs. 0.131 (KO), p=0.22~~, Kruskal-Wallis ~~test for all comparisons~~tests. Average in bar graphs shown with SEM. (E) Survival curves of mortality revealed that WT mice sustained greater mortality over the course of EAE than CKO or KO, Mantel-Cox test, p<0.0001. (F) At Day 28, mortality rate was higher in WT mice compared to CKO and KO (0.41 (WT) vs. 0

For Review Only

(CKO) vs. 0.26 (KO), $p<0.0001$, Kruskal-Wallis test). (G) Disease curves demonstrated no differences in susceptibility to or timing of disease, Mantel-Cox test, $p=0.65$. (H) At Day 28, rates of disease resistance (proportion of mice that did not develop neurological deficit) showed an increased trend that was not statistically significant for CKO and KO compared to WT (0.014 (WT) vs 0.108 (CKO) vs. 0.131 (KO), $p=0.22$, Kruskal-Wallis test).

(~~S~~I) WT mice with EAE treat~~men~~ed ~~t~~ with daily intraperitoneal injection of a JAM-A blocking peptide (JAM-Ap) from day 7 post-immunization ~~were protected against~~showed a milder course of clinical disability compared to scramble peptide treated controls (scramble vs. JAM-A: $p=0.04$~~day~~ by non-parametric Kolmogorov-Smirnov test comparing cumulative distributions; at Day 13-15, $p<0.0001$, day 16-17, $p<0.05$, day 20-21, $p<0.05$; total JAM-Ap n=24, scramble n=26, two-way ANOVA with Bonferroni correction; graph shows pooled data from 3 independent EAE experiments with a minimum of 8 mice per group for each experiment~~; .total JAM-Ap n=24, scramble n=26, two-way ANOVA with Bonferroni correction~~).

For Review Only

Figure 5

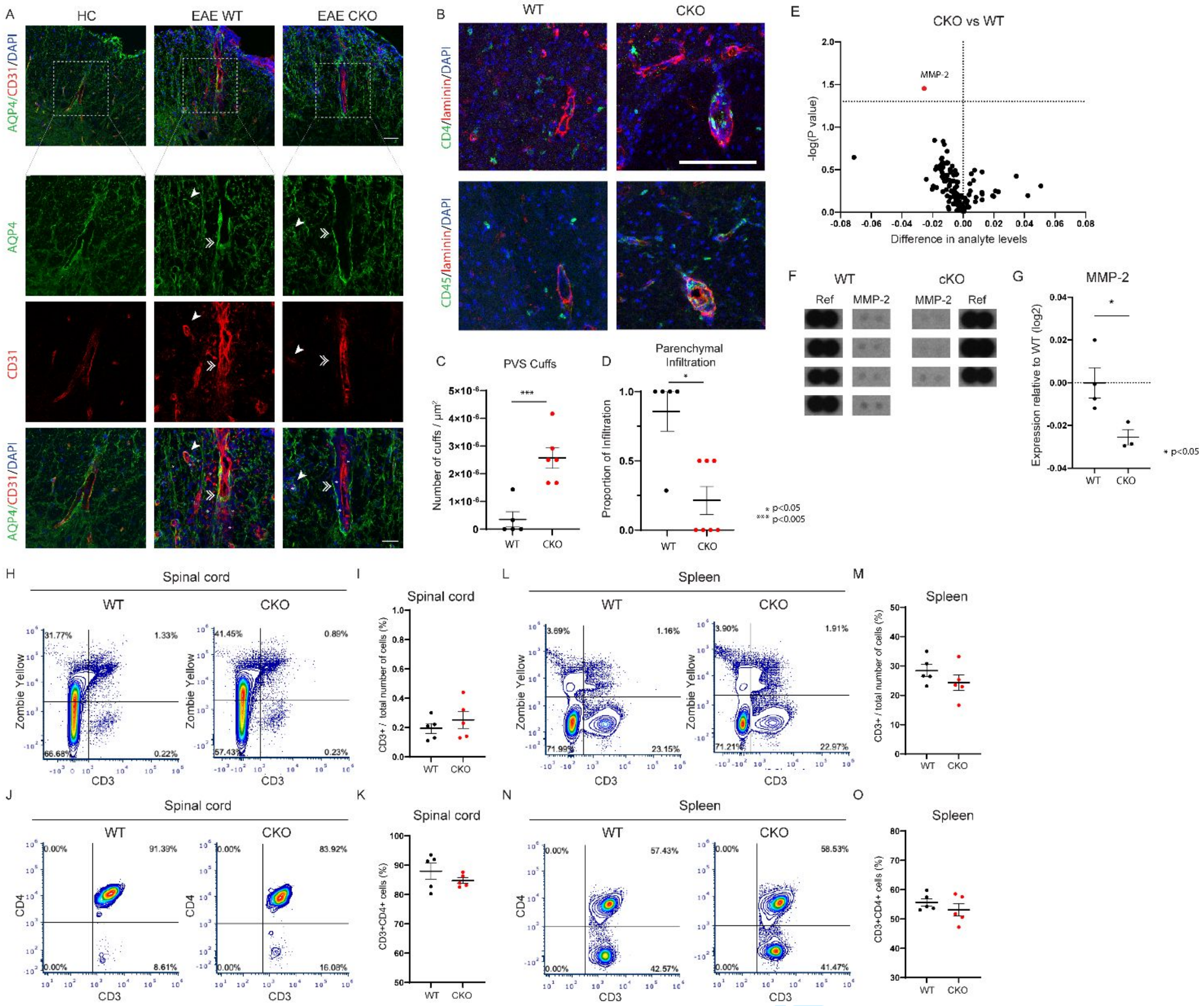
A
HC
EAE WT
EAE CKO
AQP4/CD31/DAPI
AQP4
CD31
B
WT
CKO
CD4/laminin/DAPI
CD45/laminin/DAPI
C
PVS Cuffs
Number of cuffs / µm²
D
Parenchymal Infiltration
Proportion of Infiltration
* p<0.05
*** p<0.005
E
CKO vs WT
MMP-2
-log(P value)
Difference in analyte levels
F
WT
cKO
Ref
MMP-2
G
MMP-2
Expression relative to WT (log2)
* p<0.05
H
Spinal cord
Zombie Yellow
CD3
I
Spinal cord
CD3+ / total number of cells (%)
L
Spleen
M
Spleen
CD3+ / total number of cells (%)
J
Spinal cord
CD4
K
Spinal cord
CD3+CD4+ cells (%)
N
Spleen
O
Spleen
CD3+CD4+ cells (%)
WT
CKO

Figure 5: Astrocytic JAM-A promotes T lymphocyte entry into the CNS parenchyma from the perivascular spaces via MMP-2 in EAE. (A) High power images of the neurovascular unit and perivascular spaces were identified using aquaporin-4 staining of the astrocyte endfeet and CD31 staining of the endothelium in HC, WT and CKO mice. Cell infiltrates (DAPI, blue) were seen in the CNS at 5 days post EAE disease onset in both WT and CKO but not in HC. Representative images demonstrate that in JAM-A CKO, immune cells accumulated within the perivascular spaces (between aquaporin-4 and CD31) whereas in WT, immune cells localized diffusely past the perivascular spaces within the CNS parenchyma. Scale bar 50 μm top panels, 30 μm bottom panels representing inset outlined by dotted white box. (~~F~~B)~~) Images show i~~In inflammatory lesions ~~in the spinal cord of CKO and WT mice with~~of EAE at ~~day~~ Day 21 post-immunization, i~~. I~~n CKO mice, $CD4^+$ (green, upper panel) and $CD45^+$ cells (green, lower panel) ~~were mostly~~ clustered in perivascular (PVS) "cuffs," colocalizing with the pan-laminin marker (in red), whereas in WT mice, $CD4^+$ and $CD45^+$ cells were instead located in the parenchyma. Scale bar 100 μm. (~~G~~C) Number of $CD4^+$ cuffs per μm^2~~quantified within a 20x field of view of~~ of lumbar~~thoracic~~ spinal cord cross-sections were increased in JAM-A CKO mice compared to WTs (average 3.5×10^{-7}~~0.21~~ (WT) vs. ~~1.61~~2.6×10^{-6} (CKO), p=0.0022~~5~~, Mann Whitney test, number of mice: WT n=5, CKO n=~~7~~6). In the WT, the number of analyzed cuffs per mouse ranged from 0 to 6 (average 1.4); in the CKO, the range was 0 to 10 (average 4.8). (~~H~~C) Proportion of sections demonstrating parenchymal infiltration of $CD4^+$ cells was~~ere~~ lower in JAM-A CKO compared to WT mice (average 0.86 (WT) vs. 0.21 (CKO), p=0.01, Mann Whitney test). (E) A Volcano Plot shows the differential level of 111 pro-inflammatory cytokines, chemokines, proteases and acute phase reactants in spinal cord lysates of JAM-A CKO and WT mice at 5 days post EAE disease onset as measured using mouse proteome ELISA immunoassays. CKO mice showed an overall

For Review Only

reduction of many factors, though MMP-2 (highlighted in red) was the sole statistically significant factor compared to WT controls. (F) MMP-2 probes on the ELISA array in CKO and WT mice, along with reference spots, used for signal (pixel intensity) normalization, are shown. Complete ELISA arrays from each mouse are shown in **Supplementary Figure 7** and original blots are included in the **Supplementary Materials**. (G) Spinal cord levels of MMP-2 in CKO mice relative to WT controls were significantly decreased at 5 days post EAE disease onset (relative log2 expression -0.025, p=0.0351, two-tailed unpaired t-test, WT, n=4; CKO, n=3). (~~I–K~~H-O) Flow cytometry was performed on spinal cords and spleens of WT and CKO mice with EAE on day 5 from onset of disease. (~~I~~H) Representative plots show (I) similar t~~T~~otal CD3^{+} cell counts ~~were similar~~ in CKO and WT (mean CKO 0.23~~%~~ vs. WT 0.19%, p= 0.~~480~~42, unpaired two-tailed t-test~~, ;~~ n=5 WT, n=5 CKO, average EAE score WT 2.8, CKO 2.6 for all flow experiments).~~-~~ (J) Representative plots reflect (K) no difference found in ~~The~~ the proportion of ~~CD4~~CD3^{+}/~~CD3~~CD4^{+} cells ~~showed a decreasing~~in ~~, statistically non-significant trend in~~ CKOs compared to WTs (mean CKO ~~81~~84.~~69~~73% vs. ~~81.69~~87.97%, p=0.~~07~~29, unpaired two-tailed t-test). (~~K, L~~L-O) In spleen, total CD3^{+} and ~~CD4~~CD3^{+}/~~CD3~~CD4^{+} were unchanged between CKO and WT groups (CD3^{+}: ~~CKO vs. WT~~, mean CKO ~~26~~24.3% vs. WT ~~30~~28.4%, p=0.~~24~~25; ~~CD4~~CD3^{+}/~~CD3~~CD4^{+}: mean CKO ~~52~~53.1% vs. ~~54~~55.6%, p=0.~~28~~34~~; CD8+/CD3+: 42% vs 41%, p=0.53,~~ unpaired two-tailed t-test). ~~(M, N) Demyelination (fluoromyelin positive area of the lumbar anterolateral white matter tracts) was spared in CKO mice compared to WTs (average 0.66 (WT) vs. 0.86 (CKO), p=0.026, Mann Whitney test). Scale bar 400 µm.~~

For Review Only

Figure 6

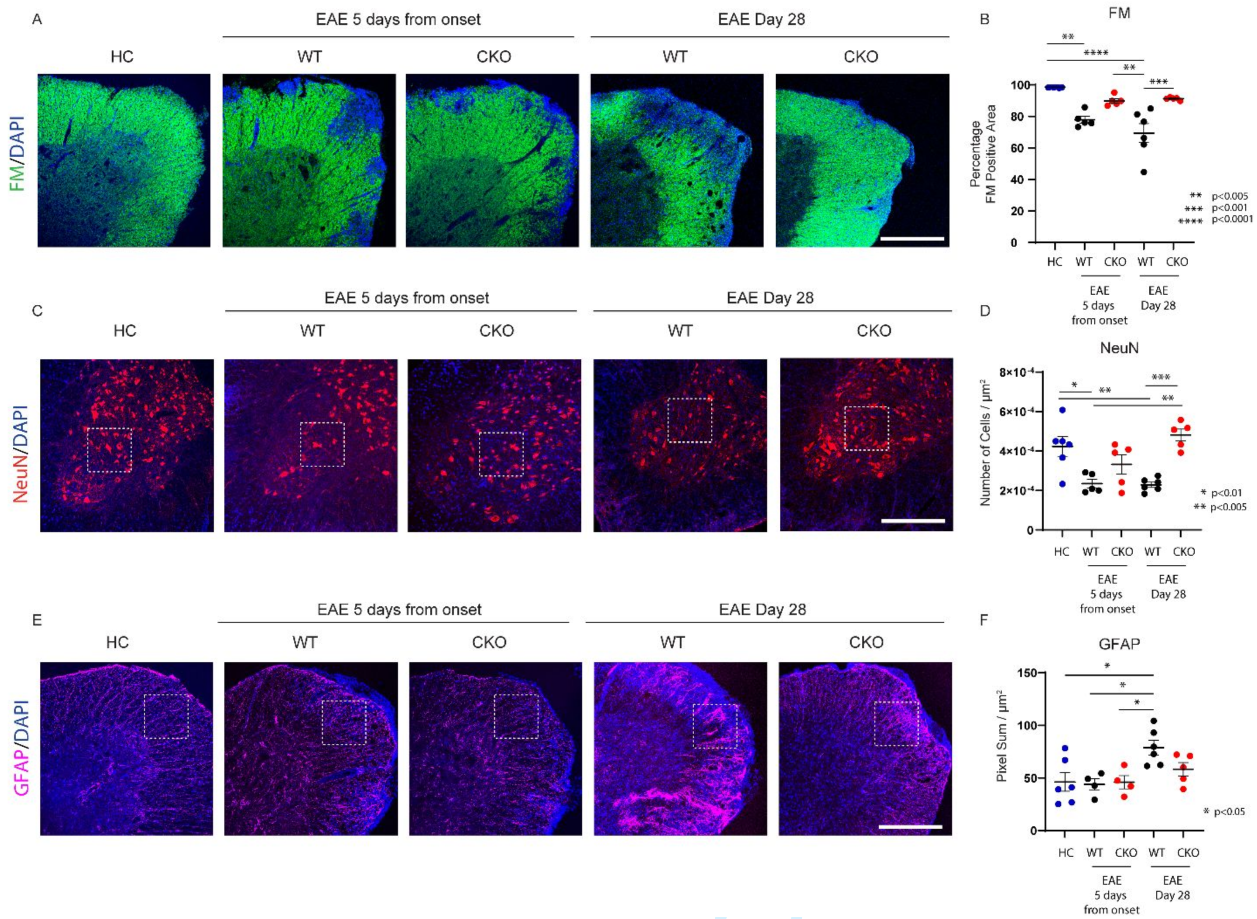
A
EAE 5 days from onset
EAE Day 28
HC
WT
CKO
WT
CKO
FM/DAPI
B
FM
Percentage FM Positive Area
** p<0.005
*** p<0.001
**** p<0.0001
HC WT CKO WT CKO
EAE 5 days from onset
EAE Day 28
C
EAE 5 days from onset
EAE Day 28
HC
WT
CKO
WT
CKO
NeuN/DAPI
D
NeuN
Number of Cells / µm²
* p<0.01
** p<0.005
E
EAE 5 days from onset
EAE Day 28
HC
WT
CKO
WT
CKO
GFAP/DAPI
F
GFAP
Pixel Sum / µm²
* p<0.05

Figure 6: Astrocytic JAM-A exacerbates histopathological markers of neuroinflammatory damage in EAE. (A, B) Proportion of fluoromyelin (FM, green; marker of myelin) positive area of the lumbar anterolateral white matter tracts was significantly increased in EAE CKO mice at a late chronic stage of disease (EAE Day 28 post immunization) compared to time matched WT mice controls (EAE WT Day 28 post immunization) and EAE WT in the acute phase of disease (EAE WT 5 days from onset) (average HC 98.53% vs. EAE WT 5 days from disease onset 77.85% vs. EAE CKO 5 days from disease onset 89.91% vs. EAE WT Day 28 post immunization 69.35% vs. EAE CKO Day 28 post immunization 91.2%; HC vs. EAE WT 5 days: p=0.001, HC vs. EAE WT Day 28: p<0.0001, EAE CKO 5 days vs. EAE WT Day 28: p=0.001, EAE WT Day 28 vs. EAE CKO Day 28: p=0.0007, all other p values>0.05, one way ANOVA with Tukey's multiple comparison test). HC n=6, EAE WT 5 days from disease onset n=5, EAE CKO 5 days from disease onset n=5, EAE WT Day 28 post immunization n=6, EAE CKO Day 28 post immunization n=5 animals. Scale bar 400 µm. -(~~O, P~~C, D) Number of NeuN$^+$ neurons per µm^2 within the ventral gray matter of the lumbar spinal cord was significantly higher in EAE CKO mice at day 5 after disease onset and at Day 28 post immunization than in time-matched WT controls~~CKO mice at EAE day 28 post-immunization were protected from neurodegeneration compared with time-matched WT controls, indicated by a greater neuronal survival in the lumbar spinal cord (number of NeuN+ neurons per 200µm^2)~~ ~~(~~(average HC 4.2 x 10^{-4} vs. EAE WT 5 days from disease onset 2.35 x 10^{-4} vs. EAE CKO 5 days from disease onset 3.32x 10^{-4} vs. EAE WT Day 28 post immunization 2.29 x 10^{-4} vs. EAE CKO Day 28 post immunization 4.82 x 10^{-4}; HC vs. EAE WT 5 days: p=0.01, HC vs. EAE WT Day 28: p=0.005, EAE WT 5 days vs. EAE WT Day 28: p=0.001, EAE WT Day 28 vs. EAE CKO Day 28: p=0.0005, all other p values>0.05, one way ANOVA with Tukey's multiple comparison test). HC n=6, WT EAE 5 days from disease

For Review Only

onset n=5, CKO 5 days from disease onset n=5, WT EAE Day 28 post induction n=6, EAE CKO Day 28 post induction n=5 animals. average 9.8 (WT) vs. 25.6 (CKO), p=0.016, WT n=5, CKO n=5, Mann Whitney test). Scale bar 100 µm. In the WT, a range of 3-15 cells (average 9.8) were analyzed per mouse; in the CKO, a range was 15 to 45 cells (average 25.6) were analyzed per mouse. (Q, RE, F) GFAP (purple, marker of astrocytes) positive pixel sum per µm^2 of the lumbar anterolateral white matter tracts showed a decreasing trend at day 28 post EAE immunization in CKO compared to WT (average HC 46.24 vs. EAE WT 5 days from disease onset 43.9 vs. EAE CKO 5 days from disease onset 46.04 vs. EAE WT Day 28 post immunization 78.96 vs. EAE CKO Day 28 post immunization 58.38; HC vs. WT Day 28: p=0.01, EAE WT 5 days vs. EAE WT Day 28: p=0.04, EAE WT Day 5 vs. EAE WT Day 28: p=0.04, all other p values>0.05, one way ANOVA with Tukey's multiple comparison test). HC n=6, EAE WT 5 days from disease onset n=4, EAE CKO 5 days from disease onset n=4, WT EAE Day 28 post induction n=6, EAE CKO Day 28 post induction n=5 animals. Astrocytic activation within white matter lesions of EAE (GFAP pixel sum per 200µm^2 area of the lumbar white matter) was decreased in CKO compared to WT (average 5.2 x 10^6 (WT) vs. 2.0 x 10^6 (CKO), p=0.017, WT n=5, CKO n=6, Mann Whitney test). Scale bar 100 µm. (S) WT mice with EAE treatment with daily intraperitoneal injection of a JAM-A blocking peptide (JAM-Ap) from day 7 post-immunization were protected against clinical disability compared to scramble peptide treated controls (day 13-15, p<0.0001, day 16-17, p<0.05, day 20-21, p<0.05; graph shows pooled data from 3 independent EAE experiments with a minimum of 8 mice per group for each experiment; total JAM-Ap n=24, scramble n=26, two-way ANOVA with Bonferroni correction).

Supplemental Figure 1

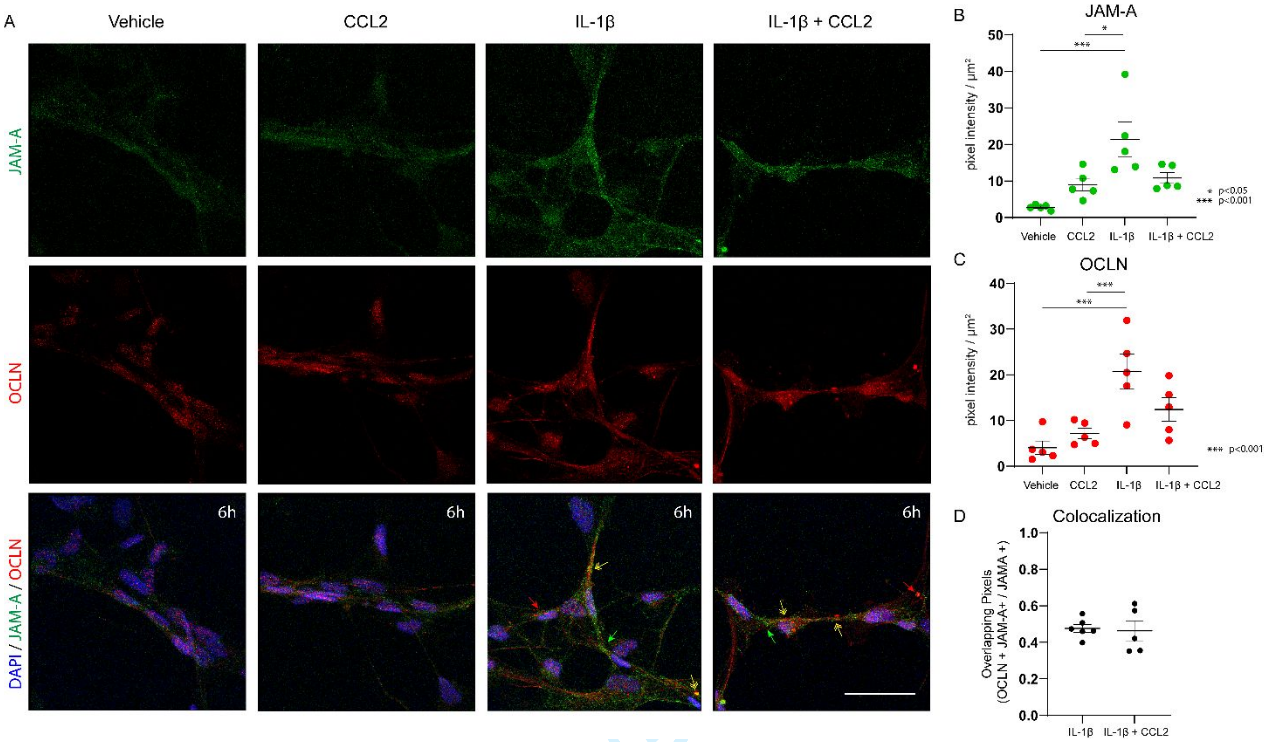

For Peer Review Only

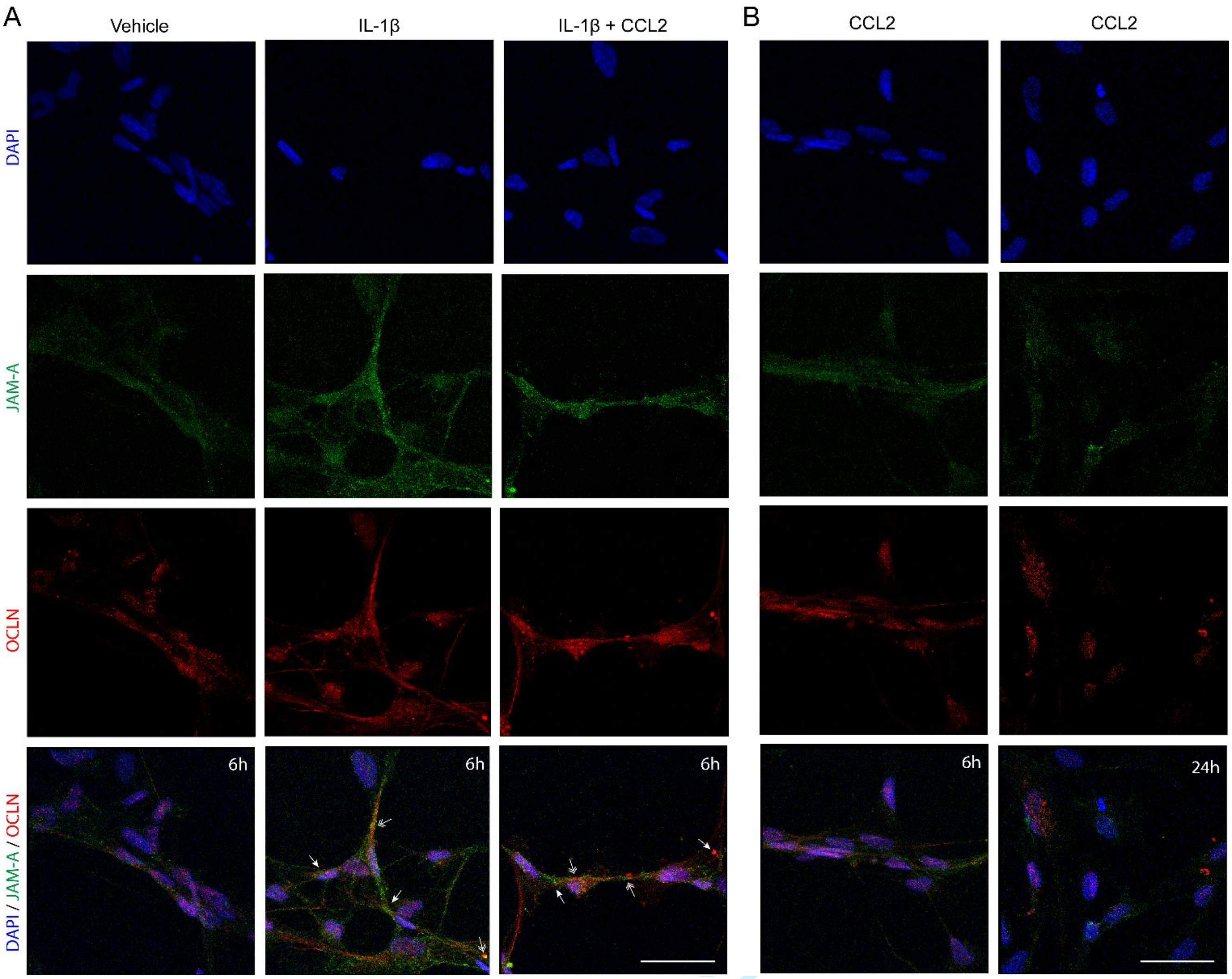
A
Vehicle
IL-1β
IL-1β + CCL2
B
CCL2
CCL2
DAPI
JAM-A
OCLN
DAPI / JAM-A / OCLN
6h
6h
6h
6h
24h

Supplemental Figure 1: Spatial ~~and temporal~~ dynamics of astrocytic JAM-A and OCLN induction *in vitro* at 6 hours. (A-B) Astrocytic JAM-A (green) ~~induction~~ was induced *in vitro* ~~is detected~~ at 6 hours after treatment with 20ng/~~ml~~ mL IL-1β alone but not after treatment with 100ng/mL CCL2 or the combination of IL-1β and CCL2 (average vehicle (2.826) vs. IL-1β (21.34) vs. CCL2 (9.015) vs. IL-1β + CCL2 (10.83), vehicle vs. IL-1β: p=0.0007, CCL-2 vs. IL-1β: p=0.02, other comparisons p>0.05, analyzed images n=5 all groups, one way ANOVA with Tukey's multiple comparison test). JAM-A ~~shows~~ showed a similar localization pattern as seen at 24 hours **(Figure 1A-E)**: ~~in which it is~~ both diffusely localized throughout the cell membrane (~~white~~ green arrows) and co-localized with the tight junction marker, occludin (OCLN, red, red arrows; ~~white double headed~~yellow arrows pointing to areas of JAM-A$^+$ and OCLN$^+$ overlapping pixels~~y of the two proteins in yellow)~~. All results were quantified from at least 4 fields of view from two to three technical replicates per group. Scale bar 50 µm. (~~B~~C) ~~Addition of CCL-2 did not change the distribution of JAM-A as has been previously demonstrated in CNS vascular endothelium. Scale bar 50 µm. Results were confirmed in at least 3 fields of view on two to three technical replicates in each group.~~Astrocytic occludin was similarly induced at 6 hours after treatment with IL-1β but not CCL2 or the combination of IL-1β and CCL2 (average vehicle (4.058) vs. IL-1β (20.76) vs. CCL2 (7.137) vs. IL-1β + CCL2 (12.41), vehicle vs. IL-1β: p=0.001, CCL-2 vs. IL-1β: p=0.0062, other comparisons p>0.05, n=5 all groups, one way ANOVA with Tukey's multiple comparison test). (D) The addition of CCL2 to IL-1β did not change the proportion of JAM-A$^+$ pixels colocalized with OCN$^+$ pixels at 6 hours. (IL-1β (0.476) vs. IL-1β + CCL2 (0.4632), p=0.81, analyzed images n=6 IL-1β, n=5 IL-1β + CCL2, unpaired two-tailed t-test.

For Review Only

Supplemental Figure 2

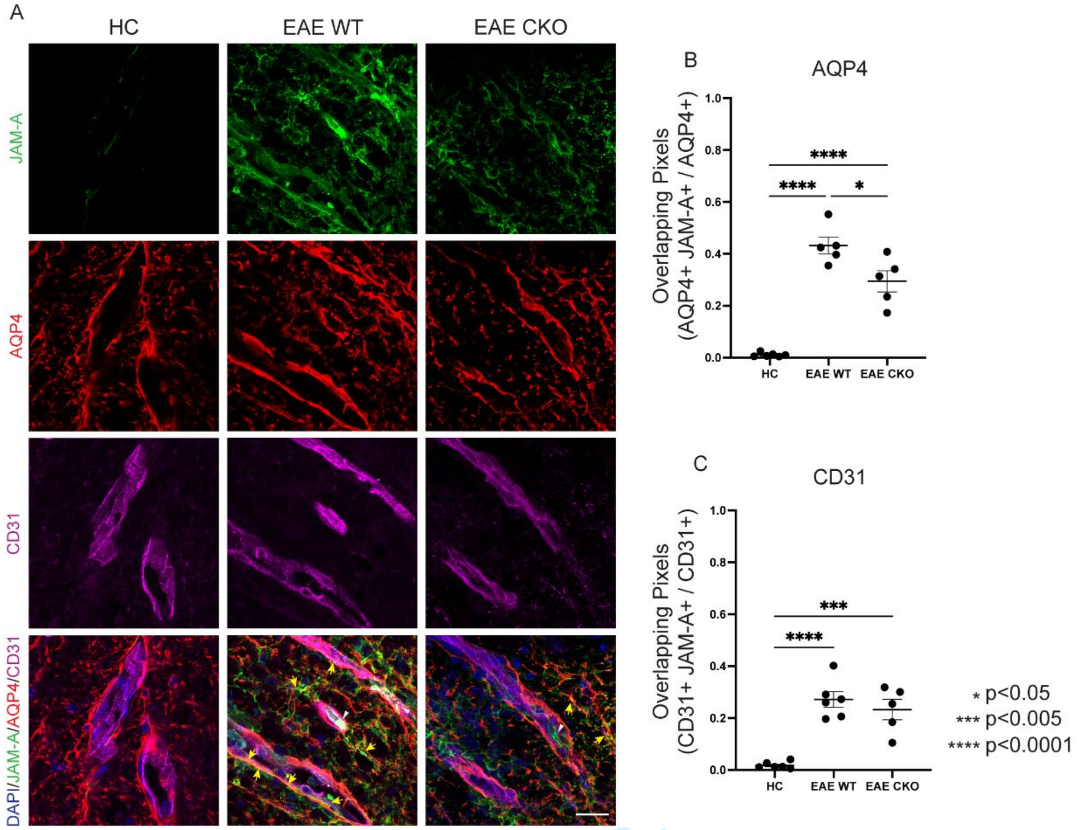

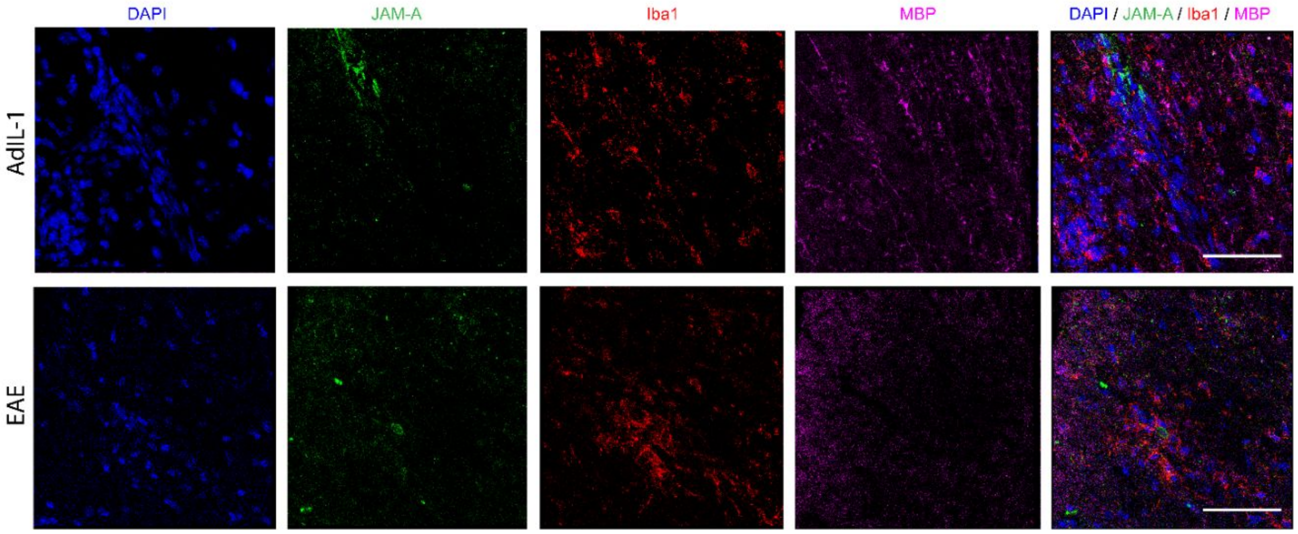

Supplemental Figure 2: JAM-A is induced in both endothelial cells and the astrocytic endfeet during EAE. (A) Images show immunohistochemistries for JAM-A (green), AQP4 (red, marker of astrocytic endfeet), CD31 (purple, marker of endothelial cells), and DAPI (blue) in the spinal cord of healthy control (HC), and WT and CKO mice with EAE 5 days from disease onset (EAE WT and EAE CKO, respectively). Colocalization of JAM-A with AQP4 (yellow arrows) and CD31 (white arrowheads) is observed in the spinal cord of mice with EAE but not in HCs. Scale bar = 20 μm. (B) Proportion of $AQP4^+$ pixels colocalizing with JAM-A^+ pixels demonstrate JAM-A expression in astrocytic endfeet (AQP4+) during EAE (HC vs EAE WT and HC vs EAE CKO: $p < 0.0001$), which is decreased in EAE CKO compared to EAE WT (EAE WT vs EAE CKO: $p = 0.0139$). (C) Proportion of endothelial cells ($CD31^+$) positive for JAM-A is increased during EAE compared to HCs (HC vs EAE WT, $p < 0.0001$; HC vs EAE CKO, $p = 0.002$). No differences in the proportion of JAM-A^+ and $CD31^+$ overlapping pixels were observed between EAE WT and EAE CKO mice ($p > 0.05$). Analyses in (B-C) one way ANOVA with Tukey's multiple comparison test. HC, n=6; EAE WT, n=5; EAE CKO, n=5. ~~Reactive astrocytic JAM-A is not expressed in microglia or oligodendrocytes in vivo. JAM-A expression in AdIL-1 cortical lesions and EAE spinal cord lesions does not co-localize with microglial marker Iba1 or oligodendrocyte marker MBP. Scale bar 50μm.~~

For Review Only

Supplemental Figure 3

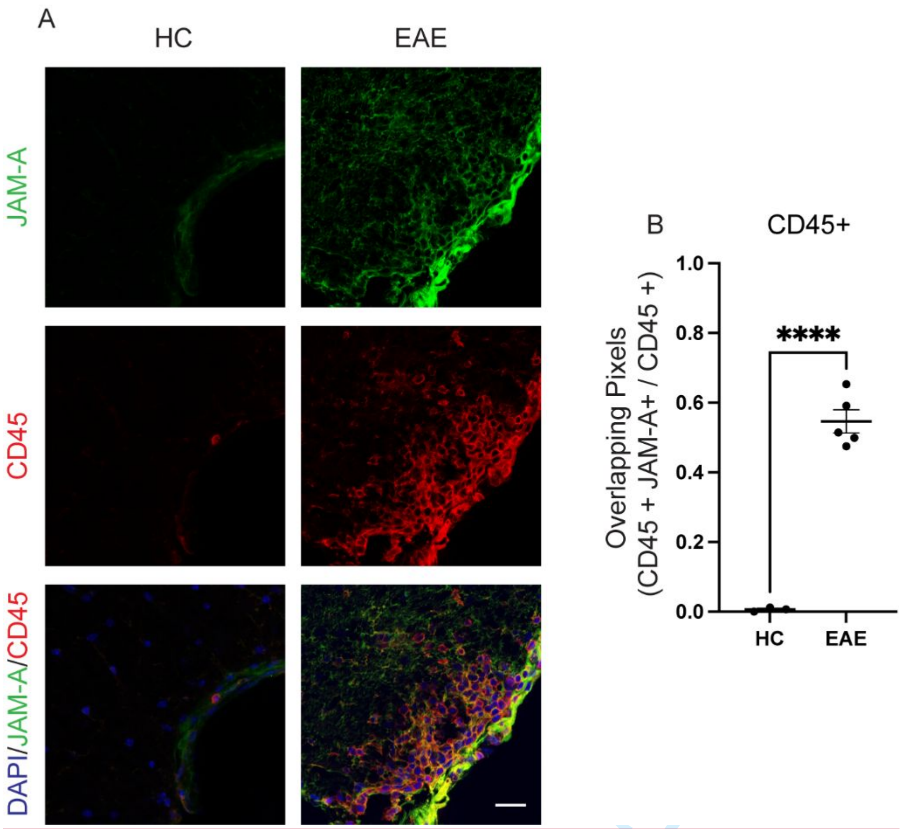

w Only

Supplemental Figure 3: JAM-A is expressed by CD45+ immune cells infiltrating the spinal cord of mice with EAE. (A) Immunohistochemistry for JAM-A (green), CD45 (red, a marker of immune cells), and DAPI (blue) in the spinal cord dorsal column of mice with EAE 5 days from disease onset and in healthy controls (HC). Scale bar = 20 µm. (B) Colocalization analysis of CD45 and JAM-A shows that the proportion of $CD45^+$ pixels overlapping with JAM-A^+ pixels increases in EAE mice compared to HC mice ($p<0.0001$, unpaired two-tailed t-test). HC, n=3; EAE, n=5.

For Review Only

Supplemental Figure 4

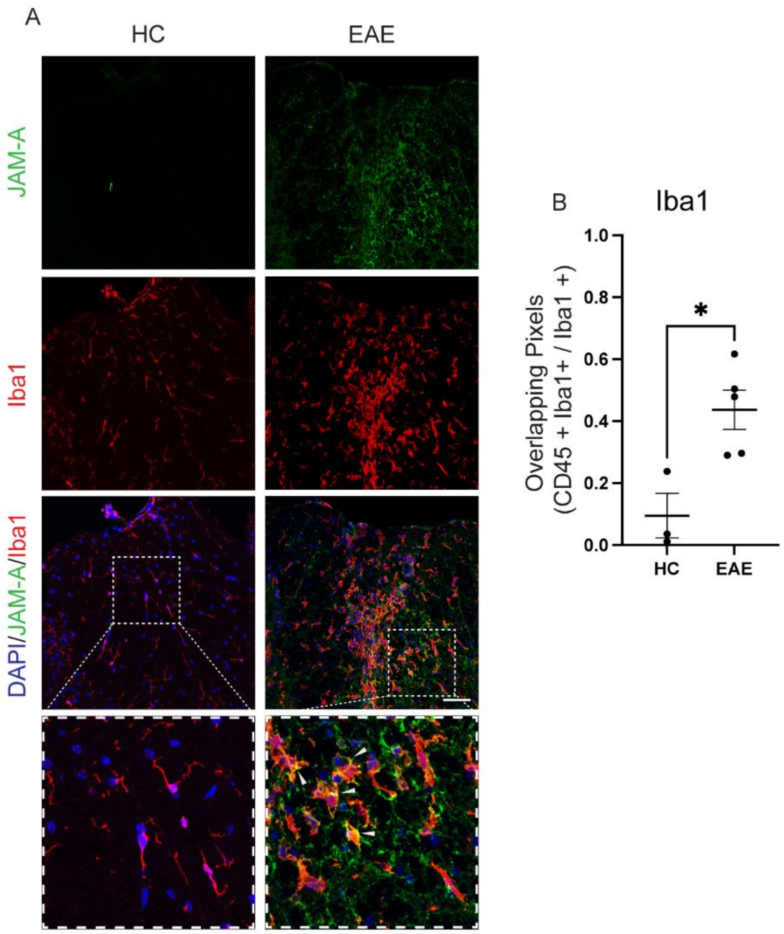

nly

Supplemental Figure 4: JAM-A is expressed by Iba^{+} microglia in the spinal cord of mice with EAE. (A) Images show immunohistochemistries for JAM-A (green), Iba1(red, marker of microglia), and DAPI (blue) in the spinal cord dorsal column of mice with EAE and healthy controls (HC). JAM-A expression is absent in the spinal cord of HC mice, while it is upregulated in mice with EAE. Iba1 positive microglia express JAM-A in areas of inflammation in the spinal cord of mice with EAE (white arrowheads). Scale bar = 50 µm. (B) Colocalization analysis of Iba1 with JAM-A shows that the proportion of Iba1^{+} pixels overlapping with JAM-A^{+} pixels increases in EAE mice compared to HC mice (p=0.0138, unpaired t-test). HC, n=3; EAE, n=5.

For Review Only

Supplemental Figure 5

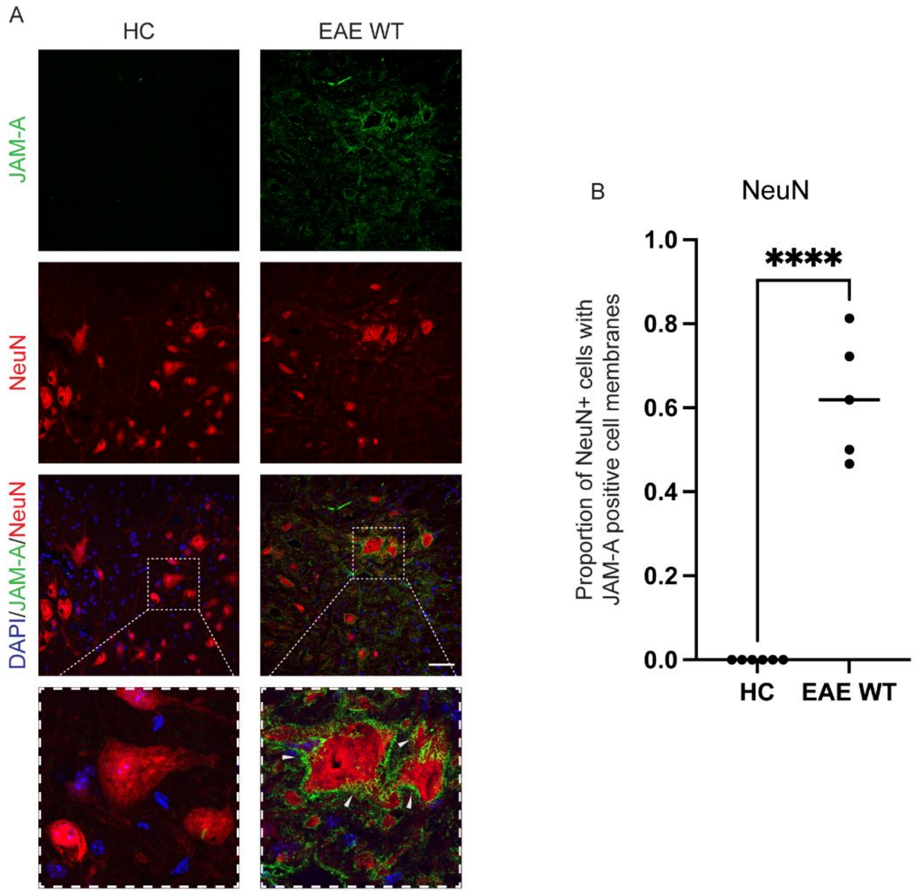

Only

For Review Only

Supplemental Figure 5: Spinal neurons express JAM-A during EAE. (A) Images show immunohistochemistries for JAM-A (green), NeuN (red, marker of neurons' cell body), and DAPI (blue) in the spinal cord ventral horn of healthy control (HC) and WT mice at 5 days from EAE disease onset (EAE WT). JAM-A expression is minimally detectable in the spinal cord of HC mice, while it is upregulated in EAE and expressed on the cell surface of neurons of the spinal cord ventral horn (white arrowheads). Scale bar = 50 µm. (B) Quantification of the number of JAM-A^{+} neurons relative to the total number of neurons (NeuN^{+}) in HC and EAE WT mice showed increases in EAE WT compared to HC ($p<0.0001$, unpaired two-tailed t-test). HC, n=6; EAE WT, n=5.

For Review Only

Supplemental Figure 6

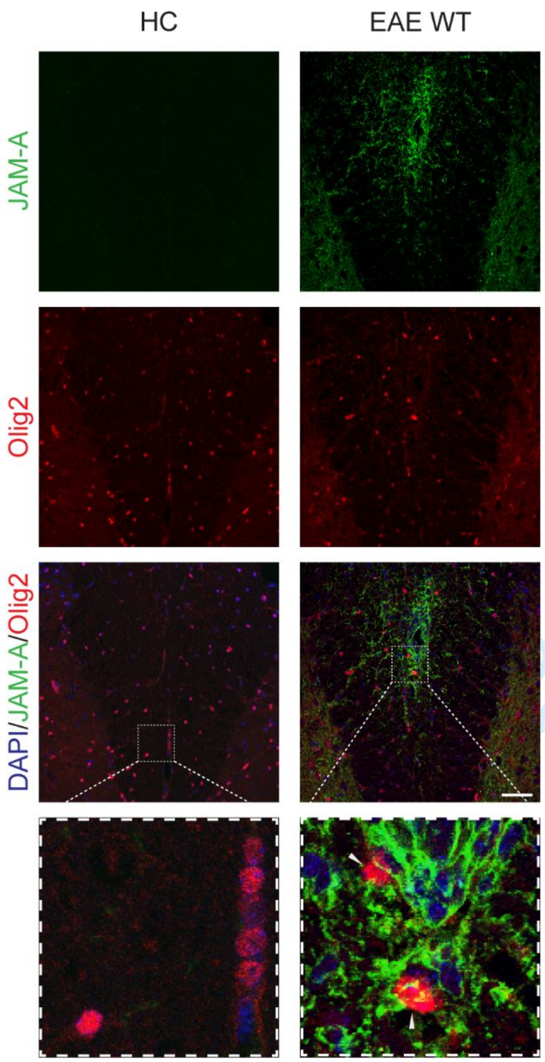

For Review Only

Supplemental Figure 6: JAM-A expression is not clearly detected in oligodendrocytes on immunohistochemistry during EAE. (A) Images show immunohistochemistries for JAM-A (green), Olig2 (red, marker of oligodendrocytes), and DAPI (blue) in the spinal cord dorsal column of healthy control (HC), and WT mice at 5 days from EAE disease onset (EAE WT). JAM-A expression is absent in the spinal cord of HC mice, while it is upregulated in EAE and expression appears to be separated from the cell surface of the few oligodendrocytes within inflammatory lesions (white arrowheads) in EAE mice. More sensitive techniques, such as cell sorting, may be needed to determine whether oligodendrocytes express JAM-A. Scale bar = 50 μm.

For Review Only

Supplemental Figure 7

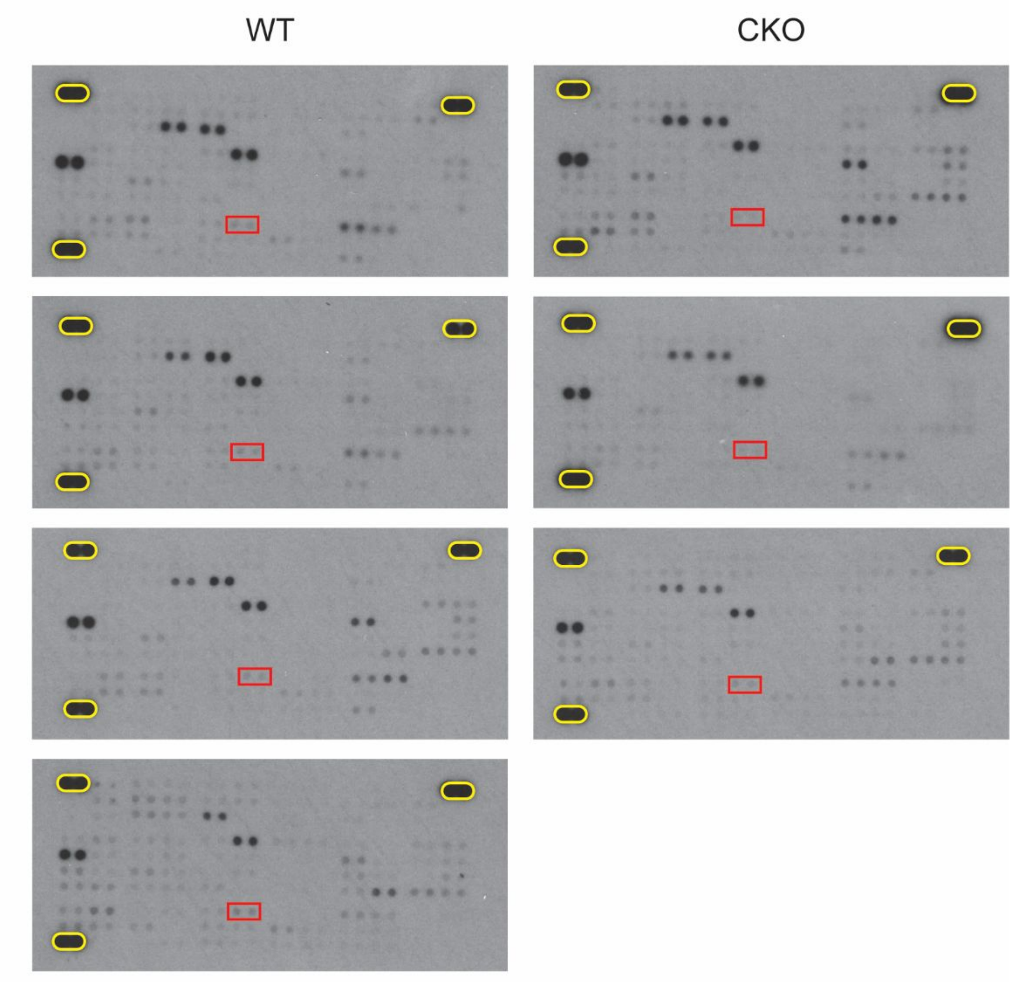

Supplemental Figure 7: Differential expression of MMP-2 is detected on proteome arrays of spinal cord lysates from JAM-A CKO and WT mice during EAE. Images show individual ELISA immunoarrays (WT, n=4; cKO, n=3) assessing the expression of 111 cytokines, chemokines, proteases and acute phase reactants in the spinal cord of mice at 5 days from EAE disease onset. Red rectangles highlight MMP-2 expression which was significantly decreased in CKO mice compared with WT controls, while yellow ovals demarcate the reference spots in each

array. A minimal linear contrast enhancement step was performed uniformly across all original blot images prior to analysis. Original blot images are available in the Supplemental Materials.

For Review Only

Astrocytic JAM-A Regulates T Cell Entry Past the Glia Limitans to Promote CNS Autoimmune Attack

Mario Amatruda PhD,[1*] Candice Chapouly PhD,[2*] Viola Woo BS,[1] Farinaz Safavi MD PhD,[3] Joy Zhang BS,[4] David Dai BA,[5] Anthony Therattil BS,[6] Chang Moon BA,[1] Alexandra Gordon BA,[7] Charles Parkos MD,[8] and Sam Horng MD PhD[1,9] *Co-first authors

[1] Icahn School of Medicine at Mount Sinai, Dept of Neurology, NY, NY, USA

[2] Univ. Bordeaux, Inserm, Biology of Cardiovascular Diseases, U1034, CHU de Bordeaux, F-33604 Pessac, France

[3] National Institute of Neurological Disorders and Stroke, National Institutes of Health, Bethesda, MD, USA

[4] University of Virginia School of Medicine, Charlottesville, VA, USA

[5] Perelman School of Medicine at the University of Pennsylvania, Dept of Neurology, Philadelphia, PA, USA

[6] New York Medical College, Valhalla, NY, USA

[7] Miller School of Medicine at University of Miami, Miami, FL, USA

[8] University of Michigan, Dept of Pathology, Ann Arbor, MI, USA

[9] Icahn School of Medicine at Mount Sinai, Dept of Neuroscience, NY, NY, USA

Corresponding Author:

Sam Horng MD PhD

Icahn School of Medicine at Mount Sinai

Icahn 10-20A

1468 Madison Avenue

New York, NY 10029

Email: sam.horng@mssm.edu

(212) 659-1692

For Review Only

Graphical Abstract:

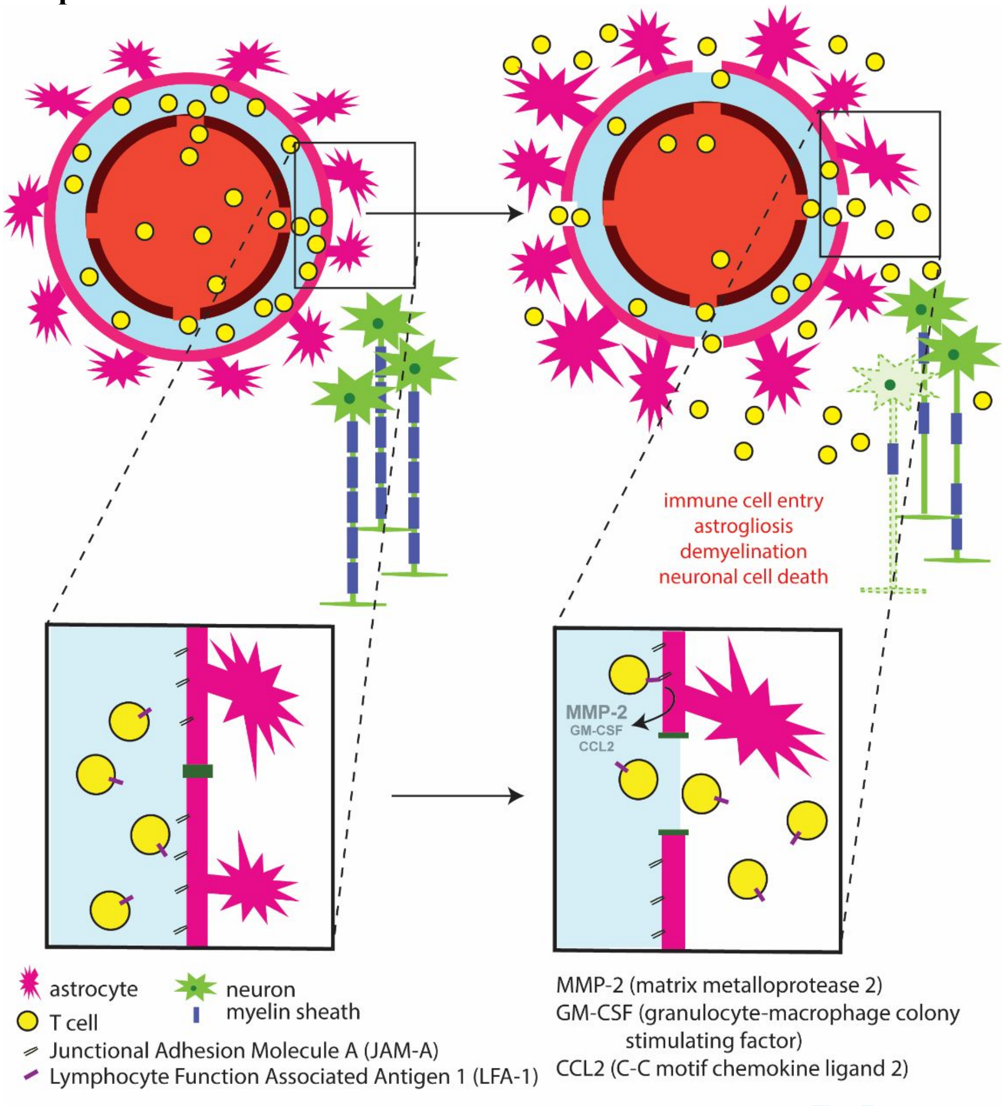

Abbreviated Summary:

Amatruda et al. report that the astrocytic immune cell receptor, Junctional Adhesion Molecule-A (JAM-A) promotes T cell entry into the CNS during autoimmune attack via the production of matrix metalloprotease 2 (MMP-2). Blocking contact-mediated astrocyte immune cell signals represents a novel therapeutic approach against multiple sclerosis and other CNS autoinflammatory diseases.

Abstract: Contact mediated interactions between the astrocytic endfeet and infiltrating immune cells within the perivascular space are underexplored, yet represent potential regulatory check-points against CNS autoimmune disease and disability. Reactive astrocytes upregulate Junctional Adhesion Molecule-A (JAM-A), an immunoglobulin-like cell surface receptor that binds to T cells via its ligand, the integrin, lymphocyte function-associated antigen-1 (LFA-1). Here, we tested the role of astrocytic JAM-A in regulating CNS autoinflammatory disease. In cell co-cultures, we found that JAM-A mediated signaling between astrocytes and T cells increases levels of MMP-2, CCL-2 and GM-CSF, proinflammatory factors driving lymphocyte entry and pathogenicity in multiple sclerosis (MS) and experimental autoimmune encephalomyelitis (EAE), an animal model of CNS autoimmune disease. In EAE, mice with astrocyte-specific *JAM-A* deletion (*mGFAP:CreJAM-A*$^{fl/fl}$) exhibit decreased levels of MMP2, a failure of T cells to infiltrate the CNS parenchyma from the perivascular spaces (PVS), and a milder histopathological and clinical course of disease compared to wild-type controls (*JAM-A*$^{fl/fl}$). Treatment of wild-type mice with intraperitoneal injection of soluble JAM-A blocking peptide (JAM-Ap) protects against EAE, highlighting the potential of contact mediated astrocyte-immune cell signaling as a novel translational target against neuroinflammatory disease.

For Review Only

Introduction:

In multiple sclerosis (MS) and other autoimmune diseases of the central nervous system (CNS), immune cells inappropriately invade the CNS from the bloodstream and drive inflammatory lesion formation (Frischer *et al.*, 2015; Lassmann, 2018). CNS entry is a two-step process through a specialized structure termed the neurovascular unit (NVU): first, immune cells cross the

endothelial blood-brain barrier, using contact-mediated interactions with the endothelial surface to traffic into an intermediary compartment termed the perivascular space (PVS) (Abbott *et al.*, 2006; Engelhardt and Ransohoff, 2012; Schlager *et al.*, 2016). Within the PVS, immune cells encounter the glia limitans (GL), a barrier comprised of astrocytic endfeet through which cells must subsequently cross to access the CNS parenchyma and inflict damage (Owens *et al.*, 2008).

Interactions between the astrocytic endfeet and immune cells within the PVSs have been minimally explored despite their potential significance in regulating the autoimmune response. Cross-talk is known to involve leukocyte matrix metalloproteases (MMP-2 and MMP-9) in degrading PVS basement membranes, enabling the parenchymal entry of infiltrating immune cells during EAE (Song *et al.*, 2013; Song *et al.*, 2015; Gerwien *et al.*, 2016). Astrocytic VCAM-1 has also been identified as a TNFR1-induced cell adhesion molecule critical for immune cell trafficking past the GL and into the CNS parenchyma during EAE (Gimenez *et al.*, 2004; Gimenez *et al.*, 2006; Laureys *et al.*, 2014). Moreover, astrocytic VCAM-1 is modulated in a region-specific manner by effector T cell (Th1 and Th17) secreted factors accounting for regional differences in immune cell infiltration during EAE (Williams *et al.*, 2020). Recently, tissue resident CD8 T cells were found within multiple sclerosis lesions to express PD-1 while reactive astrocyte endfeet express PD-1 ligand, suggestive of potential inhibitory interactions within the PVS (Smolders *et al.*, 2018). Therefore, both soluble and contact-mediated signals between astrocytes and immune cells within the PVS may play a significant role in regulating CNS autoinflammatory disease.

We previously reported (Horng *et al.*, 2017)that reactive astrocytes upregulate Junctional Adhesion Molecule-A (JAM-A), an immunoglobulin-like cell-surface receptor, in response to the

For Review Only

proinflammatory cytokine, interleukin-1 beta (IL-1β) *in vitro* as well as in *in vivo* models of CNS inflammation. JAM-A has a dual role: 1) initiating and stabilizing tight junction complexes via homophilic binding between identical cell types (Weber *et al.*, 2007; Luissint *et al.*, 2014; Kummer and Ebnet, 2018) and 2) serving as an immune cell surface receptor via heterophilic binding to LFA-1 on T cells and monocytes (Nourshargh *et al.*, 2006). In gut and CNS vascular endothelium, JAM-A binds to immune cells to induce intracellular signal transduction pathways and promote transmigration through the endothelial layer, ultimately leading to a pro-inflammatory, tissue damaging state (Engelhardt and Ransohoff, 2012; Lakshmi *et al.*, 2012; Schmitt *et al.*, 2014; Sladojevic *et al.*, 2014; Flemming *et al.*, 2018; Fan *et al.*, 2019; Luissint *et al.*, 2019).

We hypothesized that astrocytic JAM-A interacts with immune cells within the PVS to promote effector pathways of CNS inflammation and tissue damage. Here, we focused on the T cell population given its central role in driving pathogenesis of EAE, an animal model of CNS autoimmune demyelinating disease. We first characterized the effects of astrocytic JAM-A on protease and cytokines implicated in EAE and MS pathogenesis. Then, using a genetic mouse model in which JAM-A is selectively deleted from reactive astrocytes ($mGFAP{:}CreJAM\text{-}A^{fl/fl}$) compared to unaffected ($JAM\text{-}A^{fl/fl}$) controls, we investigated how astrocytic JAM-A regulates lesion pathology in two models of CNS inflammation and its effects on T cell trafficking, the inflammatory proteome and clinical disability in EAE (Cera *et al.*, 2004; Garcia *et al.*, 2004).Additionally, we tested the therapeutic potential of an exogenously administered JAM-A blocking peptide in EAE.

For Review Only

Results:

Astrocytic JAM-A is upregulated diffusely on the astrocytic cell surface in response to interleukin-1β *in vitro* and in *in vivo* models of CNS inflammatory disease

We reported previously that reactive astrocytes upregulate the tight junction proteins, Claudin-1 (Cldn-1), Claudin-4 (Cldn-4) and JAM-A in response to CNS inflammation (Horng *et al.*, 2017). In CNS vascular and gut endothelial cells, JAM-A acts both as a tight junction molecule in trans dimeric form and as an immune cell receptor in monomeric form (Ebnet *et al.*, 2004; Wojcikiewicz *et al.*, 2009; Stamatovic *et al.*, 2012). In vascular endothelium, the cytokine CCL-2 serves as a switch, causing JAM-A internalization from the tight junction and relocalization to the cell surface as a monomer (Stamatovic *et al.*, 2012; Sladojevic *et al.*, 2014).

Using human astrocyte cultures, we confirmed that treatment with IL-1β, but not CCL2 alone, induced expression of JAM-A and the tight junction protein occludin by 6 and 24 hours (**Figure 1A, B, Supplemental Figure 1A,B**). Upon induction, astrocytic JAM-A not only co-localized with the tight junction protein occludin but was also distributed more diffusely throughout the cell membrane (**Figure 1C, Supplemental Figure 1C**). Combined treatment with IL-1β and CCL-2 did not augment or change the colocalization of JAM-A with the tight junction protein occludin, compared to IL-1β alone (**Figure 1C, Supplemental Figure 1C**). Therefore, astrocytic JAM-A is found both in overlap with the tight junction and apart from it after induction by IL-1β and its distribution apart from the tight junction does not appear to change with the addition of CCL-2.

For Review Only

We characterized the expression of astrocytic JAM-A in two models of CNS inflammation in the mouse. In resting (ie. healthy) cortex, JAM-A was not expressed in astrocytes of the CNS parenchyma (identified using GFAP staining) (**Figure 1F**). It was most strongly detected in a pattern matching that of the vascular endothelium, consistent with previous studies (**Figure 1F**) (Padden *et al.*, 2007; Stamatovic *et al.*, 2012; Sladojevic *et al.*, 2014; Bhowmick *et al.*, 2019). In asymptomatic inflammatory lesions produced by intracortical injections of IL-1β expressing adenovirus (AdIL-1), reactive astrocytes expressed JAM-A, most prominently within the astrocytic processes known to encircle blood vessels, as identified using aquaporin-4 (AQP4) staining (**Figure 1G, supplemental Figure 2A, B**). In healthy spinal cord, astrocytic JAM-A was largely undetectable (**Figure 1H, Supplemental Figure 2A,B**). In inflammatory demyelinating spinal cord lesions of EAE, JAM-A was expressed in astrocytes, particularly in the AQP4 positive endfeet of the perivascular astrocytes (**Figure 1H,I, Supplemental Figure 2A,B**), as well as on infiltrating leukocytes (**Figure 1H, Supplemental Figure 3**) and additional CNS resident cell types within EAE lesions. JAM-A was present at low levels on blood vessels ($CD31^+$) (**Supplemental Figure 2A, C**), robustly expressed by microglial cells ($Iba1^+$), being enriched within areas of inflammation (**Supplemental Figure 4**) and on the surface of ventral horn neurons ($NeuN^+$) (**Supplemental Figure 5**). Oligodendrocytes ($Olig2^+$) did not show clear overlap with JAM-A staining. (**Supplemental Figure 6**).. Astrocyte specific knock-down of JAM-A was demonstrated in EAE lesions, but not in healthy cortex and spinal cord, of conditional knock-out (*mGFAP:CreJAM-A$^{fl/fl}$*, CKO) mice compared to littermate wild-type (*JAM-A$^{fl/fl}$*, WT) controls (**Figure 1H-J, Supplemental Figure 2A, B**). CKO mice showed decreases of JAM-A expression within astrocytes (GFAP), including the endfeet (AQP4), but not the endothelium (CD31) during EAE (**Figure 1I, J, Supplemental Figure 2A-C**).

For Review Only

Astrocytic JAM-A increases levels of pro-inflammatory cytokines and proteases critical for CNS autoinflammatory disease

Local protease and cytokine levels within the PVS play a critical role in facilitating immune cell priming, CNS entry and autoimmune attack (Song *et al.*, 2015; Williams *et al.*, 2020). We tested whether astrocytic JAM-A leads to changes in protease and cytokine levels in an astrocyte-T cell co-culture system. Here, we used a pan-T cell population ($CD3^+$) to assess the net effects of astrocytic JAM-A signaling to both $CD4^+$ and $CD8^+$ T cells. ELISA arrays were performed on co-cultures of activated (IL-1β treated) human astrocytes and $CD3^+$ T cells in the presence or absence of astrocytic JAM-A. Efficacy of siRNA mediated knock-down of JAMA (*siJAM-A*) in comparison to a non-targeting siRNA (*siNT*) was demonstrated previously (Horng *et al.*, 2017). Supernatants extracted from co-cultures with *siJAM-A* transfected astrocytes showed statistically significant decreases in MMP-2 (**Figure 2A**) and GM-CSF (**Figure 2D**), both factors previously shown to promote EAE pathogenesis (dos Santos *et al.*, 2005; Agrawal *et al.*, 2006; Kroenke *et al.*, 2010; Rasouli *et al.*, 2015; Song *et al.*, 2015; Gerwien *et al.*, 2016; Levesque *et al.*, 2016; Ifergan *et al.*, 2017; Imitola *et al.*, 2018; Galli *et al.*, 2019; Monaghan and Wan, 2020; Wheeler *et al.*, 2020). Lysates of *siJAM-A* transfected astrocytes showed decreased levels of ADAM9, cathepsin C and CCL-2 (**Figures 2B, E**), the last of which is known to promote EAE pathogenesis via its chemotactic effects on infiltrating monocytes (Ge *et al.*, 2012; Kim *et al.*, 2014). Lysates of $CD3^+$ T cells showed no statistically significant protease or cytokine changes (**Figures 2C, F**). In sum, in astrocyte-T cell co-cultures subjected to pro-inflammatory conditioning of both cell types, astrocytic JAM-A lead to increased levels of MMP-2, CCL-2 and GM-CSF, EAE promoting signals involved in both immune cell infiltration into the CNS

parenchyma and pathogenic T cell activity.

Astrocytic JAM-A regulates immune cell infiltration past the neurovascular unit (NVU) in IL-1β induced cortical lesions

To test how astrocytic JAM-A controls immune cell trafficking through the neurovascular unit (NVU), we characterized patterns of immune cell entry in asymptomatic cortical lesions induced by intracortical AdIL-1 injection. Lesion size after AdIL-1 injection, measured as area of neuronal loss, showed a decreasing trend not reaching statistical significance in *mGFAP:CreJAM-A$^{fl/fl}$* (CKO) mice compared to *JAM-A$^{fl/fl}$* (WT) mice (**Figures 3A, B**). Lesions in CKO mice demonstrated on average more $CD4^+$ immune cells than the WT group (**Figures 3C, D**). However, $CD4^+$ immune cells in CKOs were restricted to the NVU, as demarcated by pan-laminin staining which labels the basement membranes of the NVU (**Figures 3C, E, F**). By contrast, in WT mice, the majority of $CD4^+$ cells were located in the parenchyma, indicating successful migration out of the NVU (**Figures 3C, E, G**). Therefore, astrocytic JAM-A facilitated $CD4^+$ immune cell infiltration past the glia limitans and into the CNS parenchyma in cortical AdIL-1induced lesions.

Astrocytic JAM-A promotes EAE disease severity and tissue damage via the regulation of MMP-2 levels and T cell trafficking out of the perivascular spaces (PVS)

To test the role of astrocytic JAM-A in a model of CNS autoimmune demyelinating disease, EAE was induced in *JAM-A$^{fl/fl}$* (WT), *mGFAP:CreJAM-A$^{fl/fl}$* (CKO), and *JAM-A$^{-/-}$* (KO) mice. CKO and KO mice were studied to differentiate effects of astrocytic JAM-A loss from total JAM-A deletion. CKO and

For Review Only

KO mice both showed statistically significant milder courses of disease (**Figure 4A**), including lower average (**Figure 4B**), peak (**Figure 4C**) and cumulative (**Figure 4D**) disease scores at Day 28 post-immunization compared to WT mice.. Survival curves showed statistically significant differences in mortality, but not disease induction between WT and the CKO and KO mice (**Figures 4E, 4G**). Rates of mortality at Day 28 post-immunization were significantly reduced in both CKO and KO mice (**Figure 4F**) while rates of resistance to disease induction showed a greater, non-statistically significant trend in CKO and KO mice compared to WTs (**Figure 4H**). To confirm the translational potential of JAM-A blockade, WT mice with EAE were treated with daily intraperitoneal injection starting at Day 7 post-immunization of either a soluble JAM-A blocking peptide (JAM-Ap) specifically targeting the monomeric form or a scramble non-targeting peptide. Treatment with JAM-Ap demonstrated a protective effect against clinical disability in EAE compared to the scramble control (**Figure 4I**).

The course and severity of disease did not differ between CKO and KO mice, suggesting that the protective effect of JAM-A blockade may be fully attributed to astrocytic JAM-A. To eliminate confounding mechanisms of JAM-A deletion in other tissues and cell types, we decided to focus on neuropathology in the CKO line. Immunohistopathology was performed in *mGFAP:CreJAM-A$^{fl/fl}$* (CKO) and *JAM-A$^{fl/fl}$* (WT) mice to measure first patterns of immune cell infiltration into spinal cord lesions. At 5 days from EAE onset, when immune cells are most exponentially infiltrating the CNS (Barthelmes *et al.*, 2016), immune cells were found to be diffusely distributed throughout the CNS parenchyma in WT mice but accumulated within the perivascular spaces (PVS) between the astrocytic endfeet (AQP4) and blood vessel wall (CD31) in CKO mice (Figure 5A). Subsequently at day 21 post EAE immunization, WT mice continued to show CD4^{+} and CD45^{+} immune cells throughout the CNS parenchyma while CKOs demonstrated ongoing arrest, or cuffing, of cells within the laminin-rich

For Review Only

perivascular spaces (PVS) and limited infiltration of cells into the parenchyma (**Figures 5B-D**).

To establish a mechanistic link between astrocytic JAM-A signaling and T cell infiltration *in vivo*, a proteome profiler probing 111 soluble mouse proteins, including cytokines, chemokines, proteases, growth factors and acute phase signals was used to compare the proteomic patterns of spinal cord tissues from WT and CKO mice at 5 days from EAE disease onset. Of 111 probes, MMP-2, which had shown strong astrocytic-JAM-A dependent regulation *in vitro*, demonstrated the highest fold change *in vivo* and was the sole factor with a statistically significant difference in expression between WT and CKO mice (**Figure 5E-G**).

To test whether the absence of astrocytic JAM-A and PVS cuffing altered the total number of $CD3^+$ and $CD4^+$ T cells entering the CNS during EAE, flow cytometry was performed in CKOs and WTs at 5 days from EAE disease onset during the ascending phase of disease. No difference in total $CD3^+$ T cell number was found in the spinal cord (**Figures 5H, I**) or spleen of CKO mice compared to WTs (**Figure 5L, M**). In the spinal cord, $CD3^+CD4^+$ T cell counts showed a decreasing trend not reaching statistical significance in CKOs compared to WTs (**Figure 5J, K**). In the spleen, $CD3^+CD4^+$ counts were not significantly different between groups (**Figure 5N, O**). Therefore, astrocytic JAM-A deletion affected the spatial distribution but not total number of T cells within the CNS during EAE.

Immunohistochemical analysis in healthy control (HC) spinal cord tissues, at 5 days from EAE disease onset and at 28 days post-immunization demonstrated that CKO mice were protected against neuropathological damage of EAE. Throughout EAE, CKO mice maintained levels of anterolateral tract flouromyelin staining that were higher than WT and comparable to HCs (**Figures 6A, B**). EAE CKO mice also showed increased numbers of NeuN positive cells (neurons) within the ventral horn of the lumbar spinal cord to HC compared with time-matched (at day 5 from EAE onset and at Day 28

For Review Only

post immunization) WT controls and, in EAE CKO mice at Day 28 post immunization, the number of NeuN$^+$ cells was comparable to that observed in HC (**Figures 6C, D)**). Finally, EAE CKO mice also had similar levels of GFAP signals in the white matter of the lumbar spinal cord compared to HC while WT mice with EAE at 28 days post immunization showed significant increases in GFAP signal, suggesting greater astrogliosis (**Figures 6E, F**). Collectively, these histopathologic changes reflect a milder course of the disease which resembles other genetic models (Korner *et al.*, 1997; Song *et al.*, 2015) in which immune cell trapping within the PVSs prevents parenchymal damage and clinical disability.

For Review Only

Discussion:

The glia limitans (GL) is the final barrier separating peripheral infiltrating immune cells and soluble factors from the CNS parenchyma (Abbott *et al.*, 2006; Engelhardt and Coisne, 2011). The perivascular spaces (PVSs) therefore represent the penultimate compartment for incoming cells and factors during CNS autoinflammatory disease. Contact-mediated signals between the astrocytic end-feet of the GL and immune cells have the potential to act as critical checkpoints for both 1) the entry of inflammatory cells into the CNS parenchyma from the PVS and 2) the functional differentiation of both cell types in the inflammatory context (De Keyser *et al.*, 2010; Sofroniew, 2015; Prajeeth *et al.*, 2017; Liddelow and Sofroniew, 2019; Williams *et al.*, 2020). Here, we demonstrate a novel role for the astrocyte cell signaling receptor, JAM-A, in controlling via MMP-2 lymphocyte trafficking into the CNS parenchyma promoting downstream effects of histopathological damage and clinical disability. Experiments using functional gene network analysis to characterize how the astrocytic JAM-A mediated interaction between astrocytes and T cells regulates the functional differentiation of both cell

types are currently underway. Additional *in vivo* imaging experiments to characterize the dynamics of immune cell trafficking in the presence and absence of astrocytic JAM-A mediated signaling are also in progress.

JAM-A is an immunoglobulin-like cell surface receptor with well-characterized roles in tight junction formation, endothelial diapedesis and immune cell signal transduction in vascular, gut and lung endothelial cells (Weber *et al.*, 2007; Luissint *et al.*, 2014; Kummer and Ebnet, 2018; Hartmann *et al.*, 2020). We demonstrated that astrocytes upregulate JAM-A *in vitro* in response to IL-1β, a critical pro-inflammatory cytokine in multiple sclerosis and EAE pathogenesis, and *in vivo* during EAE and intracortical injection of AdIL-1. Previous work detailing JAM-A expression within active multiple sclerosis lesions, noted patterns within the blood brain barrier that appear to conform to upregulated expression within the astrocytic endfeet (Padden *et al.*, 2007).

Protease and cytokine ELISA experiments showed that astrocytic JAM-A increases proinflammatory effector proteins MMP-2, CCL-2 and GM-CSF in co-culture with a $CD3^+$ T cell population. These factors have previously been demonstrated to promote EAE pathogenesis and multiple sclerosis lesion formation; MMP-2 by facilitating immune cell migration out of the PVS and into the CNS parenchyma (Agrawal *et al.*, 2006; Song *et al.*, 2015; Gerwien *et al.*, 2016), CCL-2 through its chemotactic effects on infiltrating monocytes (Ge *et al.*, 2012; Kim *et al.*, 2014) and GM-CSF via its effects on monocyte recruitment and pathogenic T cell activity in the acute phase (Ponomarev *et al.*, 2007; Kroenke *et al.*, 2010; Kara *et al.*, 2015) with pleiotropic effects on tissue damage in the chronic phase (Duncker *et al.*, 2018).

For Review Only

Comparing conditional JAM-A knock out mice and controls, we found that astrocytic JAM-A promotes the entry of T cells into the CNS parenchyma in two *in vivo* models of CNS inflammation and that astrocytic deletion of JAM-A reduces clinical disability and histopathological damage during EAE. Astrocyte specific and total JAM-A deletion showed similar phenotypes suggesting that astrocytic JAM-A may fully account for its pathogenic effects in EAE, though this does not rule out the additional possibility of both pathogenic and protective effects of JAM-A in other tissues, including the intestinal epithelium and spleen. Exogenous administration of a soluble JAM-A blocking peptide protected against EAE, demonstrating a net protective effect and translational potential of blocking astrocyte-immune cell interactions during autoimmune attack.

For Review Only

Proteome ELISA arrays on spinal cord tissues at 5 days from EAE disease onset recapitulated *in vitro* findings in the EAE disease model identifying MMP-2 as a critical astrocytic JAM-A dependent factor *in vivo*. Previous work established a role for MMP-2 in promoting T cell entry into the CNS parenchyma from the perivascular spaces via several mechanisms: 1) digesting dystroglycans that anchor the astrocytic endfeet to the parenchymal basement membrane (Agrawal *et al.*, 2006), 2) activating the proinflammatory NFκB pathway in astrocytes via Notch-1 (Song *et al.*, 2015) and 3) degrading perivascular reserves of CXCL12, which promotes the retention of immune cells within the perivascular space (McCandless *et al.*, 2006). However, no statistically significant differences were found for GM-CSF and CCL2, the two other factors identified in our *in vitro* experiments. Nonetheless, it cannot be excluded that local changes in GM-CSF and CCL2 levels within the perivascular spaces were below the threshold of detection in total spinal cord lysates and may require higher resolution techniques to establish a link *in vivo*.

The extent to which T cell activation and differentiation is influenced by local signaling interactions within the PVS has yet to be determined. Experiments measuring the relative proportions of suppressor (Treg and Th2) and proinflammatory (Th1, Th17, GM-CSF secreting) helper T cell subsets in JAM-A CKOs and WTs are now underway to determine whether astrocytic JAM-A-mediated signaling has the capacity to modulate T cell differentiation patterns. Additional potential immunomodulatory players within the PVS include not only the astrocytic endfeet, but also pericytes, microglial processes, migrating oligodendrocyte precursors, basement membrane components and other circulating immune cells including dendritic cells, macrophages and B cells.

For Review Only

Astrocytes have the capacity to both promote and protect against CNS autoinflammatory disease (Cekanaviciute *et al.*, 2014; Mayo *et al.*, 2014; Anderson *et al.*, 2016; Levine *et al.*, 2016; Rothhammer *et al.*, 2016; Liddelow *et al.*, 2017; Chhatbar *et al.*, 2018; Itoh *et al.*, 2018; Tassoni *et al.*, 2019; Barbar *et al.*, 2020; Wheeler *et al.*, 2020; Williams *et al.*, 2020). In their reactive state, astrocytes drive both acute and chronic phases of neuroinflammation, and contribute to the transition from a neuroinflammatory to a neurotoxic, or neurodegenerative, state (Rothhammer et al., 2016, Wheeler et al., 2020, Liddelow et al., 2017). Conversion from acute inflammatory injury to a chronic neurodegenerative state is a clinical hallmark of secondary progressive MS and also occurs in a range of other neurologic diseases, including ischemic stroke and dementia (Cekanaviciute and Buckwalter, 2016; Arranz and De Strooper, 2019; Guerrero-Garcia, 2020). Future work defining the temporal dynamics of astrocytic JAM-A signaling and other receptor-mediated astrocyte-immune cell interactions within the PVSs will help us to understand how acute neuroinflammatory changes may prime the CNS for longitudinal injury or repair, leading to novel translational strategies for progressive

MS and other neurodegenerative diseases.

Summary:

Astrocytic JAM-A increases MMP-2, CCL-2 and GM-CSF in co-culture with T cells, and increases MMP-2 in spinal cord tissues during EAE promoting the migration of T cells out of the perivascular spaces and into the parenchyma, exacerbating inflammatory histopathology and clinical disability. Exogenous administration of soluble JAM-A blocking peptide protects against EAE demonstrating that blockade of contact mediated astrocyte-immune cell signaling within the perivascular space represents a novel therapeutic strategy against multiple sclerosis (MS) and other CNS autoimmune diseases.

Materials and Methods:

Cell mono-culture: astrocytes. Primary human fetal astrocytes were obtained from Lonza (CC-2565) and grown to confluence on glass confocal plates (Mat-Tek, P35GC-1.5-14C) in Astrocyte Growth Medium (AGM). AGM was comprised of MCDB 131 Medium (Gibco 10372-019) and Astrocyte BulletKit factors (Lonza, CC-3186), providing for 3% FBS, 2mM L-glutamine, 30μg/ml gentamicin and 15ng/ml amphotericin (GA-1000), 70uM ascorbic acid, 3ng/ml rhEGF and 7.5μg/ml insulin. Astrocytes were then pre-treated with MCDB 131 alone for 24 hours and then treated with 20ng/ml human recombinant interleukin-1 beta (IL-1β), 100ng/ml CCL-2, IL-1β + CCL-2 or vehicle for 6 and 24 hours and then were fixed in ice cold 4% paraformaldehyde (PFA) in1x PBS for 30 minutes then processed for immunohistochemical staining.

Cell co-culture: astrocytes. Primary human fetal astrocytes were plated to 70% confluence on a 20 cm^2 tissue culture dish (Corning, 353003) in AGM. Astrocytes were washed with PBS twice, dissociated gently with 0.05% trypsin, centrifuged, resuspended and nucleofected with 2 μM siRNA of either non-targeting (*siNT*) or *JAM-A (siJAM-A)* targeting sequences, as detailed below. Transfected astrocytes were re-plated and allowed to grow for 24 hours in AGM. Astrocytes were then serum-starved in MCDB 131 for 24 hours and then treated with 20ng/mL IL-1β for 24 hours. MCDB 131 was refreshed and astrocytes were then paired with 1-2 × 10^6 isolated $CD3^+$ T lymphocytes on a 20 cm^2 tissue culture dish for 24 hours. After co-culture, $CD3^+$ T lymphocytes, astrocytes and supernatants were separated, sonicated and stored at -20°C for protease and cytokine array experiments.

Cell co-culture: T lymphocytes. Human T lymphocytes were extracted from human blood of healthy adult donors freshly collected in lavender K2-EDTA tubes (BD #367861). Briefly, peripheral blood mononuclear cells (PBMCs) were isolated from whole blood samples using density centrifugation with Ficoll-Paque PLUS (GE Healthcare). Six milliliters of whole blood were diluted with an equal volume of HBSS (Mediatech Inc.) and layered onto 15-ml tubes prefilled with 4 mL of density gradient medium. Tubes were centrifuged for 1 hour at 620 relative centrifugal force (rcf). PBMCs were collected from their density gradient layer using a transfer pipette, washed in HBSS (Mediatech Inc.) and centrifuged for 15-20 minutes at 620 rcf x 2. The PBMC pellet was resuspended in eluent buffer and processed with a magnetic labeling and separation protocol using a human pan-T cell ($CD3^+$) (Miltenyi, 130-096-535). Cells were then activated in lymphocyte growth medium ((LGM), comprised of RPMI 1640 (Gibco), 10% FBS,

2mM L-glutamine, 1% 2-mercaptoethanol) at 37°C for 72 hours with 4μg/mL anti-CD28 (eBioscience 16-0298-85) on 20cm^2 tissue culture dishes pre-treated with 7μg/mL anti-CD3$^+$ (eBioscience 16-0037-85) in PBS at 37°C for 2 hours. After activation, T lymphocytes were centrifuged and 1-2 × 10^6 cells applied to astrocyte cultures for 24 hours before sample separation and processing, as above.

Human Protease and Cytokine Arrays. Reactive astrocyte and CD3$^+$ T lymphocyte co-cultures were prepared as outlined above. Culture medium supernatant with CD3$^+$ T lymphocytes was aspirated from co-cultures after 24 hours. Aspirant was centrifuged at 620 rcf for 7 minutes, then supernatant stored at 20°C. The CD3$^+$ T lymphocyte pellet was reconstituted and harvested in cell lysis buffer, which was sonicated and then stored at 20°C. Adherent astrocytes from the 20 cm^2 tissue culture dish were harvested in cell lysis buffer, sonicated and then stored at 20°C. Supernatant (500 μl), T lymphocyte (100 μg) and astrocyte (100 μg) samples were then applied to human protease (R&D, ARY021B) and cytokine (R&D, ARY005B) ELISA array kits per the manufacturer's instructions in 3 biological replicates. Quantification of protease signal was performed by densitometry as follows: non-saturated developed films were scanned using a Canon LiDE scanner (Canon USA), and mean pixel density of each duplicate array probe was measured using ImageJ software (NIH). Data were standardized to 3 duplicated reference probes, and the relative change between *siNT* and *siJAM-A* treated conditions was calculated and then compared to the relative change in reference probe signal. Statistical analyses were performed using unpaired two sample t-tests with unequal variance**.** Comparisons included all array probes initially and then those without visually detectable signals above background were excluded and not considered biologically significant.

Mouse Proteome Arrays. Spinal cord tissue from WT (n=4) and CKO (n=3) mice was harvested at 5 days from EAE disease onset, homogenized in PBS with protease inhibitors and stored at -80° C before thawing for experiments. Samples were quantified for protein concentration and 200 µg applied to mouse proteome ELISA profiler arrays (R&D, ARY028) per the manufacturer's instructions in 4 and 3 biological replicates. Quantification of protease signal and analysis of the relative change between WT and CKO was performed as outlined above in *Human Protease and Cytokine Arrays*.

Chemical and Protein Reagents. Human IL-1β and CCL-2 were purchased from PeproTech and used at 20 ng/mL and 10ng/mL, respectively, for mono-culture experiment described above.

JAM-Ap. JAM-A blocking peptide (JAM-Ap) and control peptide were synthesized to order at >95% purity from New England Biopeptide with the following sequences: NPKSTRAFSNDDYVLNPTTG for JAMA-p and NLFSVDTPNGKTASDNYPRT for control, as designed and characterized by a previous group (Sladojevic *et al.*, 2014). Daily intraperitoneal injection of 1µg in 0.1mL of sterile 0.9% NaCl starting on Day 7 post EAE immunization was performed in EAE experiments.

Antibodies. Catalog numbers and concentrations of all antibodies are as follows. Anti-GFAP (130300, rat, 1:200), anti-occludin (711500, rabbit, 1:125), anti-IgG (A11029, mouse, 1:100) were from Invitrogen. Anti–JAM-A (sc53623, mouse, 1:100) was from Santa Cruz Biotechnology. Fluoromyelin was from ThermoFisher (F34651, 1:300). Anti-fibrinogen (A0080,

rabbit, 1:150) was from Dako. Anti-CD3 (16-0037-85, 1:100), anti-CD4 (14-9766-82, 1:100), anti-CD31 (550274 1:100), and anti-CD45 (550539, 1:100), all rat, were from eBioscience. Anti-CD4 (ab183685, mouse, 1:50)was from Abcam. Anti-NeuN (MAB377, mouse, 1:100) and anti–myelin basic protein (MBP) (MAB386, rat, 1:500), anti-Olig2 (MABN50, mouse, 1:500) and anti-AQP4 (AB3594, 1:200) were from Millipore. Anti-laminin (L9393, rabbit, 1:200) was from Sigma-Aldrich. Anti-Iba1 (109-19741, rabbit, 1:500) was from Wako.

siRNA. Human astrocyte cultures were nucleofected with siRNA (2 μM) with non-targeting sequences (*siNT*) or *JAM-A (siJAM-A)* targeting sequences (Thermo Scientific Dharmacon, siGENOME SMART pool), using an Amaxa nucleofector (program A033) with the Basic Glial Kit (Amaxa). The extent and specificity of gene silencing was confirmed by immunoblotting as reported in a previous study (Horng *et al.*, 2017).

Mice. *mGfap-Cre* (B6.Cg-Tg(Gfap-cre)73.12Mvs/J) mice were genetically engineered in the laboratory of Michael Sofroniew (UCLA) and are available for purchase from Jackson laboratories (https://www.jax.org/strain/012886). *Cre* expression is astrocyte-specific except in areas of adult neurogenesis, where it is also observed in some neural progenitors (Garcia *et al.*, 2004). *JAM-A*$^{f/fll}$ mice were obtained from Charles Parkos (University of Michigan, Ann Arbor, Michigan, USA) and Terence Dermody (University of Pittsburgh, Pittsburgh, Pennsylvania, USA) (Cera *et al.*, 2004; Laukoetter *et al.*, 2007). For all experiments, *mGfap-Cre JAM-A*$^{fl/fl}$ female mice were crossed with *JAM-A*$^{fl/fl}$ male mice to generate ~50% *mGfap-Cre JAM-A*$^{fl/fl}$ (conditional knock-out (CKO) mice) and ~50% *JAM-A*$^{fl/fl}$ (wild type (WT)) littermate controls. Selective deletion of JAM-A in GFAP positive cells was confirmed in a previous study

For Peer Review Only

(Horng *et al.*, 2017). Total JAM-A knock out (KO) mice were generated by breeding *mGfap-Cre JAM-A$^{fl/fl}$* male mice (which express Cre in germline cells) to *JAM-A$^{fl/fl}$* to create JAM-A$^{fl/-}$ mice which were then crossed to create JAM-A$^{-/-}$. Genotyping primers were: *mGfap-Cre* forward (GfF) ACC AGC CAG CTA TCA ACT C, reverse (GfR) TAT ACG CGT GCT AGC GAA GAT CTC CAT CTT CCA GCA G, 350 bp; *JAM-A* forward (JaKOF) TCT TTT CAC CAA TCG GAA CG, reverse (JF2R) AAA AAC TCT AGG AAC TCA CCC AGG A, band 200 bp (wt), 320 bp (flox); *JAM-A* excised forward (TS379) CCT CTC TTT TCA CCA ATC GGA, JAM-A excised reverse (TS512) TCT TCT TCA GAC GCC GAA CCT. PCR conditions for all primer sets were: 94°C for 4 minutes; 35 cycles of 94°C for 30 seconds, 56°C for 30 seconds, and 72°C for 30 seconds; then 72°C for 10 minutes.

Cortical microinjection of AdIL-1. Mice (8–12 weeks old, at least 5 per condition per time point, on the C57BL/6 background) were anesthetized using isoflurane and placed into a stereotactic frame (Kopf). AdIL-1 or AdDL70 control (AdCtrl) (10^6 PFU) was microinjected into the cerebral cortex at y = 1 mm caudal to bregma, x = 2 mm, z = 1.5 mm. Animals were allowed to recover for 7 days and then were sacrificed and perfused with 10mL of 1x PBS and 10ml 4% PFA in1x PBS.

EAE. Mice (male and females, 10–13 weeks old, at least 8 animals per group for each experiment, on the C57BL/6 background) were subcutaneously injected at cervical and lumbar sites 0.1 cc of $MOG_{35–55}$ in complete Freund's adjuvant (CFA) (1 mg/mL) followed by intraperitoneal injection of 0.1 cc pertussis toxin on day 0 and day 1 (Hooke Laboratories) . Healthy control (HC) mice received the injection of CFA emulsion with no MOG_{35-55}. Mice were

rated daily on a standard 5-point motor scale from days 7–28 after induction: 0, no symptoms; 1, floppy tail; 2, hind limb weakness (paraparesis); 3, hind limb paralysis (paraplegia); 4, forelimb and hind limb paralysis; 5, death. The average EAE score consisted of the average score across all animals of the same genotype at Day 28 (the end of the experiment). Cumulative EAE score consisted of the average summed score per animal across all animals of the same genotype at Day 28 (the end of the experiment). Average mortality was calculated as the proportion of animals with a score of 5 by the end of the experiment (Day 28). Disease free values were calculated by the proportion of animals who maintained a score of 0 throughout the entire experiment (Day 7-28). Survival curves for mortality and disease induction were performed and analyzed with a Mantel-Cox test. Three independent experiments were performed for WT and CKO and two independent experiments for KO. Raters were blind to genotype where possible; breeding conditions required separate WT and KO cages in parallel to the CKO colony, which produced both WT and CKO offspring. Three independent experiments were performed for JAM-Ap and scramble control experiments. Raters were blinded to treatment group in the treatment experiments.

Flow cytometry. WT and CKO mice were anesthetized and perfused with 5 mL PBS at 4-5 days from onset of disease in EAE (days 14-20 from induction). Spinal cords and spleens were collected in cold PBS and mechanically dissociated. Spleen samples were passed through a 70-µm filter, then incubated in red blood cell (RBC) lysis buffer (BioLegend) for 2 minutes at room temperature and washed with PBS. Spinal cords were passed through a 100-µm filter and separated from myelin using a 60%/30% Percoll gradient. Cell suspensions were then collected, counted, and subjected first to a Zombie Yellow stain (Biolegend, 423103) and wash followed by

incubation with FITC-anti-CD3 (Biolegend, 100306) and APC/Cy7-anti-CD4 (Biolegend, 100355) antibodies in cell staining buffer (BioLegend). Cells were washed with FACS buffer (2% FBS in PBS), fixed and permeabilized following manufacturer's instructions for staining using the FIX & PERM® Cell Permeabilization Kit (Invitrogen). Forward scatter and side scatter were used to gate cells excluding debris and cell aggregates, Zombie Yellow was used to exclude dead cells and then percentages of CD3 positive cells were measured with a subsequent gate to CD4. Flow cytometry for spinal cord tissues was set to run $CD3^+$ events up to 2000; total $CD3^+$ counts from actual samples ranged from 298 to 1340. Flow cytometry for spleen tissues all exceeded $CD3^+$ counts of 40,000. Data was acquired on the Invitrogen™ Attune™ NxT Flow Cytometer and analyzed with FCS Express software (De Novo) at the Flow Cytometry CoRE at Mount Sinai.

Immunohistochemistry. Brains and spinal cords were dissected from animals perfused with 10mL ice cold 1x PBS followed by 10ml 4% PFA-1x PBS, tissues were subjected to 2 hours post-fixation in 4% PFA-1xPBS followed by storage in 30% sucrose-1x PBS at 4 degrees until sectioning. Immunostaining was performed on 25um coronal (brain) and axial (spinal cord) sections. For all antibody staining, sections underwent antigen retrieval in citrate (pH 6.0; 100°C) for 20 minutes. For laminin, CD4 and CD45, sections were treated with 0.5 mg/mL protease XIV (Sigma-Aldrich) at 37°C for 5 minutes. Primary antibodies were used at concentrations ranging from 1:50-1:500. Samples were examined using a Leica Microsystems confocal microscope, and stacks were collected with z of 1 μm.

Morphometric analysis. Morphometric analyses were performed using NIH ImageJ and Leica LAS softwares and all analyses were performed blinded to treatment group and genotype. For studies *in vitro*, JAM-A and occludin histochemical stains were analyzed in projections from astrocyte cultures. Colocalization analysis was performed using the ImageJ Just Another Colocalization Plugin (JACoP). For studies *in vivo*, JAM-A, occludin, pan-laminin, Olig2, Iba1, CD4, CD45, fluoromyelin (FM), NeuN and GFAP histochemical stains were analyzed in projections from cortical and spinal cord sections at the lumbar level. Cortical AdIL-1 injection lesions were analyzed on coronal brain sections of 25 μm thickness distributed serially across 10 slides from the posterior to anterior end of the brain; adjacent sections on the same slide were roughly 250 μm apart. Sections for analysis were selected to represent the center of the lesion, corresponding to the area of greatest lesion length as noted by width of NeuN loss and GFAP positivity. Field of analysis for lymphocyte localization relative to the PVS was selected on a 40x field of approximately 385 μm width centered over the midline of the cortical injection site. EAE spinal cord lesions were analyzed on axial spinal cord sections of 25 μm thickness distributed serially across 10 slides from the caudal to rostral end of the lumbar, thoracic and cervical spinal cord; adjacent sections on the same slide were roughly 250 μm apart. Sections for analysis focused on the lumbar white matter (anterolateral tract) and gray matter (dorsal horn). At least three representative images were quantified and averaged from 3-6 age- and sex-matched animals per condition per genotype per time point. Myelin loss, neuronal loss and astrocyte reactivity were quantified by measuring the FM positive area, counting $NeuN^+$ cells and measuring GFAP positive pixel sums normalized to total area in each 200 x 200 μm^2 field of interest in matched projections at ×20 magnification.

Statistics. Results are reported as mean ± SEM. Student's *t* test and Mann-Whitney U tests were used to compare two groups of unmatched samples. One way ANOVA and Kruskal-Wallis H test were used to compare more than two groups with multiple comparisons. For multiple comparisons of the EAE disease course, 2-way ANOVA followed by Bonferroni post-test correction was used. Non-parametric analyses of EAE scores were also performed using one-way ANOVA (Friedman) with Dunn's multiple comparisons correction and Kolmogorov-Smirnov test comparing cumulative distributions. In all cases, p less than 0.05 was considered significant.

Study approval. Use of commercially available human astrocytes and anonymized human blood donor samples was approved by the IRB at the Icahn School of Medicine at Mount Sinai (ISMMS). Studies using mice were approved by the IACUC at the ISMMS, and adhered to the American Veterinary Medical Association guidelines. The ISMMS has an Animal Welfare Assurance on file with the Office for Laboratory Animal Welfare (Assurance no. A3111-01).

Data Availability Statement:

Only

The data that support the findings of this study are available from the corresponding author, upon reasonable request.

Competing Interests:

The authors have no conflicts of interest to disclose.

CRediT Author contributions:

Mario Amatruda: Conceptualization, Methodology, Visualization, Validation, Formal analysis, Investigation, Writing – Review and Editing, Project administration

Candice Chapouly: Conceptualization, Methodology, Validation, Formal analysis, Investigation, Writing – Review and Editing, Visualization, Project administration

Viola Woo: Methodology, Validation, Formal analysis

Farinaz Safavi: Methodology, Formal analysis

Joy Zhang: Methodology, Investigation, Validation, Formal analysis

David Dai: Methodology, Investigation, Validation, Formal analysis

Anthony Therattil: Methodology, Investigation

Chang Moon: Investigation

Alexandra Gordon: Investigation

Charles Parkos: Resources

Sam Horng: Conceptualization, Methodology, Validation, Formal analysis, Investigation, Writing – Original Draft, Review and Editing, Visualization, Supervision, Project administration, Funding acquisition

For Review Only

Acknowledgments:

The authors thank Dr. Anne Schaefer, Dr. Patrizia Casaccia, Dr. Fred Lublin and Dr. Stuart Sealfon for their helpful discussions on this project and the manuscript.

Funding:

This work was supported by grant funding to Dr. Horng: National Institutes of Health (NIH) National Institute of Neurological Diseases and Stroke (NINDS) K08 NS102507-01A1, NIH R25NS079102, a Career Transition Award by the National Multiple Sclerosis Society and the Conrad N. Hilton Foundation, philanthropic support by the Jayne and Harvey Beker Foundation and a post-doctoral Neuroscience fellowship from the Leon Levy Foundation. Dr. Safavi was supported by a post-doctoral Neuroscience fellowship from the Leon Levy Foundation.

The MSSM Microscopy and Flow Cytometry Shared Resource Facilities were utilized in this study; core facilities receive support from National Institutes of Health/National Cancer Institute Grant R24 CA095823.

For Review Only

References:

Abbott NJ, Ronnback L, Hansson E. Astrocyte-endothelial interactions at the blood-brain barrier. Nat Rev Neurosci 2006; 7(1): 41-53.
Agrawal S, Anderson P, Durbeej M, van Rooijen N, Ivars F, Opdenakker G*, et al.* Dystroglycan is selectively cleaved at the parenchymal basement membrane at sites of leukocyte extravasation in experimental autoimmune encephalomyelitis. J Exp Med 2006; 203(4): 1007-19.
Anderson MA, Burda JE, Ren Y, Ao Y, O'Shea TM, Kawaguchi R*, et al.* Astrocyte scar formation aids central nervous system axon regeneration. Nature 2016; 532(7598): 195-200.
Arranz AM, De Strooper B. The role of astroglia in Alzheimer's disease: pathophysiology and clinical implications. Lancet Neurol 2019; 18(4): 406-14.
Barbar L, Jain T, Zimmer M, Kruglikov I, Sadick JS, Wang M*, et al.* CD49f Is a Novel Marker of Functional and Reactive Human iPSC-Derived Astrocytes. Neuron 2020; 107(3): 436-53 e12.
Barthelmes J, Tafferner N, Kurz J, de Bruin N, Parnham MJ, Geisslinger G*, et al.* Induction of Experimental Autoimmune Encephalomyelitis in Mice and Evaluation of the Disease-dependent Distribution of Immune Cells in Various Tissues. J Vis Exp 2016(111).
Bhowmick S, D'Mello V, Caruso D, Wallerstein A, Abdul-Muneer PM. Impairment of pericyte-endothelium crosstalk leads to blood-brain barrier dysfunction following traumatic brain injury. Exp Neurol 2019; 317: 260-70.
Cekanaviciute E, Buckwalter MS. Astrocytes: Integrative Regulators of Neuroinflammation in Stroke and Other Neurological Diseases. Neurotherapeutics 2016; 13(4): 685-701.
Cekanaviciute E, Fathali N, Doyle KP, Williams AM, Han J, Buckwalter MS. Astrocytic transforming growth factor-beta signaling reduces subacute neuroinflammation after stroke in mice. Glia 2014; 62(8): 1227-40.
Cera MR, Del Prete A, Vecchi A, Corada M, Martin-Padura I, Motoike T*, et al.* Increased DC trafficking to lymph nodes and contact hypersensitivity in junctional adhesion molecule-A-deficient mice. J Clin Invest 2004; 114(5): 729-38.
Chhatbar C, Detje CN, Grabski E, Borst K, Spanier J, Ghita L*, et al.* Type I Interferon Receptor Signaling of Neurons and Astrocytes Regulates Microglia Activation during Viral Encephalitis. Cell Rep 2018; 25(1): 118-29 e4.
De Keyser J, Laureys G, Demol F, Wilczak N, Mostert J, Clinckers R. Astrocytes as potential targets to suppress inflammatory demyelinating lesions in multiple sclerosis. Neurochem Int 2010; 57(4): 446-50.
dos Santos AC, Barsante MM, Arantes RM, Bernard CC, Teixeira MM, Carvalho-Tavares J. CCL2 and CCL5 mediate leukocyte adhesion in experimental autoimmune encephalomyelitis--an intravital microscopy study. J Neuroimmunol 2005; 162(1-2): 122-9.
Duncker PC, Stoolman JS, Huber AK, Segal BM. GM-CSF Promotes Chronic Disability in Experimental Autoimmune Encephalomyelitis by Altering the Composition of Central Nervous System-Infiltrating Cells, but Is Dispensable for Disease Induction. J Immunol 2018; 200(3): 966-73.
Ebnet K, Suzuki A, Ohno S, Vestweber D. Junctional adhesion molecules (JAMs): more molecules with dual functions? J Cell Sci 2004; 117(Pt 1): 19-29.

For Review Only

Engelhardt B, Coisne C. Fluids and barriers of the CNS establish immune privilege by confining immune surveillance to a two-walled castle moat surrounding the CNS castle. Fluids Barriers CNS 2011; 8(1): 4.

Engelhardt B, Ransohoff RM. Capture, crawl, cross: the T cell code to breach the blood-brain barriers. Trends Immunol 2012; 33(12): 579-89.

Fan S, Weight CM, Luissint AC, Hilgarth RS, Brazil JC, Ettel M, *et al.* Role of JAM-A tyrosine phosphorylation in epithelial barrier dysfunction during intestinal inflammation. Mol Biol Cell 2019; 30(5): 566-78.

Flemming S, Luissint AC, Nusrat A, Parkos CA. Analysis of leukocyte transepithelial migration using an in vivo murine colonic loop model. JCI Insight 2018; 3(20).

Frischer JM, Weigand SD, Guo Y, Kale N, Parisi JE, Pirko I, *et al.* Clinical and pathological insights into the dynamic nature of the white matter multiple sclerosis plaque. Ann Neurol 2015; 78(5): 710-21.

Galli E, Hartmann FJ, Schreiner B, Ingelfinger F, Arvaniti E, Diebold M, *et al.* GM-CSF and CXCR4 define a T helper cell signature in multiple sclerosis. Nat Med 2019; 25(8): 1290-300.

Garcia AD, Doan NB, Imura T, Bush TG, Sofroniew MV. GFAP-expressing progenitors are the principal source of constitutive neurogenesis in adult mouse forebrain. Nat Neurosci 2004; 7(11): 1233-41.

Ge S, Shrestha B, Paul D, Keating C, Cone R, Guglielmotti A, *et al.* The CCL2 synthesis inhibitor bindarit targets cells of the neurovascular unit, and suppresses experimental autoimmune encephalomyelitis. J Neuroinflammation 2012; 9: 171.

Gerwien H, Hermann S, Zhang X, Korpos E, Song J, Kopka K, *et al.* Imaging matrix metalloproteinase activity in multiple sclerosis as a specific marker of leukocyte penetration of the blood-brain barrier. Sci Transl Med 2016; 8(364): 364ra152.

Gimenez MA, Sim J, Archambault AS, Klein RS, Russell JH. A tumor necrosis factor receptor 1-dependent conversation between central nervous system-specific T cells and the central nervous system is required for inflammatory infiltration of the spinal cord. Am J Pathol 2006; 168(4): 1200-9.

Gimenez MA, Sim JE, Russell JH. TNFR1-dependent VCAM-1 expression by astrocytes exposes the CNS to destructive inflammation. J Neuroimmunol 2004; 151(1-2): 116-25.

Guerrero-Garcia JJ. The role of astrocytes in multiple sclerosis pathogenesis. Neurologia 2020; 35(6): 400-8.

Hartmann C, Schwietzer YA, Otani T, Furuse M, Ebnet K. Physiological functions of junctional adhesion molecules (JAMs) in tight junctions. Biochim Biophys Acta Biomembr 2020; 1862(9): 183299.

Horng S, Therattil A, Moyon S, Gordon A, Kim K, Argaw AT, *et al.* Astrocytic tight junctions control inflammatory CNS lesion pathogenesis. J Clin Invest 2017; 127(8): 3136-51.

Ifergan I, Davidson TS, Kebir H, Xu D, Palacios-Macapagal D, Cann J, *et al.* Targeting the GM-CSF receptor for the treatment of CNS autoimmunity. J Autoimmun 2017; 84: 1-11.

Imitola J, Rasouli J, Watanabe F, Mahajan K, Sharan AD, Ciric B, *et al.* Elevated expression of granulocyte-macrophage colony-stimulating factor receptor in multiple sclerosis lesions. J Neuroimmunol 2018; 317: 45-54.

Itoh N, Itoh Y, Tassoni A, Ren E, Kaito M, Ohno A, *et al.* Cell-specific and region-specific transcriptomics in the multiple sclerosis model: Focus on astrocytes. Proc Natl Acad Sci U S A 2018; 115(2): E302-E9.

For Review Only

Kara EE, McKenzie DR, Bastow CR, Gregor CE, Fenix KA, Ogunniyi AD*, et al.* CCR2 defines in vivo development and homing of IL-23-driven GM-CSF-producing Th17 cells. Nat Commun 2015; 6: 8644.
Kim RY, Hoffman AS, Itoh N, Ao Y, Spence R, Sofroniew MV*, et al.* Astrocyte CCL2 sustains immune cell infiltration in chronic experimental autoimmune encephalomyelitis. J Neuroimmunol 2014; 274(1-2): 53-61.
Korner H, Riminton DS, Strickland DH, Lemckert FA, Pollard JD, Sedgwick JD. Critical points of tumor necrosis factor action in central nervous system autoimmune inflammation defined by gene targeting. J Exp Med 1997; 186(9): 1585-90.
Kroenke MA, Chensue SW, Segal BM. EAE mediated by a non-IFN-gamma/non-IL-17 pathway. Eur J Immunol 2010; 40(8): 2340-8.
Kummer D, Ebnet K. Junctional Adhesion Molecules (JAMs): The JAM-Integrin Connection. Cells 2018; 7(4).
Lakshmi SP, Reddy AT, Naik MU, Naik UP, Reddy RC. Effects of JAM-A deficiency or blocking antibodies on neutrophil migration and lung injury in a murine model of ALI. Am J Physiol Lung Cell Mol Physiol 2012; 303(9): L758-66.
Lassmann H. Pathogenic Mechanisms Associated With Different Clinical Courses of Multiple Sclerosis. Front Immunol 2018; 9: 3116.
Laukoetter MG, Nava P, Lee WY, Severson EA, Capaldo CT, Babbin BA*, et al.* JAM-A regulates permeability and inflammation in the intestine in vivo. J Exp Med 2007; 204(13): 3067-76.
Laureys G, Gerlo S, Spooren A, Demol F, De Keyser J, Aerts JL. beta(2)-adrenergic agonists modulate TNF-alpha induced astrocytic inflammatory gene expression and brain inflammatory cell populations. J Neuroinflammation 2014; 11: 21.
Levesque SA, Pare A, Mailhot B, Bellver-Landete V, Kebir H, Lecuyer MA*, et al.* Myeloid cell transmigration across the CNS vasculature triggers IL-1beta-driven neuroinflammation during autoimmune encephalomyelitis in mice. J Exp Med 2016; 213(6): 929-49.
Levine J, Kwon E, Paez P, Yan W, Czerwieniec G, Loo JA*, et al.* Traumatically injured astrocytes release a proteomic signature modulated by STAT3-dependent cell survival. Glia 2016; 64(5): 668-94.
Liddelow SA, Guttenplan KA, Clarke LE, Bennett FC, Bohlen CJ, Schirmer L*, et al.* Neurotoxic reactive astrocytes are induced by activated microglia. Nature 2017; 541(7638): 481-7.
Liddelow SA, Sofroniew MV. Astrocytes usurp neurons as a disease focus. Nat Neurosci 2019; 22(4): 512-3.
Luissint AC, Nusrat A, Parkos CA. JAM-related proteins in mucosal homeostasis and inflammation. Semin Immunopathol 2014; 36(2): 211-26.
Luissint AC, Williams HC, Kim W, Flemming S, Azcutia V, Hilgarth RS*, et al.* Macrophage-dependent neutrophil recruitment is impaired under conditions of increased intestinal permeability in JAM-A-deficient mice. Mucosal Immunol 2019; 12(3): 668-78.
Mayo L, Trauger SA, Blain M, Nadeau M, Patel B, Alvarez JI*, et al.* Regulation of astrocyte activation by glycolipids drives chronic CNS inflammation. Nat Med 2014; 20(10): 1147-56.
McCandless EE, Wang Q, Woerner BM, Harper JM, Klein RS. CXCL12 limits inflammation by localizing mononuclear infiltrates to the perivascular space during experimental autoimmune encephalomyelitis. J Immunol 2006; 177(11): 8053-64.
Monaghan KL, Wan ECK. The Role of Granulocyte-Macrophage Colony-Stimulating Factor in Murine Models of Multiple Sclerosis. Cells 2020; 9(3).

For Review Only

Nourshargh S, Krombach F, Dejana E. The role of JAM-A and PECAM-1 in modulating leukocyte infiltration in inflamed and ischemic tissues. J Leukoc Biol 2006; 80(4): 714-8.
Owens T, Bechmann I, Engelhardt B. Perivascular spaces and the two steps to neuroinflammation. Journal of neuropathology and experimental neurology 2008; 67(12): 1113-21.
Padden M, Leech S, Craig B, Kirk J, Brankin B, McQuaid S. Differences in expression of junctional adhesion molecule-A and beta-catenin in multiple sclerosis brain tissue: increasing evidence for the role of tight junction pathology. Acta Neuropathol 2007; 113(2): 177-86.
Ponomarev ED, Shriver LP, Maresz K, Pedras-Vasconcelos J, Verthelyi D, Dittel BN. GM-CSF production by autoreactive T cells is required for the activation of microglial cells and the onset of experimental autoimmune encephalomyelitis. J Immunol 2007; 178(1): 39-48.
Prajeeth CK, Kronisch J, Khorooshi R, Knier B, Toft-Hansen H, Gudi V*, et al.* Effectors of Th1 and Th17 cells act on astrocytes and augment their neuroinflammatory properties. J Neuroinflammation 2017; 14(1): 204.
Rasouli J, Ciric B, Imitola J, Gonnella P, Hwang D, Mahajan K*, et al.* Expression of GM-CSF in T Cells Is Increased in Multiple Sclerosis and Suppressed by IFN-beta Therapy. J Immunol 2015; 194(11): 5085-93.
Rothhammer V, Mascanfroni ID, Bunse L, Takenaka MC, Kenison JE, Mayo L*, et al.* Type I interferons and microbial metabolites of tryptophan modulate astrocyte activity and central nervous system inflammation via the aryl hydrocarbon receptor. Nat Med 2016; 22(6): 586-97.
Schlager C, Korner H, Krueger M, Vidoli S, Haberl M, Mielke D*, et al.* Effector T-cell trafficking between the leptomeninges and the cerebrospinal fluid. Nature 2016; 530(7590): 349-53.
Schmitt MM, Fraemohs L, Hackeng TM, Weber C, Koenen RR. Atherogenic mononuclear cell recruitment is facilitated by oxidized lipoprotein-induced endothelial junctional adhesion molecule-A redistribution. Atherosclerosis 2014; 234(2): 254-64.
Sladojevic N, Stamatovic SM, Keep RF, Grailer JJ, Sarma JV, Ward PA*, et al.* Inhibition of junctional adhesion molecule-A/LFA interaction attenuates leukocyte trafficking and inflammation in brain ischemia/reperfusion injury. Neurobiol Dis 2014; 67: 57-70.
Smolders J, Heutinck KM, Fransen NL, Remmerswaal EBM, Hombrink P, Ten Berge IJM*, et al.* Tissue-resident memory T cells populate the human brain. Nat Commun 2018; 9(1): 4593.
Sofroniew MV. Astrocyte barriers to neurotoxic inflammation. Nat Rev Neurosci 2015; 16(5): 249-63.
Song J, Wu C, Korpos E, Zhang X, Agrawal SM, Wang Y*, et al.* Focal MMP-2 and MMP-9 activity at the blood-brain barrier promotes chemokine-induced leukocyte migration. Cell Rep 2015; 10(7): 1040-54.
Song J, Wu C, Zhang X, Sorokin LM. In vivo processing of CXCL5 (LIX) by matrix metalloproteinase (MMP)-2 and MMP-9 promotes early neutrophil recruitment in IL-1beta-induced peritonitis. J Immunol 2013; 190(1): 401-10.
Stamatovic SM, Sladojevic N, Keep RF, Andjelkovic AV. Relocalization of junctional adhesion molecule A during inflammatory stimulation of brain endothelial cells. Mol Cell Biol 2012; 32(17): 3414-27.
Tassoni A, Farkhondeh V, Itoh Y, Itoh N, Sofroniew MV, Voskuhl RR. The astrocyte transcriptome in EAE optic neuritis shows complement activation and reveals a sex difference in astrocytic C3 expression. Sci Rep 2019; 9(1): 10010.
Weber C, Fraemohs L, Dejana E. The role of junctional adhesion molecules in vascular inflammation. Nat Rev Immunol 2007; 7(6): 467-77.

For Review Only

Wheeler MA, Clark IC, Tjon EC, Li Z, Zandee SEJ, Couturier CP*, et al.* MAFG-driven astrocytes promote CNS inflammation. Nature 2020; 578(7796): 593-9.
Williams JL, Manivasagam S, Smith BC, Sim J, Vollmer LL, Daniels BP*, et al.* Astrocyte-T cell crosstalk regulates region-specific neuroinflammation. Glia 2020; 68(7): 1361-74.
Wojcikiewicz EP, Koenen RR, Fraemohs L, Minkiewicz J, Azad H, Weber C*, et al.* LFA-1 binding destabilizes the JAM-A homophilic interaction during leukocyte transmigration. Biophys J 2009; 96(1): 285-93.

For Review Only

Figure 1

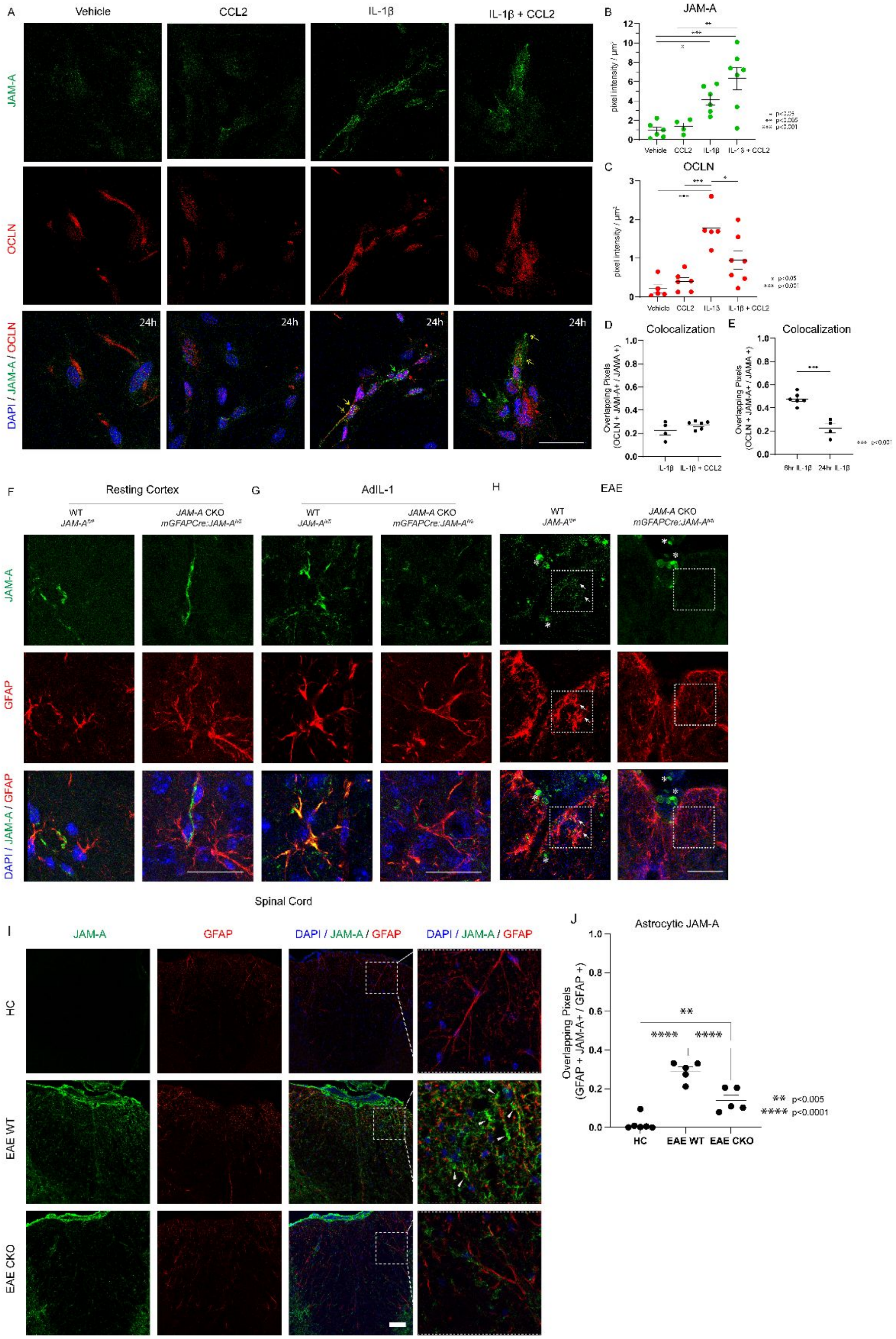
A
Vehicle
CCL2
IL-1β
IL-1β + CCL2
JAM-A
OCLN
DAPI / JAM-A / OCLN
24h
B
JAM-A
C
OCLN
D
Colocalization
E
Colocalization
F
Resting Cortex
G
AdIL-1
H
EAE
GFAP
DAPI / JAM-A / GFAP
Spinal Cord
I
HC
EAE WT
EAE CKO
J
Astrocytic JAM-A
Overlapping Pixels (GFAP + JAM-A+ / GFAP +)
** p<0.005
**** p<0.0001

Figure 1: **Inflammation induces reactive astrocytes to express JAM-A diffusely throughout the cell surface membrane *in vitro* and *in vivo* and this expression was successfully prevented using an astrocyte-specific JAM-A knock-out mouse line.** (A-B) Astrocytic JAM-A (green) was induced *in vitro* at 24 hours after treatment with 20ng/mL IL-1β and the combination of IL-1β + 100ng/mL CCL2 but not CCL2 alone (average vehicle (0.93) vs. IL-1β (4.14) vs. CCL2 (1.36) vs. IL-1β + CCL2 (6.316), vehicle vs. IL-1β: p=0.04, vehicle vs. IL-1β + CCL2: p=0.0004, CCL2 vs. IL-1β + CCL2: p=0.0030, other comparisons p>0.05, analyzed images n=6 vehicle, n=4 CCL2, n=6 IL-1β, n=7 IL-1β + CCL2, one way ANOVA with Tukey's multiple comparison test). JAM-A was both diffusely localized throughout the cell membrane (green arrows) and co-localized with the tight junction marker, occludin (OCLN, red, red arrows; white double headed arrows pointing to overlay of the two proteins in yellow). All results were quantified from at least 4 fields of view from two to three technical replicates per group. Scale bar 50 µm. (C) Astrocytic occludin was similarly induced at 24 hours after treatment with IL-1β but not CCL2 or the combination of IL-1β and CCL2 (average vehicle (0.21) vs. IL-1β (1.78) vs. CCL2 (0.39) vs. IL-1β + CCL2 (0.95), vehicle vs. IL-1β: p=0.0002, CCL-2 vs. IL-1β: p=0.0004, IL-1β vs. IL-1β + CCL2: p=0.028, other comparisons p>0.05, analyzed images n=5 vehicle, n=6 CCL2, n=5 IL-1β, n=7 IL-1β + CCL2, one way ANOVA with Tukey's multiple comparison test). (D) The addition of CCL2 to IL-1β did not change the proportion of JAM-A$^+$ pixels colocalized with OCN$^+$ pixels at 24 hours. (IL-1β (0.22) vs. IL-1β + CCL2 (0.27), p=0.19, analyzed images n=4 IL-1β, n=6 IL-1β + CCL2, unpaired two-tailed t-test. (E) In IL-1β treated cultures, the proportion of JAM-A$^+$ pixels colocalized with OCN$^+$ pixels decreased over time from 6 hours to 24 hours (average at 6 hr (0.47) vs 24 hr (0.21), p<0.0003, analyzed images n=6 at 6 hr, n=4 at 24 hr, unpaired two-tailed t-test).(F-H) JAM-A and GFAP expression patterns were visualized *in*

For Review Only

vivo in resting cortex, AdIL-1 and EAE spinal cord tissue of control (WT) (*JAMA*$^{fl/fl}$), and *JAM-A* conditional knock-out (CKO) (*mGFAPCre:JAMA*$^{fl/fl}$) mice. In the resting cortex (F), JAM-A (green) does not strongly overlap (yellow)with astrocytes(GFAP, red)In the inflamed cortex of WT AdIL-1 brains (G), JAM-A (green) overlaps with with GFAP (red, overlap: yellow) and astrocytic JAM-A appears diminished in CKOs. Scale bars in (F, G) 25 µm. In EAE spinal cord at Day 28 (H), JAM-A (green) overlaps with reactive astrocytes (GFAP, red) of the glia limitans (area of interest outlined in the dotted white box with white arrows pointing to JAM-A within the astrocytic processes), as well as by leukocytes infiltrating the subarachnoid space and CNS parenchyma (white asterisks). In CKOs, leukocytes (white asterisks), but not astrocytes, show JAM-A signal. Scale bar in (H) 50 µm. (I) Astrocytic expression of JAM-A was quantified in spinal cord tissues of healthy controls (HC) and WT and CKO mice at 5 days after the onset of EAE (clinical scores ranging 2.5-3.5 for both groups). Images show immunofluorescences for JAM-A (green), GFAP (red), and DAPI (blue) in the spinal cord dorsal column of HC, WT and CKO mice with EAE. JAM-A expression is nearly undetectable in the spinal cord of HC mice, while it is upregulated in EAE WT and EAE CKO mice. EAE CKO mice show decreased immunoreactivity to JAM-A in GFAP positive astrocytes compared with EAE WT as shown in the higher magnification inset (white dashed square). White arrowheads point to JAM-A^{+} GFAP^{+} astrocytes. Scale bar = 50 um. (J) Colocalization analysis shows a greater proportion of GFAP^{+} pixels that co-localize with JAM-A^{+} pixels during EAE compared with HC (average HC (0.19) vs. EAE WT (0.29) vs. EAE CKO (0.14), HC vs. EAE WT: $p<0.0001$; HC vs EAE CKO: $p=0.0036$) and a decreased proportion in EAE CKO mice compared to EAE WT mice (EAE WT vs. EAE CKO: $p=0.0009$), number of animals HC, n=6; EAE WT, n=5; EAE CKO, n=5, one way ANOVA with Tukey's multiple comparison test. Image analysis was performed on at least 2 images per animal.

For Review Only

Figure 2

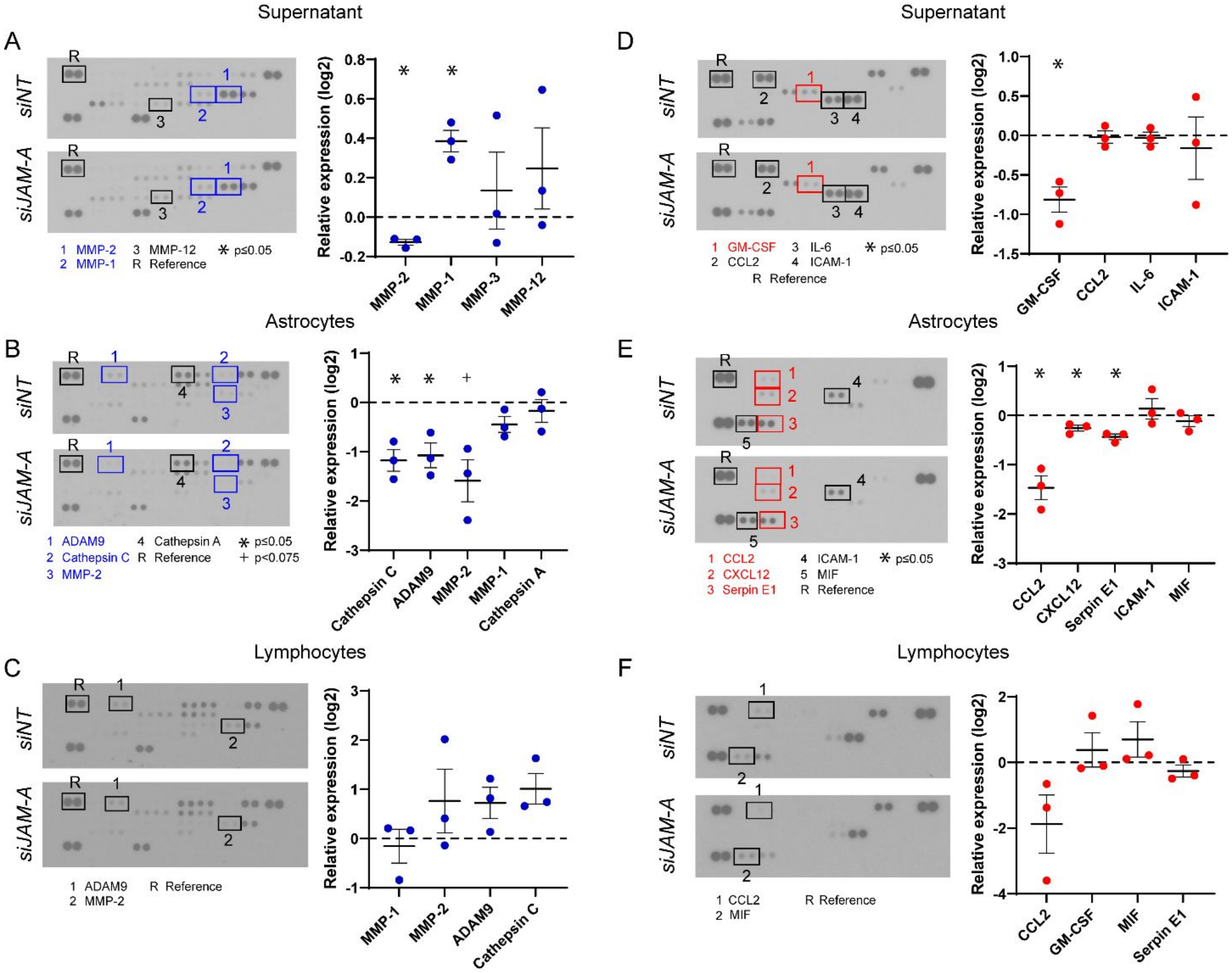
Supernatant
A
siNT
siJAM-A
1 MMP-2 3 MMP-12 * p≤0.05
2 MMP-1 R Reference
Relative expression (log2)
MMP-2
MMP-1
MMP-3
MMP-12
Astrocytes
B
1 ADAM9 4 Cathepsin A * p≤0.05
2 Cathepsin C R Reference + p<0.075
3 MMP-2
Cathepsin C
ADAM9
MMP-2
MMP-1
Cathepsin A
Lymphocytes
C
1 ADAM9 R Reference
2 MMP-2
MMP-1
MMP-2
ADAM9
Cathepsin C
Supernatant
D
1 GM-CSF 3 IL-6 * p≤0.05
2 CCL2 4 ICAM-1
R Reference
GM-CSF
CCL2
IL-6
ICAM-1
Astrocytes
E
1 CCL2 4 ICAM-1 * p≤0.05
2 CXCL12 5 MIF
3 Serpin E1 R Reference
CCL2
CXCL12
Serpin E1
ICAM-1
MIF
Lymphocytes
F
1 CCL2 R Reference
2 MIF
CCL2
GM-CSF
MIF
Serpin E1

Figure 2: Astrocytic JAM-A increases pro-inflammatory protease and cytokine levels in astrocyte-CD3⁺ T cell co-culture. Astrocytes were transfected with JAM-A or non-targeted siRNA (*siJAM-A* vs. *siNT*, then co-cultured with $CD3^+$ T cells for 24 hours and samples processed for human protease and cytokine ELISA immunoassays. (A-C) JAM-A knock-down in astrocytes led to an increase of MMP-1 (relative log_2 expression 0.38, p=0.019, two-tailed unpaired two sample t-test with unequal variance) and decrease of MMP-2 (relative log_2 expression -0.13, p=0.014) in the supernatant and decrease of ADAM9 (relative log_2 expression -1.074, p=0.05) and cathepsin C (relative log_2 expression -1.174, p=0.006) in astrocyte lysates (B). There were no significant changes in protease levels seen in lymphocyte lysates (C). (D-F) Astrocytic JAM-A knock down led to decreased levels of (D) GM-CSF (relative log_2 expression -0.79, p=0.04) in the supernatant and (E) CCL-2 (relative log_2 expression -1.3, p=0.03) in astrocytic lysates. There were no significant changes in cytokine levels seen in lymphocyte lysates (F). Data (A-F) are from three biological replicates; two-tailed paired t-tests were performed on probes demonstrating a visually detectable signal in normalized expression values relative to a reference control.

For Review Only

Figure 3

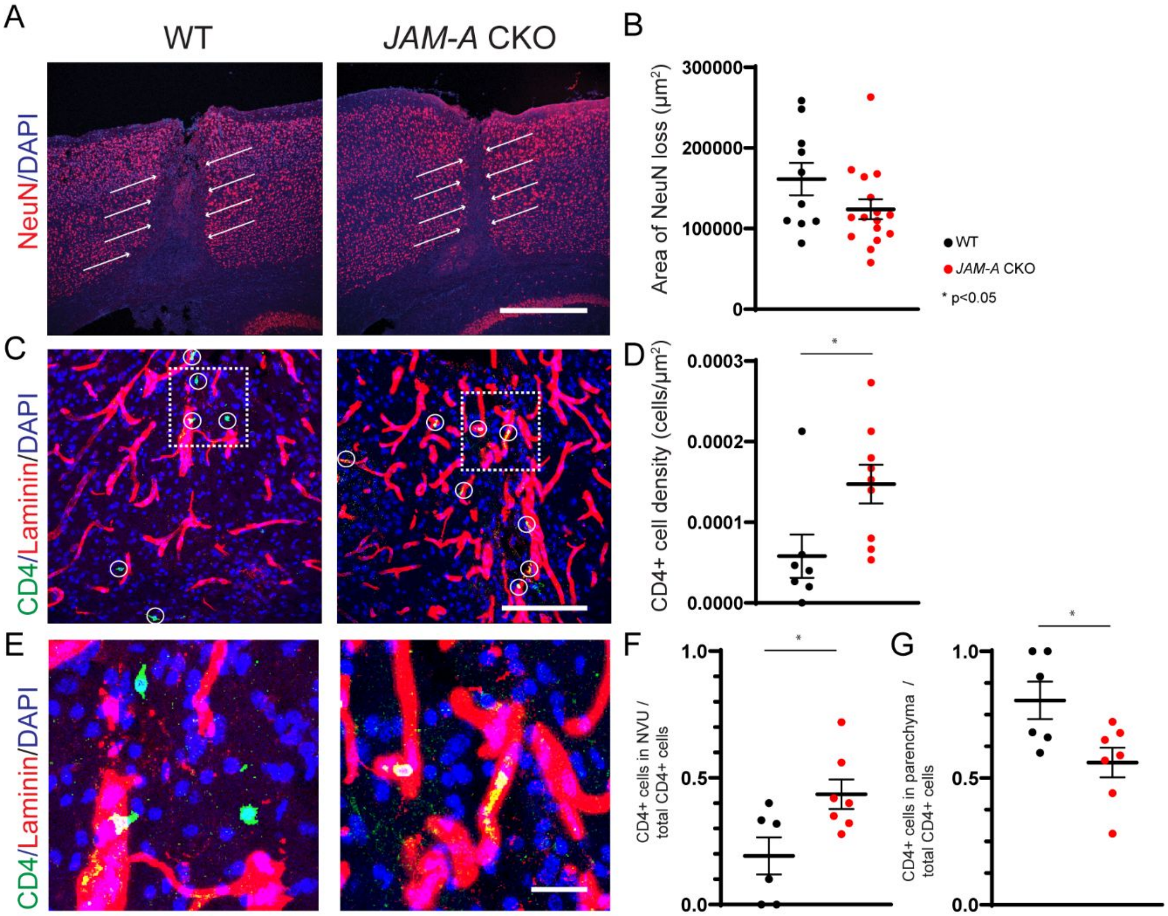

Only

Figure 3: **In inflammatory cortical lesions, $CD4^+$ T cells are arrested at the neurovascular unit (NVU) in the absence of astrocytic JAM-A**. Asymptomatic inflammatory cortical lesions were induced in JAM-A CKO and WT mice with an IL-1β expressing adenovirus (ADIL-1) microinjected into the frontal cortex, with brains harvested for histopathology at 7 days post-injection. (A,B) Lesions in JAM-A CKO mice, as measured by the area of neuronal cell death (loss of NeuN, red, white arrows) showed a trend in smaller lesion size compared to WT mice that did not reach statistical significance (p= 0.18, CKO n=11 mice, WT n=8 mice, Mann Whitney test). (C, D) $CD4^+$ cells (green and circled in white) were increased in number in JAM-A CKO lesions, scale bar: 125 μm (average number/μm^2, 1.5×10^{-4} (CKO) vs. 5.8×10^{-5} (WT), p=0.012, for WT, a range of 0 to 78 cells were analyzed per mouse (Average 22.6); for CKO, a range of 8 to 230 cells were analyzed per mouse (average 47); CKO n=9 mice, WT n=7 mice, Mann-Whitney test). (E-G) In JAM-A CKO mice, a higher proportion of $CD4^+$ cells (green) co-localize to the the laminin (red) positive basement membrane of the neurovascular unit (NVU and a lower proportion in the parenchyma (laminin-negative CNS parenchyma demarcated by DAPI) than in WT mice (F) Average proportion of $CD4^+$ cells in NVU over total $CD4^+$ cells: 0.43 (CKO) vs. 0.19 (WT), p=0.03. (G) Average proportion of $CD4^+$ cells in the parenchyma /total $CD4^+$ cells: 0.81 (CKO) vs 0.56 (WT), p=0.03; in the WT, a range of 1 to 10 cells (average 6.5) were analyzed per mouse; in the CKO, a range of 5 to 18 cells (average 10.1) were analyzed per mouse; CKO n=8 mice, WT n=6 mice, Mann-Whitney tests; scale bar: 25μm).

For Review Only

Figure 4

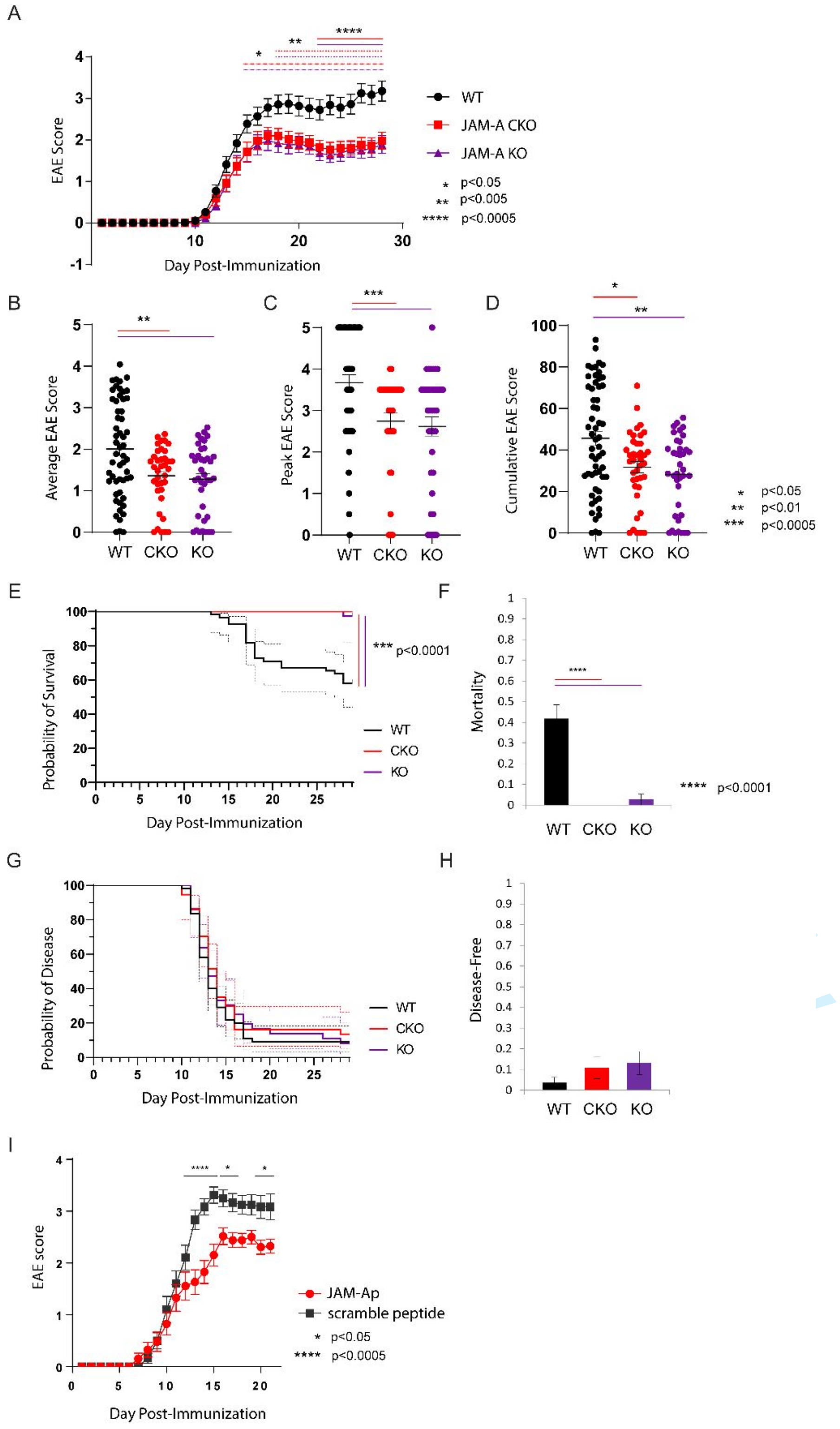
A
EAE Score
Day Post-Immunization
WT
JAM-A CKO
JAM-A KO
* p<0.05
** p<0.005
**** p<0.0005
B
Average EAE Score
WT CKO KO
C
Peak EAE Score
WT CKO KO
D
Cumulative EAE Score
WT CKO KO
* p<0.05
** p<0.01
*** p<0.0005
E
Probability of Survival
Day Post-Immunization
WT
CKO
KO
*** p<0.0001
F
Mortality
WT CKO KO
**** p<0.0001
G
Probability of Disease
Day Post-Immunization
WT
CKO
KO
H
Disease-Free
WT CKO KO
I
EAE score
Day Post-Immunization
JAM-Ap
scramble peptide
* p<0.05
**** p<0.0005

Figure 4: Astrocytic JAM-A promotes clinical disease severity during EAE. (A) JAM-A CKO and KO mice showed a milder course of clinical disability than WT mice with EAE and there was no difference between CKO and KO animals.; (WT vs. CKO: p=0.01, WT vs KO: p<0.0001, CKO vs KO: p=0.07 using non-parametric one way ANOVA Friedman test with Dunn's multiple comparison test; day 15-17: p<0.05 for WT vs CKO and WT vs. KO; day 18-21: p<0.005 for WT vs. CKO and WT vs. KO; day 22-25: p<0.0005 for WT vs. CKO and WT vs. KO, two-way ANOVA with Bonferroni correction). Graph shows pooled data from 2-3 independent EAE experiments with a minimum of 8 mice per group in each experiment, total WT n=55, JAM-A CKO n=37, JAM-A KO n=38. (B-D) Average (B),peak (C) and cumulative (D) scores of the EAE trial shown in (A) were significantly lower in CKO and KO mice compared to WT (average score: 2.0 (WT) vs. 1.36 (CKO) vs. 1.28 (KO), WT vs. CKO: p=0.03, WT vs. KO: p=0.01, CKO vs. KO: p>0.999; peak score: 3.6 (WT) vs. 2.7 (CKO) vs. 2.6 (KO), WT vs. CKO: p=0.003, WT vs. KO: p=0.002, CKO vs. KO: p>0.999; cumulative score: 45.58 (WT) vs. 31.75 (CKO) vs. 28.2 (KO), WT vs. CKO: p=0.003, WT vs. KO: p=0.002, CKO vs. KO: p>0.999 , Kruskal-Wallis tests. Average in bar graphs shown with SEM. (E) Survival curves of mortality revealed that WT mice sustained greater mortality over the course of EAE than CKO or KO, Mantel-Cox test, p<0.0001. (F) At Day 28, mortality rate was higher in WT mice compared to CKO and KO (0.41 (WT) vs. 0 (CKO) vs. 0.26 (KO), p<0.0001, Kruskal-Wallis test). (G) Disease curves demonstrated no differences in susceptibility to or timing of disease, Mantel-Cox test, p=0.65. (H) At Day 28, rates of disease resistance (proportion of mice that did not develop neurological deficit) showed an increased trend that was not statistically significant for CKO and KO compared to WT (0.014 (WT) vs 0.108 (CKO) vs. 0.131 (KO), p=0.22, Kruskal-Wallis test).

For Review Only

(I) WT mice with EAE treated with daily intraperitoneal injection of a JAM-A blocking peptide (JAM-Ap) from day 7 post-immunization showed a milder course of clinical disability compared to scramble peptide treated controls (scramble vs. JAM-A: $p=0.04$ by non-parametric Kolmogorov-Smirnov test comparing cumulative distributions; at Day 13-15, $p<0.0001$, day 16-17, $p<0.05$, day 20-21, $p<0.05$; total JAM-Ap $n=24$, scramble $n=26$, two-way ANOVA with Bonferroni correction; graph shows pooled data from 3 independent EAE experiments with a minimum of 8 mice per group for each experiment.).

For Review Only

Figure 5

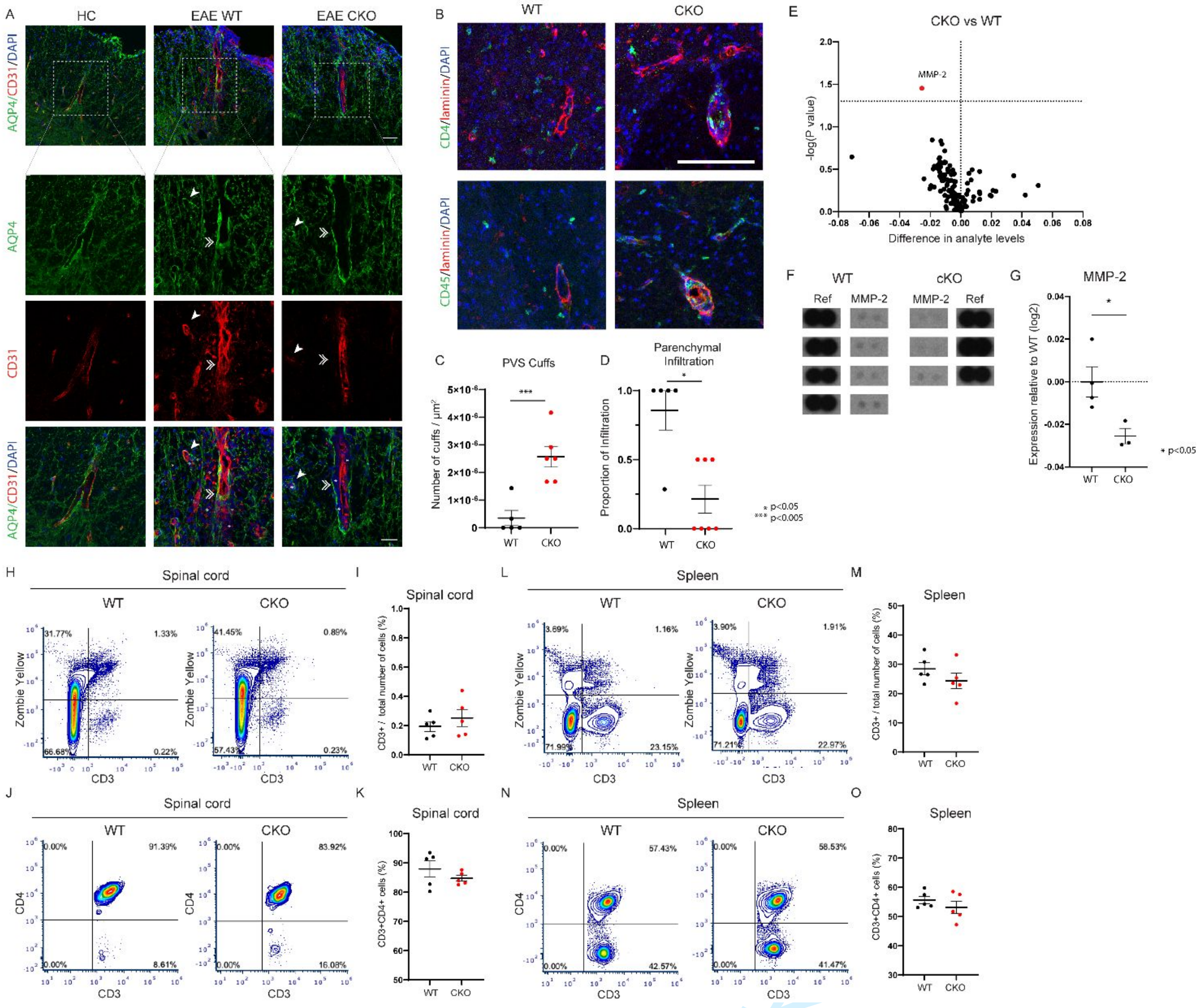
A
HC
EAE WT
EAE CKO
AQP4/CD31/DAPI
AQP4
CD31
AQP4/CD31/DAPI
B
WT
CKO
CD4/laminin/DAPI
CD45/laminin/DAPI
C
PVS Cuffs
Number of cuffs / µm²
WT
CKO
D
Parenchymal Infiltration
Proportion of Infiltration
* p<0.05
*** p<0.005
E
CKO vs WT
MMP-2
-log(P value)
Difference in analyte levels
F
WT
cKO
Ref
MMP-2
G
MMP-2
Expression relative to WT (log2)
* p<0.05
H
Spinal cord
Zombie Yellow
CD3
I
Spinal cord
CD3+ / total number of cells (%)
L
Spleen
M
Spleen
J
Spinal cord
CD4
K
Spinal cord
CD3+CD4+ cells (%)
N
Spleen
O
Spleen

Figure 5: Astrocytic JAM-A promotes T lymphocyte entry into the CNS parenchyma from the perivascular spaces via MMP-2 in EAE. (A) High power images of the neurovascular unit and perivascular spaces were identified using aquaporin-4 staining of the astrocyte endfeet and CD31 staining of the endothelium in HC, WT and CKO mice. Cell infiltrates (DAPI, blue) were seen in the CNS at 5 days post EAE disease onset in both WT and CKO but not in HC. Representative images demonstrate that in JAM-A CKO, immune cells accumulated within the perivascular spaces (between aquaporin-4 and CD31) whereas in WT, immune cells localized diffusely past the perivascular spaces within the CNS parenchyma. Scale bar 50 µm top panels, 30 µm bottom panels representing inset outlined by dotted white box. (B)In inflammatory lesions of EAE at Day 21 post-immunization, in CKO mice, $CD4^+$ (green, upper panel) and $CD45^+$ cells (green, lower panel) clustered in perivascular (PVS) "cuffs," colocalizing with the pan-laminin marker (in red), whereas in WT mice, $CD4^+$ and $CD45^+$ cells were instead located in the parenchyma. Scale bar 100 µm. (C) Number of $CD4^+$ cuffs per μm^2 of lumbar spinal cord cross-sections were increased in JAM-A CKO mice compared to WTs (average 3.5 x 10^{-7} (WT) vs. 2.6 x 10^{-6} (CKO), p=0.0022, Mann Whitney test, number of mice: WT n=5, CKO n=6). In the WT, the number of analyzed cuffs per mouse ranged from 0 to 6 (average 1.4); in the CKO, the range was 0 to 10 (average 4.8). (C) Proportion of sections demonstrating parenchymal infiltration of $CD4^+$ cells was lower in JAM-A CKO compared to WT mice (average 0.86 (WT) vs. 0.21 (CKO), p=0.01, Mann Whitney test). (E) A Volcano Plot shows the differential level of 111 pro-inflammatory cytokines, chemokines, proteases and acute phase reactants in spinal cord lysates of JAM-A CKO and WT mice at 5 days post EAE disease onset as measured using mouse proteome ELISA immunoassays. CKO mice showed an overall reduction of many factors, though MMP-2 (highlighted in red) was the sole statistically significant factor compared to WT controls. (F)

For Review Only

MMP-2 probes on the ELISA array in CKO and WT mice, along with reference spots, used for signal (pixel intensity) normalization, are shown. Complete ELISA arrays from each mouse are shown in **Supplementary Figure 7** and original blots are included in the **Supplementary Materials**. (G) Spinal cord levels of MMP-2 in CKO mice relative to WT controls were significantly decreased at 5 days post EAE disease onset (relative log2 expression -0.025, p=0.0351, two-tailed unpaired t-test, WT, n=4; CKO, n=3). (H-O) Flow cytometry was performed on spinal cords and spleens of WT and CKO mice with EAE on day 5 from onset of disease. (H) Representative plots show (I) similar total $CD3^+$ cell counts in CKO and WT (mean CKO 0.23 vs. WT 0.19%, p= 0.42, unpaired two-tailed t-test; n=5 WT, n=5 CKO, average EAE score WT 2.8, CKO 2.6 for all flow experiments). (J) Representative plots reflect (K) no difference found in the proportion of $CD3^+CD4^+$ cells in CKOs compared to WTs (mean CKO 84.73% vs. 87.97%, p=0.29, unpaired two-tailed t-test). (L-O) In spleen, total $CD3^+$ and $CD3^+CD4^+$ were unchanged between CKO and WT groups ($CD3^+$: , mean CKO 24.3% vs. WT 28.4%, p=0.25; $CD3^+CD4^+$: mean CKO 53.1% vs. 55.6%, p=0.34unpaired two-tailed t-test).

For Review Only

Figure 6

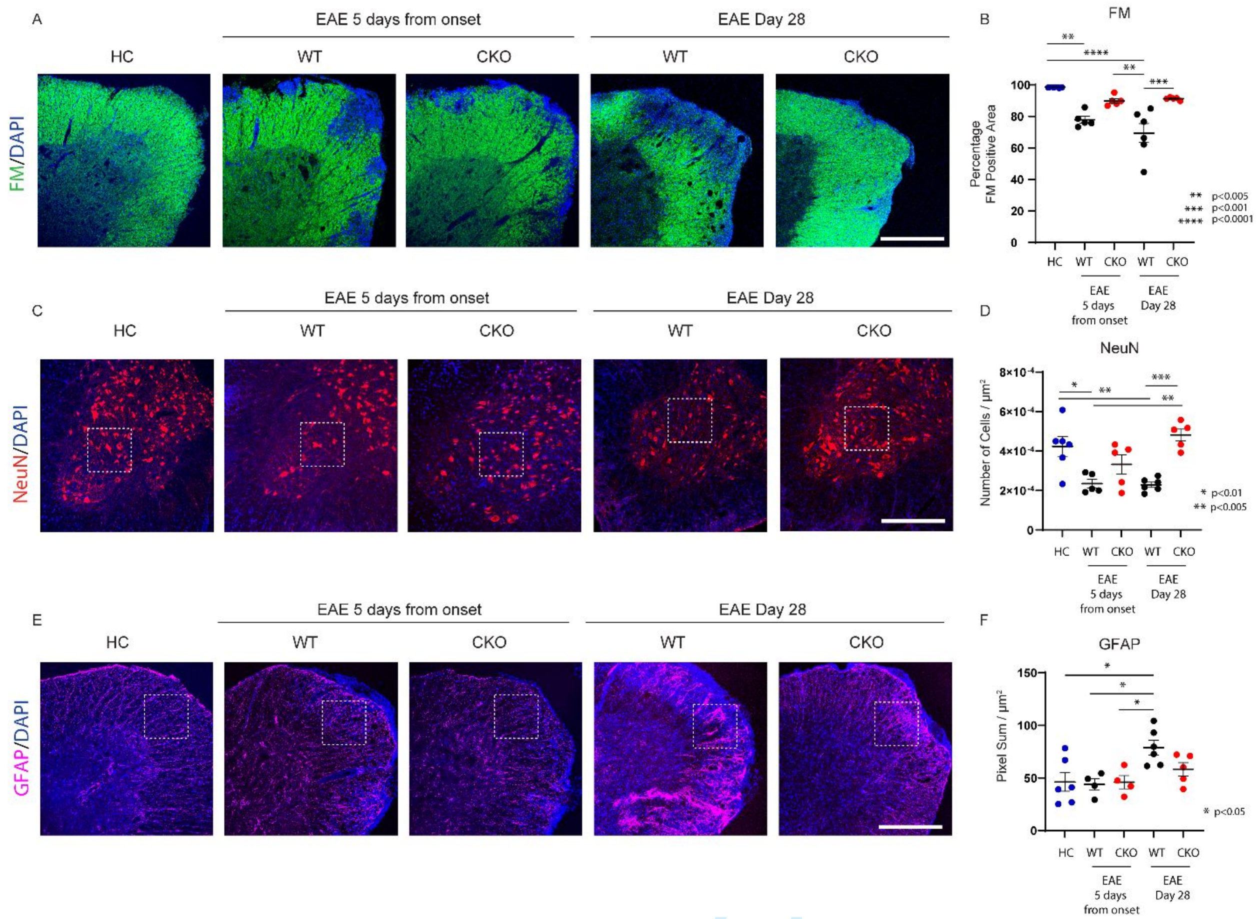

Only

Figure 6: Astrocytic JAM-A exacerbates histopathological markers of neuroinflammatory damage in EAE. (A, B) Proportion of fluoromyelin (FM, green; marker of myelin) positive area of the lumbar anterolateral white matter tracts was significantly increased in EAE CKO mice at a late chronic stage of disease (EAE Day 28 post immunization) compared to time matched WT mice controls (EAE WT Day 28 post immunization) and EAE WT in the acute phase of disease (EAE WT 5 days from onset) (average HC 98.53% vs. EAE WT 5 days from disease onset 77.85% vs. EAE CKO 5 days from disease onset 89.91% vs. EAE WT Day 28 post immunization 69.35% vs. EAE CKO Day 28 post immunization 91.2%; HC vs. EAE WT 5 days: $p=0.001$, HC vs. EAE WT Day 28: $p<0.0001$, EAE CKO 5 days vs. EAE WT Day 28: $p=0.001$, EAE WT Day 28 vs. EAE CKO Day 28: $p=0.0007$, all other p values>0.05, one way ANOVA with Tukey's multiple comparison test). HC n=6, EAE WT 5 days from disease onset n=5, EAE CKO 5 days from disease onset n=5, EAE WT Day 28 post immunization n=6, EAE CKO Day 28 post immunization n=5 animals. Scale bar 400 µm. (C, D) Number of $NeuN^+$ cells per μm^2 within the ventral gray matter of the lumbar spinal cord was significantly higher in EAE CKO mice at day 5 after disease onset and at Day 28 post immunization than in time-matched WT controls (average HC 4.2×10^{-4} vs. EAE WT 5 days from disease onset 2.35×10^{-4} vs. EAE CKO 5 days from disease onset 3.32×10^{-4} vs. EAE WT Day 28 post immunization 2.29×10^{-4} vs. EAE CKO Day 28 post immunization 4.82×10^{-4}; HC vs. EAE WT 5 days: $p=0.01$, HC vs. EAE WT Day 28: $p=0.005$, EAE WT 5 days vs. EAE WT Day 28: $p=0.001$, EAE WT Day 28 vs. EAE CKO Day 28: $p=0.0005$, all other p values>0.05, one way ANOVA with Tukey's multiple comparison test). HC n=6, WT EAE 5 days from disease onset n=5, CKO 5 days from disease onset n=5, WT EAE Day 28 post induction n=6, EAE CKO Day 28 post induction n=5 animals. Scale bar 100 µm. In the WT, a range of 3-15 cells (average 9.8) were analyzed per mouse; in the CKO, a range was 15

For Review Only

to 45 cells (average 25.6) were analyzed per mouse. (E, F) GFAP (purple, marker of astrocytes) positive pixel sum per μm^2 of the lumbar anterolateral white matter tracts showed a decreasing trend at day 28 post EAE immunization in CKO compared to WT (average HC 46.24 vs. EAE WT 5 days from disease onset 43.9 vs. EAE CKO 5 days from disease onset 46.04 vs. EAE WT Day 28 post immunization 78.96 vs. EAE CKO Day 28 post immunization 58.38; HC vs. WT Day 28: p=0.01, EAE WT 5 days vs. EAE WT Day 28: p=0.04, EAE WT Day 5 vs. EAE WT Day 28: p=0.04, all other p values>0.05, one way ANOVA with Tukey's multiple comparison test). HC n=6, EAE WT 5 days from disease onset n=4, EAE CKO 5 days from disease onset n=4, WT EAE Day 28 post induction n=6, EAE CKO Day 28 post induction n=5 animals. Scale bar 100 μm.

For Review Only

Supplemental Figure 1

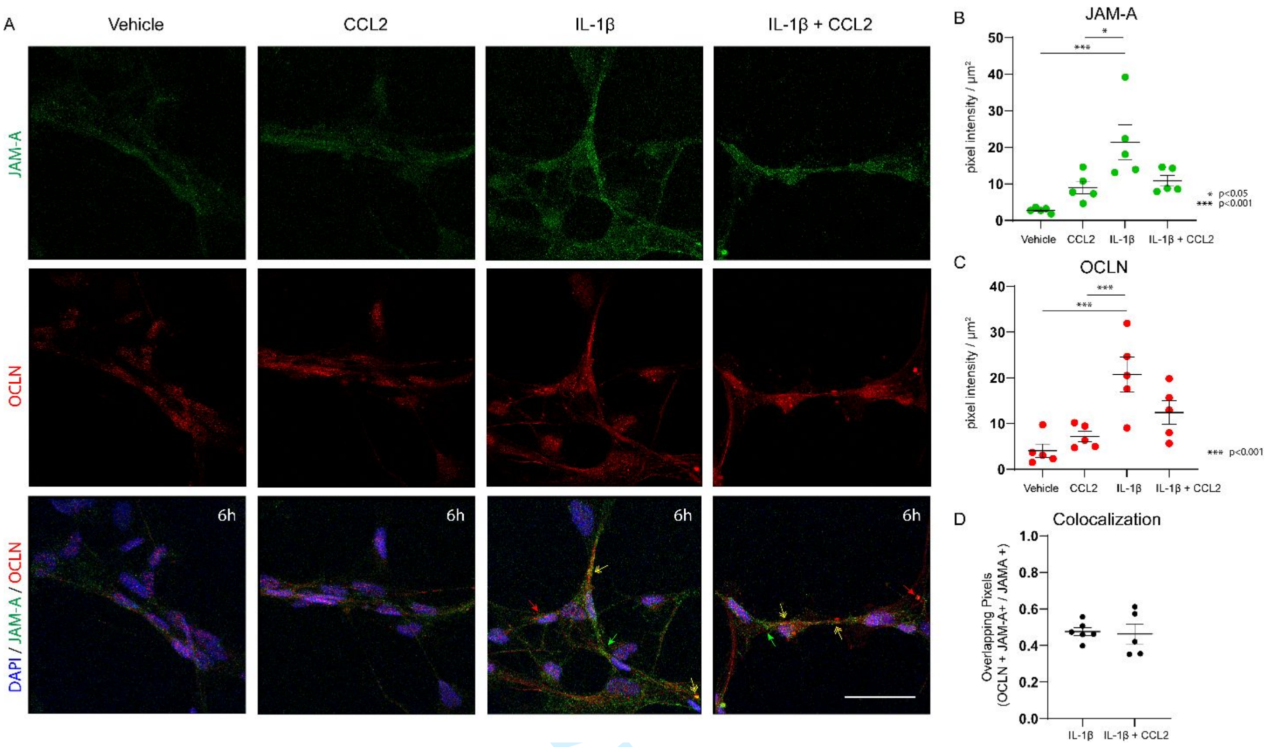

For Review Only

Supplemental Figure 1: Spatial dynamics of astrocytic JAM-A and OCLN induction *in vitro* at 6 hours. (A-B) Astrocytic JAM-A (green) was induced *in vitro* at 6 hours after treatment with 20ng/mL IL-1β alone but not after treatment with 100ng/mL CCL2 or the combination of IL-1β and CCL2 (average vehicle (2.826) vs. IL-1β (21.34) vs. CCL2 (9.015) vs. IL-1β + CCL2 (10.83), vehicle vs. IL-1β: $p=0.0007$, CCL-2 vs. IL-1β: $p=0.02$, other comparisons $p>0.05$, analyzed images n=5 all groups, one way ANOVA with Tukey's multiple comparison test). JAM-A showed a similar localization pattern as seen at 24 hours (**Figure 1A-E**): both diffusely localized throughout the cell membrane (green arrows) and co-localized with the tight junction marker, occludin (OCLN, red, red arrows; yellow arrows pointing to areas of JAM-A$^+$ and OCLN$^+$ overlapping pixels. All results were quantified from at least 4 fields of view from two to three technical replicates per group. Scale bar 50 µm. (C) Astrocytic occludin was similarly induced at 6 hours after treatment with IL-1β but not CCL2 or the combination of IL-1β and CCL2 (average vehicle (4.058) vs. IL-1β (20.76) vs. CCL2 (7.137) vs. IL-1β + CCL2 (12.41), vehicle vs. IL-1β: $p=0.001$, CCL-2 vs. IL-1β: $p=0.0062$, other comparisons $p>0.05$, n=5 all groups, one way ANOVA with Tukey's multiple comparison test). (D) The addition of CCL2 to IL-1β did not change the proportion of JAM-A$^+$ pixels colocalized with OCN$^+$ pixels at 6 hours. (IL-1β (0.476) vs. IL-1β + CCL2 (0.4632), $p=0.81$, analyzed images n=6 IL-1β, n=5 IL-1β + CCL2, unpaired two-tailed t-test.

For Review Only

Supplemental Figure 2

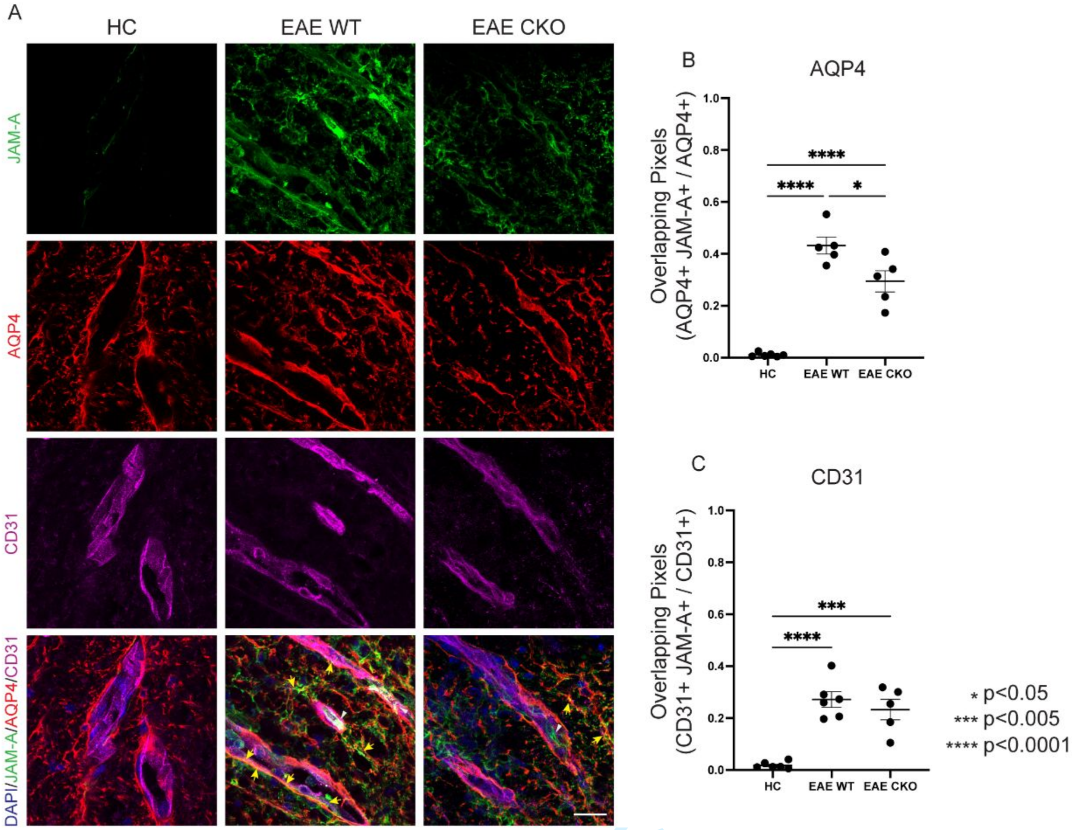

For Review Only

Supplemental Figure 2: JAM-A is induced in both endothelial cells and the astrocytic endfeet during EAE. (A) Images show immunohistochemistries for JAM-A (green), AQP4 (red, marker of astrocytic endfeet), CD31 (purple, marker of endothelial cells), and DAPI (blue) in the spinal cord of healthy control (HC), and WT and CKO mice with EAE 5 days from disease onset (EAE WT and EAE CKO, respectively). Colocalization of JAM-A with AQP4 (yellow arrows) and CD31 (white arrowheads) is observed in the spinal cord of mice with EAE but not in HCs. Scale bar = 20 µm. (B) Proportion of $AQP4^{+}$ pixels colocalizing with JAM-A^{+} pixels demonstrate JAM-A expression in astrocytic endfeet ($AQP4^{+}$) during EAE (HC vs EAE WT and HC vs EAE CKO: $p < 0.0001$), which is decreased in EAE CKO compared to EAE WT (EAE WT vs EAE CKO: $p = 0.0139$). (C) Proportion of endothelial cells ($CD31^{+}$) positive for JAM-A is increased during EAE compared to HCs (HC vs EAE WT, $p < 0.0001$; HC vs EAE CKO, $p = 0.002$). No differences in the proportion of JAM-A^{+} and $CD31^{+}$ overlapping pixels were observed between EAE WT and EAE CKO mice ($p > 0.05$). Analyses in (B-C) one way ANOVA with Tukey's multiple comparison test. HC, n=6; EAE WT, n=5; EAE CKO, n=5.

For Review Only

Supplemental Figure 3

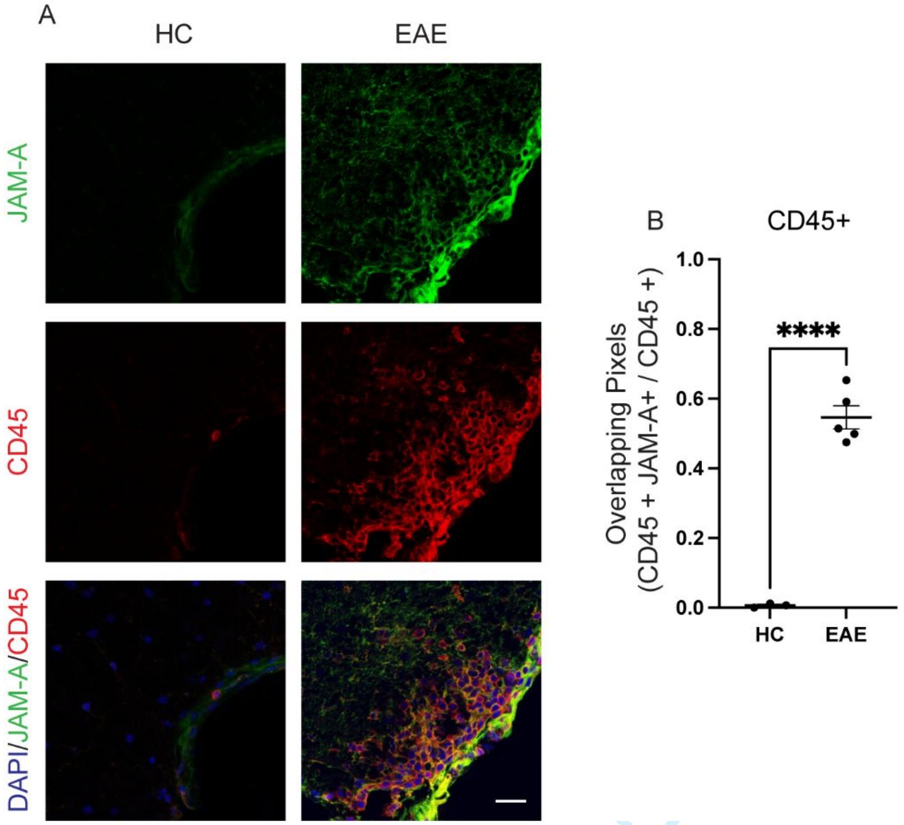

w Only

Supplemental Figure 3: JAM-A is expressed by CD45$^+$ immune cells infiltrating the spinal cord of mice with EAE. (A) Immunohistochemistry for JAM-A (green), CD45 (red, a marker of immune cells), and DAPI (blue) in the spinal cord dorsal column of mice with EAE 5 days from disease onset and in healthy controls (HC). Scale bar = 20 µm. (B) Colocalization analysis of CD45 and JAM-A shows that the proportion of CD45$^+$ pixels overlapping with JAM-A$^+$ pixels increases in EAE mice compared to HC mice ($p<0.0001$, unpaired two-tailed t-test). HC, n=3; EAE, n=5.

For Review Only

Supplemental Figure 4

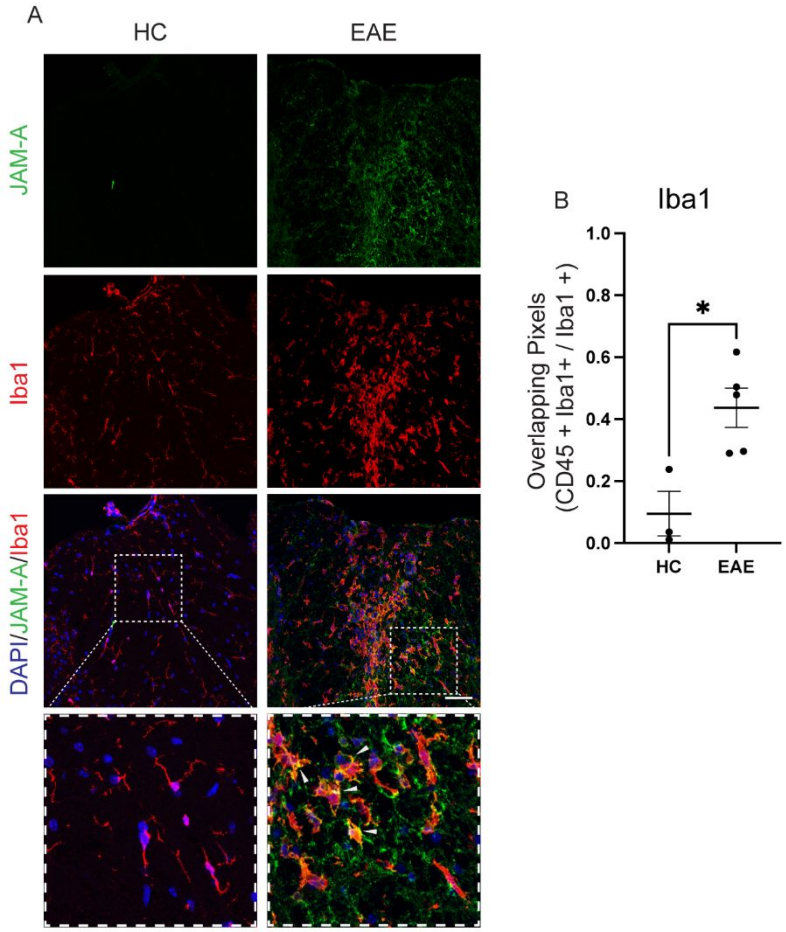

Supplemental Figure 4: JAM-A is expressed by Iba^{+} microglia in the spinal cord of mice with EAE. (A) Images show immunohistochemistries for JAM-A (green), Iba1(red, marker of microglia), and DAPI (blue) in the spinal cord dorsal column of mice with EAE and healthy controls (HC). JAM-A expression is absent in the spinal cord of HC mice, while it is upregulated in mice with EAE. Iba1 positive microglia express JAM-A in areas of inflammation in the spinal cord of mice with EAE (white arrowheads). Scale bar = 50 µm. (B) Colocalization analysis of Iba1 with JAM-A shows that the proportion of Iba1^{+} pixels overlapping with JAM-A^{+} pixels increases in EAE mice compared to HC mice (p=0.0138, unpaired t-test). HC, n=3; EAE, n=5.

For Review Only

Supplemental Figure 5

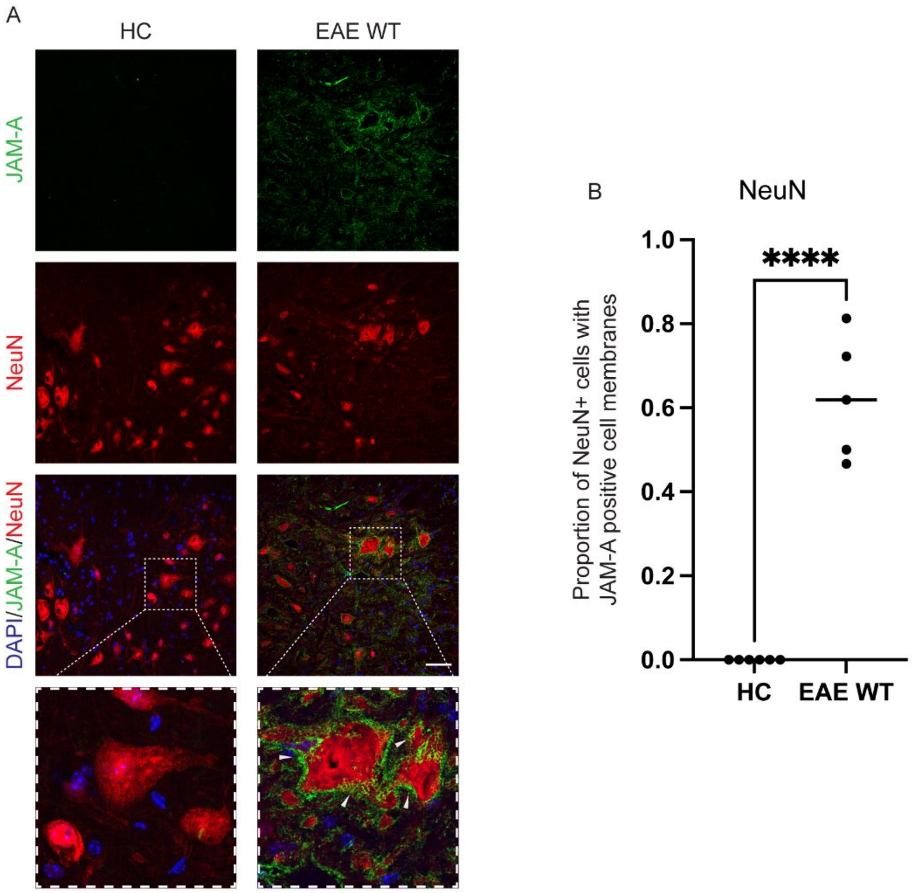

Only

Supplemental Figure 5: Spinal neurons express JAM-A during EAE. (A) Images show immunohistochemistries for JAM-A (green), NeuN (red, marker of neurons' cell body), and DAPI (blue) in the spinal cord ventral horn of healthy control (HC) and WT mice at 5 days from EAE disease onset (EAE WT). JAM-A expression is minimally detectable in the spinal cord of HC mice, while it is upregulated in EAE and expressed on the cell surface of neurons of the spinal cord ventral horn (white arrowheads). Scale bar = 50 µm. (B) Quantification of the number of JAM-A^+ neurons relative to the total number of neurons ($NeuN^+$) in HC and EAE WT mice showed increases in EAE WT compared to HC ($p<0.0001$, unpaired two-tailed t-test). HC, n=6; EAE WT, n=5.

For Review Only

Supplemental Figure 6

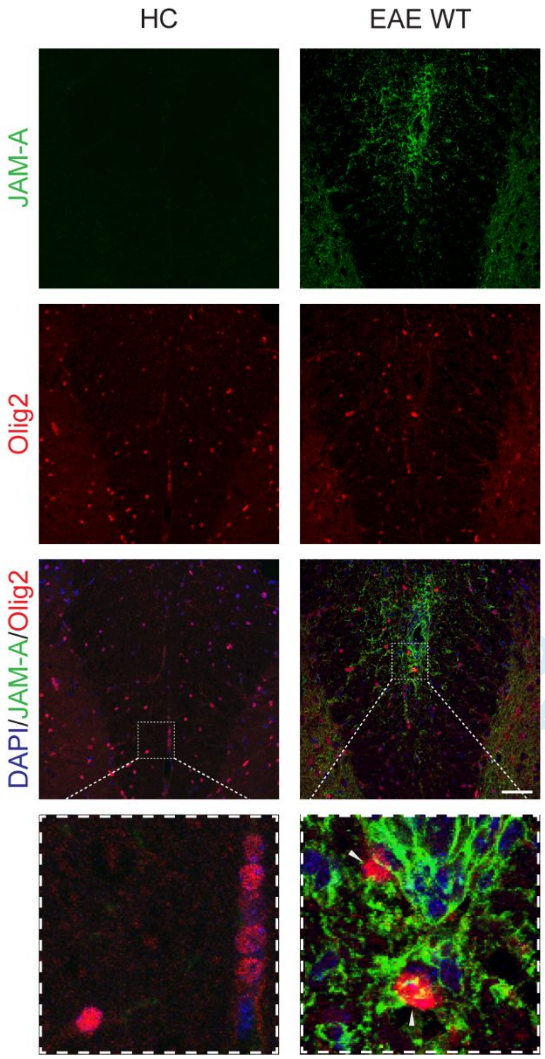

For Peer Review Only

Supplemental Figure 6: JAM-A expression is not clearly detected in oligodendrocytes on immunohistochemistry during EAE. (A) Images show immunohistochemistries for JAM-A (green), Olig2 (red, marker of oligodendrocytes), and DAPI (blue) in the spinal cord dorsal column of healthy control (HC), and WT mice at 5 days from EAE disease onset (EAE WT). JAM-A expression is absent in the spinal cord of HC mice, while it is upregulated in EAE and expression appears to be separated from the cell surface of the few oligodendrocytes within inflammatory lesions (white arrowheads) in EAE mice. More sensitive techniques, such as cell sorting, may be needed to determine whether oligodendrocytes express JAM-A. Scale bar = 50 μm.

For Review Only

Supplemental Figure 7

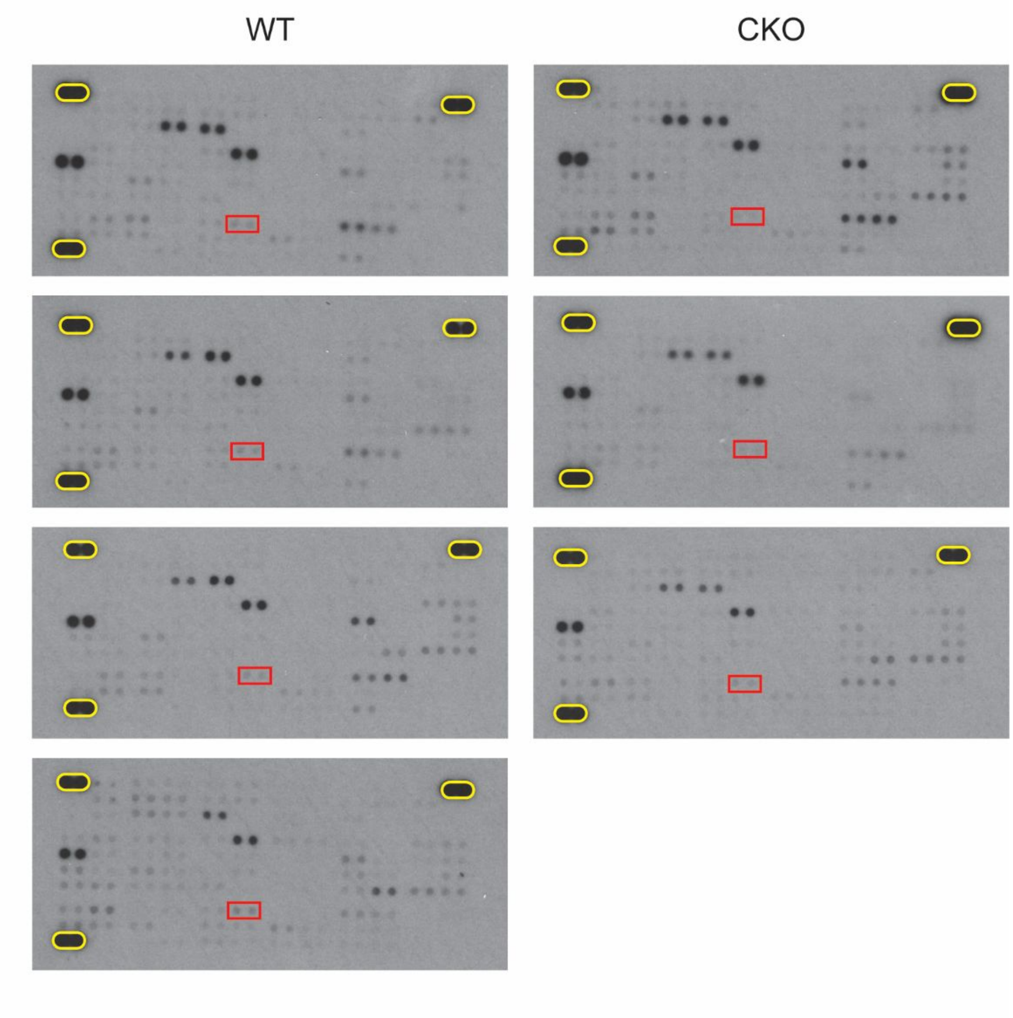

Supplemental Figure 7: Differential expression of MMP-2 is detected on proteome arrays of spinal cord lysates from JAM-A CKO and WT mice during EAE. Images show individual ELISA immunoarrays (WT, n=4; cKO, n=3) assessing the expression of 111 cytokines, chemokines, proteases and acute phase reactants in the spinal cord of mice at 5 days from EAE disease onset. Red rectangles highlight MMP-2 expression which was significantly decreased in CKO mice compared with WT controls, while yellow ovals demarcate the reference spots in each array. A minimal linear contrast enhancement step was performed uniformly across all original blot images prior to analysis. Original blot images are available in the Supplemental Materials.

For Review Only

Protease Array - astrocytes and lymphocytes sample 1

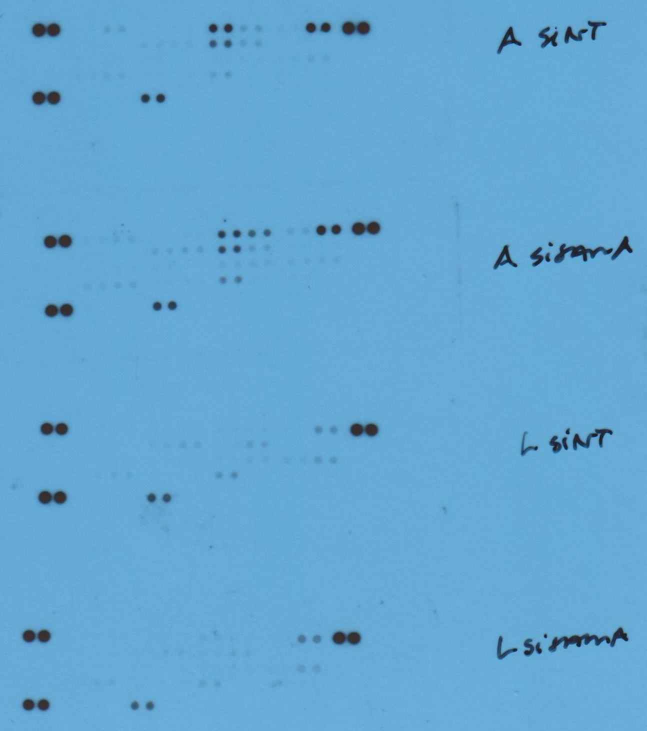

Protease array - astrocyte and lymphocyte sample 2

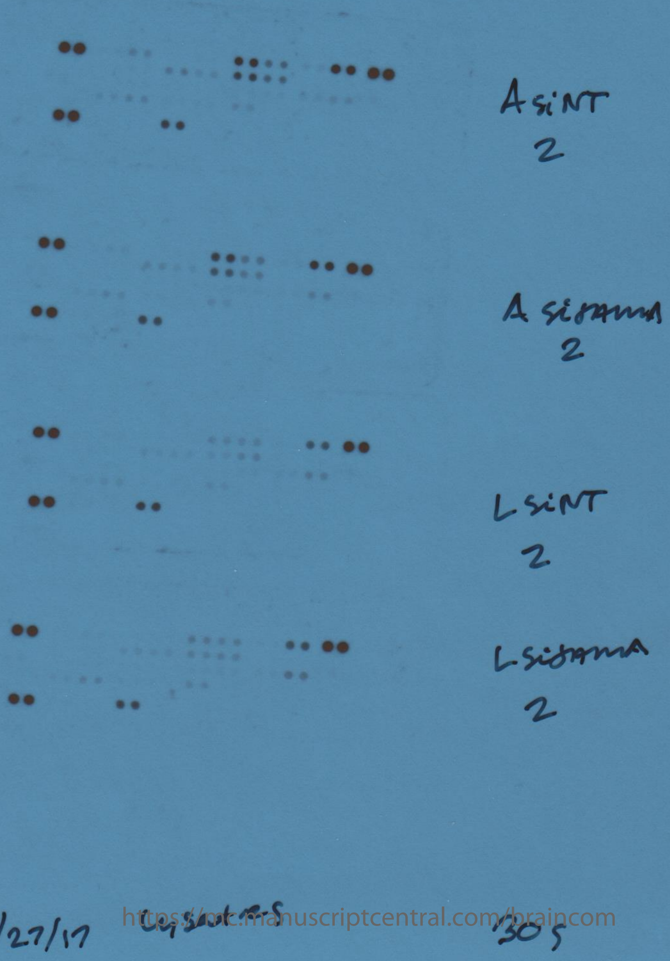

https://mc.manuscriptcentral.com/braincom

Protease

10 s

Protease array - astrocyte and lymphocyte sample 3

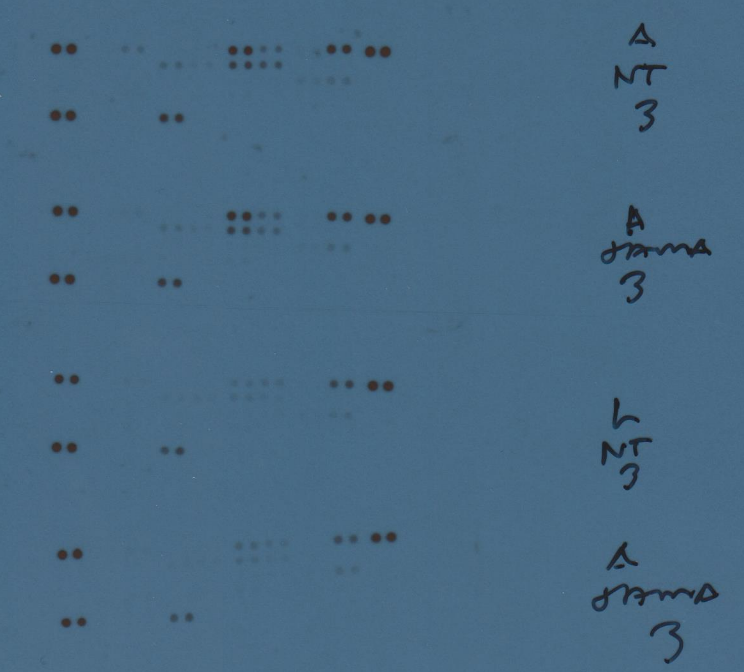

https://mc.manuscriptcentral.com/braincom

Protease array - supernatant sample 1 (biological sample 2")

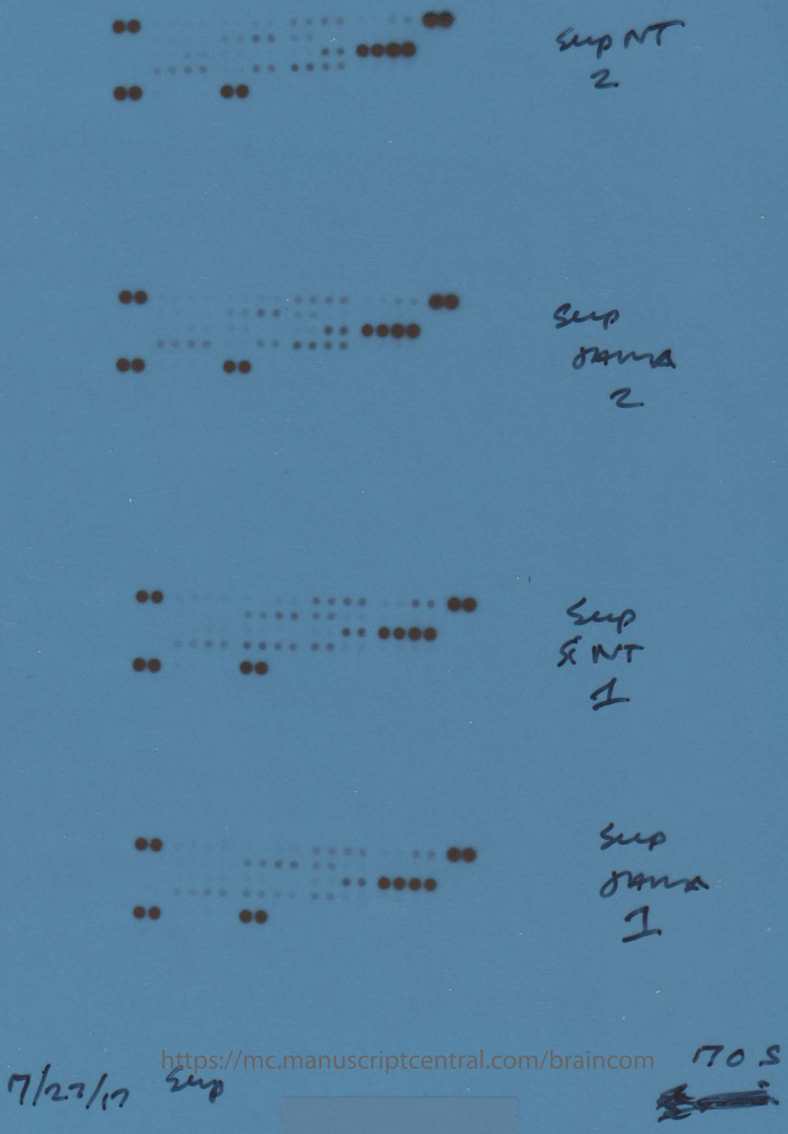

https://mc.manuscriptcentral.com/braincom

Protease array - supernatant samples 2 and 3 (biological samples "3" and "4")

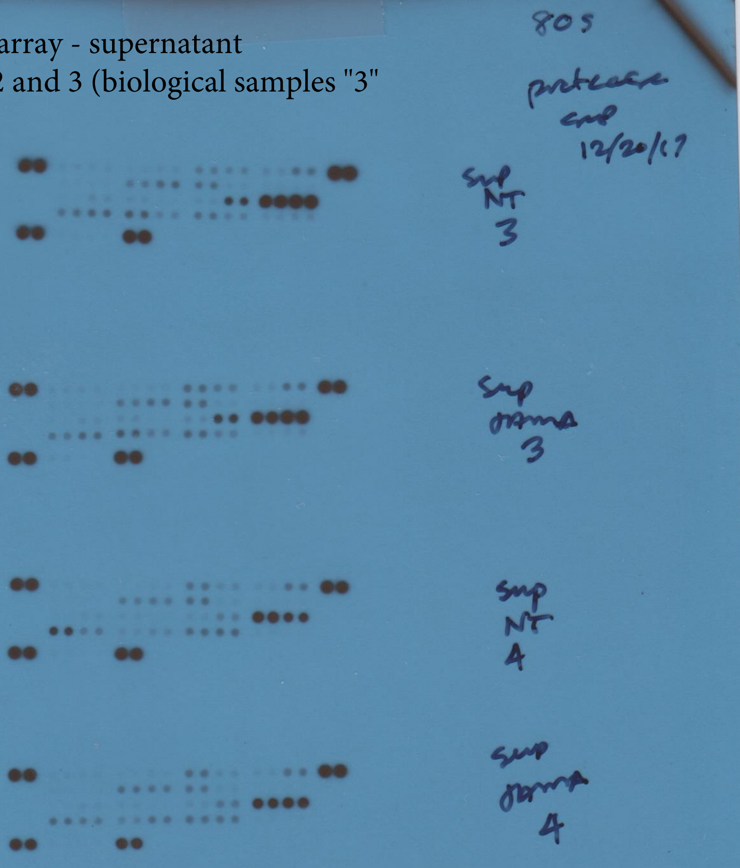

Cytokine array - supernatant
sample 1 and 2 (biological sample "2" and "3")

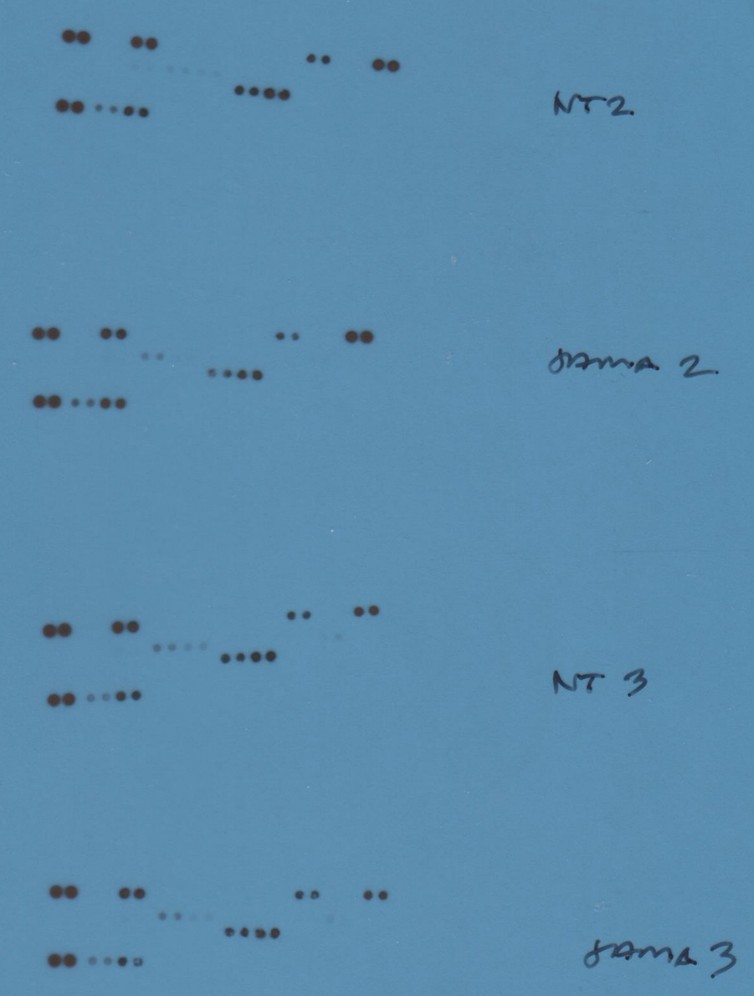

Cytokine array - supernatant sample 3 (biological sample "4")

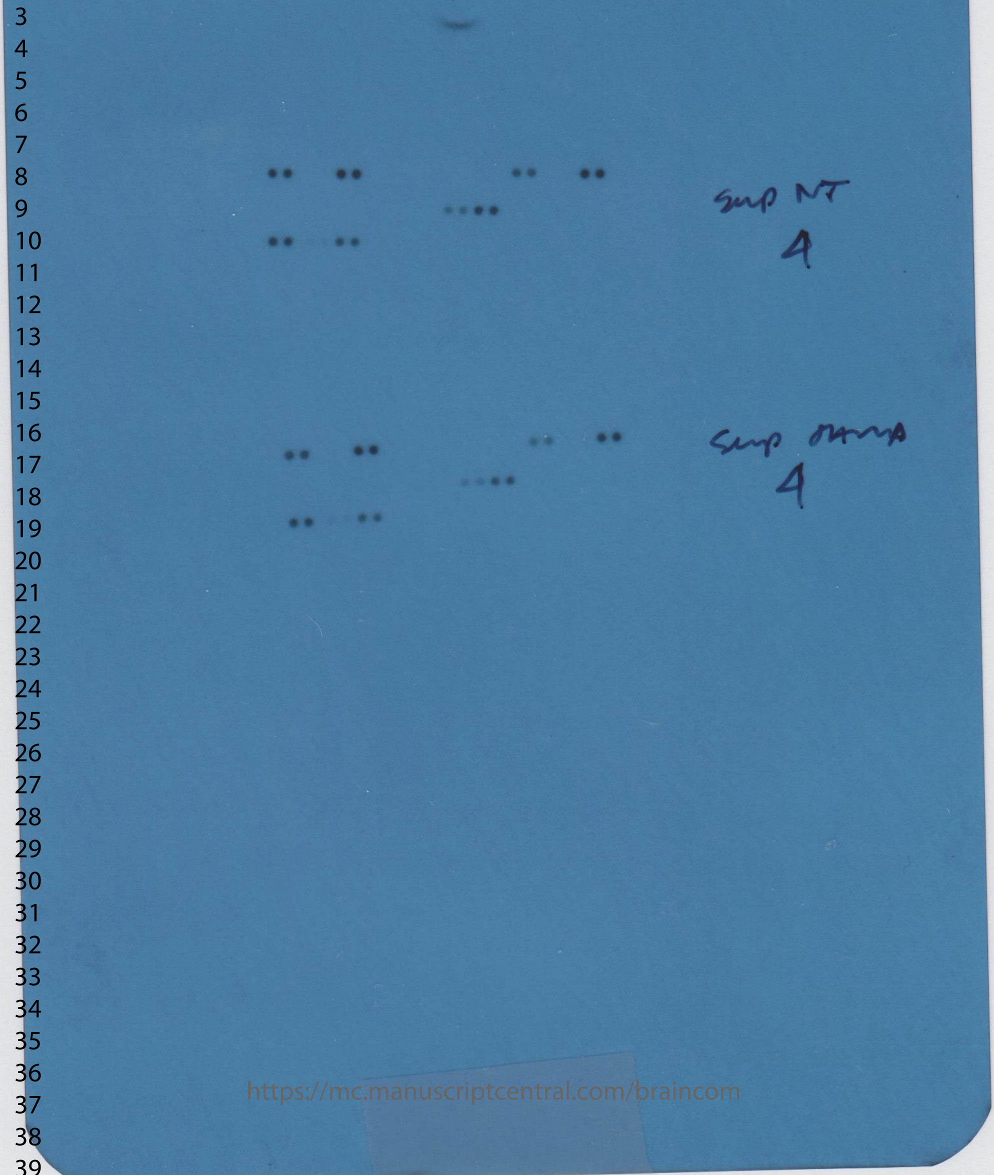

Cytokine array - astrocyte and lymphocyte sample 1

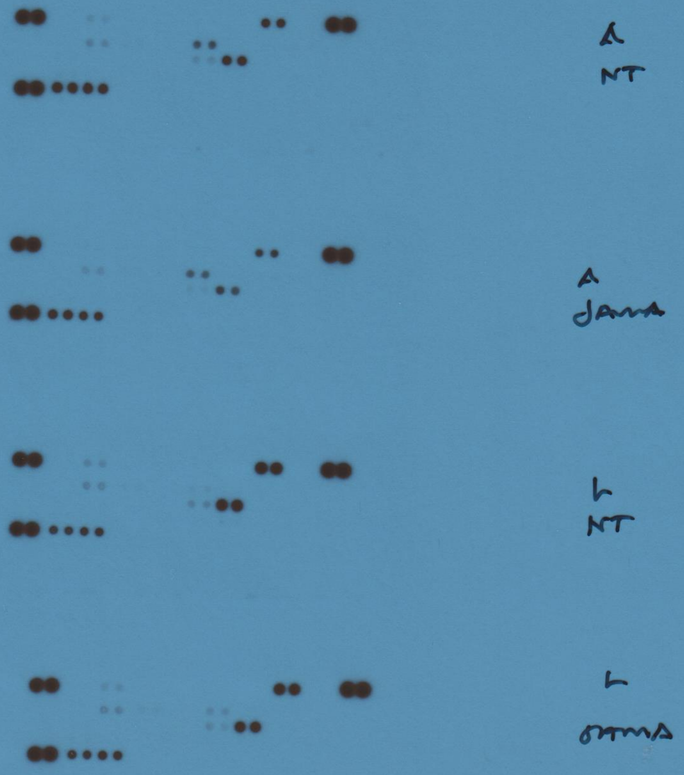

Cytokine array - astrocyte and lymphocyte sample 2

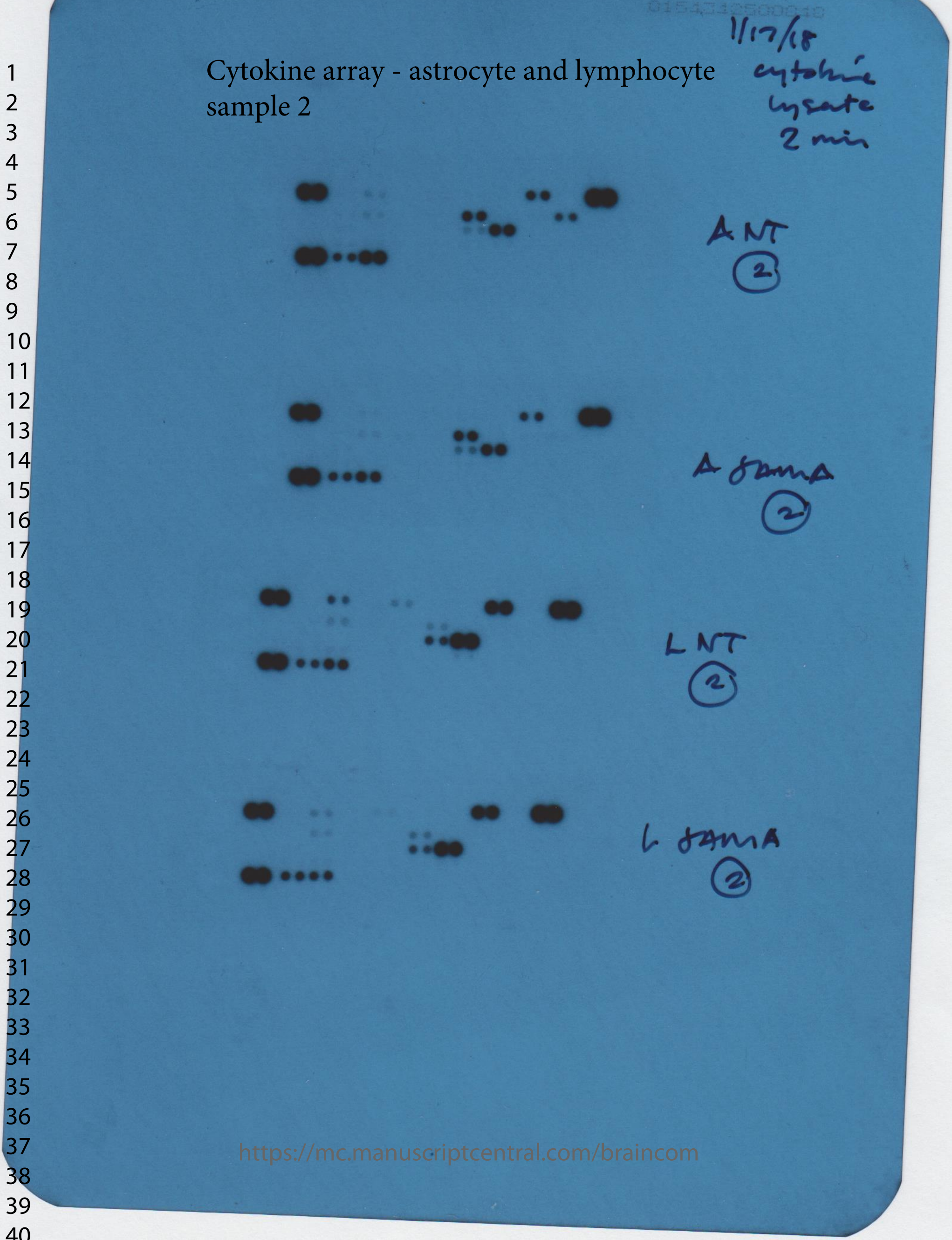

https://mc.manuscriptcentral.com/braincom

Cytokine array - astrocyte and lymphocyte sample 3 (biological sample "4")

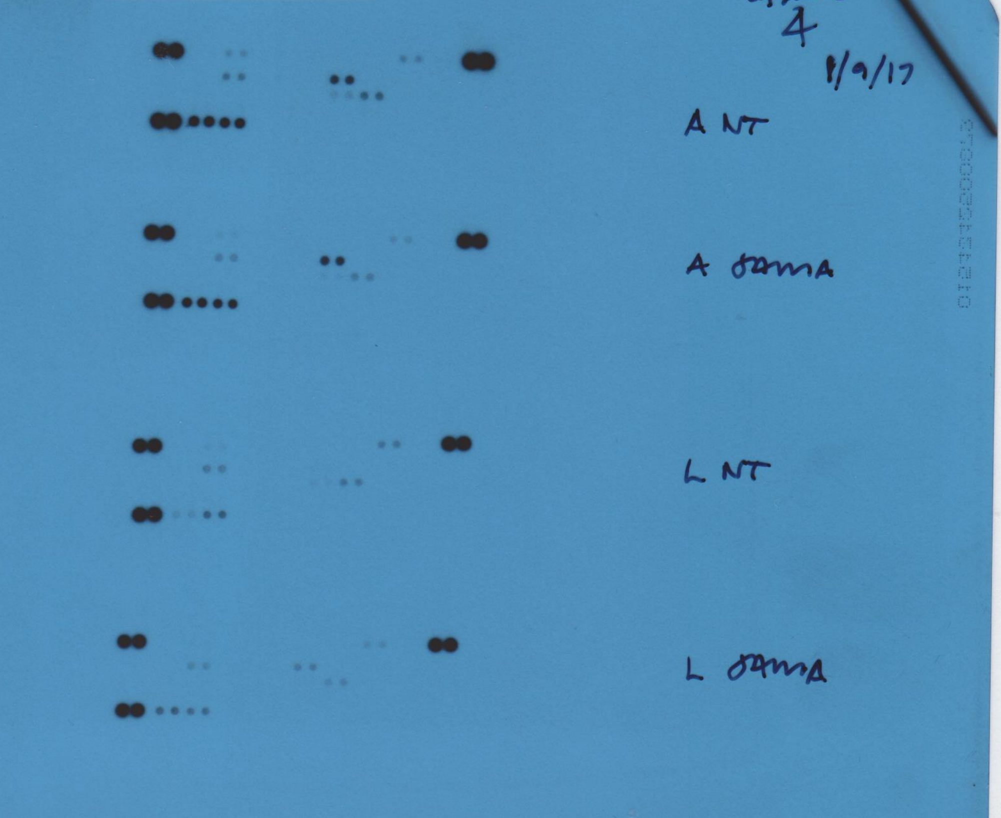

Proteome ELISA arrays - spinal cord lysates harvested 5 days from EAE disease onset

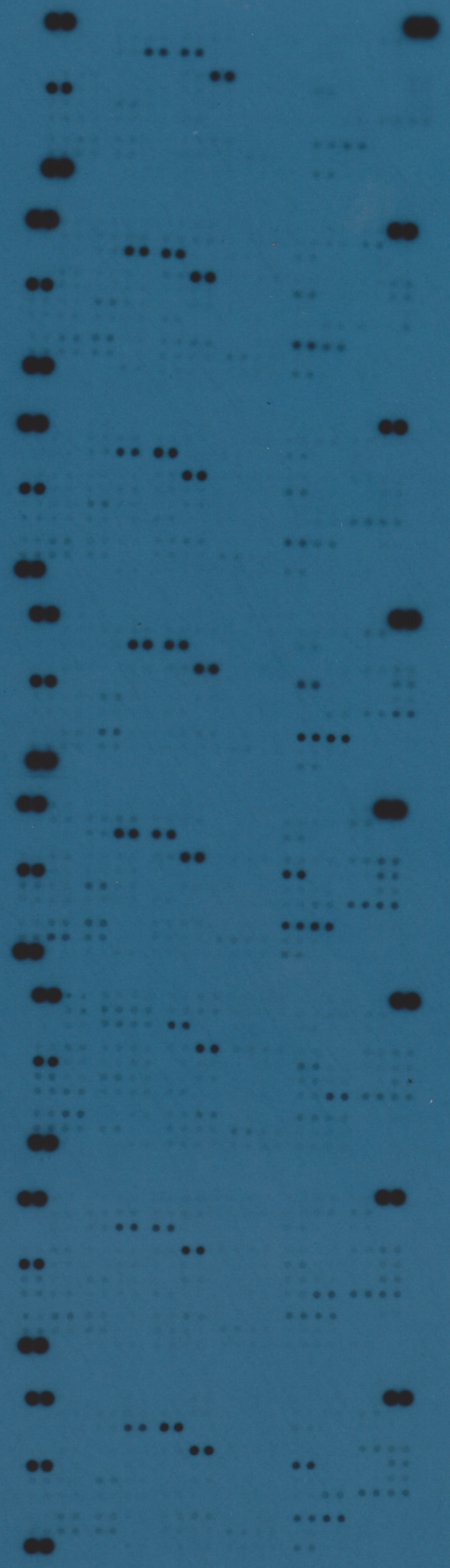

CKO 1

WT 1

WT 2

WT (excluded from analysis as tissue harvested at 7 days from EAE disease onset)

CKO 2

WT 3

CKO 3

WT 4
