# Supplementary material for: Astrocytic junctional adhesion molecule-A regulates T-cell entry past the glia limitans to promote central nervous system autoimmune attack
Source: Brain Commun. 2022 Feb 18;4(2):fcac044. doi: 10.1093/braincomms/fcac044 (PMC8899531; doi:10.1093/braincomms/fcac044)
Supplement: fcac044_Supplementary_Data [file fcac044_supplementary_data.zip › Supplementary_material.pdf]

Supplemental Figure 1

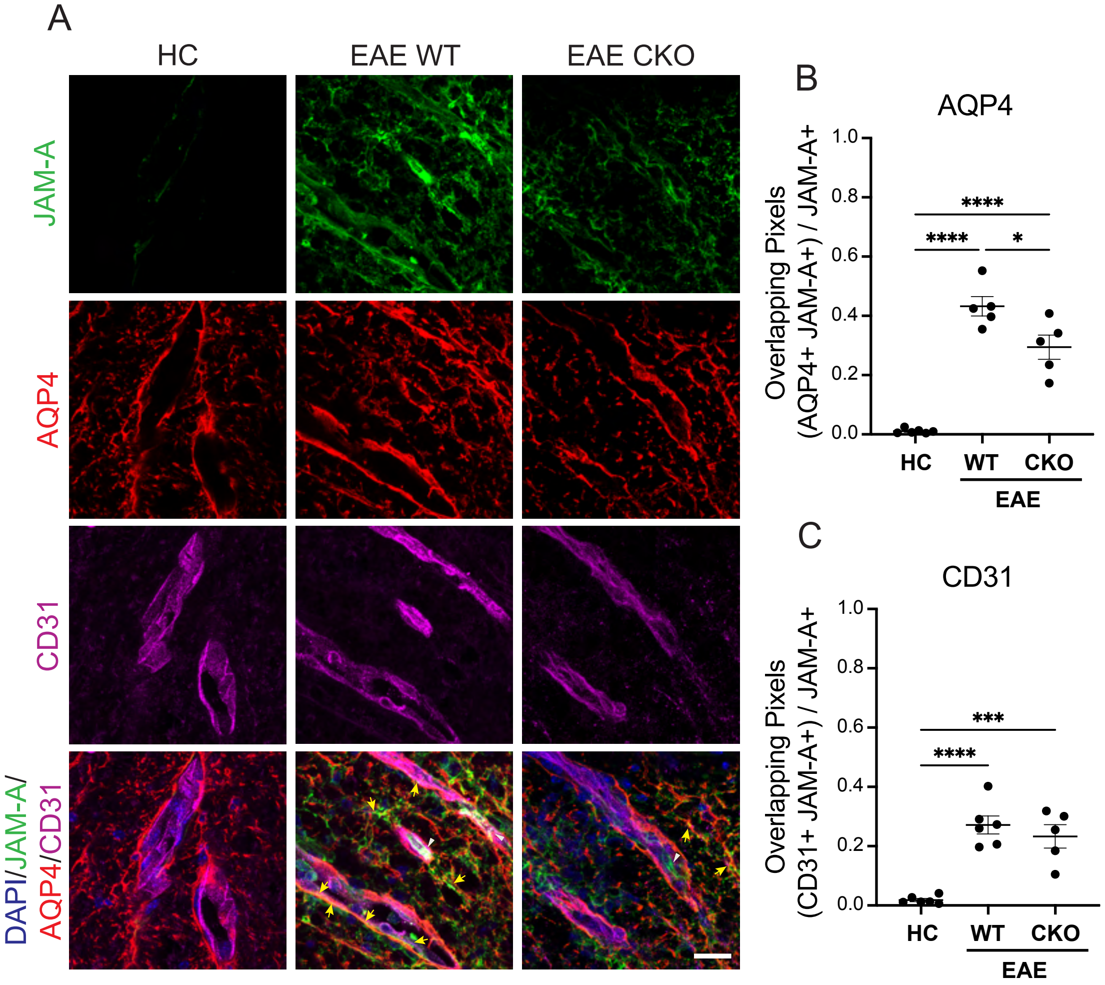

Supplemental Figure 1: JAM-A is induced in endothelial cells and astrocytic endfeet during EAE. (A) Images show immunohistochemistry for JAM-A (green), AQP4 (red, marker of astrocytic endfeet), CD31 (purple, marker of endothelial cells), and DAPI (blue) in the spinal cord of healthy control (HC), WT and CKO mice with EAE 5 days from disease onset (EAE WT and EAE CKO, respectively). Colocalization of JAM-A with AQP4 (yellow arrows) and CD31 (white arrowheads) is observed in the spinal cord of mice with EAE but not in HCs. Scale bar = 20 µm. (B) Proportion of AQP4+ pixels colocalizing with JAM-A+ pixels demonstrate JAM-A expression in astrocytic endfeet (AQP4+) during EAE (HC vs. EAE WT and HC vs. EAE CKO: $p < 0.0001$), which is decreased in EAE CKO compared to EAE WT (EAE WT vs. EAE CKO, $p = 0.0139$). (C) Proportion of endothelial cells (CD31+) positive for JAM-A is increased during EAE compared to HCs (HC vs EAE WT, $p < 0.0001$; HC vs. EAE CKO, $p = 0.0002$). No difference in the proportion of JAM-A+ and CD31+ overlapping pixels were observed between EAE WT and EAE CKO mice ($p > 0.05$). (B-C) One way ANOVA with Tukey's multiple comparison test. HC, n=6; EAE WT, n=5; EAE CKO, n=5.

Supplemental Figure 2

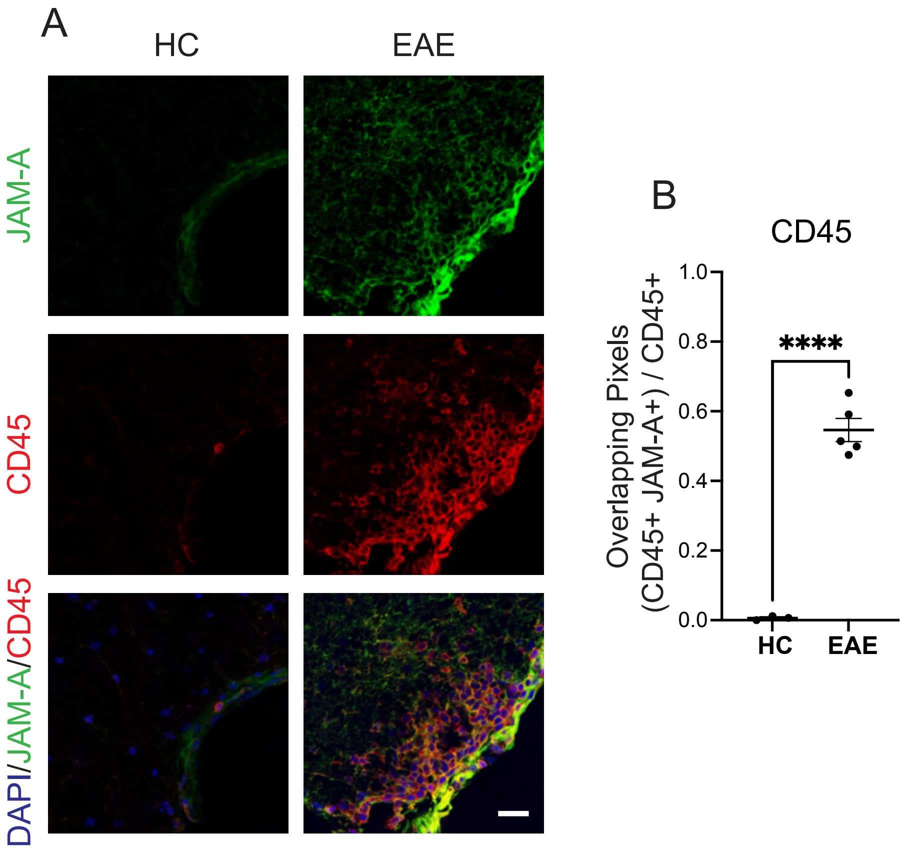

Supplemental Figure 2: JAM-A is expressed by CD45+ immune cells infiltrating the spinal cord of mice with EAE. (A) Immunohistochemistry for JAM-A (green), CD45 (red, a marker of immune cells), and DAPI (blue) in the spinal cord dorsal column of mice with EAE 5 days from disease onset and in healthy controls (HC). Scale bar = 20 µm. (B) Colocalization analysis of CD45 and JAM-A shows that the proportion of CD45+ pixels overlapping with JAM-A+ pixels increases in EAE mice compared to HC mice ($p<0.0001$, unpaired two-tailed t-test). HC, n=3; EAE, n=5.

Supplemental Figure 3

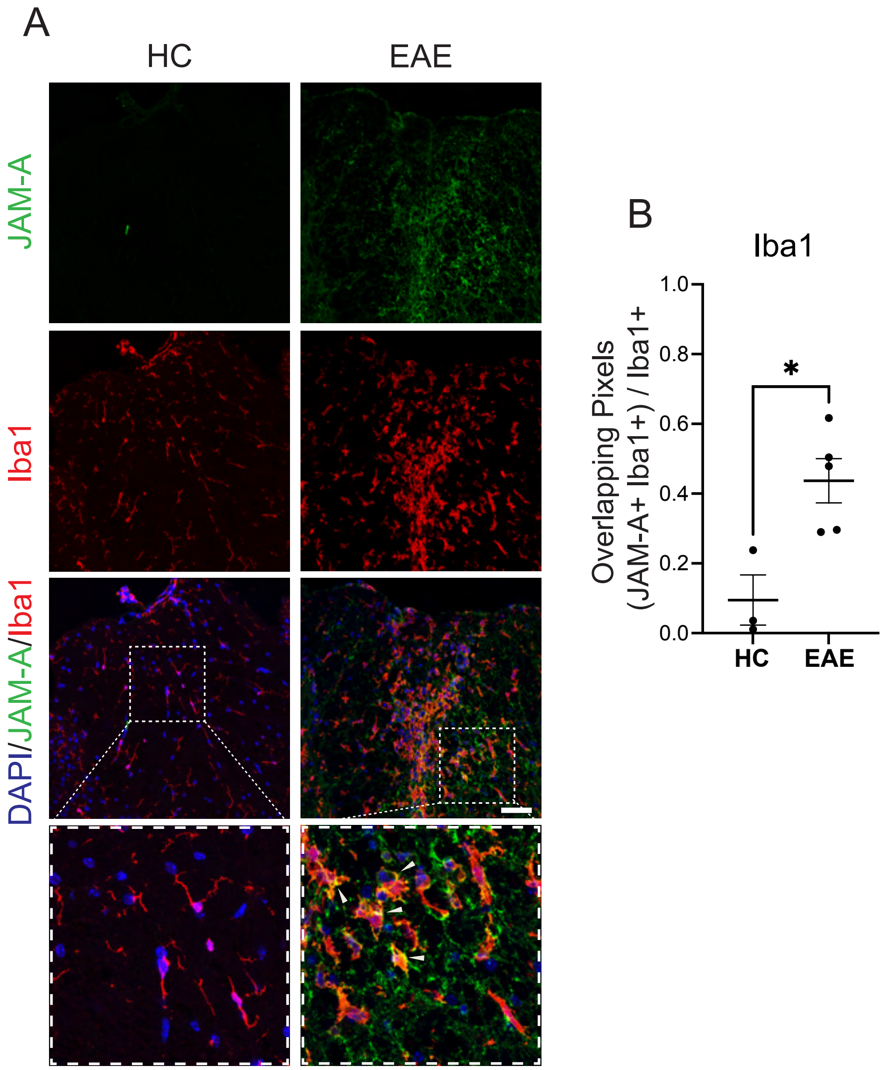

Supplemental Figure 3: JAM-A is expressed by Iba1+ microglia in the spinal cord of mice with EAE. (A) Images show immunohistochemistry for JAM-A (green), Iba1 (red, marker of microglia), and DAPI (blue) in the spinal cord dorsal column of mice with EAE and healthy controls (HC). JAM-A expression is absent in the spinal cord of HC mice, while it is upregulated in mice with EAE. Iba1 positive microglia express JAM-A in areas of inflammation in the spinal cord of mice with EAE (white arrowheads). Scale bar = 50 µm. (B) Colocalization analysis of Iba1 with JAM-A shows that the proportion of Iba1+ pixels overlapping with JAM-A+ pixels increases in EAE mice compared to HC mice ($p=0.0138$, unpaired two-tailed t-test). HC, $n=3$; EAE, $n=5$.

Supplemental Figure 4

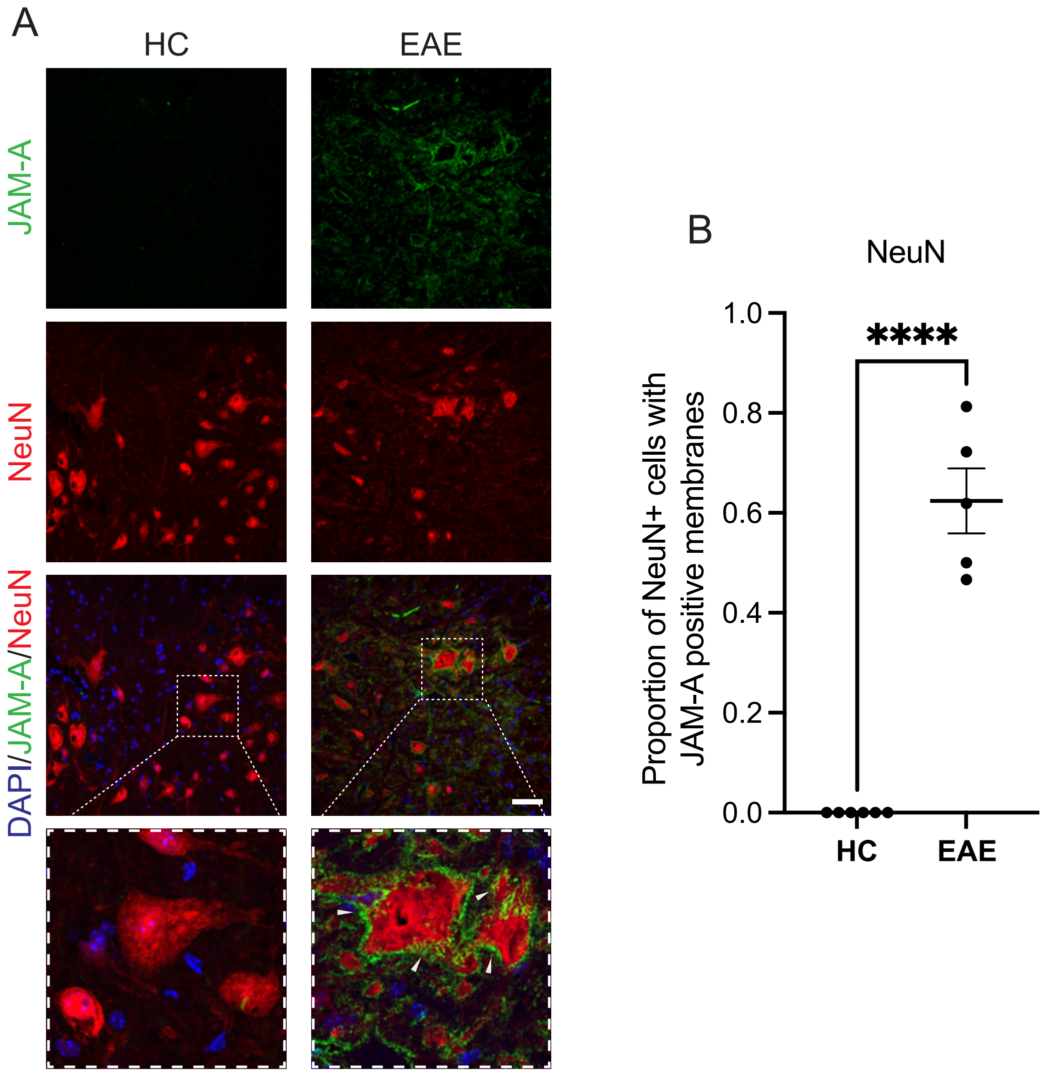

Supplemental Figure 4: Spinal neurons express JAM-A during EAE. (A) Images show immunohistochemistry for JAM-A (green), NeuN (red, marker of neurons' cell body), and DAPI (blue) in the spinal cord ventral horn of healthy control (HC) and WT mice at 5 days from EAE disease onset (EAE). JAM-A expression is minimally detectable in the spinal cord of HC mice, while it is upregulated in EAE and expressed on the cell surface of neurons of the spinal cord ventral horn (white arrowheads). Scale bar = 50 µm. (B) Quantification of the number of JAM-A+ neurons relative to the total number of neurons (NeuN+) in HC and EAE mice showed increases in EAE compared to HC ($p<0.0001$, unpaired two-tailed t-test). HC, $n=6$; EAE, $n=5$.

Supplemental Figure 5

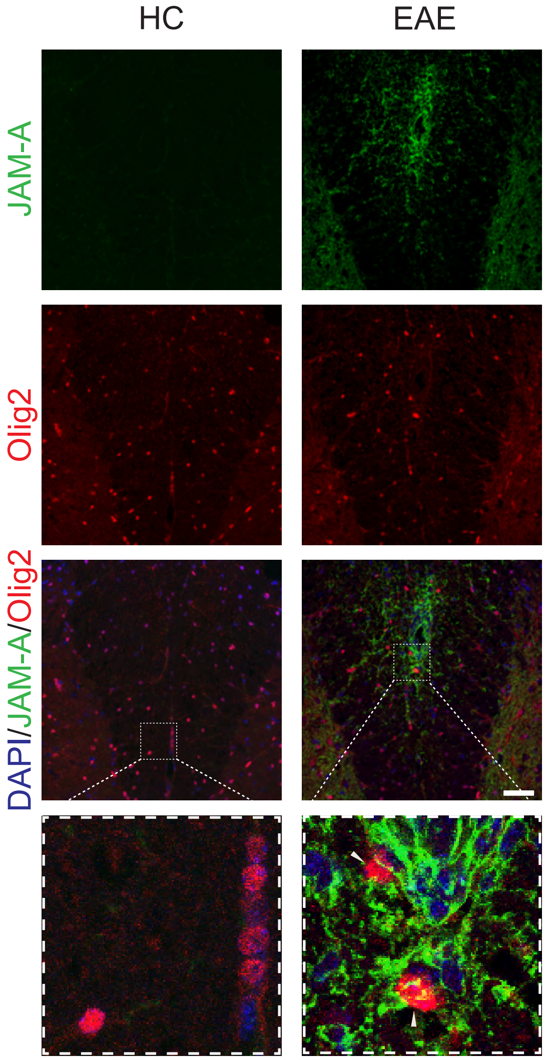

Supplemental Figure 5: JAM-A expression is not clearly detected in oligodendrocytes on immunohistochemistry during EAE. (A) Images show immunohistochemistry for JAM-A (green), Olig2 (red, marker of oligodendrocytes), and DAPI (blue) in the spinal cord dorsal column of healthy control (HC), and WT mice at 5 days from EAE disease onset (EAE). JAM-A expression is absent in the spinal cord of HC mice, while it is upregulated in EAE and expression appears to be separated from the cell surface of the few oligodendrocytes within inflammatory lesions (white arrowheads) in EAE mice. More sensitive techniques, such as cell sorting, may be needed to determine whether oligodendrocytes express JAM-A. Scale bar = 50 µm.

Supplemental Figure 6

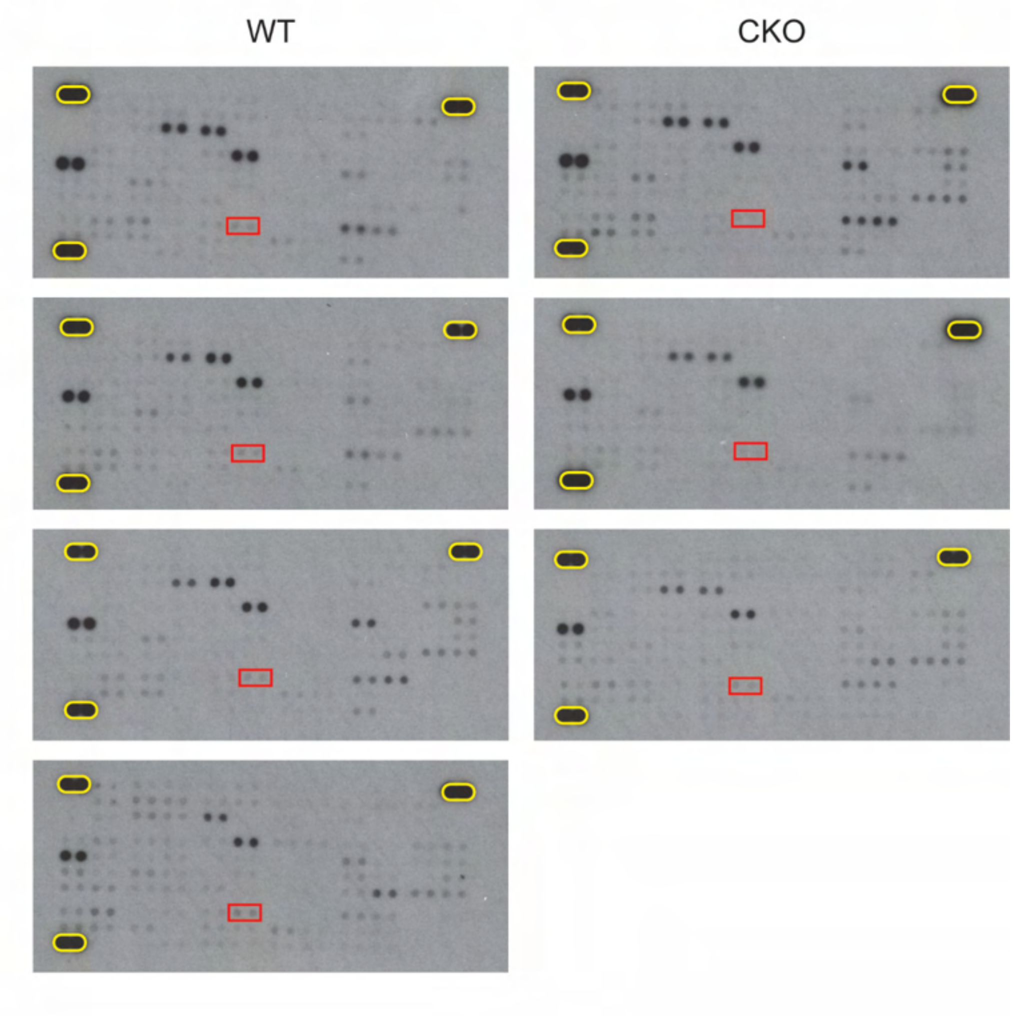

Supplemental Figure 6: Differential expression of MMP-2 is detected on proteome arrays of spinal cord lysates from JAM-A CKO and WT mice during EAE. Images show individual ELISA immunoarrays (WT, n=4; CKO, n=3) assessing the expression of 111 cytokines, chemokines, proteases and acute phase reactants in the spinal cord of mice at 5 days from EAE disease onset. Red rectangles highlight MMP-2 expression which was significantly decreased in CKO mice compared with WT controls, while yellow ovals demarcate the reference spots in each array. A minimal linear contrast enhancement step was performed uniformly across all original blot images prior to analysis. Original blot images are available in the Supplemental Materials.

6/26/17

1 min

Protease Array - astrocytes and lymphocytes
sample 1

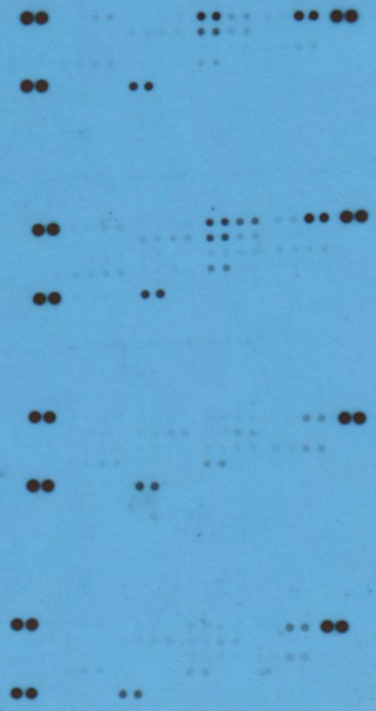

Protease array - astrocyte and lymphocyte sample 2

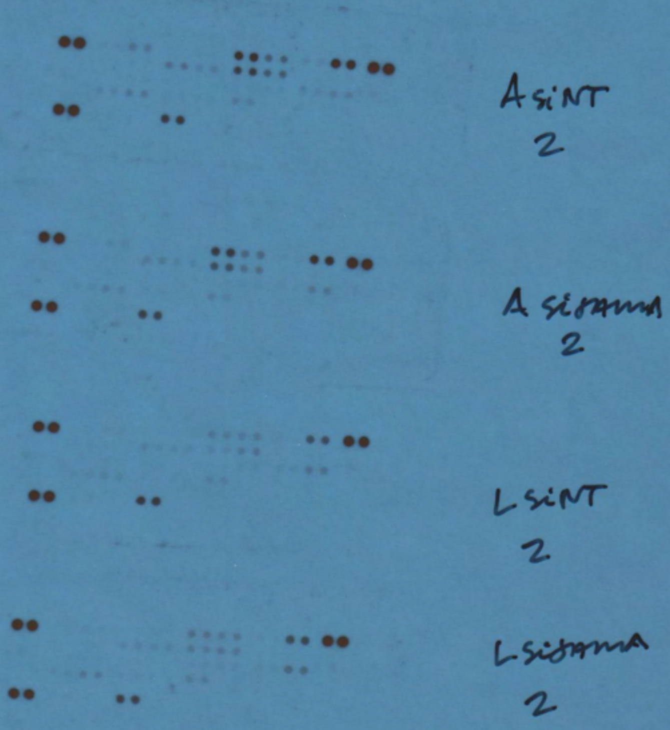

7/27/17 Lysates 30 s

Protease

10 s

Protease array - astrocyte and lymphocyte
sample 3

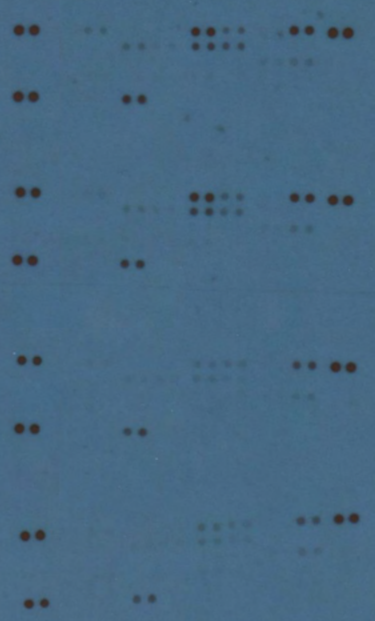

Protease array - supernatant sample 1 (biological sample 2")

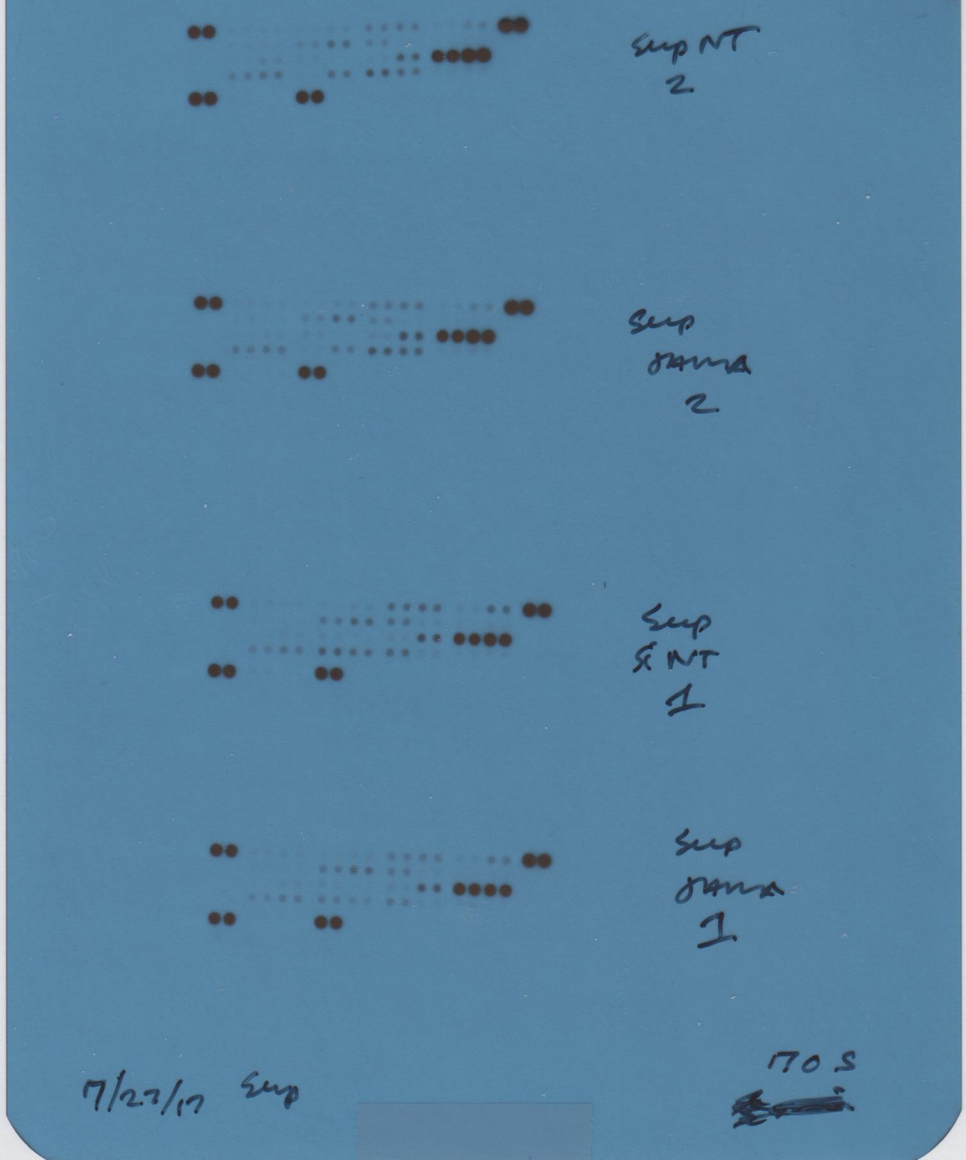

Protease array - supernatant samples 2 and 3 (biological samples "3" and "4")

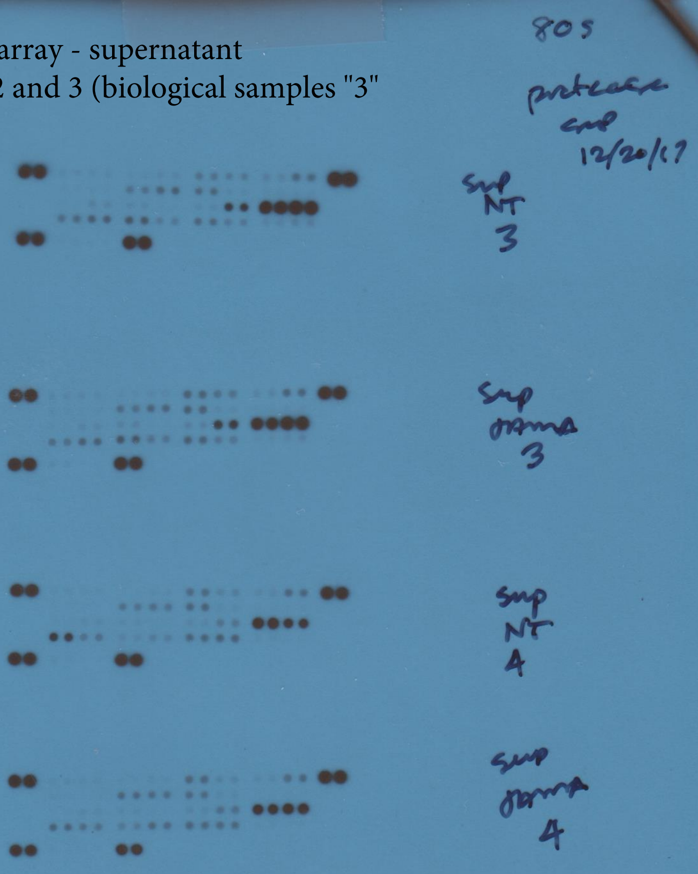

1min supernatant

Cytokine array - supernatant
sample 1 and 2 (biological sample "2" and "3")

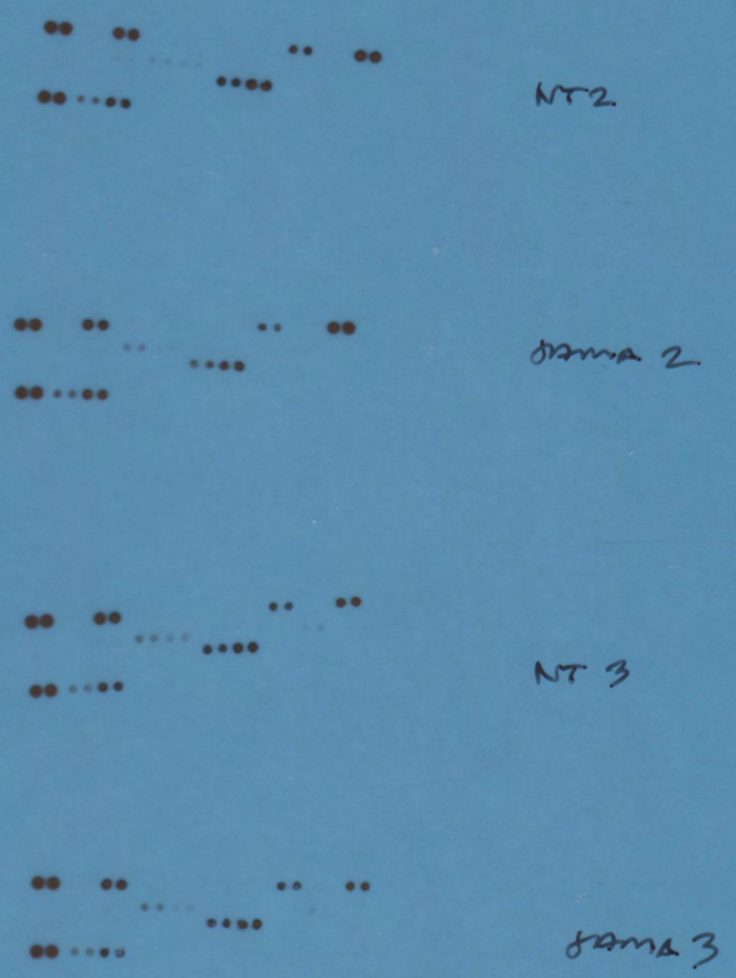

Cytokine array - supernatant sample 3 (biological sample "4")

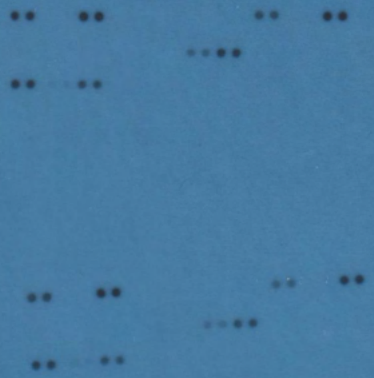

cytokine
15 min

Cytokine array - astrocyte and lymphocyte sample 1

8/11

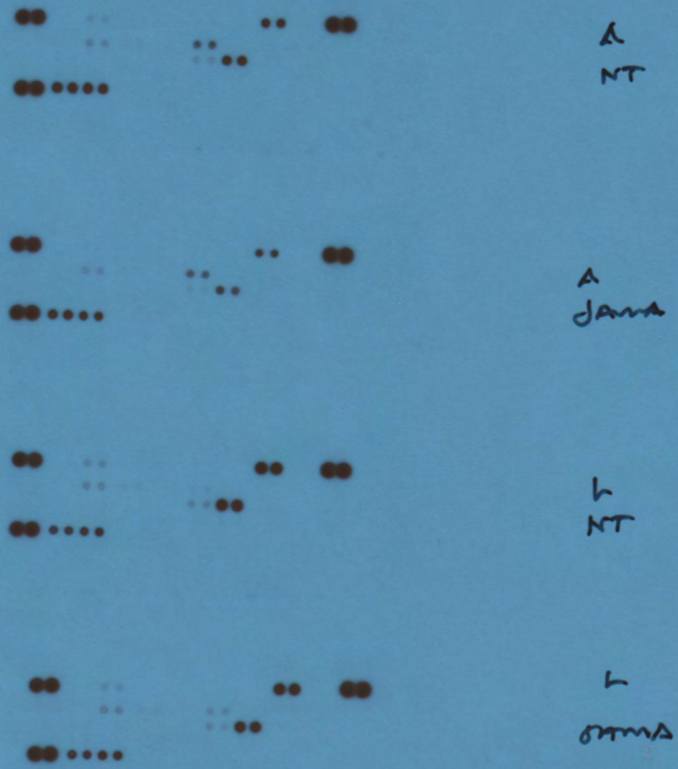

Cytokine array - astrocyte and lymphocyte sample 2

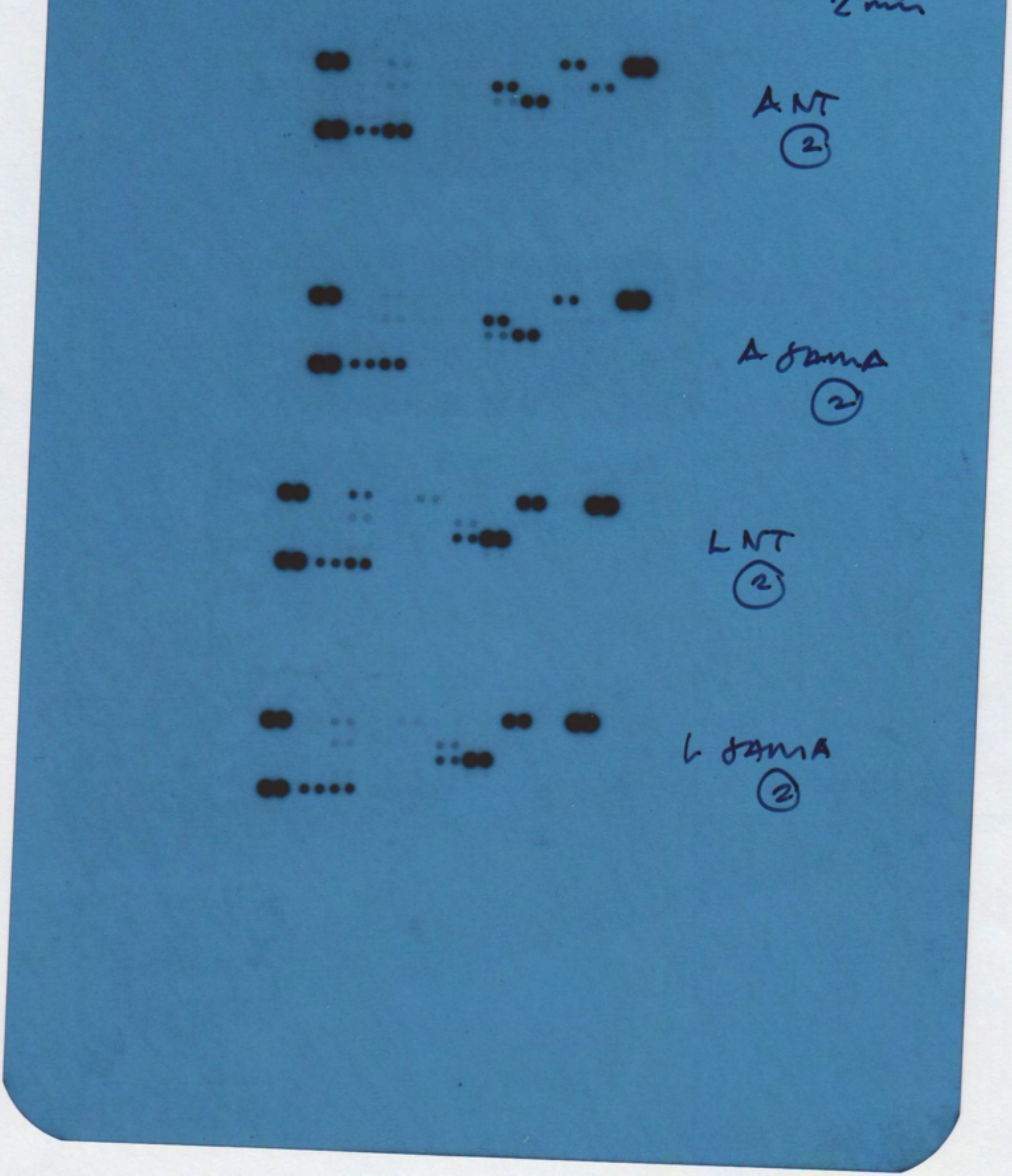

Cytokine array - astrocyte and lymphocyte sample 3 (biological sample "4")

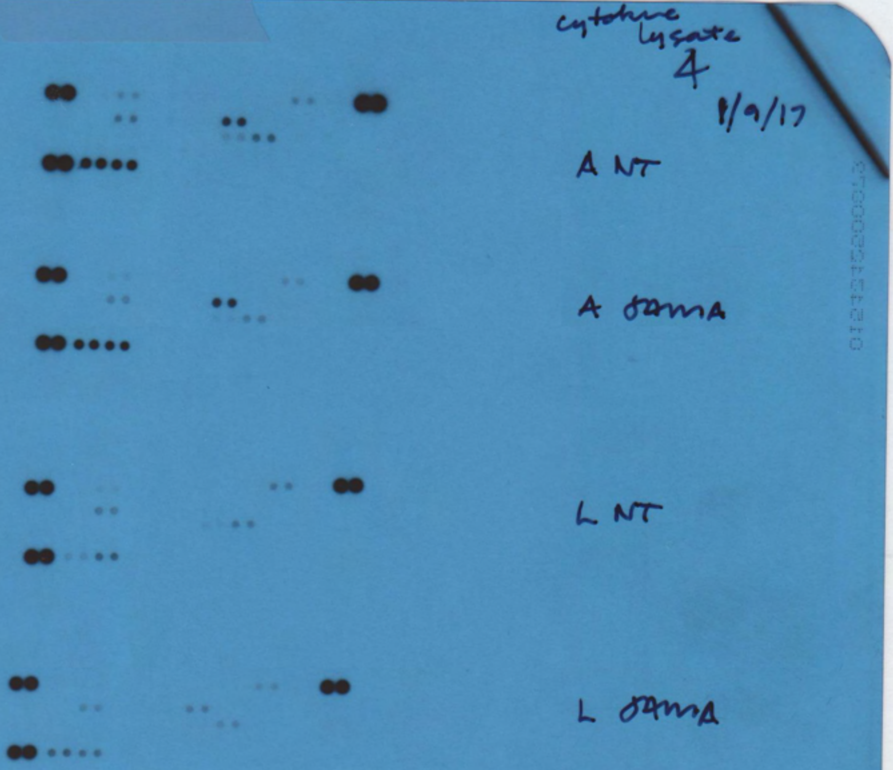

Proteome ELISA arrays - spinal cord lysates harvested 5 days from EAE disease onset

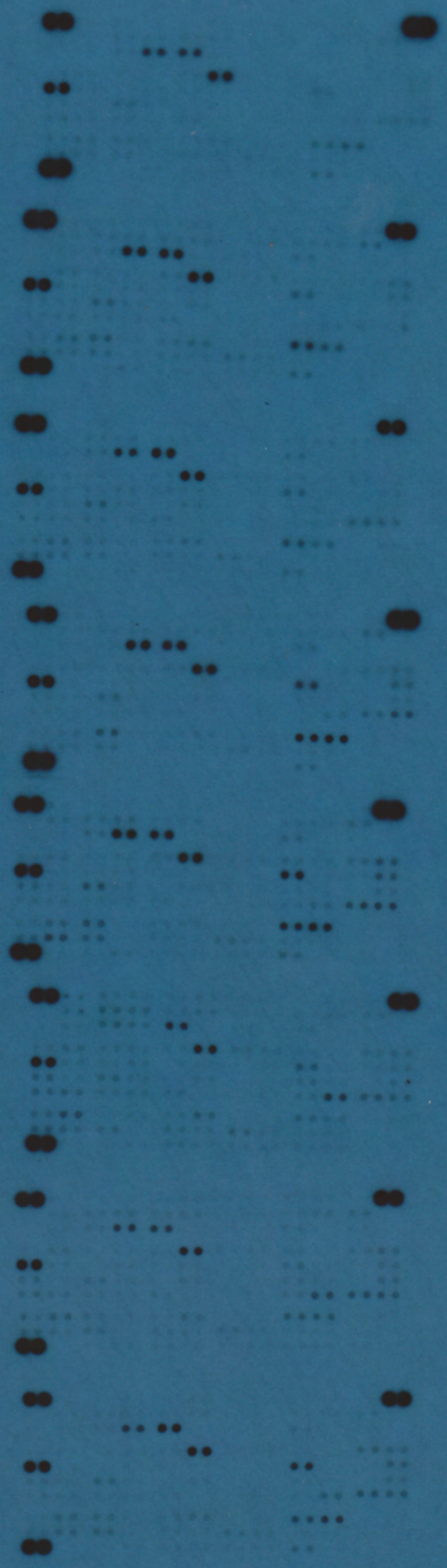

CKO 1

WT 1

WT 2

WT (excluded from analysis as tissue harvested at 7 days from EAE disease onset)

CKO 2

WT 3

CKO 3

WT 4
