# Supplementary material for: Astrocytic junctional adhesion molecule-A regulates T-cell entry past the glia limitans to promote central nervous system autoimmune attack
Source: Brain Commun. 2022 Feb 18;4(2):fcac044. doi: 10.1093/braincomms/fcac044 (PMC8899531; doi:10.1093/braincomms/fcac044)
Supplement: fcac044_Supplementary_Data [file Original Submission-Author's Response.pdf › Original Submission-Author's Response.pdf]

We thank both reviewers for their interest in our work, their thorough reviews of the manuscript and their constructive comments on the study.

We attempted to address all of the comments with additional experiments where feasible along with extensive edits to the text and figures as outlined below. We feel that these major changes to the manuscript have significantly improved the scientific rigor of the study and support our conclusion that astrocytic JAM-A is a contact mediated regulator of T cell entry via MMP-2 with downstream effects on histopathological damage and clinical disability during autoimmune attack.

Reviewer 1

Comments to the Author
In this manuscript Chapouly and colleagues assess the contribution of astrocyte JAM-A to T-cell entry into the brain parenchyma in inflammatory diseases. The authors follow up on previous research that showed an increase of JAM-A in astrocytes in models of central nervous system (CNS) inflammation. In this study, they perform in vitro (astrocyte and T-cell cultures) and in vivo (adenoassociated virus driven IL-1β expression and experimental autoimmune encephalomyelitis (EAE)), to determine the effects of knocking out JAM-A specifically in astrocytes. They find changes in the number of perivascular T-cells, compared to the controls. Interestingly, EAE JAM-A KOs and EAE mice treated with a JAM-A blocking peptide, displayed significant phenotypical recovery when compared to EAE controls.
This manuscript aims to address an interesting hypothesis that is that astrocyte JAM-A contributes to the entry of T-cells from the perivascular space into the CNS parenchyma through the glia limitans. Although I think the topic is timely and important, I consider some of the conclusions raised are not supported by the data in this manuscript. Major corrections will be needed for the paper to be published.

Major points:
Figure 1, Supp Figure 1, and Page 5: The authors determine that JAM-A is upregulated in astrocytes in response to IL-1β and that this does not occur in JAM-A cKO astrocytes. However, these statements are a mere description, no quantification or statistical tests are provided. The authors should include proper quantification with statistics.

We quantified astrocytic JAM-A and occludin expression and performed statistical analysis on both expression levels and co-localization measurements under the different *in vitro* treatment conditions. These results are now included in the text, Figure 1 and Supplemental Figure 1.

Figure 1 and Page 6: The authors state that JAM-A is expressed in infiltrating leukocytes. The authors should perform immunohistochemistry in order to be able to draw that conclusion.

We performed additional immunohistochemistry stains for CD45 and JAMA-A in EAE tissues to demonstrate that JAM-A is expressed in infiltrating leukocytes. These data are included in Supplemental Figure 3.

Page 6, line 43-45: The authors say "Therefore, astrocytic JAM-A is expressed diffusely as a monomeric protein from the time of its upregulation by IL-1β." There are no experiments in this manuscript that

demonstrate that. This sentence should be removed, it is an overinterpretation.

This sentence has been removed from the text and replaced with a conclusion more appropriately supported by the data: "Therefore, astrocytic JAM-A is found both in overlap with the tight junction and apart from it after induction by IL-1β and its distribution apart from the tight junction does not appear to change with the addition of CCL-2."

Supp Figure 2 and Page 6: Iba1 immunostaining does not resemble the expected Iba1 positive microglia pattern. I checked the antibody used and it is not stated in the methods. Are the authors sure the staining is working?

Additional Iba1 – JAM-A co-stains were performed with an alternate Iba antibody (Wako, 019-19741, 1:500) and the expected Iba1 positive microglial pattern was confirmed. These data are now included in the new Supplemental Figure 4. The new Iba1 antibody, along with all additional antibodies used for revision experiments, are cited in the updated Materials and Methods under the Antibodies section.

In addition, quantification and statistics of the colocalization will have to be provided in order to conclude the lack of expression of JAM-A in these cells.

These data were quantified and subjected to statistical analysis and are now included in the new Supplemental Figure 4. Quantified data show a moderate co-localization of JAM-A with Iba1 suggesting that microglia do indeed express JAM-A in the setting of EAE. JAM-A expression has been previously documented in tumor associated microglia of malignant glioma (Pong et al, PLoS One 2013; 8(10):e77571; Turaga et al, Neuro Oncol. 2020 22(11):1591-1601) indicating that microglial upregulation of JAM-A occurs in various disease associated contexts. The role of microglial JAM-A in EAE and other neuroinflammatory contexts remains to be tested.

Finally, is JAM-A expressed in neurons? The authors should include this analysis.

We performed additional immunohistochemistry stains for NeuN and JAM-A in control and EAE tissues and found that JAM-A is indeed expressed on the surface of NeuN expressing cells. These data are quantified and presented in Supplemental Figure 5. The role of neuronal JAM-A in EAE and other neuroinflammatory contexts remains to be tested.

Page 7, lines 20-22: The authors say "astrocytic JAM-A expression in co-culture with CD3+ T cells leads to increased levels of MMP-2, CCL-2 and GM-CSF, ..." The authors show that siJAM-A reduces the expression of MMP-2, CCL-2, and GM-CSF. There are no experiments in this manuscript that demonstrate the statement the authors make. In order to draw such a conclusion, the authors need to have a baseline control and observe an increase of these molecules in relation to baseline levels. Baseline controls could include astrocytes not treated with IL-1β or astrocytes cocultured with non-activated T-cells. This is relevant to Page10, lines 47-49.

We believe that the comparison between activated (IL-1β treated) astrocytes treated with siNT vs. siJAM-A in co-culture with activated T-cells uses the appropriate control and demonstrates that

under pro-inflammatory conditioning of both cell types**, the presence of astrocytic JAM-A leads to higher levels of MMP-2, CCL-2 and GM-CSF than are seen in its absence. We have qualified the statement of concern in the text with the phrase: "in astrocyte-T cell co-cultures subjected to pro-inflammatory conditioning of both cell types." We maintain that siNT is the appropriate control for this experiment, as it necessarily holds all conditions other than astrocytic JAM-A expression of the co-culture constant. Using suggested comparison groups of either astrocytes not treated with IL-1β, or astrocytes co-cultured with non-activated T-cells, introduces additional factors, such as parallel pathways downstream of IL-1β signaling, that potentially contribute to MMP-2, CCL-2 and GM-CSF levels independent of astrocytic JAM-A.**

Page 7, line 54: The authors say "astrocytic JAM-A facilitated CD4+ immune cell infiltration past the glia limitans and into the CNS parenchyma in cortical AdIL-1induced lesions." The authors show in Figure 3 that JAM-A KO have more CD4+ cells colocalizing with the vessels. They do not quantify the number of CD4+ cells in the parenchyma, hence they can not draw the conclusion stated. The authors will need to quantify the number of CD4+ cell in the parenchyma of AdIL-1 injected animals. This is relevant to Page 11, line 20. It is only shown in one model, not two.

Cell localization in cortical AdIL-1 induced lesions is now classified as either within the neurovascular unit (which includes the vascular and perivascular spaces and is demarcated with pan-laminin, a marker of the vascular basement membrane) vs. parenchymal. Cells that did not co-localize with pan-laminin and were located within the parenchymal tissues as demarcated by DAPI staining were interpreted as residing in the CNS parenchyma. We have now added Figure 3G to explicitly report the proportion of CD4+ cells in the parenchyma over the total number of cells.

Moreover, in Figure 3, how do the authors know that the CD4+ cells are in the perivascular spaces and not inside the vessels?

We attempted co-staining combinations to label aquaporin4, CD31 and CD4 or CD45 in AdIL1 and EAE tissues in order to localize the infiltrating immune cells to the perivascular spaces. Unfortunately, we were unable to obtain a clear signal to noise ratio using multiple host-appropriate CD4 and CD45 antibodies in combination with our available working CD31 and aquaporin4 antibodies.

In the AdIL1 model, immune cells do not accumulate in high numbers as they do in the EAE model, so we could not conclude from DAPI staining alone whether the infiltrating immune cells were in the perivascular spaces and not within the vessel lumen. Therefore, we have qualified our interpretation of Figure 3 as localizing these cells to "the neurovascular unit", which is identified by the laminin-positive areas demarcating the basement membranes of the unit.

In EAE, a more detailed immunostaining of the immune cell infiltration pattern is provided in the updated Figure 5. Immune cell infiltration relative to the aquaporin-4 positive endfeet and CD31 positive blood vessels was easily identified by DAPI (costains for CD4 and CD45 were not possible as outlined above, though these cells were identified as CD45+ in separate stains shown in Supplemental Figure 3). In the JAM-A CKO mice, the accumulation of immune cells was localizable to the perivascular spaces.

Figure 4: at least for the analyses on panels M-R, a non EAE control should be included to be able to compare to baseline levels.

Immunohistochemistry stains for FM, NeuN and GFAP have now been added for non-EAE controls as well as EAE at an earlier time point (5 days from EAE disease onset) and have now been included along with the Day 28 data in a new analysis in Figure 6.

Page 10, lines 13-18: The authors say "we demonstrate a novel astrocyte-T cell interaction that modulates lymphocyte trafficking into the CNS parenchyma with downstream effects on histopathological damage and clinical disability." Astrocyte-T cell interactions are not shown in vivo in this manuscript. This statement should be rephrased, or experiments performed to show that is the case.

This statement has been modified to: "we demonstrate a novel *role for the astrocyte cell signaling receptor, JAM-A, in modulating lymphocyte trafficking* into the CNS parenchyma with downstream effects on histopathological damage and clinical disability." We have also noted that "Additional *in vivo* imaging experiments to characterize the dynamics of immune cell trafficking in the presence and absence of astrocytic JAM-A are also in progress."

Minor points
Figure 1 and Page 6: Aquaporin 4 staining would reinforce the point that JAM-A expression is increased in astrocyte endfeet.

Aquaporin 4 staining along with CD31 and JAM-A in control and EAE tissues have now been performed and are now included in Supplemental Figure 2, demonstrating upregulation of JAM-A in both the astrocytic endfeet and endothelial cells during EAE.

Figure 1 and Page 6: It would be interesting to know what cells are infected with AdIL-1 and express IL-1β.

The AdIL-1 virus was constructed by Kolb et al, J Clin Invest. 2001; 107(12): 1529–1536, and consists of a human IL-1β cDNA driven by the human CMV promoter which is predicted to induce IL-1β expression in all mammalian cell types of the mouse central nervous system, although this has not been systematically characterized with in situ hybridizations post-AdIL-1 injection. The inflammatory phenotype of IL-1β injections into the rodent CNS, has been well characterized previously in rats in Ferrari et al, Am J Path. 2004; 165(5): 1827-1837 and Godoy et al, J Neuroimmunol. 2010; 222(1-2): 29-39 and in mice in Argaw et al, J Clin Invest 2012. 122(7): 2454-2468 and Horng et al, J Clin Invest. 2017; 127(8): 3136-51. The latter two studies demonstrated that AdIL-1 induces blood brain barrier breakdown, leukocyte recruitment and microglial and astrocytic activation within 7 days after intracortical injection.

Page 6, line 52: Since the efficacy of siJAM-A is not shown in this manuscript it would be useful for the readers to know that it is shown in Horng et al 2017. It would be good to reference it here.

This citation has been added to the text: "Efficacy of siRNA mediated knock-down of JAMA (*siJAM-A*) in comparison to a non-targeting siRNA (*siNT*) was demonstrated previously (Horng et al, 2017)."

Page 7, line 43: The authors should specify what CKO and WT is referring to.

Genotype references for CKO and WT mice have been added to the text.

Figure 3: Could the authors indicate if the n numbers refer to number of mice or something else?

"n" refers to numbers of mice in each group and has been clarified in the figure legend text.

Figure 4N: The authors should include the units.

Proportion of the FM positive area of the lumbar anterolateral white matter tracts was reported and has been clarified in Figure 6 and legend text.

Page 9, line 20: The authors should refer to changes in immunohistochemical staining rather than decreased demyelination, neuronal cell death and astrocytic activation. This could be an overinterpretation, e.g. NeuN expression could be reduced without neuronal cell death.

Reporting of immunohistochemical staining has now been modified to describe changes in staining patterns rather than indirect inferences on demyelination, neuronal cell death and astrocytic activation.

Figure 4: The authors should indicate the timepoint at which the mortality differences were assessed.

Differences in mortality rates were assessed at Day 28 and this has been added to the text and in the legend of Figure 4. We have also now included in the updated Figure 4 a survival analysis both for mortality (which was statistically significant between groups) and induction of disease (which was not).

Page 11, line 27: The manuscript would benefit of quantification of potential changes of expression of JAM-A in endothelial cells in the CKO.

JAM-A and CD31 colocalization was performed in immunohistochemical stains of aquaporin-4, JAM-A and CD31 from control and EAE tissues in WT and JAM-A CKO mice and quantified. These data are now included in Supplemental Figure 2.

Reviewer: 2

Comments to the Author
This is an interesting study focusing on an important aspect of neuroinflammatory disease pathology, namely the step-wise process of T cell infiltration into the CNS parenchyma, and in particular the last step of this process, the crossing of the glia limitans. However, the study seems to make leaps between almost preliminary in vitro associations and true mechanistic links between a proposed hypothesis and an established and well supported body of evidence.

Some key concerns are listed below:
Some paragraph titles are overstating biological significance indicative of in vivo findings, based solely on marginal differences detected with a minimal number of in vitro experiments. Similarly, summarizing sentences also over-interpret results shown with often marginal differences or trends, and certainly not well substantiated by the data presented.
Overall, other than the limited in scope and importance in vitro co-culture experiments showing changes in some inflammatory mediators, no real link is established showing how / when astrocytic JAM-A promotes T cell infiltration on the cellular or molecular mechanism level.

We have now included data which establishes a mechanistic link between astrocytic JAM-A signaling and T cell infiltration *in vivo* via the protease MMP-2, which recapitulates our *in vitro* findings in the EAE disease model. Using a mouse XL proteome profiler probing 111 soluble mouse proteins, including cytokines, chemokines, proteases, growth factors and acute phase signals, we compared the proteomic patterns of spinal cord tissues from WT and CKO mice at 5 days from EAE disease onset. New immunohistochemical stains (included in the updated Figure 4) demonstrate that at this timepoint, immune cells are accessing the perivascular spaces and interacting with the astrocytic endfeet in both WT and CKO mice. Results from this experiment reveal that of 111 probes, MMP-2 demonstrates the highest fold change and represents the sole factor with a statistically significant difference in expression between WT and CKO mice. Previous work established a role for MMP-2 in promoting T cell entry into the CNS parenchyma from the perivascular spaces via several mechanisms: 1) digesting dystroglycans that anchor the astrocytic endfeet to the parenchymal basement membrane (Agrawal et al, 2006; J. Exp. Med. 203, 1007-1019), 2) activating the proinflammatory NFκB pathway in astrocytes via Notch-1 (Song et al, Cell Reports. 2015; 10, 1040-1054) and 3) degrading perivascular reserves of CXCL12, which promotes the retention of T cells within the perivascular space (McCandless et al, J Neuroimmunol. 2006; 177, 8053-8064). Though no statistically significant differences were found for GM-CSF and CCL2, the two other factors identified in our *in vitro* screen, local changes within the perivascular spaces as mediated by astrocytic JAM-A cannot be excluded and may require more sensitive techniques for detection *in vivo*.

The reduced clinical severity conferred by either the genetic mutation or the pharmacological treatment is significant, but does not seem to justify statements such as "protects against clinical disability" when mice seem to get peak scores between 2.5-3.

To more accurately describe the effects of the genetic mutation and pharmacological treatment, we have changed "protects against clinical disability" to "reduces clinical disability" in the text.

Per unit area measurements need to be performed by using actual tissue area measurement tools and not be deduced as per "20x or 40x field" as individual pictures can include empty spaces (devoid of tissue material) of variable sizes.

Number of CD4+ cells have now been reported as number of cells per μm2 in Figure 3D. NeuN positive cells have now been reported as number of cells per μm2 in Figure 6D. GFAP pixel sum has now been expressed as pixel sum per μm2 in Figure 6E.

How tissues were sampled across the relevant affected lesions or perilesional areas needs to be better described. If experiments and analyses were performed in a blinded or unblended manner and how data were compiled and checked for reproducibility need to be better described.

Details of how tissues were sampled across affected areas of the cortical injections and EAE spinal cord tissues are now more thoroughly described in the Materials and Methods section under Morphometric analysis.

Details on the blinding of experiments and analyses have now been added to the Materials and Methods under EAE and Morphometric Analyses sections.

The type of tissue shown and anatomical location should be specified in the legends for all histology shown in all figures.

Types of tissue and anatomical location are now detailed in the legends for all histology shown in the figures.

Figure 1: Images are not very conclusive as it is hard to appreciate if there is tight junction co-localization with the CCL2 addition or not (seems absent in fig1A) but the lack of precise colocalization analysis done per se makes any conclusion seem random. Proper statistical analyses need to be performed and described.

As noted in response to the similar request above from Reviewer 1, we quantified astrocytic JAM-A and occludin expression and performed statistical analysis on both expression levels and co-localization measurements under the different *in vitro* treatment conditions. Representative images for quantification were analyzed using 3-4 images derived from at least two technical replicates and experiments were conducted using 2-3 biological replicates. These results are now included in the text, Figure 1 and Supplemental Figure 1.

Fig. 1 and Suppl. Figs 1 and 2: No quantification, no statistical analyses. What stage of EAE and clinical score were these analyses performed at?

Quantification and statistical analyses are now included in Figures 1 and Supplemental Figure 1 and in the colocalization analyses of JAM-A with aquaporin-4, CD31, GFAP, Iba1 and NeuN included in Figure 1 and the new Supplemental Figures 2-6. EAE tissues were from tissues collected at 5 days from disease onset and scores for both WT and CKO mice used in the analysis ranged from 2.5 to 3.5 with no significant difference in average clinical scores. This information has now been included in the above Figure legends.

Fig 1 and S2: why is there so little JAMA in the EAE images? If it is such an essential mediator of CNS infiltration, shouldn't it be detected in abundance at least within CNS lesions?

We performed additional JAM-A staining in EAE tissues and provided additional higher power images in Figures 1 and Supplemental Figures 2 through 5, which make it easier to appreciate the increased expression of JAM-A, particularly in GFAP positive cells (which was quantified) in EAE.

Fig. 2: Three biological replicates likely from the same single experiment seems limited, especially given the size of the error bars for several markers. What do error bars represent? The statement that statistical comparisons were only performed on those comparisons that had visually detectable differences sounds subjective and raises doubts about the selection process and the statistical methodology employed. The presentation of the figure could be much improved over its current lab meeting layout.

Three different biological replicates of astrocyte and T cell samples were used in three *separate* experiments. These therefore represent three technical replications. The error bars represent standard errors of the mean and reflect the variance across experiments which involved both biological and technical replication.

Comparisons were expanded to include all array probes. Probe signal was standardized to the average of 3 duplicated reference probes, and the relative change between *siNT* and *siJAM-A* treated conditions was calculated and then compared to the relative change in the *siNT* condition (ie. zero). Statistical analyses were performed using two sample t-tests with unequal variance. Comparisons with statistical significance needed also to consist of probe signals that appeared visually detectable above background in order to be included in the Results. Updates to the Materials and Methods, the figures and figure legends have been made to reflect the revised analysis.

The presentation of the figure has been updated as requested by the reviewer and to meet the editorial office's requests below.

Fig 3: Why are there more CD4 T cells in the JAMA cKO mice?

It is unclear why there are more CD4+T cells in the cortical injections of CKO mice compared to WTs. Because we did not see differences in the CD4 T cell number between CKO and WT spinal cords in EAE, we speculate that the traumatic injury of injection could have contributed to the T cell infiltration response in addition to AdIL-1 in the presence and absence of astrocytic JAM-A. The arrest of T cells within the neurovascular unit in both cortical injection and EAE tissue supports a common role in the direct modulation for astrocytic JAM-A in lymphocyte trafficking in both *in vivo* inflammatory models. However, the increased number of CD4+ T cells in the cortical injection model alone suggests that there are additional chemotactic pathways that perhaps fail to receive negative feedback either due to the direct absence of astrocytic JAM-A signaling or to the downstream absence of T cell signaling events within the parenchyma. These questions are an interesting area of future study.

How is the PVS determined based on these pan-laminin staining patterns that show no perivascular cuff? Far better histological, imaging and/or other complementary methods would be required to establish that the claims made regarding exact localization of cells relative to the glia limitans are anatomically accurate.

As noted above in response to a similar concern raised by Reviewer 1, we attempted to better define the location of the immune cells within the neurovascular unit in AdIL1 injected cortex using aquaporin4 and CD31 staining along with an immune cell marker such as CD45 and CD4. Unfortunately, we were unable to find a host appropriate CD4 or CD45 antibody that gave a clear signal to noise signal on immunostaining with aquaporin4 and CD31. In AdIL1 injected tissues, single immune cells infiltrate into the lesion as opposed to the large clusters of infiltrating cells seen in EAE. Therefore, within AdIL1 lesions, it was difficult to establish precise localization of single immune cells within the neurovascular unit using DAPI signals in relation to aquaporin4 and CD31 stains. We have qualified our interpretation of these experiments referring to the immune cells that co-localize to the laminin positive basement membrane of vasculature as coincident with the "neurovascular unit" and not yet accessing the parenchyma.

Using the same immunohistochemical approach in EAE tissues, where there are many more infiltrating cells, we were able to localize immune cells in more detail with respect to the neurovascular unit and these results are included in the updated Figure 5. These new images show DAPI positive immune cells accumulated within the perivascular spaces between the aquaporin4 positive endfeet and CD31 positive endothelial wall in JAM-A CKO mice, whereas these cells are localized in the parenchyma in WT mice.

What measures were taken to ensure sections analyzed where at the epicenter of the needle path (namely that the difference or trend seen between the two groups was not due to different distance from the needle path?) were serial sections analyzed?

Serial sections were captured through the cortical injection site – each brain was sectioned in 25 µm thickness distributed serially across 10 slides from the posterior to anterior end of the brain; adjacent sections on the same slide were roughly 250 µm apart. Sections for analysis were selected to represent the center of the lesion, corresponding to the area of greatest lesion length as noted both by the width of NeuN loss and GFAP positivity. Field of analysis for lymphocyte localization relative to the PVS was selected on a 40x field of approximately 385µm width centered over the midline of the cortical injection site.

Details of this analysis are now outlined in the Materials and Methods section under morphometric analysis.

Fig 4: How were the average EAE score, mortality, and disease free values calculated?

Mice were scored daily on a standard 5-point motor scale from Days 7–28 after induction: 0, no symptoms; 1, floppy tail; 2, hind limb weakness (paraparesis); 3, hind limb paralysis (paraplegia); 4, forelimb and hind limb paralysis; 5, death. The average EAE score consisted of the average score across all animals of the same genotype on that day. Average mortality was calculated as the proportion of animals with a score of 5 by the end of the experiment (Day 28). Disease free values were calculated by the proportion of animals who maintained a score of 0 throughout the entire experiment (Day 7-28). These additional descriptions are now included in updated Materials and Methods.

Why are there almost no cuffs in WT EAE mice at peak disease?

Prior work characterizing the histopathology of EAE in WT C57BL/6J mice at disease onset and peak, demonstrates that at the peak of EAE, immune cells have typically crossed the glia limitans and are located diffusely within the CNS parenchyma (Brown and Sawchencko. 2007. J. Comp Neurol., 507; 236-260; Caravagna et al 2018 Sci. Rep. 8(1):5146). Perivascular localization and cuffs are typically observed prior to or at the time of clinical onset (ie. in the earlier stages of clinical disease). It has been inferred from experiments in which the blockade of signals critical for immune cell entry from the blood stream (VLA-4, Yednock et al, 1992 Nature 356, 63–66) or from the perivascular spaces (MMP-2/MMP-9, Song et al, 2015, Cell Reports 10, 1040–1054) protects against clinical disease, that parenchymal access is a pathological step that coincides with the peak of clinical disability in EAE.

Day 5 from clinical onset likely corresponds to peak EAE? That is a time point at which one expects low numbers of encephalitogenic T cells in the spleen as they are mostly already in or on their way into the CNS.

In EAE of WT mice, peak disease is typically observed 4-6 days from clinical onset. Previous time course experiments have found that at the time of peak disease, CD4+ T cells increase modestly within the spleen and exponentially spike within the spinal cord (Barthelmes, et al. 2016, J Vis Exp. 111: 53933). We selected this most informative time point to test whether astrocytic JAM-A affected total CD3+ and CD3+CD4+ T cell numbers present in the spleen or spinal cord.

J: 81.69% for both conditions shows no trend.

We thank the reviewer for noting this typographical error, the intended reporting of data has been updated into the text as 84.73% vs. 87.97%.

Results speak of CD3 and CD4 cell comparisons by flow, but figure legend speaks of CD3, CD4 and CD8 comparisons but they are not shown in the figure.

Again, we thank the reviewer for identifying this typographical error which was carried over from a previous draft which included data on CD8+ cell proportions. We have removed this data from the current manuscript.

The figure shows a massive difference in demyelination between WT and cKO spinal cords, but the quantification does not give the impression that the examples shown are indeed representative of the small difference actually quantified.

A more representative set of images demonstrating the average level of difference in demyelination between WT and CKO spinal cord has now been included and the previous images have been removed from updated Figure 6.

The NeuN images shown are clearly acquired at a different magnification therefore the comparisons shown on “representative images” is misleading.

The NeuN images are all acquired at the same magnification. We have replaced all images in the updated Figure 6.

Non parametric statistical tests should perhaps be considered for EAE clinical curves among non –equal groups.

In addition to the two way ANOVA test (which accounts for the 2 variables: time and severity) with Bonferroni correction, we additionally analyzed the EAE data using a non-parametric one way ANOVA test (Friedman) with Dunn's multiple comparisons correction and found similar statistical differences. The results of both of these analyses are now reported in the updated manuscript. Individual data points for Average, Peak and Cumulative Score have also now been graphically and statistically analyzed using the non-parametric Mann Whitney U-test in the graphs of the new Figure 4.

Although commonly and casually used, "ug" is not a proper prefix symbol referring to an international unit of mass and thus should not be used in a scientific publication.

The abbreviation μg has now been used in all notations referring to micrograms in the text.

Editorial Office comments:

-Please supply a graphical abstract. A graphical abstract is a visual representation of the central finding or methodology of the paper. It will be displayed in the table of contents. Authors should strive to make the graphical abstract informative, interesting, visually appealing, and straightforward (no caption needed, no abbreviations except gene names). Please upload as a separate high resolution image file (tiff preferred). The technical requirements for the graphical abstracts are:
--size of the submitted image: 1200 pixels square at 300dpi
--font of the text: Arial 12-16 point (smaller fonts will not be legible online)

A graphical abstract has now been included in the manuscript.

-Please add an Abbreviated Summary (up to 50 words) of your paper that captures the main purpose and conclusions of the work. The summary should state the main results written in the third person ('[1st author surname] et al. report that ...') and conclusion. This summary will be used in the contents list online and should not contain any abbreviations other than those on the accepted abbreviations list and accepted gene/protein names, making it accessible to the non-specialist. Please upload it as a Word document. Allowed abbreviations: AIDS; ANOVA; AMPA; ATP; A,T,C,G; CNS; CSF; CT; DNA; ECG; EEG; EMG; GABA; HIV; MRI; NMDA; PET; PCR; RNA.

An abbreviated summary has now been included in the manuscript.

-Fig.2: Full- size and uncropped blots/gels are to be made available as Supplementary material.

A .pdf file of the full size and uncropped blots are provided as Supplementary material. We have also included a .pdf file of the full size and uncropped blots for the new in vivo ELISA experiments included in the new Figure 5.

- Fig2: please provide the number of data points in each group and if the number is <10 show the single data-points.

Number of data points is n=3 for all groups and the updated Figure 2 has now been updated to show the single data points.

- Fig3b,d,f and Fig4g-l,n,p,r: can you please specify in the corresponding figure legend, how many cells/mouse were analysed?

For Figure 3B, each data point refers to size of NeuN area in one section at the center of the lesion (where the width is widest on serial sections as detailed in the Materials and Methods); number of mice analyzed were n=11 CKO and n=8 WT.

For Figure 3D, in the WT, a range of 0 to 78 cells were analyzed per mouse (average 22.6); in the CKO, a range of 8 to 230 cells were analyzed per mouse (average 47). Number of mice analyzed were n=9 CKO and n=7 WT.

For Figure 3F and G, in the WT, a range of 1 to 10 cells (average 6.5) were analyzed per mouse; in the CKO, a range of 5 to 18 cells (average 10.1) were analyzed per mouse. Number of mice analyzed were n=8 CKO and n=6 WT.

For the old Figures 4G-H (new Figures 5C-D), in the WT, the number of analyzed cuffs per mouse ranged from 0 to 6 (average 1.4); in the CKO, the range was 0 to 10 (average 4.8); number of mice analyzed were n=5 WT and n=7 CKO.

For the old Figures 4I-J (new Figures 5H-K), flow cytometry for spinal cord tissues was set to run CD3+ events up to 2000; total CD3+ counts from actual samples ranged from 298 to 1340.

For the old Figures 4K-L (new Figures 5L-O), flow cytometry for spleen tissues all exceeded CD3+ counts of 40,000.

For the old Figure 4P (new Figure 6C-E), a range of 6-25 cells per field of view in each mouse; number of mice analyzed were n=6 HC, n=5 WT EAE 5 days from onset, n=5 CKO EAE 5 days from onset, WT EAE Day 28 n=6, WT CKO Day 28 n=5.

For the old Figures 4N and 4R (new Figure 6A-B and Figure 6F-G) cell counts were not involved in the analysis. Details of the number of representative samples and numbers of mice are included in the 1 updated figure legends.

All of this additional information has been added to the corresponding figure legends.
