# Supplementary material for: Astrocytic junctional adhesion molecule-A regulates T-cell entry past the glia limitans to promote central nervous system autoimmune attack
Source: Brain Commun. 2022 Feb 18;4(2):fcac044. doi: 10.1093/braincomms/fcac044 (PMC8899531; doi:10.1093/braincomms/fcac044)
Supplement: fcac044_Supplementary_Data [file Original Submission-Decision Letter(1).pdf › Original Submission-Decision Letter(1).pdf]

Original Submission – Decision Letter

29-Dec-2020

Dear Dr. Horng,

Thank you for your recent submission BRAINCOM-2020-359 entitled "Astrocytic JAM-A Regulates T Cell Entry Past the Glia Limitans to Promote CNS Autoimmune Attack" to Brain Communications.

Your manuscript has been reviewed, and while the reviewers and editorial team appreciate many aspects of the paper, they raise some concerns that you will need to address in order for the paper to be considered for publication.

If you can address these concerns, I invite you to submit a revised manuscript and a response to each of the reviewers' comments. The reviewer(s)' comments are included at the bottom of this message.

In addition to the reviewers' comments, please note our editorial comments added below to comply with our requirements for presentation of scientific data at Brain Communications.

Please format your revised manuscript according to our Instructions to Authors https://academic.oup.com/braincomms/pages/General_Instructions – a summary of key points is attached to help with this.

To revise your manuscript, click the link below or log into https://mc.manuscriptcentral.com/braincom and enter your Author Centre, where you will find your manuscript title listed under "Manuscripts with Decisions." Under "Actions," click on "Create a Revision." Your manuscript number has been appended to denote a revision.

*** PLEASE NOTE: This is a two-step process. After clicking on the link, you will be directed to a webpage to confirm. ***

https://mc.manuscriptcentral.com/braincom?URL_MASK=d06e536f3dd644f79d9e215c6da20615

Please upload a revised manuscript with changes tracked with track changes or in coloured text.

When submitting your revised manuscript, you will be asked to respond to the reviewer(s) comments. Please be as specific as possible in your response to all of the comments.

The editorial team at Brain Communications aims to increase transparency in scientific publishing and to demonstrate the value that peer review brings to the scientific process. Please therefore consider whether you are willing to have reviewer(s)' comments on your paper and your response published as supplementary data with the paper if it is accepted. This is entirely optional and will only be done if both the authors and reviewers agree. While submitting your revision there will be an opportunity to review or update the answer you gave previously, if necessary.

IMPORTANT: Your original files are available to you when you upload your revised manuscript. Please delete any redundant files before completing the submission.

Because we are trying to facilitate timely publication of manuscripts submitted to Brain Communications, we would appreciate your revised manuscript as soon as possible. If it is not possible for you to resubmit within 3 months, the option to submit a revision will expire, and we would then consider your revised paper as a new submission. However, if you need a little more time, please get in touch and we can extend this deadline for you.

Once again, thank you for submitting your manuscript to Brain Communications and I look forward to receiving your revision.

Yours sincerely,

Dr. Ragnhildur Káradóttir
Editor, Brain Communications

Reviewer(s)' Comments to Author:

Reviewer: 1 (Blanca Diaz-Castro)

Comments to the Author

In this manuscript Chapouly and colleagues assess the contribution of astrocyte JAM-A to T-cell entry into the brain parenchyma in inflammatory diseases. The authors follow up on previous research that showed an increase of JAM-A in astrocytes in models of central nervous system (CNS) inflammation. In this study, they perform in vitro (astrocyte and T-cell cultures) and in vivo (adenoassociated virus driven IL-1β expression and experimental autoimmune encephalomyelitis (EAE)), to determine the effects of knocking out JAM-A specifically in astrocytes. They find changes in the number of perivascular T-cells, compared to the controls. Interestingly, EAE JAM-A KOs and EAE mice treated with a JAM-A blocking peptide, displayed significant phenotypical recovery when compared to EAE controls.
This manuscript aims to address an interesting hypothesis that is that astrocyte JAM-A contributes to the entry of T-cells from the perivascular space into the CNS parenchyma through the glia limitans. Although I think the topic is timely and important, I consider some of the conclusions raised are not supported by the data in this manuscript. Major corrections will be needed for the paper to be published.

Major points:
Figure 1, Supp Figure 1, and Page 5: The authors determine that JAM-A is upregulated in astrocytes in response to IL-1β and that this does not occur in JAM-A cKO astrocytes. However, these statements are a mere description, no quantification or statistical tests are provided. The authors should include proper quantification with statistics.
Figure 1 and Page 6: The authors state that JAM-A is expressed in infiltrating leukocytes. The authors should perform immunohistochemistry in order to be able to draw that conclusion.
Page 6, line 43-45: The authors say "Therefore, astrocytic JAM-A is expressed diffusely as a monomeric protein from the time of its upregulation by IL-1β." There are no experiments in this manuscript that demonstrate that. This sentence should be removed, it is an overinterpretation.

Supp Figure 2 and Page 6: Iba1 immunostaining does not resemble the expected Iba1 positive microglia pattern. I checked the antibody used and it is not stated in the methods. Are the authors sure the staining is working?
In addition, quantification and statistics of the colocalization will have to be provided in order to conclude the lack of expression of JAM-A in these cells.
Finally, is JAM-A expressed in neurons? The authors should include this analysis.
Page 7, lines 20-22: The authors say "astrocytic JAM-A expression in co-culture with CD3+ T cells leads to increased levels of MMP-2, CCL-2 and GM-CSF, ..." The authors show that siJAM-A reduces the expression of MMP-2, CCL-2, and GM-CSF. There are no experiments in this manuscript that demonstrate the statement the authors make. In order to draw such a conclusion, the authors need to have a baseline control and observe an increase of these molecules in relation to baseline levels. Baseline controls could include astrocytes not treated with IL-1β or astrocytes cocultured with non-activated T-cells. This is relevant to Page10, lines 47-49.

Page 7, line 54: The authors say "astrocytic JAM-A facilitated CD4+ immune cell infiltration past the glia limitans and into the CNS parenchyma in cortical AdIL-1induced lesions." The authors show in Figure 3 that JAM-A KO have more CD4+ cells colocalizing with the vessels. They do not quantify the number of CD4+ cells in the parenchyma, hence they can not draw the conclusion stated. The authors will need to quantify the number of CD4+ cell in the parenchyma of AdIL-1 injected animals. This is relevant to Page 11, line 20. It is only shown in one model, not two.
Moreover, in Figure 3, how do the authors know that the CD4+ cells are in the perivascular spaces and not inside the vessels?

Figure 4: at least for the analyses on panels M-R, a non EAE control should be included to be able to compare to baseline levels.

Page 10, lines 13-18: The authors say "we demonstrate a novel astrocyte-T cell interaction that modulates lymphocyte trafficking into the CNS parenchyma with downstream effects on histopathological damage and clinical disability." Astrocyte-T cell interactions are not shown in vivo in this manuscript. This statement should be rephrased, or experiments performed to show that is the case.

Minor points
Figure 1 and Page 6: Aquaporin 4 staining would reinforce the point that JAM-A expression is increased in astrocyte endfeet.
Figure 1 and Page 6: It would be interesting to know what cells are infected with AdIL-1 and express IL-1β.
Page 6, line 52: Since the efficacy of siJAM-A is not shown in this manuscript it would be useful for the readers to know that it is shown in Horng et al 2017. It would be good to reference it here.
Page 7, line 43: The authors should specify what CKO and WT is referring to.
Figure 3: Could the authors indicate if the n numbers refer to number of mice or something else?
Figure 4N: The authors should include the units.
Page 9, line 20: The authors should refer to changes in immunohistochemical staining rather than

decreased demyelination, neuronal cell death and astrocytic activation. This could be an overinterpretation, e.g. NeuN expression could be reduced without neuronal cell death.

Figure 4: The authors should indicate the timepoint at which the mortality differences were assessed.

Page 11, line 27: The manuscript would benefit of quantification of potential changes of expression of JAM-A in endothelial cells in the CKO.

Reviewer: 2

Comments to the Author
This is an interesting study focusing on an important aspect of neuroinflammatory disease pathology, namely the step-wise process of T cell infiltration into the CNS parenchyma, and in particular the last step of this process, the crossing of the glia limitans. However, the study seems to make leaps between almost preliminary in vitro associations and true mechanistic links between a proposed hypothesis and an established and well supported body of evidence. Some key concerns are listed below:
Some paragraph titles are overstating biological significance indicative of in vivo findings, based solely on marginal differences detected with a minimal number of in vitro experiments. Similarly, summarizing sentences also over-interpret results shown with often marginal differences or trends, and certainly not well substantiated by the data presented. Overall, other than the limited in scope and importance in vitro co-culture experiments showing changes in some inflammatory mediators, no real link is established showing how / when astrocytic JAM-A promotes T cell infiltration on the cellular or molecular mechanism level.
The reduced clinical severity conferred by either the genetic mutation or the pharmacological treatment is significant, but does not seem to justify statements such as "protects against clinical disability" when mice seem to get peak scores between 2.5-3.
Per unit area measurements need to be performed by using actual tissue area measurement tools and not be deduced as per "20x or 40x field" as individual pictures can include empty spaces (devoid of tissue material) of variable sizes.
How tissues were sampled across the relevant affected lesions or perilesional areas needs to be better described. If experiments and analyses were performed in a blinded or unblended manner and how data were compiled and checked for reproducibility need to be better described.
The type of tissue shown and anatomical location should be specified in the legends for all histology shown in all figures.
Figure 1: Images are not very conclusive as it is hard to appreciate if there is tight junction co-localization with the CCL2 addition or not (seems absent in fig1A) but the lack of precise colocalization analysis done per se makes any conclusion seem random. Proper statistical analyses need to be performed and described.
Fig. 1 and Suppl. Figs 1 and 2: No quantification, no statistical analyses. What stage of EAE and clinical score were these analyses performed at?
Fig 1 and S2: why is there so little JAMA in the EAE images? If it is such an essential mediator of CNS infiltration, shouldn't it be detected in abundance at least within CNS lesions?
Fig. 2: Three biological replicates likely from the same single experiment seems limited, especially given the size of the error bars for several markers. What do error bars represent? The statement that statistical comparisons were only performed on those comparisons that had visually detectable differences sounds subjective and raises doubts about the selection process and the statistical methodology employed. The presentation of the figure could be much improved over its current lab meeting layout.
Fig 3: Why are there more CD4 T cells in the JAMA cKO mice?
How is the PVS determined based on these pan-lamining staining patterns that show no perivascular cuff? Far better histological, imaging and/or other complementary methods would be required to establish that the claims made regarding exact localization of cells relative to the glia limitans are anatomically accurate.
What measures were taken to ensure sections analyzed where at the epicenter of the needle path (namely that the difference or trend seen between the two groups was not due to different distance from the needle path?) were serial sections analyzed?
Fig 4: How were the average EAE score, mortality, and disease free values calculated?
Why are there almost no cuffs in WT EAE mice at peak disease?
Day 5 from onset likely corresponds to peak EAE? That is a time point at which one expects low numbers of encephalitogenic T cells in the spleen as they are mostly already in or on their way into the CNS.
J: 81.69% for both conditions shows no trend.
Results speak of CD3 and CD4 cell comparisons by flow, but figure legend speaks of CD3, CD4 and CD8 comparisons but they are not shown in the figure.
The figure shows a massive difference in demyelination between WT and cKO spinal cords, but the quantification does not give the impression that the examples shown are indeed representative of the small difference actually quantified.

The NeuN images shown are clearly acquired at a different magnification therefore the comparisons shown on "representative images" is misleading.
Non parametric statistical tests should perhaps be considered for EAE clinical curves among non –equal groups.
Although commonly and casually used, "ug" is not a proper prefix symbol referring to an international unit of mass and thus should not be used in a scientific publication.

Editorial Office comments:

-Please supply a graphical abstract. A graphical abstract is a visual representation of the central finding or methodology of the paper. It will be displayed in the table of contents. Authors should strive to make the graphical abstract informative, interesting, visually appealing, and straightforward (no caption needed, no abbreviations except gene names). Please upload as a separate high resolution image file (tiff preferred). The technical requirements for the graphical abstracts are:
 --size of the submitted image: 1200 pixels square at 300dpi
 --font of the text: Arial 12-16 point (smaller fonts will not be legible online)

-Please add an Abbreviated Summary (up to 50 words) of your paper that captures the main purpose and conclusions of the work. The summary should state the main results written in the third person ('[1st author surname] et al. report that …') and conclusion. This summary will be used in the contents list online and should not contain any abbreviations other than those on the accepted abbreviations list and accepted gene/protein names, making it accessible to the non-specialist. Please upload it as a Word document. Allowed abbreviations: AIDS; ANOVA; AMPA; ATP; A,T,C,G; CNS; CSF; CT; DNA; ECG; EEG; EMG; GABA; HIV; MRI; NMDA; PET; PCR; RNA.

-Fig.2: Full- size and uncropped blots/gels are to be made available as Supplementary material.

- Fig2: please provide the number of data points in each group and if the number is <10 show the single data-points.

- Fig3b,d,f and Fig4g-l,n,p,r: can you please specify in the corresponding figure legend, how many cells/mouse were analysed?
