# Supplementary material for: Astrocytic junctional adhesion molecule-A regulates T-cell entry past the glia limitans to promote central nervous system autoimmune attack
Source: Brain Commun. 2022 Feb 18;4(2):fcac044. doi: 10.1093/braincomms/fcac044 (PMC8899531; doi:10.1093/braincomms/fcac044)
Supplement: fcac044_Supplementary_Data [file Revision 1- Author's Response.pdf › Revision 1- Author's Response.pdf]

We thank both reviewers for their second round of comments and suggestions. To address these concerns, we have conducted additional *in vitro* experiments and provided clarifying edits to the text and figures as outlined below.

We have added an additional author, Jorge Villavicencio, to the manuscript as he assisted with the analysis of these additional *in vitro* experiments and his contribution was critical to the validation and interpretation of these studies. Jorge additionally reviewed, provided edits and approved of the final manuscript.

Reviewer(s)' Comments to Author:

Reviewer: 1

Comments to the Author
I thank the authors for considering my comments. The manuscript has greatly improved and the authors have addressed my concerns. I think this manuscript will be a valuable addition to the journal.

We appreciate Reviewer 1's approval of the revised manuscript.

Before publication, I have some minor comments that have arisen after reading the modified version. I would strongly recommend the authors to address them before publication:

- The graphical abstract would improve by adding a title to indicate what the left and right schematics represent. In addition, based on the data, astrocyte endfeet shouldn't express JAM-A in control conditions.

We have modified the graphical abstract, adding a title as suggested and clarifying what the left and right schematics represent (early and late inflammatory lesion formation, respectively). Because the left schematic depicts an early timepoint in inflammatory lesion formation, the astrocyte endfeet do express JAM-A at this point. We have additionally added a third panel to illustrate the late inflammatory time point in JAM-A CKO animals and the histopathological changes found in this model compared to controls.

- Throughout the manuscript, including result titles, summaries and discussion, the authors state the astrocyte JAM-A promotes T-cell parenchyma invasion via MMP2. This is not demonstrated in this manuscript. The authors show that MMP2 is downregulated in absence of astrocyte JAM-A. This does not demonstrate that the JAM-A effect on T-cells goes through MMP2, it rather shows that both alterations occur in the CKO, there is no demonstration of causality. The authors should rephrase those statements.

We have revised the language throughout the manuscript to clarify that JAM-A knock-down is associated with both decreased levels of MMP-2 and milder histopathology in EAE although a direct causal link has not yet been demonstrated.

- Page 6, line 54: Figure callings are mistaken. It should be Figure 1D and Sup Fig 1D.

Thank you for pointing out this error; it has been corrected.

- Page, lines 31-34: The authors state "IL-1β expressing adenovirus (AdIL-1), reactive astrocytes expressed JAM-A, most prominently within the astrocytic processes known to encircle blood vessels, as identified using aquaporin-4 (AQP4) staining (Figure 1G, supplemental Figure 2A, B)." The data does not support this statement. In the AdIL-1 model, JAM-A colocalizes with GFAP, there is no evidence for it to be expressed around the vessels. Sup Fig 2A,B is from EAE mice. In addition, there is no quantification that supports this statement.

We have updated the text and figures to specify that JAM-A co-expression in AQP4+ endfeet was measured in EAE only and not in AdIL-1 expressing cortical lesions. We also point out that JAM-A CKO mice appeared to have *qualitative* decreases in astrocytic JAM-A expression within AdIL-1 lesions: *"Conditional knock-out mice for JAM-A in astrocytes (mGFAP:CreJAM-A$^{fl/fl}$, CKO) appeared, at a qualitative level, to not express astrocytic JAM-A 7 days after the intracortical injection of AdIL-1 (Figure 1G)."*

- Page 9, line 45-55: In CKO CD4+ cells were not "restricted" to the NVU as the authors describe. CD4+ cells were more abundant in the NVU. In fact, Figure 3G shows that ~50% of the cells in CKO are in the parenchyma. It would also be important to consider that given that the total number of CD4+ cells is higher in CKO, it is possible that the absolute number of CD4+ cells in the parenchyma is the same between WT and CKO in the AdIL-1 model.

We agree that the CD4+ cells are not "restricted" to the blood brain barrier in the CKO as some cells do access the CNS parenchyma. We have now clarified that in CKOs, a higher proportion and total number of cells are found within the blood-brain barrier versus the parenchyma. As suggested by the reviewer, we performed an analysis of the absolute number of CD4+ cells in the parenchyma and found, as predicted, that this number was the same between WT and CKO, indicating that despite an accumulation of cells within the blood-brain barrier in CKOs, an equivalent number of cells manages to infiltrate the parenchyma, which may explain the similar cortical lesion sizes found in WT and CKO mice.

- Page 11, line 15: the authors say "immune cells were found…". Due to the technical limitations the authors highlighted in the response to the reviewers, it can't be concluded that those Dapi+ cells are immune cells. I suggest to remove the word "immune" from this sentence.

We have replaced "immune cells" with "infiltrating cells" in the text and figure legend.

Reviewer: 2

Comments to the Author
The revised manuscript is certainly improved, particularly because of the quantification of the data. And the addition of technical details and further explanations in Methods and legends. However, several significant issues remain and are listed below:

We appreciate Reviewer 2's comments and helpful suggestions for the revised manuscript.

The replacement of anatomical descriptors of the compartments through which infiltrating immune cells have to migrate with the term "neurovascular unit" was not a good choice. The NVU term is (and should remain) a more a functional one than an anatomical one, and it usually refers to how neuronal input affects blood flow and supply to a particular brain region and the opposite, how changes in vascular properties that influence blood supply affect neuronal function. It has been used also as a general term to include all the cellular components of the BBB and the neurons that signal to them, but in cases where one needs to refer purely to anatomical components of the BBB and the neuronal relationship is completely irrelevant (as is the case in this study), it would be much preferred to use terms that clearly specify the anatomical layer when is referring to. Such as the BBB if talking about all layers, the basement membranes, the perivascular cuff, etc. Moreover, the term perivascular space does not refer to the cuff only, but to any space around a blood vessel, both within the cuff and outside of it.

We agree with the reviewer's concern that NVU may cause confusion as a functional rather than neuroanatomical term. We have replaced NVU with blood-brain barrier (BBB) throughout the manuscript and in the figures.

Fig. 1: The timepoints can be shown in a more straightforward way than listed inside the merged images at the bottom row, to communicate this information to the reader easier.

We have now labeled the time point of 24 hours above Figure 1 for clarity.

Fig 1D and E are not called out in the manuscript.

We have now referenced Figure 1D correctly in the manuscript text. We removed the old Figure 1E, which had compared co-localization of occludin and JAM-A at 6 hour and 24 hour IL-1 timepoints. We focused on the 24 hour timepoint data in the updated manuscript in order to perform additional *in vitro* experiments for the 24 hour timepoint that would meet the number of technical and biological replicates recommended by the reviewer and our own biostatistics core.

The picture quality is low for both figure 1 and figure 5,
In figure 1 the arrows cannot be seen well, there are no white double headed arrows on Fig 1A, they are yellow, and not correctly positioned

We have improved the picture quality of images in Figure 1 (A, which now includes new images from additional *in vitro* experiments, and G) and Figure 5 (A and B). In Figure 1A, yellow double headed arrows refer to overlap of JAM-A (green) and occludin (red) positive pixels. These specifications are updated in the Figure legend.

Fig 1F does not show vascular pattern for JAM-A, it just shows no overlap with GFAP.

We have qualified the description of this image in the new Figure 1E (former Figure 1F) as follows: *In resting (i.e. healthy) cortex, JAM-A was not expressed in astrocytes of the CNS parenchyma (identified using GFAP staining) but it was expressed in other cell types (Figure 1E) in a pattern consistent with previous work demonstrating JAM-A expression on the vascular endothelium."*

Fig 1H does not show healthy spinal cord. It also does not show leukocytes by the use of any leukocyte marker, the asterisks are marking cells that may be leukocytes indeed, but those are only shown in Supplemental fig. 3. One cannot make definitive claims when they are simply guessing.

We removed the spinal cord EAE images of the former Figure 1H, because similar information and additional quantitative analysis is provided by the current Figures 1G, 1H and Supplemental Figure 2.

The labeling of the figures is un-necessarily confusing. Example: It is unclear how Fig 1G and 1H are different. The top row seems like it is brain tissue while the bottom (I) is spinal cord, but apparently the G panel is also showing spinal cord.

As noted above, we have removed the former Figure 1H. For clarity, the new panel of Figure 1 is now organized as follows: Fig. 1A, B → cell cultures, Fig. 1E, F → cortex (AdIL-1 lesions) and Fig. 1G, H → spinal cord (EAE lesions).

Supplemental Fig 2A,B should not be listed in the same sentence that speaks of cortex in the adenovirus injection model, cause it shows expression in spinal cord.

Discussion of the new Supplemental Figure 1A, B (former Supplemental Figure 2A, B) has been corrected in the text and now only refers to EAE data.

For figure 3 and other analyses where the anatomical location of cells relative to components of the BBB is critical, it is not clear if these analyses were performed using a confocal microscope and appreciating the location of the cells by looking through the planes of each z stack or if it was done in z-projections of such acquired stacks or, using an epifluorescence scope with poor z-resolution. Cells above or below a vessel will artificially look yellow (therefore could easily appear to "colocalize" as described in the figure legend) in projections and can easily be mis-allocated as within vessels or the perivascular cuff, instead of outside in the parenchyma where they may truly be. The description in the figure 3 legend would imply this analysis was done using co-localization tools, which is not appropriate.

In the Methods ("*Immunohistochemistry*"), we specify that all images were taken as Z stacks in confocal microscopy. We agree that for Figure 3 where anatomical localization of cells relative to the laminin staining of the perivascular basement membrane is critical, confirmation of overlap through analysis through each z-level rather than a summed z-projection is necessary to classify a cell as localized to the BBB vs. parenchyma. We performed a reanalysis of these data through each z-level and adjusted our findings accordingly and this approach has now been specified in the methods ("*Morphometric Analysis*").

Also, DAPI does not “demarcate” the parenchyma, it binds to chromatin and thus is a nuclear marker.

We have corrected the reference to DAPI as a nuclear marker rather than a demarcation of the parenchyma in the text.

Also, the legend that likely describes the range of cells counted states “1-10 cells were analyzed”, and that reads like one cell was looked at in one of the conditions. The point of the comment in the prior review was not about giving the range of cells, as this can be seen in the graph more or less. It was about making sure the analyses for all figures were performed using sufficient sampling of the tissue across the length of the spinal cord or if specific brain or spinal cord locations were chosen for the analyses, those to be stated, as they now are in the methods.

We thank the reviewer for the clarification and have now deleted the details about the number of cells analyzed from the Figure 3 legend. We have specified the location, number of tissue sections, selection of representative samples and number of animals of each analysis for experiments in Figures 3 through 6.

Figure 4: The legend is confusing in stating a minimum of 8 mice per group per experiment but listing way more mice in total. Are the numbers correct?
Also, total N numbers are not provided for the I panel. It is also confusing to use 2 statistical tests for the same graph, the authors should consult with a biostatistician and pick an appropriate method to perform the statistical comparisons.

The numbers are correct. The graph shows data combined from 2 to 3 EAE experiments and each experiment had a minimum number of 8-9 mice per group. These details are further clarified in the legend for Figure 4. Total n numbers are now provided for Figure 4I. We have reviewed our statistical analyses with our department’s biostatistics core and have now applied a consistent approach to our statistical comparisons using non-parametric tests to compare EAE scores between groups as described in the methods (“Statistics”).

Figure 5: The DAPI stain that is supposed to show the immune cells localization is not visible in these images and it needs to either be brightened up substantially or better yet be shown in a different color.

We have now improved the quality and increased the intensity of the DAPI signal in Fig.5A.

Also, the top panel of Fig5A shows a large number of DAPI stained cells in the SC of the cKO mice. It does seem like the cuffs are larger in these mice, but there clearly is a large number of cells that do infiltrate, which likely explains the substantial disease these mice get. This needs to be at least acknowledged in the discussion.

We have added the following discussion: *“Collectively, we show that astrocytic JAM-A is a key component for the effective trafficking of T cells out of the PVS and into the CNS parenchyma. However, astrocytic JAM-A is likely not the only player involved in this*

process since its deletion in astrocytes did not entirely stop T cells from crossing the glia limitans in CNS autoimmune lesions."

Fig5C the scale used for the Y axis is very hard to appreciate since it shows the number of cuffs as 0.000001/µm2. That means 1 cuff every one million square micrometers or 1 square millimeter? It seems extremely low somehow, so the authors should check these numbers again.

We have confirmed these numbers. Approximately 1-2 cuffs per 1 square millimeter is the typical frequency of viewing a perivascular cuff on an axial EAE spinal cord cross section.

Fig 5D is listed as Fig5C on the legend.

Figures 5C and 5D are now correctly annotated in the legend.

Fig 6C: This is an unconventional way of quantifying neuronal damage in EAE. Typically axonal damage is quantified in affected white matter, and not neuronal cell bodies in the grey matte,r but nothing wrong with this analysis. It is not clear though what the white boxes show and what the statement in the legend that only a "range of x number of cells" were analyzed? Were cell bodies selected? Or were total numbers of NeuN positive neurons counted per horn as the description in the methods would imply?
Similarly what the box shows for 6E is not explained in the legend.

In the original Figure 6, the white box referred to the region of analysis for NeuN and GFAP measurements. For a more robust analysis specified by anatomical landmarks, we discarded the white box and reanalyzed NeuN measurements within the entire anterior (ventral) horn and GFAP measurements within the entirety of the anterolateral white matter tract. Our new data showed similar results to the previous analysis and are now reported in the updated Figure 6.

The quantification and statistical analyses are not done properly. Consulting with a biostatistician would be very helpful to appreciate and use proper replicates and perform statistics on appropriate N numbers for both in vitro and in vivo experiments. For example, for the in vitro experiments: If different FOVs have to be taken per well to account for variability in each cell, then these should be quantified and averaged to contribute 1 value from each such measure. If multiple wells are done per condition on the same day, those averaged image values from each individual well should be averaged as well, to provide the mean of each repeat measure for that condition. Ideally, a minimum of three replicates for the entire experimental comparison (all conditions) should be performed on different days for all in vitro experiments to be properly powered. Then statistics should be performed on the average values from the different replicates (performed on the different days) which should count as the single N values (3 if the minimum number of replications is chosen) for statistical comparisons. Grouping the individual FOV image measurement values and counting them as distinct replicates adding to a total N number from a few wells (or even from actual replicates performed on different experiments) and then for statistics to be performed on this pseudo-N number, is not right, as these are not proper replicates for in vitro experiments.

We consulted with our department's biostatistics core to evaluate the sample size and statistical analyses used throughout our study. Where needed, the statistical tests were changed and the different statistical approaches are described in the Methods ("Statistics") and in the figures' legends. We agree that the *in vitro* experiments needed additional technical and biological replicates for a more robust analysis. We focused on the 24 hour time point and performed new experiments replicating the experimental design of the original Figure 1A-D with the only difference that the new images were acquired using a 40x objective with 6x zoom, as the 100x objective used in the previous Figure 1 A-D was damaged in our core facility and unavailable for use at the time of revisions. For the new experiments, we used 3 images for each of 3 technical replicates for each of 3 biological replicates of the 4 treatment groups (vehicle, CCL-2, IL1-β, CCL-2+IL1-β). We found similar results to our previous analysis and these data are reported in the updated Figure 1.

Similarly for in vivo experiments, 2 images analyzed per animal cannot possibly provide sufficient sampling for the entire brain or spinal cord. More images need to be sampled, how they are selected needs to be explained and if values are averaged needs to be stated clearly to give full clarity on what values are used for N numbers and statistics.

We consulted with our department's biostatistics core and reviewed our analysis with their team to confirm that our approach is statistically sound. For neuropathological analyses in AdIL-1 and EAE we focused on cross-sectional images taken in representative areas of the disease models rather than sampling the entire brain or spinal cord. For AdIL-1 lesions, we do believe that 2-4 representative images per animal provide sufficient sampling of disease pathology given the consistency and size of lesions in this model. AdIL-1 lesions were on average 335 and 304 µm in maximal width and 1.6 and 1.2 mm^2 in maximal cross sectional area for WT and CKO animals, respectively. We used at least 2 sections (25 µm thick acquired in z-stack) per animal located within the center of the cortical lesion. For the neuropathological analyses in EAE, where lesions typically extend the length of the lumbar cord, we used at least 3 lumbar spinal cord sections per animal consistent with other studies[1,2,3]. For colocalization analyses of JAM-A with other markers of CNS cell types, we used at least 2 images of consecutive lumbar cross-sections of inflammatory lesions in the dorsal and ventral white matter for each animal. Given the small inter- and intra-animal variation of JAM-A levels within groups, we do believe that this approach provided sufficient sampling. For all analyses, images per animal were averaged and data from each animal was used for the N numbers and statistics.

1. **Castro K *et al.*. *Body Mass Index in Multiple Sclerosis modulates ceramide-induced DNA methylation and disease course*. EBioMedicine. 2019 May; 43:392-410.**
2. **Koda T *et al.*. *Sema4A inhibits the therapeutic effect of IFN-β in EAE*. Journal of Neuroimmunology. 2014 March;268:43-49**
3. **Samus M *et al.*. *Actin-Binding Protein Cortactin Promotes Pathogenesis of Experimental Autoimmune Encephalomyelitis by Supporting Leukocyte Infiltration***

***into the Central Nervous System*. Journal of Neuroscience. 2020 February; 40 (7) 1389-1404**

The reduction in JAMA signal from the GFAPcre cKO mice is suboptimal, as one might actually expect given prior reports.

Reduction of JAMA in astrocytes was measured in this study using co-localization analysis of GFAP and JAM-A immunohistochemical stains in EAE tissues. We found at least 50% decrease in astrocytic JAM-A using this technique. We suspect our immunohistochemical measurements overestimate the expression of astrocytic JAM-A in the CKO because 1) there may be reactive GFAP production by non-astrocyte cells expressing JAM-A and 2) imaging analysis may include areas of overlap between astrocytic GFAP and neighboring non-astrocyte JAM-A producing cells (such as endothelium, microglia and infiltrating inflammatory cells), as shown in Supplemental Figures 1, 2 and 3. More sensitive measurements using cell sorting of transgenically tagged GFAP positive cells and RT-PCR amplification of JAM-A may give more accurate values, although this approach is labor intensive, requires the creation of an additional genetic mouse line and lies beyond the scope of the current study.

Terms referring to phenomena like immunohistochemistry and immunofluorescence should not be used in plural.

The terms immunohistochemistry and immunofluorescence have all been adjusted to the singular.

The discussion states that the effect of JAMA in EAE is mediated through MMP2. Though some evidence is shown for differential levels of MMP2 between the WT and cKO mice, and with the in vitro siRNA experiment, the link is far from definitively established, so the statement should be toned down accordingly.

As also suggested by Reviewer 1, we have toned down any direct or exclusive attribution of the JAM-A CKO clinical and histopathological phenotype to decreased levels of MMP2.

The peptide treatment showed a small but significant amelioration of disease course, but it did not protect from disease.

We replaced "*protected*" with "*ameliorated.*"

Editorial Office comments:

-Please supply a graphical abstract. A graphical abstract is a visual representation of the central finding or methodology of the paper. It will be displayed in the table of contents. Authors should strive to make the graphical abstract informative, interesting, visually appealing, and straightforward (no caption needed, no abbreviations except gene names). Please upload as a separate high resolution image file (tiff preferred). The technical requirements for the graphical abstracts are:

--size of the submitted image: 1200 pixels square at 300dpi
--font of the text: Arial 12-16 point (smaller fonts will not be legible online)

A graphical abstract meeting the technical requirements above is included in the current manuscript after the title page and a separate .tiff file has been uploaded.

-Please add an Abbreviated Summary (up to 50 words) of your paper that captures the main purpose and conclusions of the work. The summary should state the main results written in the third person ('[1st author surname] et al. report that ...') and conclusion. This summary will be used in the contents list online and should not contain any abbreviations other than those on the accepted abbreviations list and accepted gene/protein names, making it accessible to the non-specialist. Please upload it as a Word document. Allowed abbreviations: AIDS; ANOVA; AMPA; ATP; A,T,C,G; CNS; CSF; CT; DNA; ECG; EEG; EMG; GABA; HIV; MRI; NMDA; PET; PCR; RNA.

An abbreviated summary is included in the current manuscript after graphical abstract and a separate word file has been uploaded.
