# Supplementary material for: Astrocytic junctional adhesion molecule-A regulates T-cell entry past the glia limitans to promote central nervous system autoimmune attack
Source: Brain Commun. 2022 Feb 18;4(2):fcac044. doi: 10.1093/braincomms/fcac044 (PMC8899531; doi:10.1093/braincomms/fcac044)
Supplement: fcac044_Supplementary_Data [file Revision 1-Decision Letter.pdf › Revision 1-Decision Letter.pdf]

Revision 1-Decision Letter

02-Aug-2021

Dear Dr. Horng,

Thank you for your recent submission BRAINCOM-2020-359.R1
entitled "Astrocytic JAM-A Regulates T Cell Entry Past the Glia Limitans to Promote CNS Autoimmune Attack" to Brain Communications.

Your manuscript has been reviewed, and there are still some remaining concerns. Pending minor revisions to satisfy these final requests and to comply with our editorial and formatting guidelines.

The reviewer(s)' comments are included at the bottom of this message.

In addition to the reviewers' comments, please note our editorial comments added below to comply with our requirements for presentation of scientific data at Brain Communications.

Please format your revised manuscript according to our Instructions to Authors https://academic.oup.com/braincomms/pages/General_Instructions – a summary of key points is attached to help with this.

To revise your manuscript, click the link below or log into https://mc.manuscriptcentral.com/braincom and enter your Author Centre, where you will find your manuscript title listed under "Manuscripts with Decisions." Under "Actions," click on "Create a Revision." Your manuscript number will be appended to denote a new revision.

*** PLEASE NOTE: This is a two-step process. After clicking on the link, you will be directed to a webpage to confirm. ***

https://mc.manuscriptcentral.com/braincom?URL_MASK=8e425b651d504fcdaaa1328e6a7ffcca

Please upload a revised manuscript with changes tracked using track changes or in coloured text.

When submitting your revised manuscript, you will be asked to respond to the reviewer(s) comments. Please be as specific as possible in your response to all of the comments.

The editorial team at Brain Communications also aims to increase transparency in scientific publishing and to demonstrate the value that peer review brings to the scientific process. Please therefore consider whether you are willing to have reviewer comments on your paper and your response published as supplementary data with the paper if it is accepted. This is entirely optional and will only be done if both the authors and reviewers agree. When submitting your revision you will have the opportunity to update the answer you entered previously.

IMPORTANT: Your original files are available to you when you upload your revised manuscript. Please delete any redundant files before completing the submission.

Because we are trying to facilitate timely publication of manuscripts submitted to Brain Communications, we would appreciate your revised manuscript as soon as possible. If it is not possible for you to resubmit within 3 months, the option to submit a revision will expire, and we would then consider your revised paper as a new submission. However, if you need a little more time, please get in touch and we can extend this deadline for you.

Once again, thank you for submitting your manuscript to Brain Communications and I look forward to receiving your revision.

Best wishes

Dr. Ragnhildur Káradóttir
Associate Editor, Brain Communications

Professor Tara Spires-Jones
Founding Editor, Brain Communications

Reviewer(s)' Comments to Author:

Reviewer: 1 (Blanca Diaz-Castro)

Comments to the Author
I thank the authors for considering my comments. The manuscript has greatly improved and the authors have addressed my concerns. I think this manuscript will be a valuable addition to the journal.

Before publication, I have some minor comments that have arisen after reading the modified version. I would strongly recommend the authors to address them before publication:
- The graphical abstract would improve by adding a title to indicate what the left and right schematics represent. In addition, based on the data, astrocyte endfeet shouldn't express JAM-A in control conditions.
- Throughout the manuscript, including result titles, summaries and discussion, the authors state the astrocyte JAM-A promotes T-cell parenchyma invasion via MMP2. This is not demonstrated in this manuscript. The authors show that MMP2 is downregulated in absence of astrocyte JAM-A. This does not demonstrate that the JAM-A effect on T-cells goes through MMP2, it rather shows that both alterations occur in the CKO, there is no demonstration of causality. The authors should rephrase those statements.
- Page 6, line 54: Figure callings are mistaken. It should be Figure 1D and Sup Fig 1D.
- Page, lines 31-34: The authors state "IL-1β expressing adenovirus (AdIL-1), reactive astrocytes expressed JAM-A, most prominently within the astrocytic processes known to encircle blood vessels, as identified using aquaporin-4 (AQP4) staining (Figure 1G, supplemental Figure 2A, B)." The data does not support this statement. In the AdIL-1 model, JAM-A colocalizes with GFAP, there is no evidence for it to be expressed around the vessels. Sup Fig 2A,B is from EAE mice. In addition, there is no quantification that supports this statement.
- Page 9, line 45-55: In CKO CD4+ cells were not "restricted" to the NVU as the authors describe. CD4+ cells were more abundant in the NVU. In fact, Figure 3G shows that ~50% of the cells in CKO are in the parenchyma. It would also be important to consider that given that the total number of CD4+ cells is higher in CKO, it is possible that the absolute number of CD4+ cells in the parenchyma is the same between WT and CKO in the AdIL-1 model.
- Page 11, line 15: the authors say "immune cells were found…". Due to the technical limitations the authors highlighted in the response to the reviewers, it can't be concluded that those Dapi+ cells are immune cells. I suggest the remove the word "immune" from this sentence.

Reviewer: 2

Comments to the Author
The revised manuscript is certainly improved, particularly because of the quantification of the data. And the addition of technical details and further explanations in Methods and legends. However, several significant issues remain and are listed below:
The replacement of anatomical descriptors of the compartments through which infiltrating immune cells have to migrate with the term "neurovascular unit" was not a good choice. The NVU term is (and should remain) a more a functional one than an anatomical one, and it usually refers to how neuronal input affects blood flow and supply to a particular brain region and the opposite, how changes in vascular properties that influence blood supply affect neuronal function. It has been used also as a general term to include all the cellular components of the BBB and the neurons that signal to them, but in cases where one needs to refer purely to anatomical components of the BBB and the neuronal relationship is completely irrelevant (as is the case in this study), it would be much preferred to use terms that clearly specify the anatomical layer when is referring to. Such as the BBB if talking about all layers, the basement membranes, the perivascular cuff, etc. Moreover, the term perivascular space does not refer to the cuff only, but to any space around a blood vessel, both within the cuff and outside of it.
Fig. 1: The timepoints can be shown in a more straightforward way than listed inside the merged images at the bottom row, to communicate this information to the reader easier.
Fig 1D and E are not called out in the manuscript.
The picture quality is low for both figure 1 and figure 5,
In figure 1 the arrows cannot be seen well, there are no white double headed arrows on Fig 1A, they are yellow, and not correctly positioned
Fig 1F does not show vascular pattern for JAM-A, it just shows no overlap with GFAP.
Fig 1H does not show healthy spinal cord. It also does not show leukocytes by the use of any leukocyte marker, the asterisks are marking cells that may be leukocytes indeed, but those are only shown in Supplemental fig. 3. One cannot make definitive claims when they are simply guessing.
The labeling of the figures is un-necessarily confusing. Example: It is unclear how Fig 1G and 1H are different. The top row seems like it is brain tissue while the bottom (I) is spinal cord, but apparently the G panel is also showing spinal cord.

Supplemental Fig 2A,B should not be listed in the same sentence that speaks of cortex in the adenovirus injection model, cause it shows expression in spinal cord.

For figure 3 and other analyses where the anatomical location of cells relative to components of the BBB is critical, it is not clear if these analyses were performed using a confocal microscope and appreciating the location of the cells by looking through the planes of each z stack or if it was done in z-projections of such acquired stacks or, using an epifluorescence scope with poor z-resolution. Cells above or below a vessel will artificially look yellow (therefore could easily appear to "colocalize" as described in the figure legend) in projections and can easily be mis-allocated as within vessels or the perivascular cuff, instead of outside in the parenchyma where they may truly be. The description in the figure 3 legend would imply this analysis was done using co-localization tools, which is not appropriate. Also, DAPI does not "demarcate" the parenchyma, it binds to chromatin and thus is a nuclear marker. Also, the legend that likely describes the range of cells counted states "1-10 cells were analyzed", and that reads like one cell was looked at in one of the conditions. The point of the comment in the prior review was not about giving the range of cells, as this can be seen in the graph more or less. It was about making sure the analyses for all figures were performed using sufficient sampling of the tissue across the length of the spinal cord or if specific brain or spinal cord locations were chosen for the analyses, those to be stated, as they now are in the methods.

Figure 4: The legend is confusing in stating a minimum of 8 mice per group per experiment but listing way more mice in total. Are the numbers correct? Also, total N numbers are not provided for the I panel. It is also confusing to use 2 statistical tests for the same graph, the authors should consult with a biostatistician and pick an appropriate method to perform the statistical comparisons.

Figure 5: The DAPI stain that is supposed to show the immune cells localization is not visible in these images and it needs to either be brightened up substantially or better yet be shown in a different color. Also, the top panel of Fig5A shows a large number of DAPI stained cells in the SC of the cKO mice. It does seem like the cuffs are larger in these mice, but there clearly is a large number of cells that do infiltrate, which likely explains the substantial disease these mice get. This needs to be at least acknowledged in the discussion.

Fig5C the scale used for the Y axis is very hard to appreciate since it shows the number of cuffs as 0.000001/μm2. That means 1 cuff every one million square micrometers or 1 square millimeter? It seems extremely low somehow, so the authors should check these numbers again.

Fig 5D is listed as Fig5C on the legend.

Fig 6C: This is an unconventional way of quantifying neuronal damage in EAE. Typically axonal damage is quantified in affected white matter, and not neuronal cell bodies in the grey matte,r but nothing wrong with this analysis. It is not clear though what the white boxes show and what the statement in the legend that only a "range of x number of cells" were analyzed? Were cell bodies selected? Or were total numbers of NeuN positive neurons counted per horn as the description in the methods would imply? Similarly what the box shows for 6E is not explained in the legend.

The quantification and statistical analyses are not done properly. Consulting with a biostatistician would be very helpful to appreciate and use proper replicates and perform statistics on appropriate N numbers for both in vitro and in vivo experiments. For example, for the in vitro experiments: If different FOVs have to be taken per well to account for variability in each cell, then these should be quantified and averaged to contribute 1 value from each such measure. If multiple wells are done per condition on the same day, those averaged image values from each individual well should be averaged as well, to provide the mean of each repeat measure for that condition. Ideally, a minimum of three replicates for the entire experimental comparison (all conditions) should be performed on different days for all in vitro experiments to be properly powered. Then statistics should be performed on the average values from the different replicates (performed on the different days) which should count as the single N values (3 if the minimum number of replications is chosen) for statistical comparisons. Grouping the individual FOV image measurement values and counting them as distinct replicates adding to a total N number from a few wells (or even from actual replicates performed on different experiments) and then for statistics to be performed on this pseudo-N number, is not right, as these are not proper replicates for in vitro experiments. Similarly for in vivo experiments, 2 images analyzed per animal cannot possibly provide sufficient sampling for the entire brain or spinal cord. More images need to be sampled, how they are selected needs to be explained and if values are averaged needs to be stated clearly to give full clarity on what values are used for N numbers and statistics.

The reduction in JAMA signal from the GFAPcre cKO mice is suboptimal, as one might actually expect given prior reports.

Terms referring to phenomena like immunohistochemistry and immunofluorescence should not be used in plural.

The discussion states that the effect of JAMA in EAE is mediated through MMP2. Though some evidence is shown for differential levels of MMP2 between the WT and cKO mice, and with the in vitro siRNA experiment, the link is far from definitively established, so the statement should be toned down accordingly.

The peptide treatment showed a small but significant amelioration of disease course, but it did not protect from disease.

Editorial Office comments:

-Please supply a graphical abstract. A graphical abstract is a visual representation of the central finding or methodology of the paper. It will be displayed in the table of contents. Authors should strive to make the graphical abstract informative, interesting, visually appealing, and straightforward (no caption needed, no abbreviations except gene names). Please upload as a separate high resolution image file (tiff preferred). The technical requirements for the graphical abstracts are:
--size of the submitted image: 1200 pixels square at 300dpi
--font of the text: Arial 12-16 point (smaller fonts will not be legible online)

-Please add an Abbreviated Summary (up to 50 words) of your paper that captures the main purpose and conclusions of the work. The summary should state the main results written in the third person ('[1st author surname] et al. report that …') and conclusion. This summary will be used in the contents list online and should not contain any abbreviations other than those on the accepted abbreviations list and accepted gene/protein names, making it accessible to the non-specialist. Please upload it as a Word document. Allowed abbreviations: AIDS; ANOVA; AMPA; ATP; A,T,C,G; CNS; CSF; CT; DNA; ECG; EEG; EMG; GABA; HIV; MRI; NMDA; PET; PCR; RNA.
